# Iterative protecting group-free cross-coupling leading to chiral multiply arylated structures

**DOI:** 10.1038/ncomms11065

**Published:** 2016-04-04

**Authors:** Cathleen M. Crudden, Christopher Ziebenhaus, Jason P. G. Rygus, Kazem Ghozati, Phillip J. Unsworth, Masakazu Nambo, Samantha Voth, Marieke Hutchinson, Veronique S. Laberge, Yuuki Maekawa, Daisuke Imao

**Affiliations:** 1 Queen's University, Department of Chemistry, Chernoff Hall, Kingston, Ontario, Canada K7L 3N6; 2 Institute of Transformative Bio-Molecules (WPI-ITbM), Nagoya University, Chikusa, Nagoya 464-8602, Japan; 3 Department of Chemistry and Biomolecular Science, Faculty of Engineering, Gifu University, 1-1 Yanagido, Gifu 501-1193, Japan

## Abstract

The Suzuki–Miyaura cross-coupling is one of the most often utilized reactions in the synthesis of pharmaceutical compounds and conjugated materials. In its most common form, the reaction joins two *sp*^2^-functionalized carbon atoms to make a biaryl or diene/polyene unit. These substructures are widely found in natural products and small molecules and thus the Suzuki–Miyaura cross-coupling has been proposed as the key reaction for the automated assembly of such molecules, using protecting group chemistry to affect iterative coupling. We present herein, a significant advance in this approach, in which multiply functionalized cross-coupling partners can be employed in iterative coupling without the use of protecting groups. To accomplish this, the orthogonal reactivity of different boron substituents towards the boron-to-palladium transmetalation reaction is exploited. The approach is illustrated in the preparation of chiral enantioenriched compounds, which are known to be privileged structures in active pharmaceutical compounds.

## Abstract (web summary)

The Suzuki-Miyaura cross coupling is widely used in industrial and academic settings for the formation of carbon-carbon bonds. Here, the authors report a procedure whereby a molecule with multiple reactive carbon-boron bonds can undergo sequential, selective Suzuki-Miyaura reactions without the need for protecting groups.

## Graphical abstract

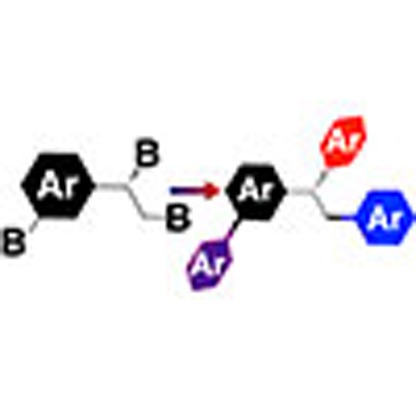

The Suzuki–Miyaura cross-coupling, in which organoboranes are coupled with organic halides or their equivalents (Fig. 1a), has changed the way organic molecules are assembled^1^,^2^. This reaction is the method of choice for the preparation of biaryl or polyene units in pharmaceutical^3^ and materials industries^4^. Because of the ease with which biaryl molecules are prepared using the Suzuki–Miyaura reaction, these substructures are now widely found in pharmaceutical products and precursors, perhaps even to the detriment of structural diversity. Indeed, evidence is emerging that ‘flat' molecules lacking stereochemistry are suboptimal drug candidates when compared with chiral molecules, which have a more complex and tunable three-dimensional shape and improved pharmacokinetic properties^5^,^6^. Molecules with chiral centres are found with significantly greater frequency (up to 30%) in final drugs than in discovery compounds, a fact attributed to improved drug performance compared with flat structures composed of sp^2^ centres^5^. Despite these compelling facts, the creation of C–C bonds with stereochemistry using the Suzuki–Miyaura reaction has only been demonstrated in the last few years, enabling the preparation of molecules with considerable diversity (Fig. 1b)^7^,^8^,^9^,^10^,^11^,^12^,^13^,^14^,^15^,^16^,^17^,^18^,^19^,^20^,^21^,^22^,^23^.

The development of methods to include the construction of chiral centres with control of stereochemistry is clearly critical to access more complex and valuable structures, and doing this in an iterative manner is a key for efficient, potentially automated applications. In fact, Burke has proposed the use of the Suzuki–Miyaura reaction as the key reaction with which the majority of non-peptidic organic molecules can be assembled in an automated manner (Fig. 1c)^24^,^25^. One critical component in any iterative synthesis involving organoboranes is the ability to modulate the reactivity of B–C bonds between ‘off' states, where no reaction takes place; and ‘on' states, from which coupling can occur. At present, this can only be accomplished through the use of blocking ligands on boron that deactivate the substrate towards coupling, which is followed by chemical removal of these ligands to generate an active-coupling partner^26^,^27^.

In a significant divergence from existing approaches, we describe herein the first example the iterative coupling of up to three B–C bonds within the same molecule without employing protecting group chemistry. This is accomplished by taking advantage of inherent differences in the transmetalation efficiency of closely related C–Bpin bonds (Fig. 1d). The orthogonal reactivity of B–C bonds in different positions in a single molecule permits the chemoselective, sequential coupling of aromatic, aliphatic and stereochemistry-bearing B–C bonds. This method permits the rapid generation of multiply arylated, chiral organic molecules with control of stereochemistry, without the need for protection/deprotection sequences. The method is simple, straightforward and holds considerable promise for the facile synthesis of new classes of pharmaceutical structures with improved properties. The Molander group has reported examples of protecting group-free iterative couplings accomplished employing the differential ability of benzylic RBF_3_K salts to undergo radical-based transmetallation^19^,^28^,^29^. Although this has not been reported on molecules containing more than one identical boron substituent, such reactivity can be envisioned.

## Results

### Orthogonal coupling of benzylic and non-benzylic C–B bonds

**Figure 2:**
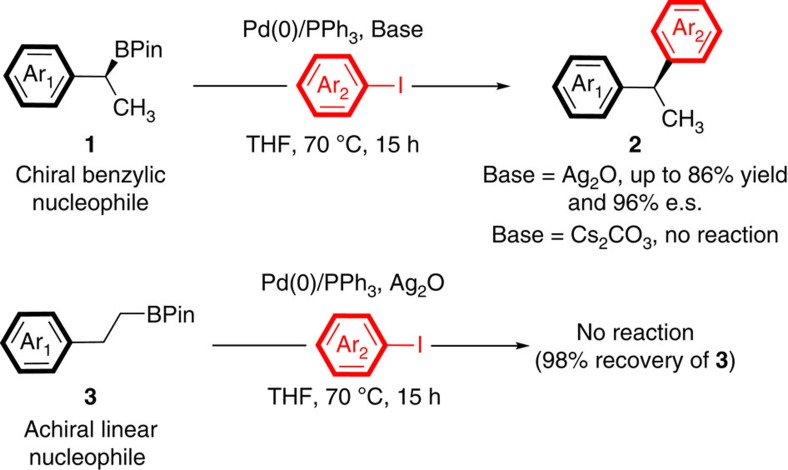
Orthogonal reactivity of branched benzylic and linear aliphatic boronic esters. Observation of complete inversion of reactivity such that branched benzylic boronic esters (**1**) react in the presence of Ag_2_O, but not carbonate bases, while linear aliphatic isomers (**3**) are recovered unchanged when treated with Ag_2_O.

In 2009, we reported the first example of the cross-coupling of chiral benzylic boronic esters (**1**) that occurred with retention of stereochemistry (Fig. 2)^9^. This method has been extended to include the cross-coupling of allylic^11^,^30^, propargylic^31^ and doubly benzylic boronic esters^14^. Using different substrates, Suginome^10^, Hall^13^, Molander^16^ and Biscoe^15^ have reported groundbreaking invertive couplings under alternative conditions.

In our 2009 report, two key observations were critical for the current work. First, we noted that when bases other than silver oxide were employed with benzylic boronic ester **1**, the starting boronic ester was recovered untouched, indicating that silver oxide was required for transmetalation. Second, under optimized conditions for the coupling of **1**, namely Pd(0)/PPh_3_ and Ag_2_O, its linear achiral isomer **3** was completely unreactive, such that **3** could be recovered from the reaction mixture with the B–C bond intact (Fig. 2a)^9^. Thus, the mild silver oxide-promoted conditions employed for branched substrate **1** were clearly insufficient to promote transmetalation of isomer **3**. This orthogonal reactivity offered the exciting opportunity to control the arylation of multiborylated organic compounds based on inherent differences in transmetalation efficiencies of different types of B–C bonds.

The critical question, then, was whether the relative reactivity described in Fig. 2 would translate to distinct B–C bonds in diborylated molecules. We first chose to examine the reactivity of 1,2 diboronates (**5**), which contain both a linear and a branched benzylic boron substituent. These compounds can be easily prepared with high enantioselectivity by diboration of the corresponding styrene derivative **4** (refs 32, 33, 34). Pinacol substituents were chosen on boron, as these are among the most common and stable ancillary groups for boron^8^,^35^. The coupling of one B–C(OR)_2_ unit in diborylated compounds followed by oxidation or, less commonly, amination or homologation of a remaining B–C bond has been amply demonstrated by Fernández^36^, Morken^37^,^38^,^39^ and Suginome^40^. However, with the exception of one example of a hydroxyl-promoted coupling^39^, there have been no reports describing the use of unprotected multiborylated compounds in iterative cross-couplings.

**Figure 3:**
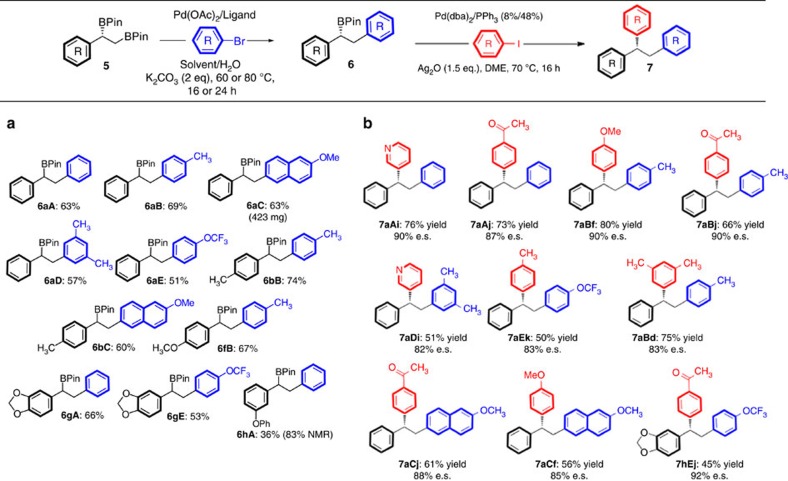
Chemoselective Suzuki–Miyaura cross-coupling of chiral 1,2-diboronic esters. (**a**) Demonstration of selective coupling of linear boronic ester, while the branched B–C bond remains intact. (**b**) Stereoretentive coupling of remaining secondary benzylic B–C bond in enantioenriched boronic esters, leading to enantiomerically enriched unsymmetrical 1,1′,2-triarylethanes with up to 92% enantiospecificity.

**Figure 4:**
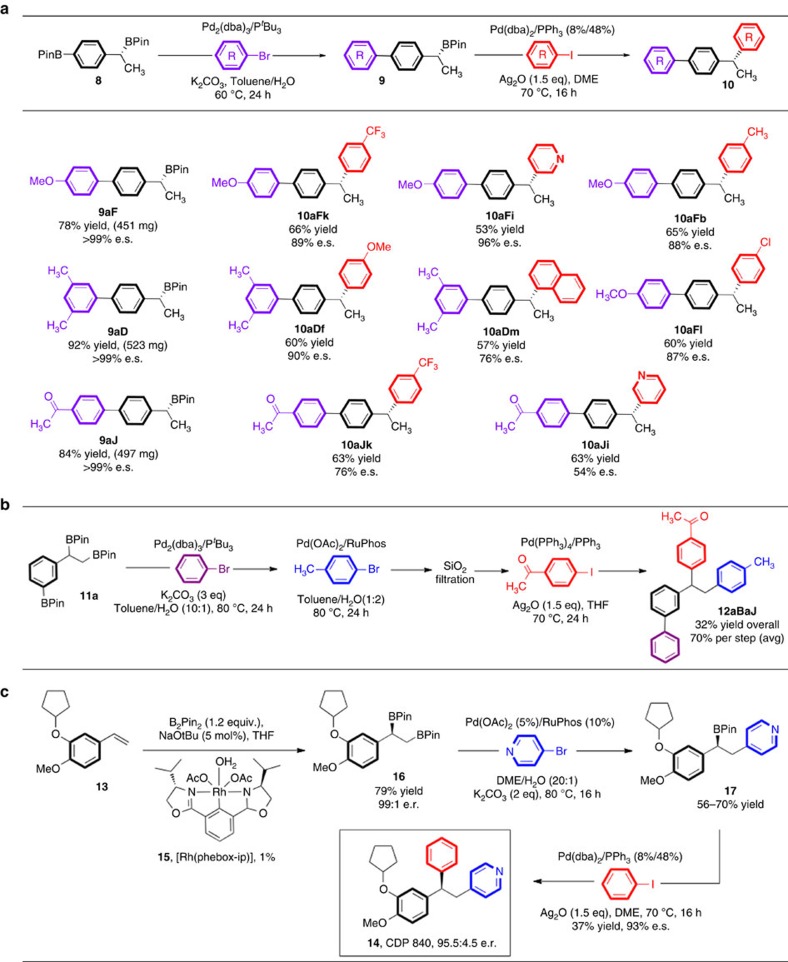
Chemoselective cross-couplings of bis- and tris-borylated chiral aromatics. (**a**) Initial selective coupling of aryl B–C bonds using Pd/P*t*Bu_3_ as the catalyst and carbonate bases, followed by second, stereospecific coupling of chiral secondary benzylic B–C bond leading to biphenyl-substituted 1,1–diaryl alkanes. (**b**) The introduction of three unique aryl groups sequentially by coupling at (1) the aryl B–C bond, (2) the linear achiral aliphatic B–C bond, and (3), the chiral benzylic B–C bond in a single pot for the first two steps, and the use of filtration through a short silica plug before the final step. (**c**) Illustration of the orthogonal coupling concept in the synthesis of phosphodiesterase inhibitor CDP 840 (**14**).

Initial attempts at reacting the benzylic, secondary B–C bond selectively in the presence of the adjacent linear B–C bond were unsuccessful, leading to a mixture of products, including those resulting from protodeboronation. However, the linear B–C bond in **5** could be enticed to undergo transmetalation and cross-coupling with aryl bromides in the presence of Pd(OAc)_2_ with RuPhos (2-dicyclohexylphosphino-2′,6′-diisopropoxybiphenyl) or SPhos (2-dicyclo-hexylphosphino-2′,6′-dimethoxybiphenyl) as ligand, and K_2_CO_3_ as base, leaving the branched B–C bond intact (Fig. 3a). Although counterintuitive, substrates showing sensitivity to deboronation benefited from the use of a significantly higher proportion of water (as seen in substrates **6aC**, **6bC**, **6gA**, **6gE** and **6hA**, and later in the linear B–C^(*sp*3)^ coupling of trisborylated substrate **11a** (Fig. 4b)). On the basis of the work of the Lloyd-Jones group^41^, this could be due to a decrease in the effective pH of the reaction medium as the proportion of water is increased. In some cases, lower yields were obtained due to losses upon chromatography, as illustrated with compound **6hA** (Fig. 3a). Under these relatively simple coupling conditions, a variety of coupling partners for the linear position were tolerated, including electron-rich, electron-poor and π-extended substrates. Heteroaromatic aryl bromides were less successful in this position, with 2-thienyl, 3-bromopyridyl and 3-bromoquinoline giving suboptimal yields, although 4-pyridylbromide could be effectively coupled as shown in Fig. 4c. In all cases studied, this reaction occurred without compromising the stereochemistry at the benzylic B–C bond.

For the next step in the iterative sequence, the branched B–C bond was coupled using our previously described silver oxide-promoted conditions (Fig. 3b)^9^. Again, electron-rich, electron-poor, π-extended and heteroaromatic aryl iodides were well tolerated as coupling partners under the conditions shown in Fig. 3b. Enantiospecificities of this reaction are on the order of 90%, which is slightly lower than those observed in couplings of simpler 1-phenethyl boronic esters^9^,^42^, possibly indicating a slightly increased susceptibility to stereochemical erosion, because of the conjugated nature of any presumed stilbene intermediate resulting from β-hydride elimination; however, no regioisomeric products were observed, and thus the possibility of loss of stereochemistry during transmetalation must also be considered (enantiospecificity, e.s.=(e.e. product/e.e. starting material) × 100%). The electronics of the aryl iodide did not appear to play any effect.

### Orthogonal coupling of benzylic sp^3^ versus sp^2^ C–B bonds

Having demonstrated that two adjacent aliphatic B–C bonds can be sequentially coupled with high chemoselectivity, we next probed whether the same approach could be used in molecules containing other types of B–C bonds with different propensities for transmetalation. Thus, we chose to pit B–C^(*sp*2)^ bonds against secondary B–C^(*sp*3)^ bonds, as in substrate **8**, which was prepared by hydroboration of 4-pinacol boronato styrene. This reaction took place with 95:5 e.r. (ref. 43). In this case, the B–C^(*sp*2)^ bond was targeted for initial coupling since it should clearly undergo transmetalation, the most readily based on >30 years of precedent. Although a number of conditions were effective for cross-coupling of this B–C bond and indeed left the secondary benzylic B–C bond untouched, small impurities derived from the incorporation of phenyl rather than aryl substituents were observed consistently. These side products originated from the use of triphenylphosphine as the ligand via well-documented P–Ph activation^44^. After several unsuccessful attempts to circumvent this unwanted side reaction, the use of P^*t*^Bu_3_ proved successful^45^, giving the desired arylated products **9aX** in high isolated yields, with the benzylic B–C bond intact for the next coupling (Fig. 4a). The reaction was readily scalable and could be routinely run on several hundred milligrams scale. Comparison of the enantiopurity of products **9aX**, after coupling at the B–C^(*sp*2)^ bond indicated, as expected, that there was no detectable loss of enantiopurity during this coupling.

With these products in hand, we next examined the scope of the Ag_2_O-promoted benzylic cross-coupling reaction (Fig. 4a). The enantiospecific cross-coupling took place as expected, except that *π*-extended biphenyl boronic esters **9aX** displayed higher sensitivity to the loss of enantiomeric purity during coupling than previously observed for simple phenylated boronic esters^9^. In particular, we noted that electron-withdrawing substituents on the biphenyl derivative as in **9aJ** led to a greater erosion in enantiospecificity (for example, 76% e.s. for coupling with 4-iodotrifluoromethyltoluene yielding **10aJk** and 54% e.s. for coupling of the same boronic ester with 3-iodopyridine yielding **10aJi**).

To confirm that this effect was due to the starting boronic ester, 3-iodopyridine and 4-iodotrifluoromethyltoluene were reacted with coupling partner **9aF**, bearing a methoxyphenyl instead of phenacyl substituent. As shown in Fig. 4a, these reactions occurred with very high enantiospecificities (96% and 89%, respectively), confirming that it is the electron-withdrawing substituent on the biphenyl that leads to higher loss of enantiopurity in the coupling. Bulky aryl iodides such as 1-napthyliodide were effective, but also resulted in decreased enantiospecificity (that is, **10aDm**). Comparing simple 1-phenethyl pinacol boronate with the π-extended systems illustrates the sensitivity of the latter systems: π-extended **9aD** reacts with 1-naphthyliodide with 76% e.s., while PhCH(BPin)CH_3_ reacts with 85% e.s. when coupled with the same aryl iodide^46^. These results will provide important information in ongoing mechanistic studies of this reaction.

### Protecting group-free iterative coupling of trisborylated species

To fully illustrate how the orthogonal reactivity of the various B–C bonds can be applied in iterative couplings, we prepared compound **11a** containing three different types of B–C bonds: a B–C^(*sp*2)^ bond, a primary B–C^(*sp*3)^ bond and a secondary B–C^(*sp*3)^ bond, and we attempted all three couplings in the same pot. For the first B–C^(*sp*2)^ coupling, Fu-type conditions^45^, previously shown to be optimal for the coupling of diborylated substrate **8**, were also effective with trisborylated substrate **11a**, giving the desired product without loss of either aliphatic pinacol boronate. The yield of this first arylation was determined to be 92–95% by NMR analysis. Optimized conditions for the second-coupling reaction involved the use of solvent with a high proportion of water (1:2 ratio of organic solvent:water) as described above. In test studies, these conditions led to NMR yields on the order of 70%, boding well for a fully iterative coupling of **11**. Indeed, we found the reaction proceeded with good yield, and importantly, leaving the benzylic B–C bond intact. Although the final benzylic cross-coupling worked well with isolated starting materials, when run in sequence, the one-pot procedure required filtration of the reaction mixture through a small silica plug, likely to deal with excess halides remaining from previous coupling reactions (Fig. 4b). In this manner, triarylated product **12aBaJ** could be obtained in 32% overall yield, representing an average yield per step of just over 70%.

### Application to compounds of medicinal importance

Finally, to illustrate the effectiveness of our orthogonal coupling methodology, we carried out the synthesis of a triarylated compound of pharmaceutical importance. Thus, compound **16** was prepared by diborylation of styrene **13** as shown in Fig. 4c, and, in only two steps, converted into biologically active derivative **14**, otherwise known as CDP 840. This compound is a prototype orally active anti-inflammatory phosphodiesterase with potential inhibitory effects against phosphodiesterase-4 (ref. 47). The key coupling step occurred with 93% e.s. giving the final product in 95.5:4.5 e.r. Although outside the scope of this paper, we envisage that our iterative coupling approach will be highly effective for the rapid preparation of derivatives of active compounds by the introduction of a series of different aryl groups at various borylated positions of a central core molecule.

In conclusion, we have shown that the resistance of certain types of B–C bonds to transmetalation, one of the key steps in the Suzuki–Miyaura reaction^48^, can be capitalized upon to develop a protecting group-free sequential cross-coupling of multiply borylated organic compounds. This approach is complementary to other approaches that involve the manipulation of the substituents on boron to control reactivity of the B–C bond. In all cases, we employed pinacol esters since these are the most versatile and readily employed type of organoboron substituent^8^,^35^. Chiral substrates are compatible with the method, leading to products with significant complexity, which are likely to provide interesting leads for pharmaceutical and medicinal applications.

## Methods

### Coupling of linear B–C^(*sp*3)^ bond in 1,2-diborylated compounds 5x

In a nitrogen-filled glovebox, diboronate **5x** (1 equiv.), bromoarene (1.2 equiv.), Pd(OAc)_2_ (0.1 equiv.), RuPhos (0.25 equiv.) and K_2_CO_3_ (1.9 equiv.) were weighed into a vial and tetrahydrofuran was added. The reaction was sealed with a septum and removed from the glovebox, and placed under a flow of argon. Degassed water was added (20:1, organic:water) and the septum was replaced with a teflon cap. The reaction mixture was sonicated for about 2 min before being stirred at 80 °C for 15 h. The reaction mixture was cooled and filtered through a plug of silica, washed through with EtOAc and concentrated *in vacuo*. Purification by column chromatography was affected as the final purification.

### Coupling of branched B–C^(*sp*3)^ bond in 1-boryl,1,2-diaryl species 6xX

In a nitrogen-filled glovebox, boronic ester **6xX** (1 equiv.), iodoarene (1.5 equiv.), Pd(dba)_2_ (0.08 equiv.), PPh_3_ (0.64 equiv.) and Ag_2_O (1.5 equiv.) were weighed into a 1 dram vial and dimethoxyethane (DME) was added. The reaction vessel was sealed, removed from the glovebox and heated at 70 °C for 16 h. The reaction mixture was cooled and filtered through a plug of silica, eluted with EtOAc and concentrated *in vacuo*. Purification by column chromatography was affected as the final purification.

### Coupling of aromatic B–C^(*sp*2)^ bond in diboryl species 8x

An oven-dried pressure tube with a stir bar in a glovebox was charged with diboronate **8x** (1 equiv.), Pd_2_(dba)_3_ (0.05 equiv.), [(*t*Bu)_3_PH]BF_4_ (0.2 equiv.), K_2_CO_3_ (3 equiv.), bromoarene (1.2 equiv.) and toluene (3.4 equiv.). The pressure tube was sealed with a rubber septum, removed from the glovebox and placed under argon. Water degassed (34:1 organic to water) was added and the rubber septum was replaced with a lid. The reaction was heated to 60 °C for 24 h. After cooling to room temperature, the reaction mixture was filtered through a plug of silica gel (ca. 2 mL) and a PROMAX 0.22 μm polytetrafluoroethylene (PTFE) syringe filter using copious ethyl acetate. The resulting product was purified by chromatography.

For NMR and super critical fluid chromatography (SFC) analysis of the compounds in this article, see Supplementary Figs 1–99.

## Additional information

**How to cite this article:** Crudden, C. M. *et al*. Iterative protecting group-free cross-coupling leading to chiral multiply arylated structures. *Nat. Commun.* 7:11065 doi: 10.1038/ncomms11065 (2016).

## Supplementary Material

Supplementary Information Supplementary Figures 1-99, Supplementary Methods and Supplementary References

## Figures and Tables

**Figure 1:**
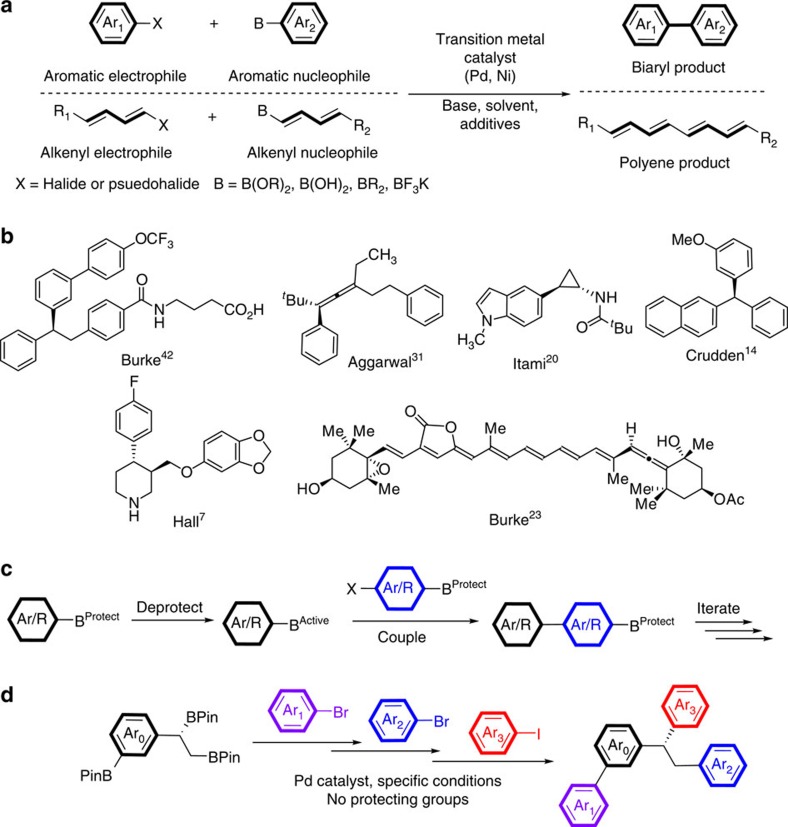
Advances in the Suzuki–Miyaura cross-coupling reaction. (**a**) Classical Suzuki–Miyaura reaction for the formation of C–C bonds between aryl or alkenyl electrophiles and aryl or alkenyl organoboron nucleophiles. (**b**) Selected examples of molecules prepared by enantiospecific Suzuki–Miyaura cross-coupling reactions. (**c**) Iterative coupling concept employing the Suzuki–Miyaura reaction as the key structure-building component. (**d**) This work: chemoselective, protecting group-free cross-coupling of multiply borylated organic compounds, including the coupling of chiral, enantioenriched B–C bonds.
