# Supplementary material for: Iterative protecting group-free cross-coupling leading to chiral multiply arylated structures
Source: Nat Commun. 2016 Apr 4;7:11065. doi: 10.1038/ncomms11065 (PMC4822017; doi:10.1038/ncomms11065)
Supplement: Supplementary Information — Supplementary Figures 1-99, Supplementary Methods and Supplementary References [file ncomms11065-s1.pdf]

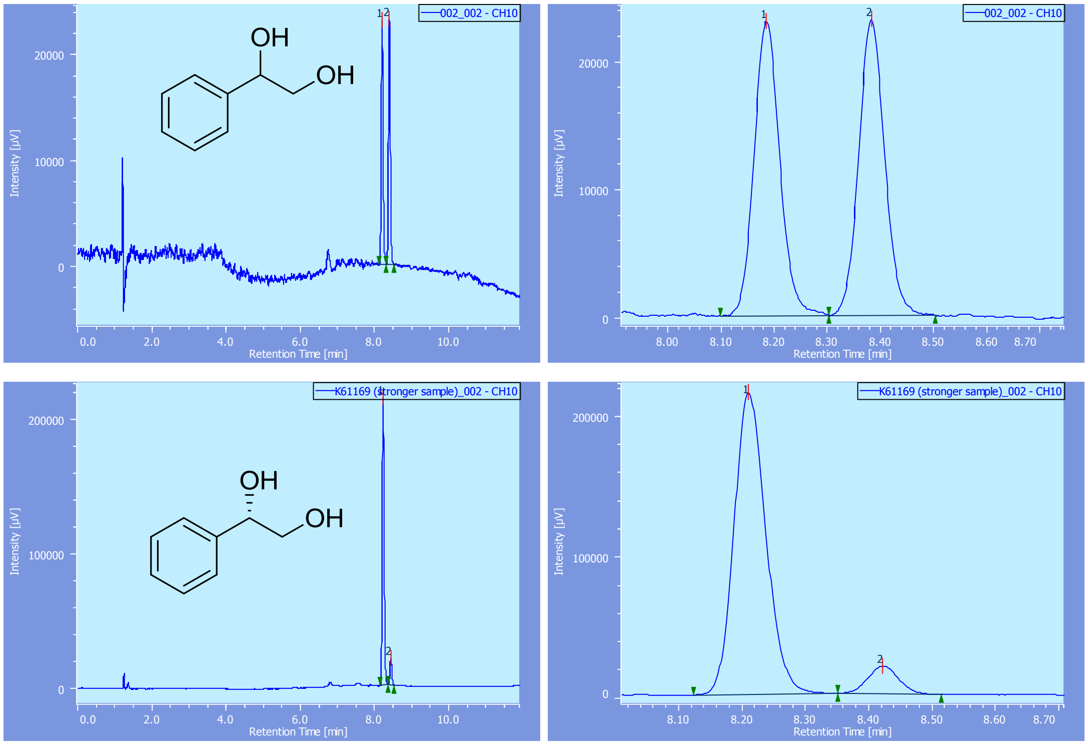

Supplementary Figure 1 | SFC traces of oxidized 5a synthesized under Pt catalysis.

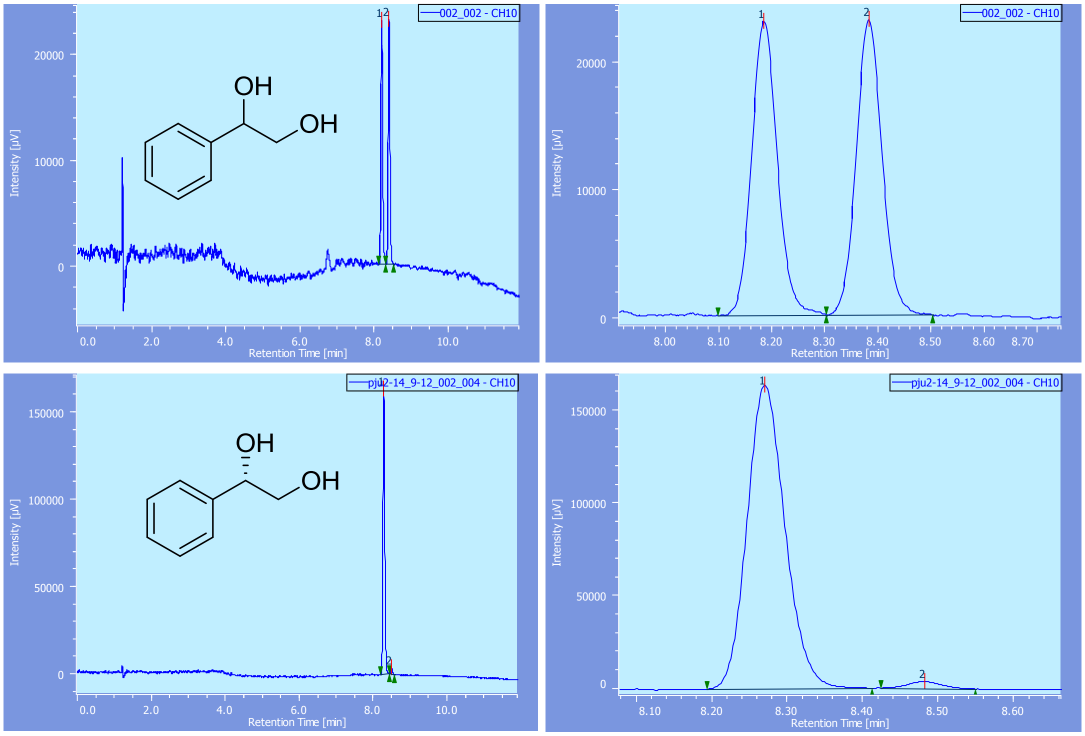

Supplementary Figure 2 | SFC traces of oxidized 5a synthesized under 1 mol% Rh catalysis.

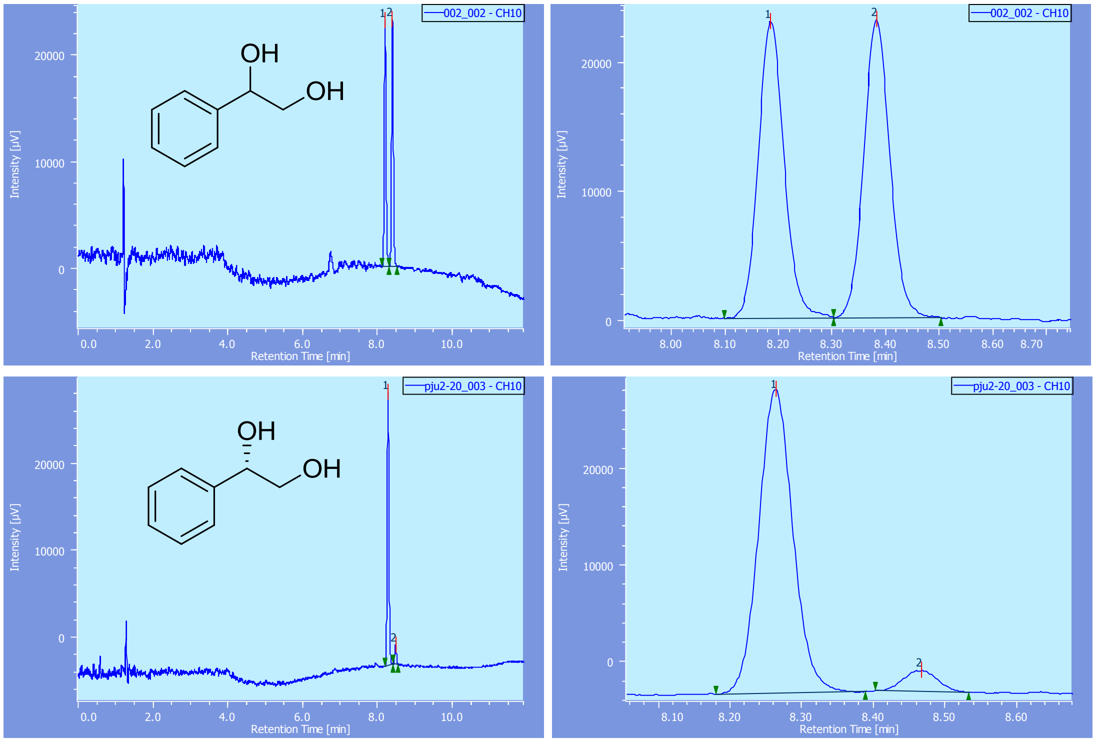

Supplementary Figure 3│SFC traces of oxidized 5a synthesized under 0.2 mol% Rh catalysis.

a)

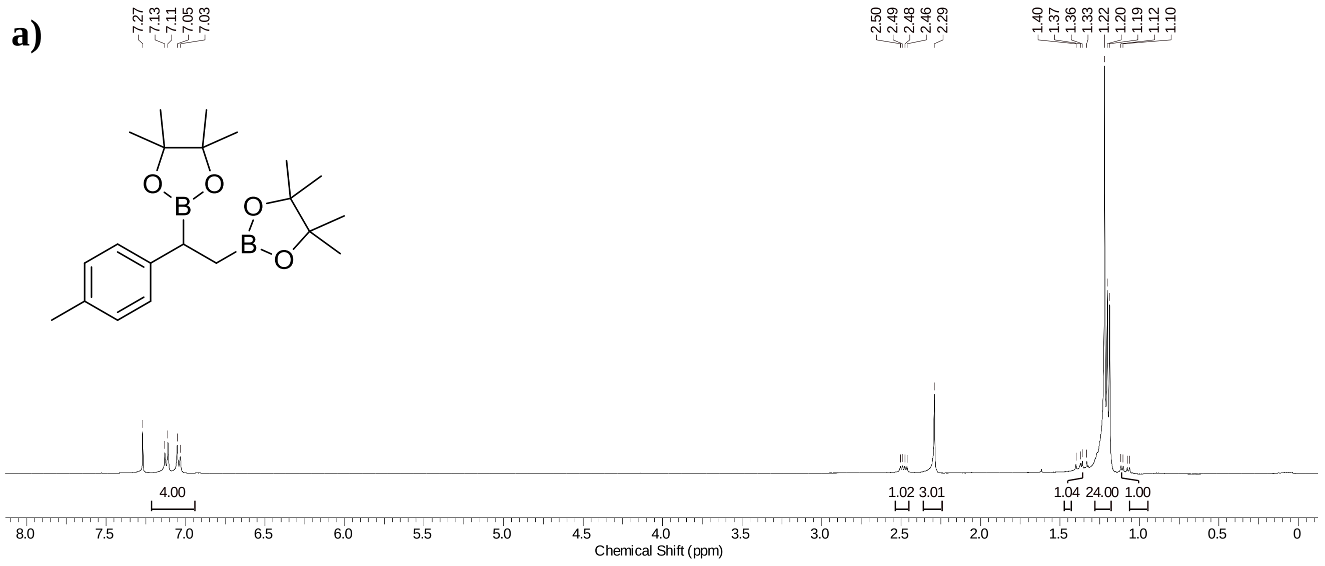

b)

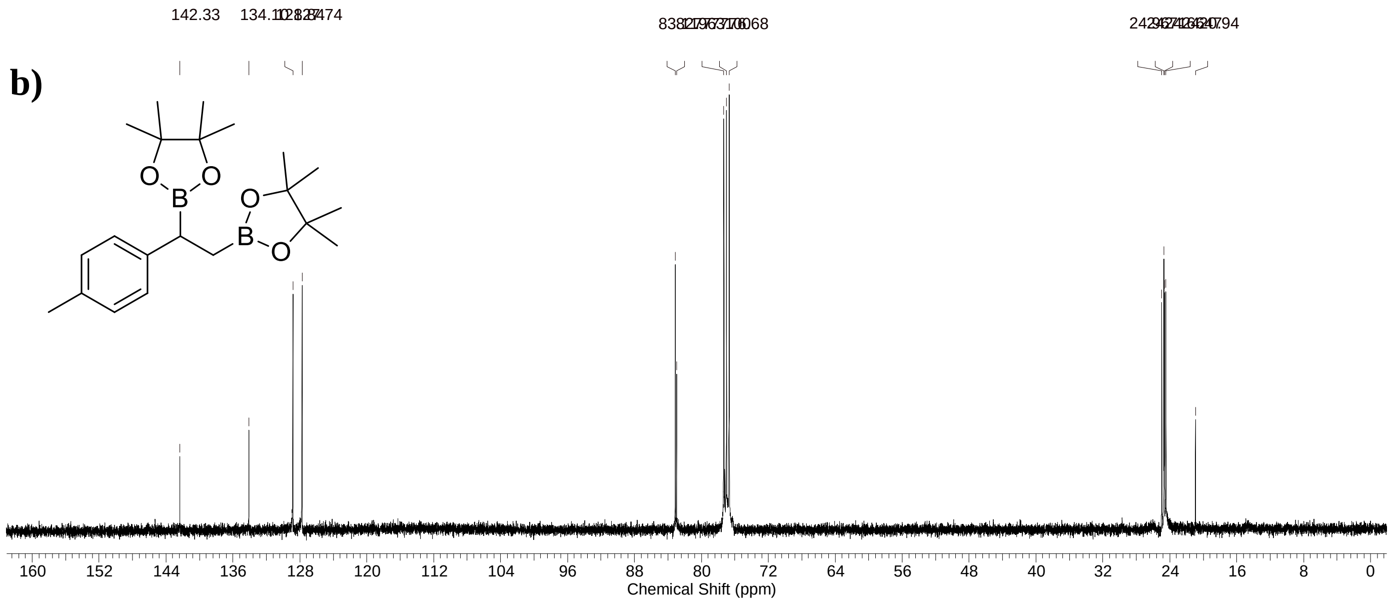

Supplementary Figure 4 │NMR spectra of 5b. a) ^{1}H NMR spectrum. b) ^{13}C NMR spectrum.

a)

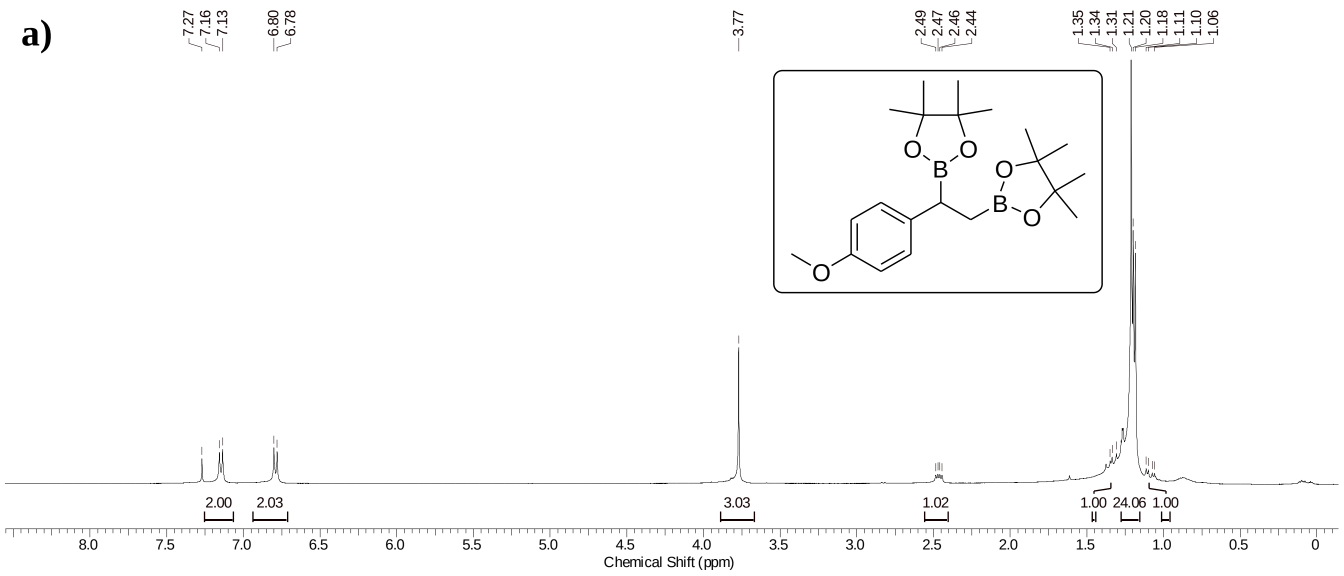

b)

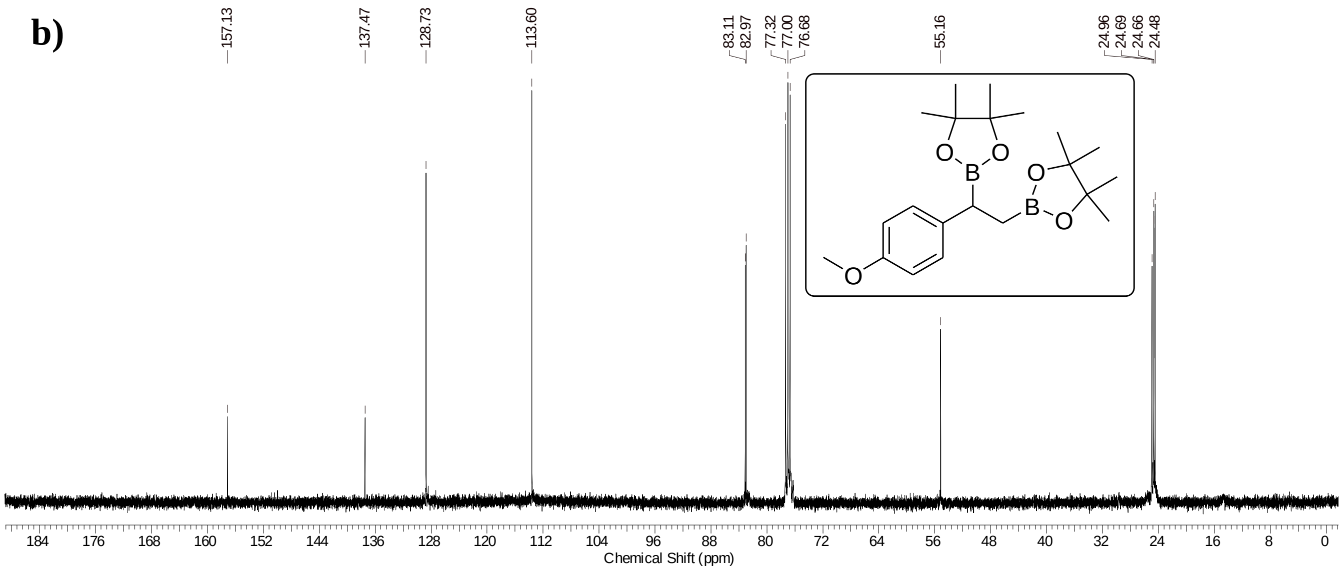

Supplementary Figure 5 │ NMR spectra of 5f. a) ^{1}H NMR spectrum. b) ^{13}C NMR spectrum.

a)

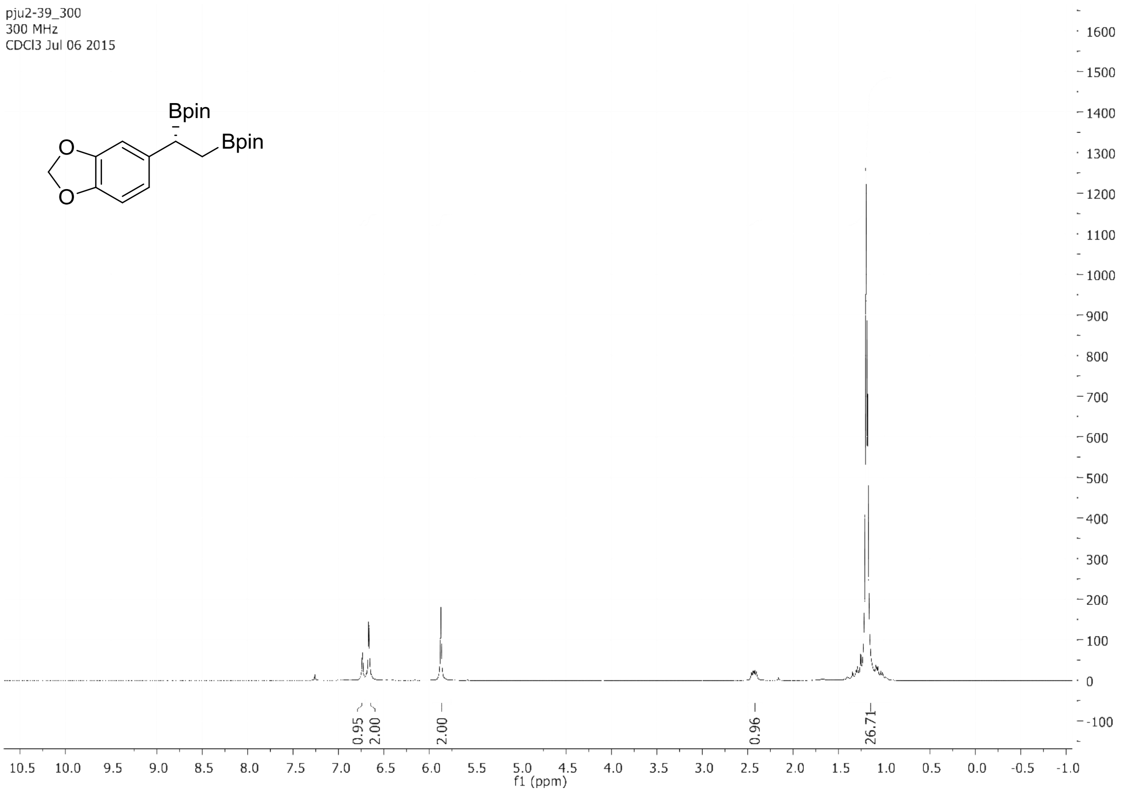

b)

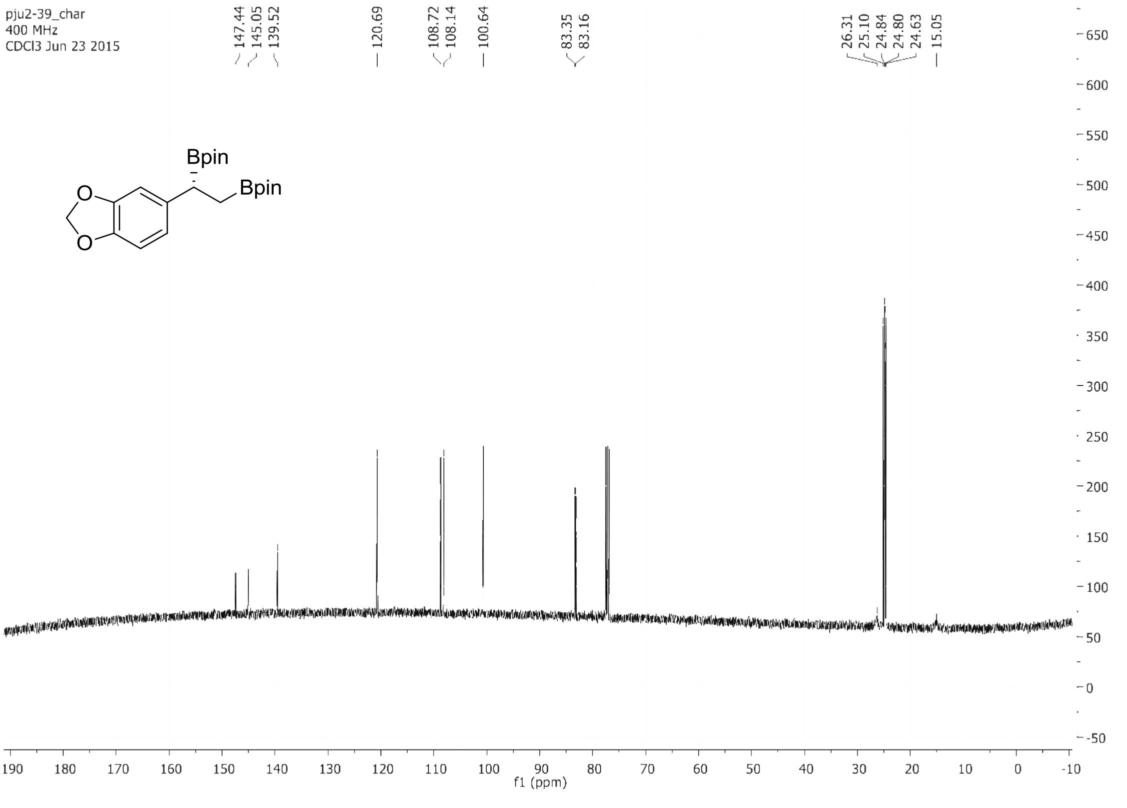

Supplementary Figure 6 | NMR spectra of 5g. a) ^{1}H NMR spectrum. b) ^{13}C NMR spectrum.

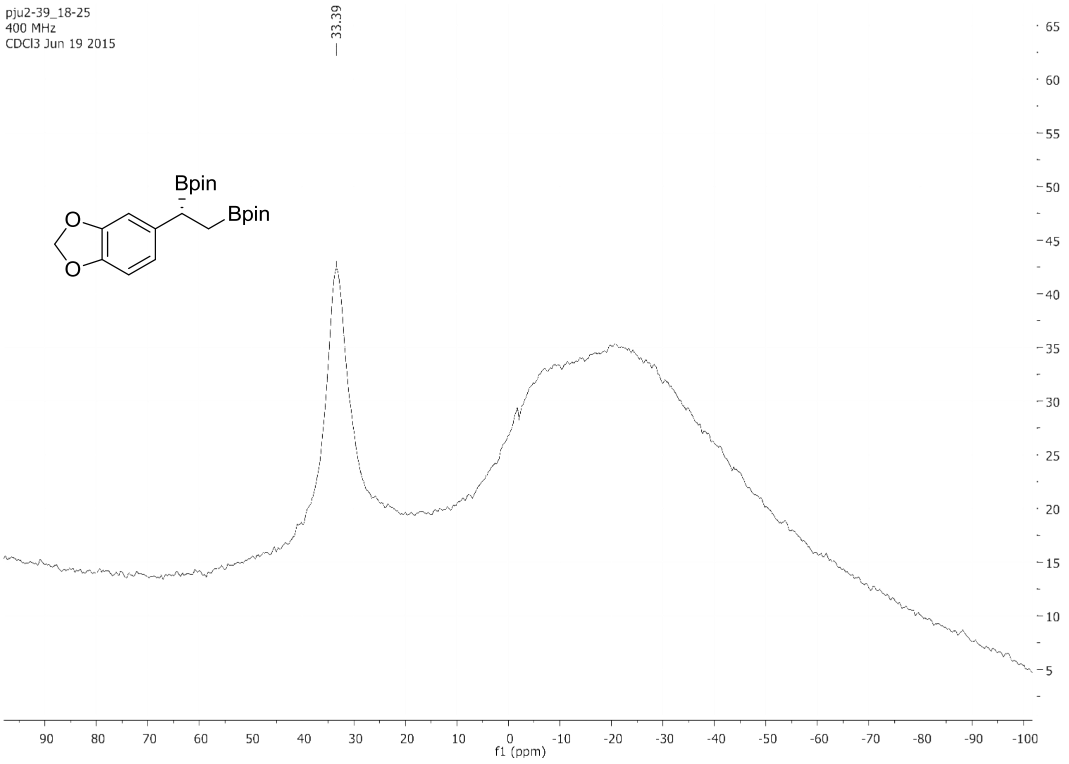

Supplementary Figure 7 | ^{11}B NMR spectrum of 5g.

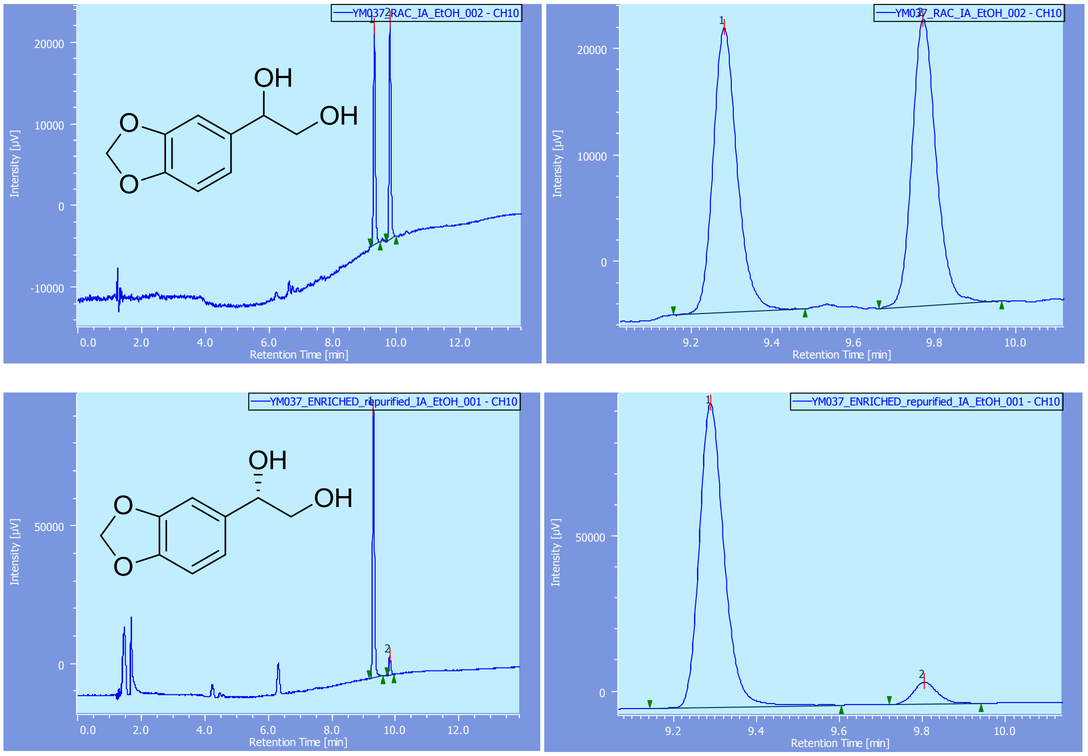

Supplementary Figure 8 | SFC traces of oxidized 5g.

a)

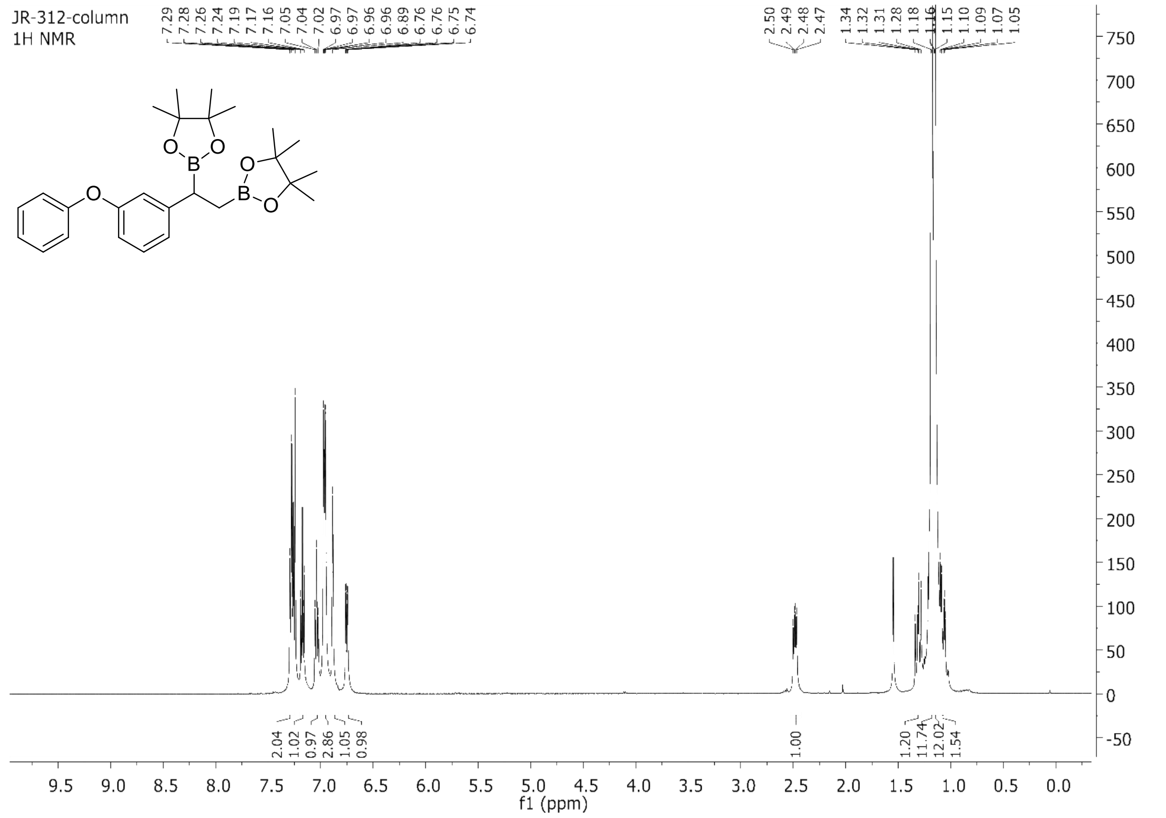

b)

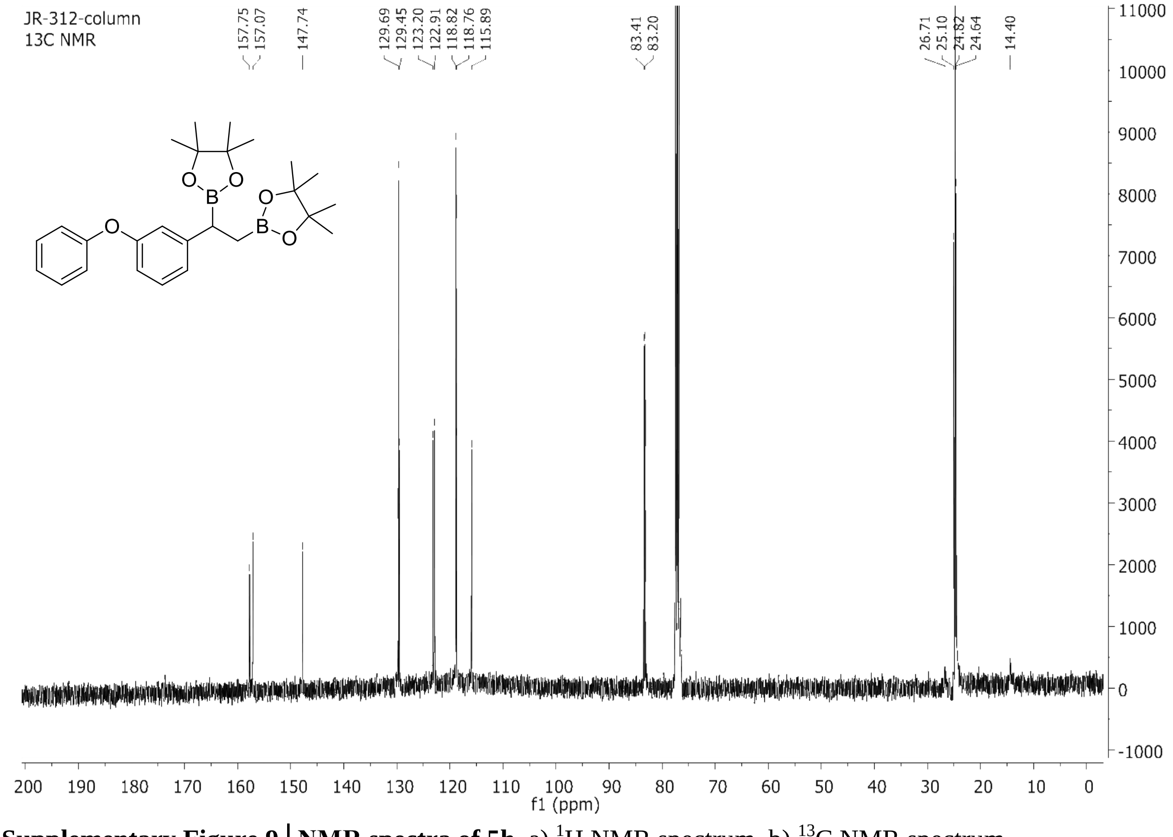

Supplementary Figure 9 | NMR spectra of 5h. a) ^{1}H NMR spectrum. b) ^{13}C NMR spectrum.

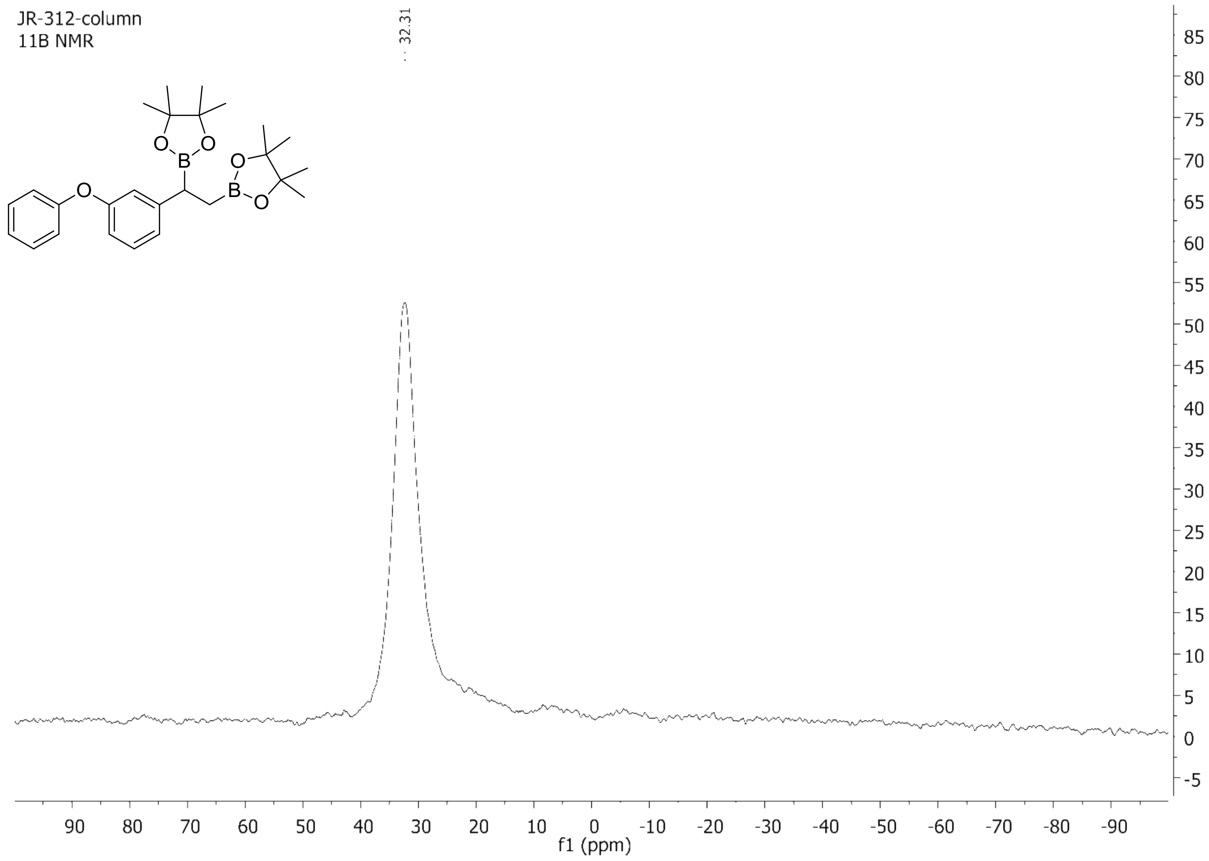

Supplementary Figure 10 | ^{11}B NMR spectrum of 5h.

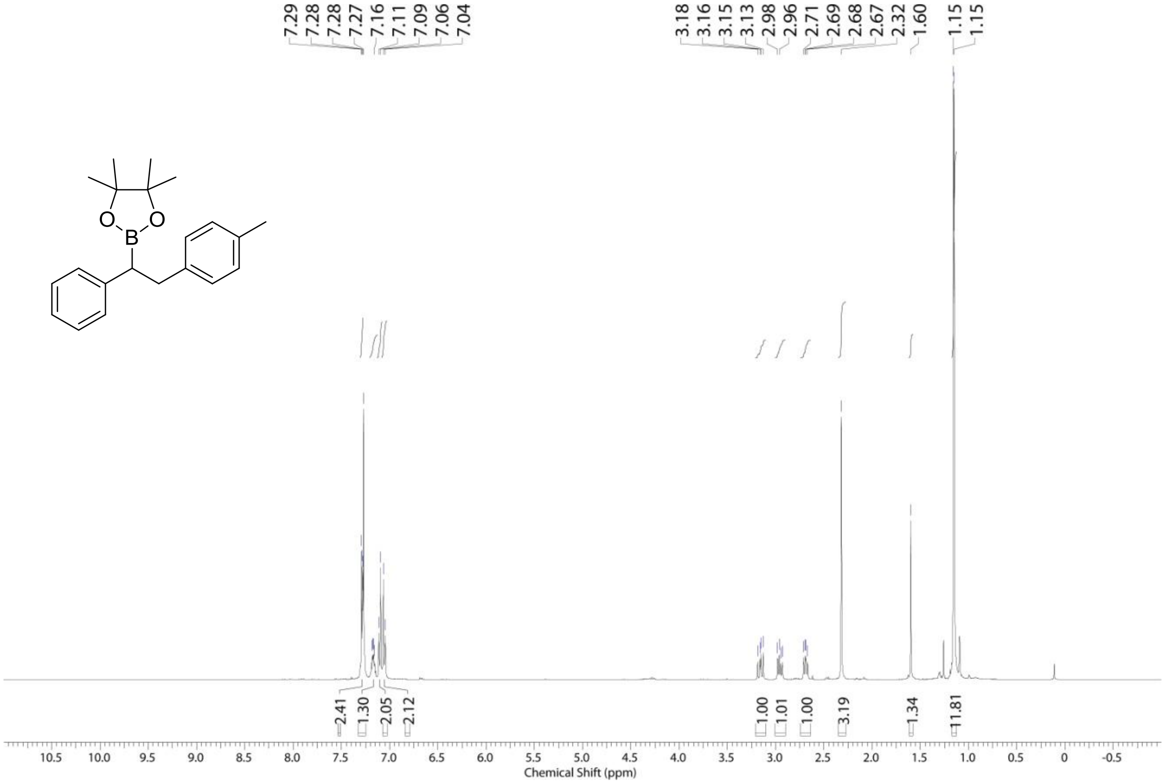

Supplementary Figure 11 | ^{1}H NMR spectrum of 6aB.

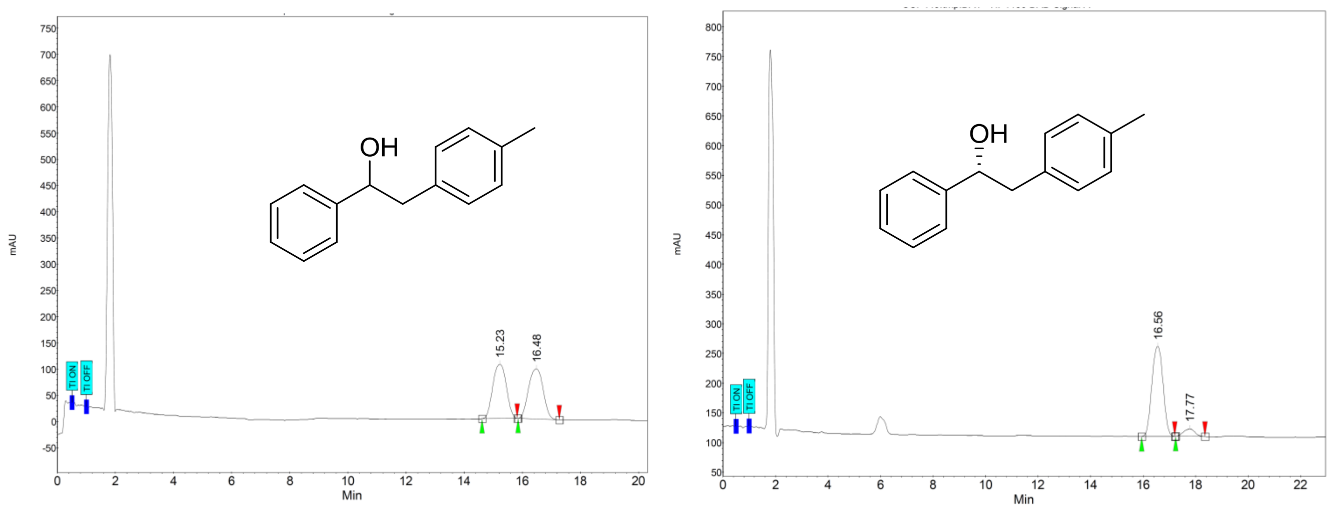

Supplementary Figure 12 | SFC traces of oxidized 6aB.

a)

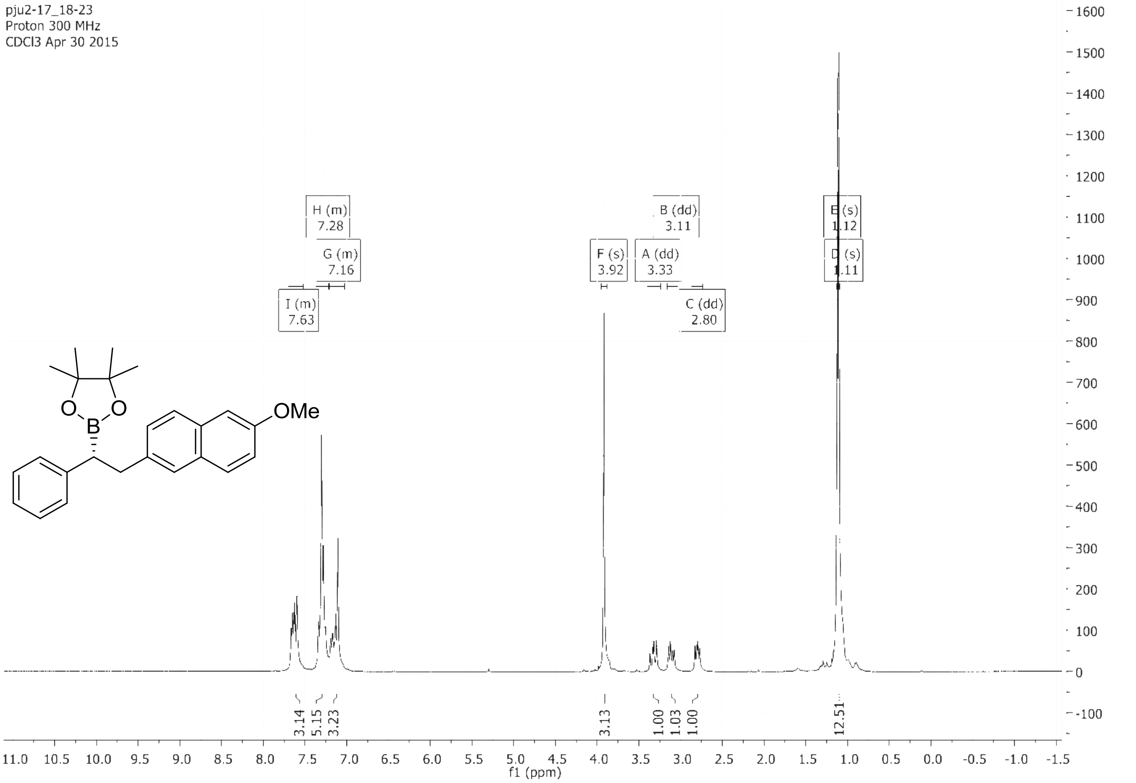

b)

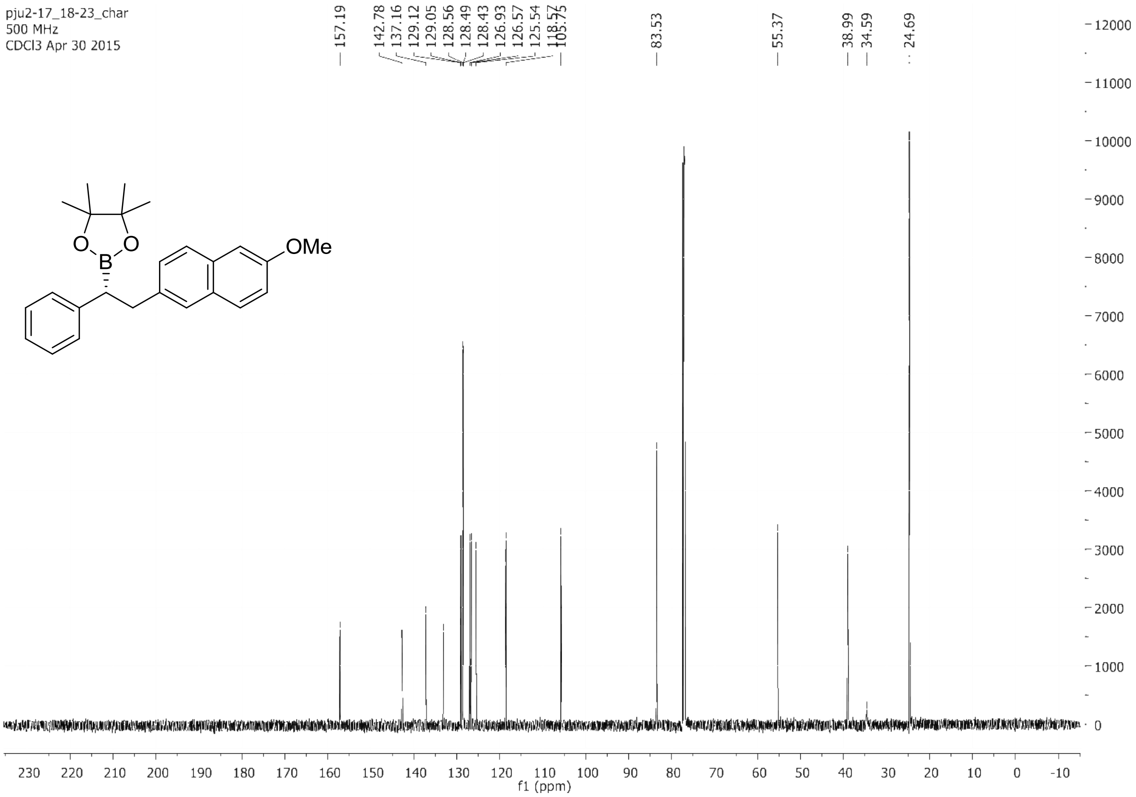

Supplementary Figure 13 | NMR spectra of 6aC. a) ^{1}H NMR spectrum. b) ^{13}C NMR spectrum.

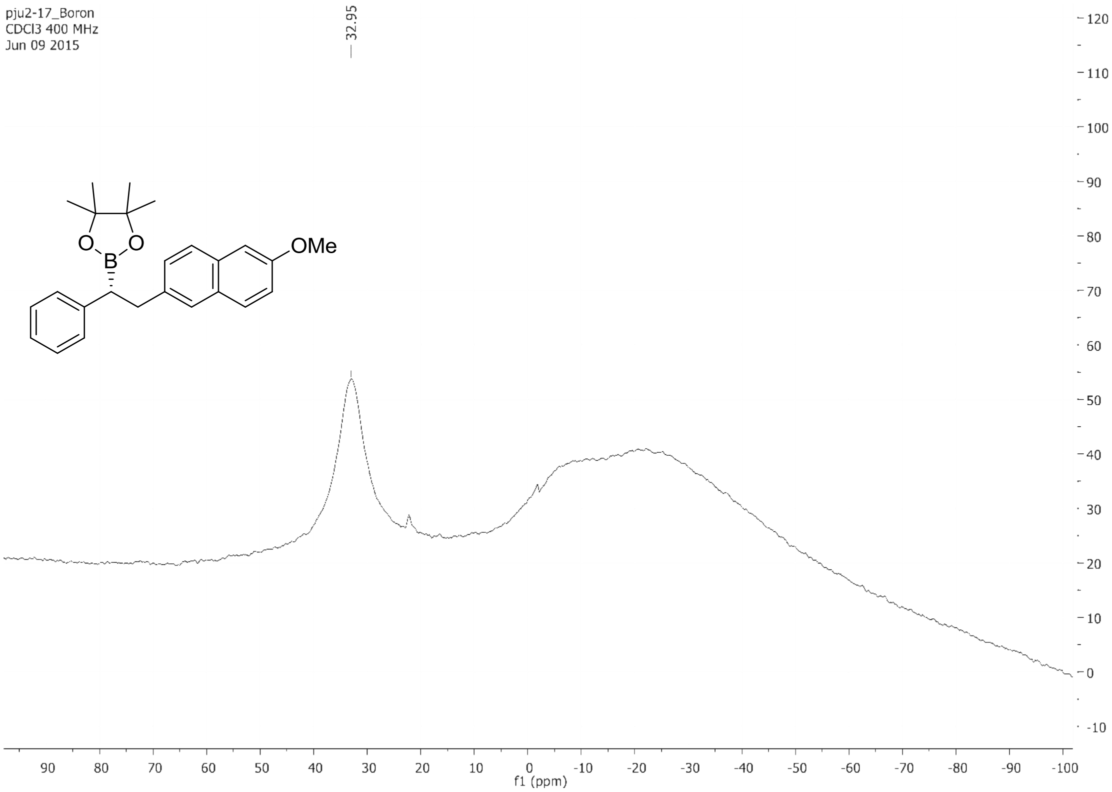

Supplementary Figure 14 | ^{11}B NMR spectrum of 6aC.

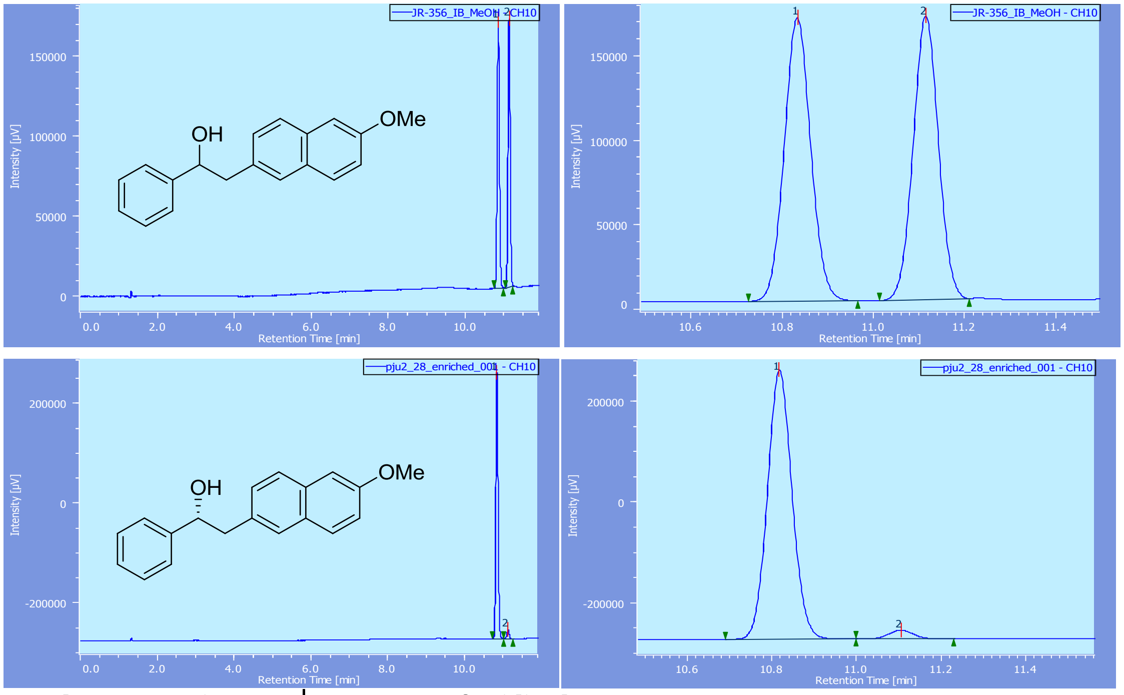

Supplementary Figure 15 | SFC traces of oxidized 6aC.

a)

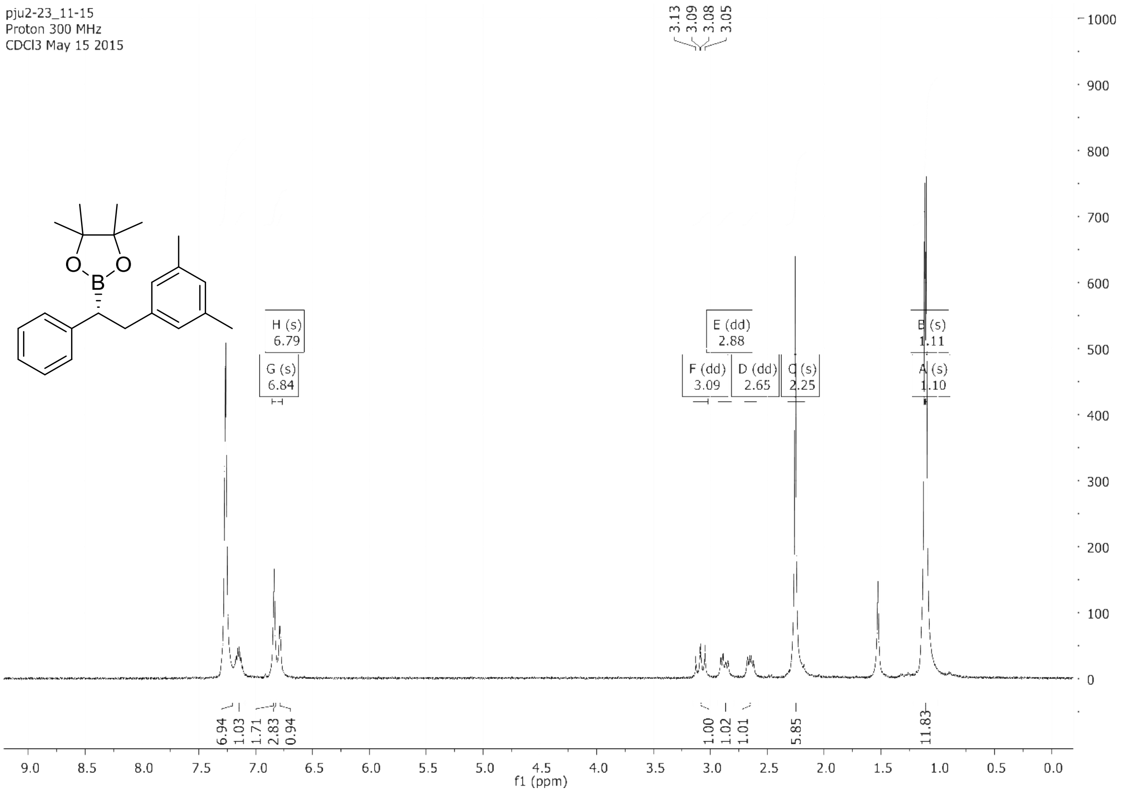

b)

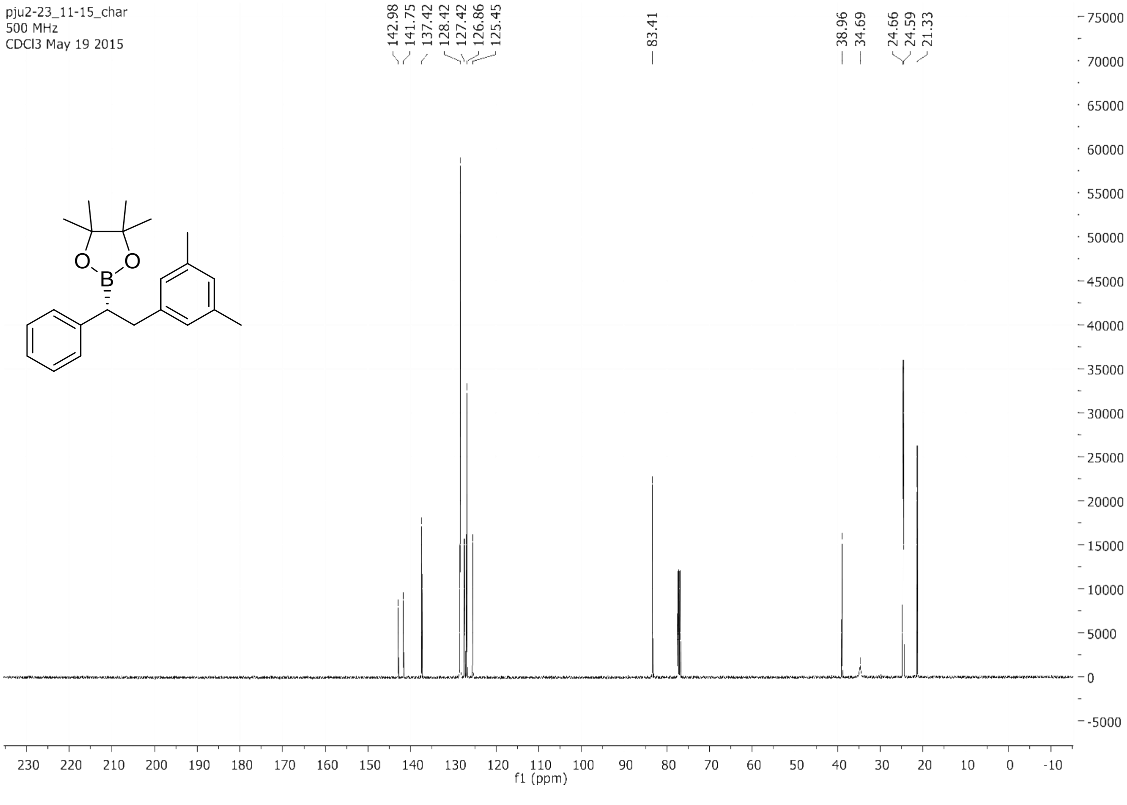

Supplementary Figure 16 | NMR spectra of 6aD. a) ^{1}H NMR spectrum. b) ^{13}C NMR spectrum.

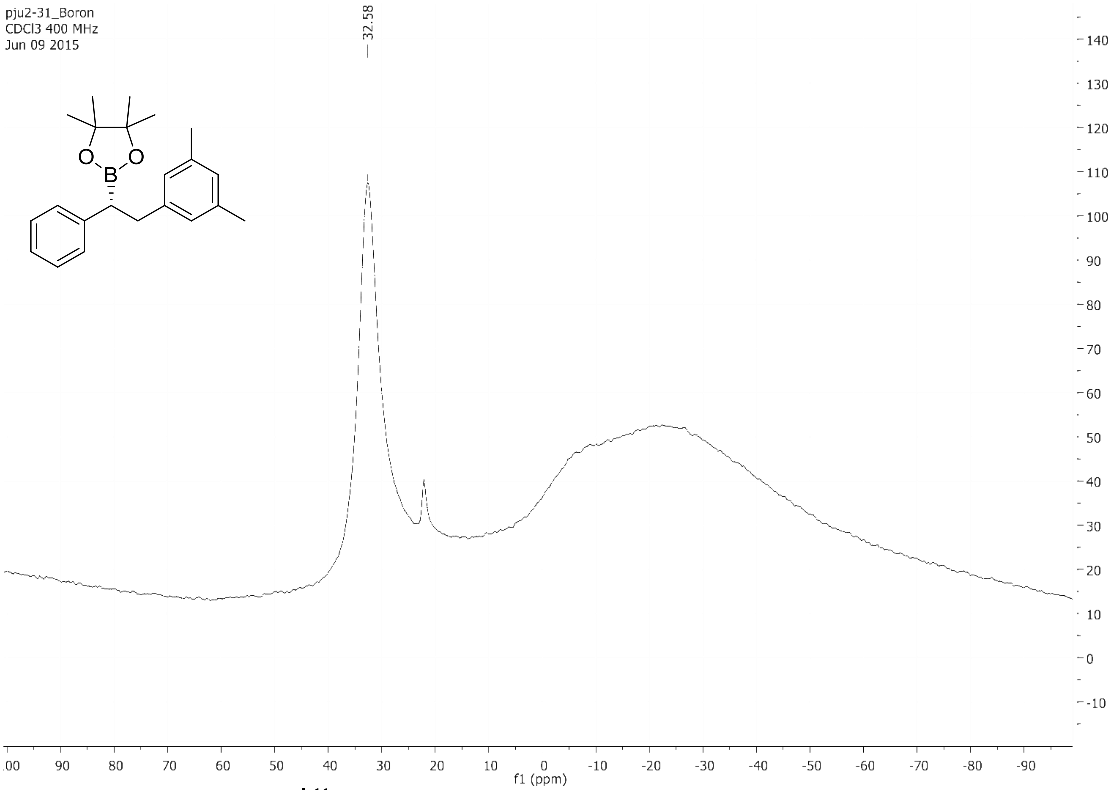

Supplementary Figure 17 | ^{11}B NMR spectrum of 6aD.

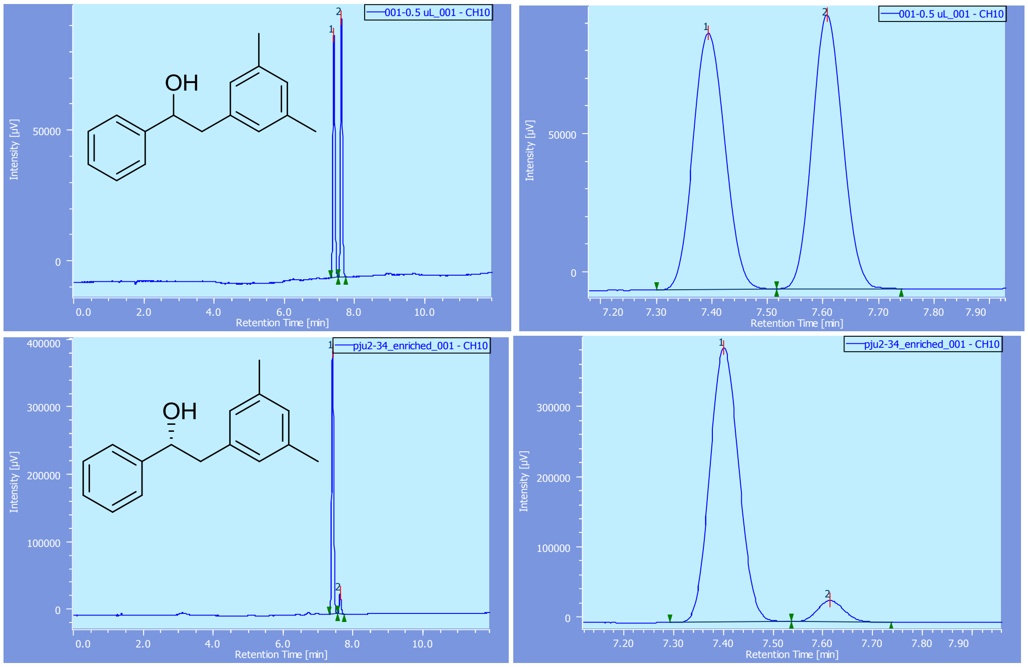

Supplementary Figure 18 | SFC traces of oxidized 6aD.

a)

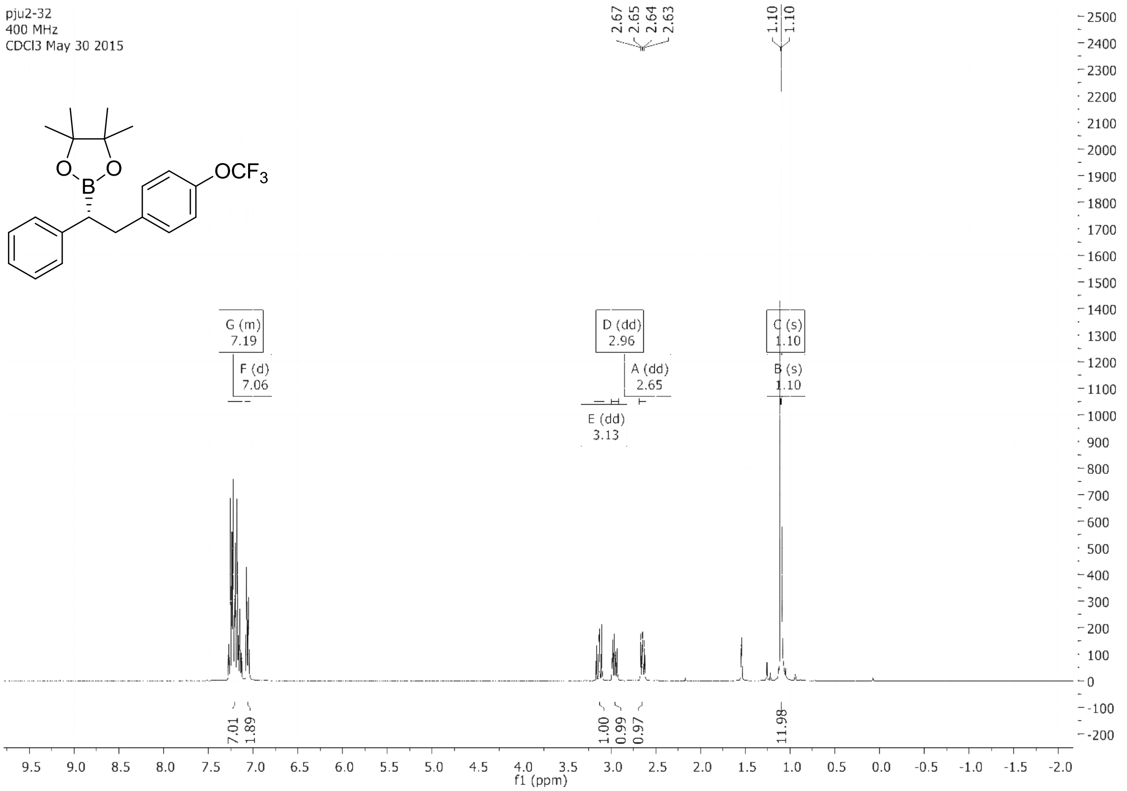

b)

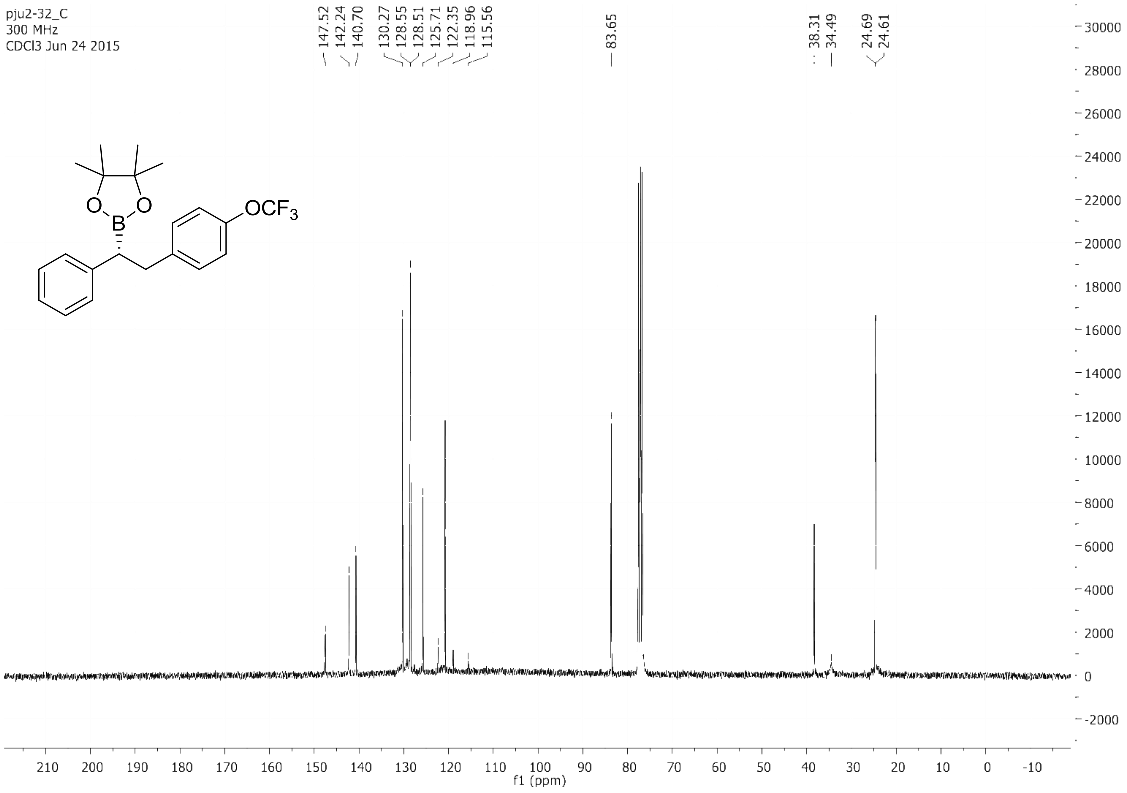

Supplementary Figure 19 | NMR spectra of 6aE. a) ^{1}H NMR spectrum. b) ^{13}C NMR spectrum.

a)

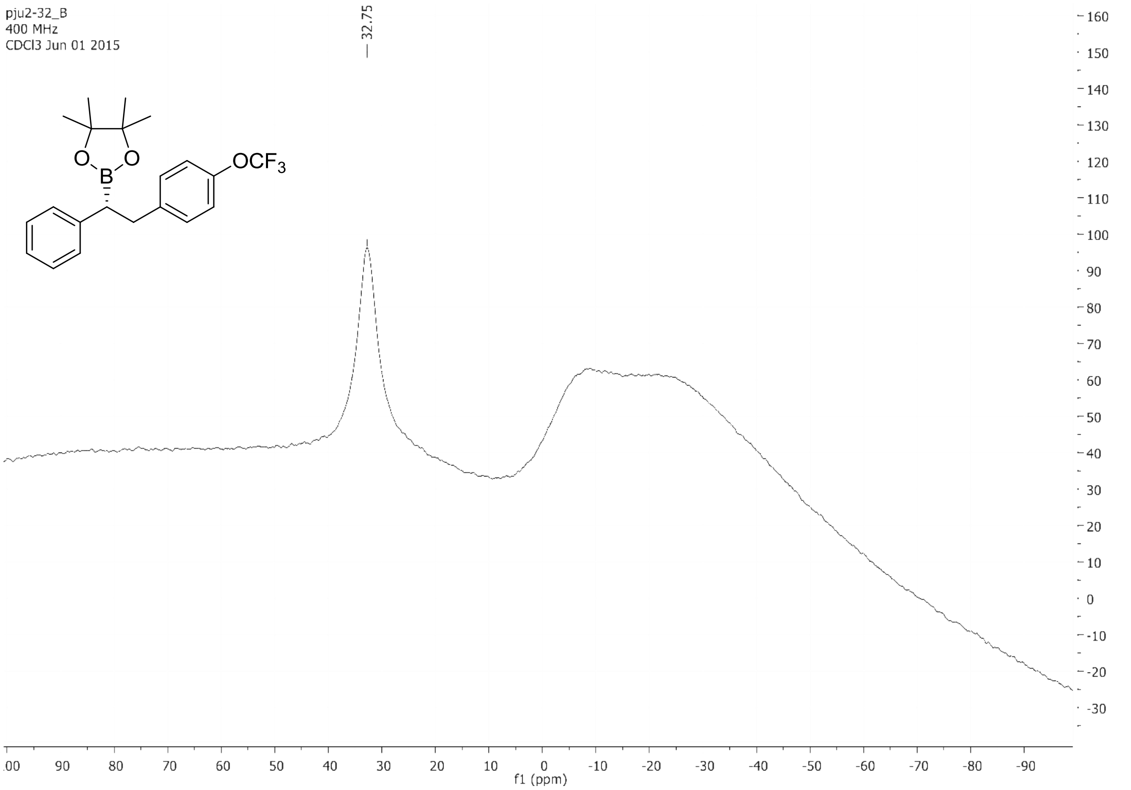

b)

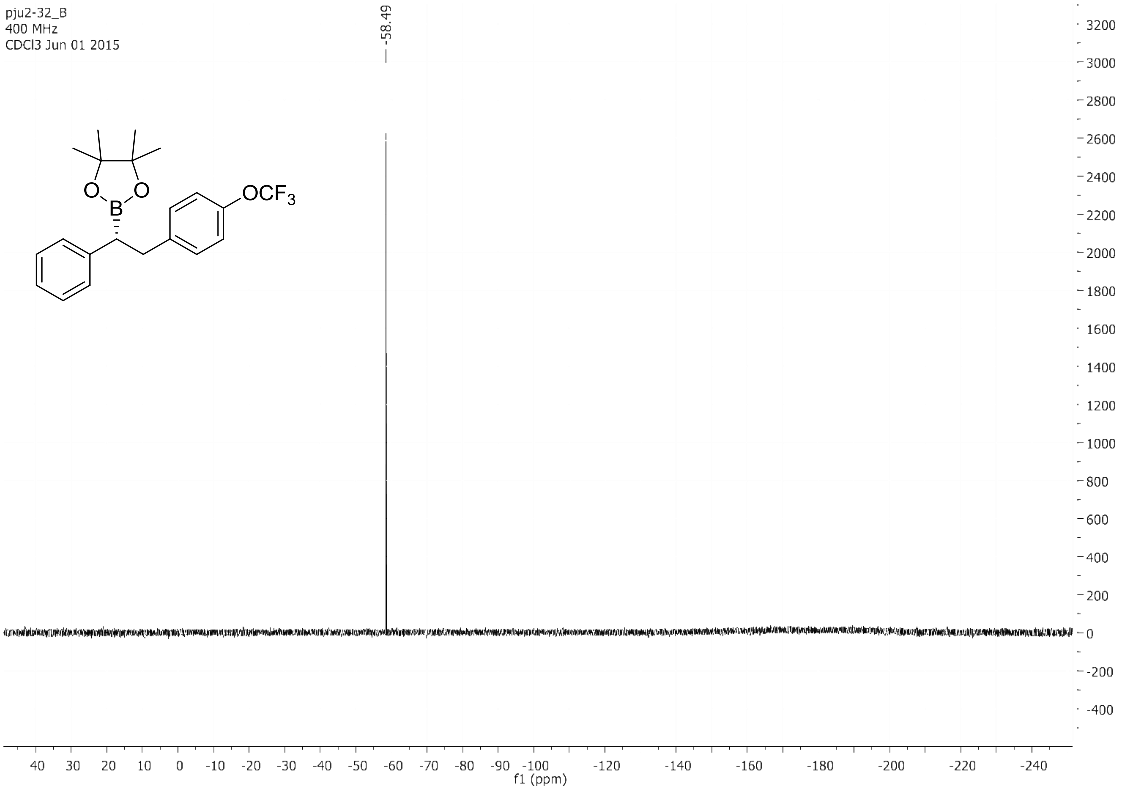

Supplementary Figure 20 | NMR spectra of 6aE. a) ^{11}B NMR spectrum. b) ^{19}F NMR spectrum.

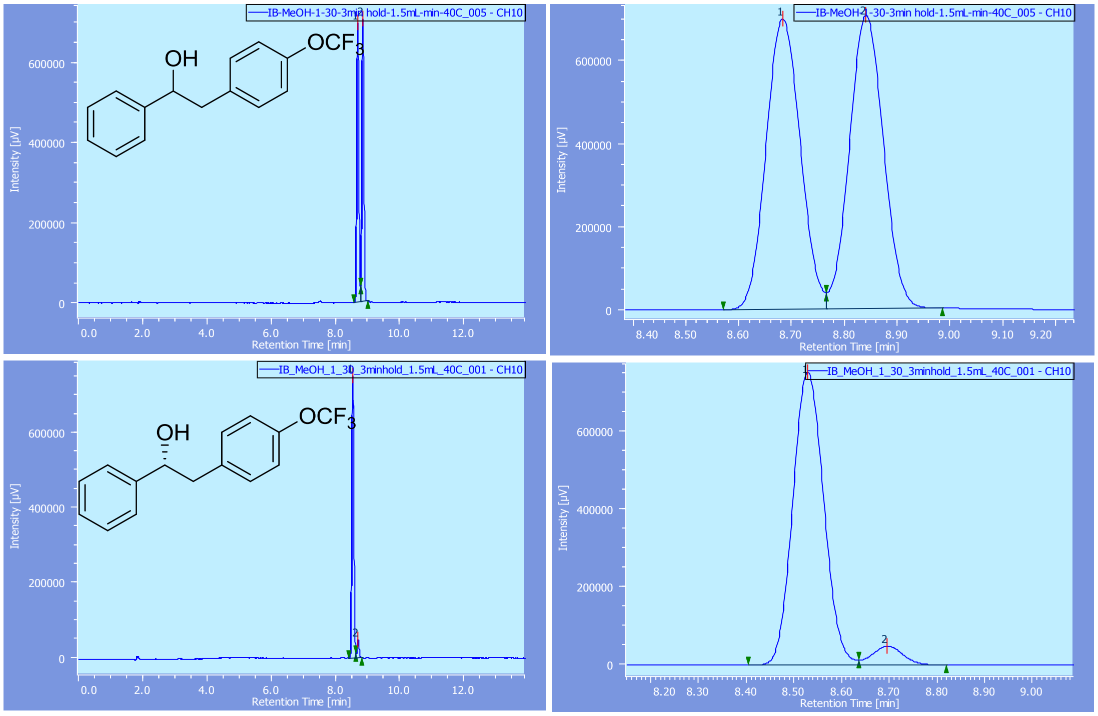

Supplementary Figure 21 | SFC spectra of oxidized 6aE.

a)

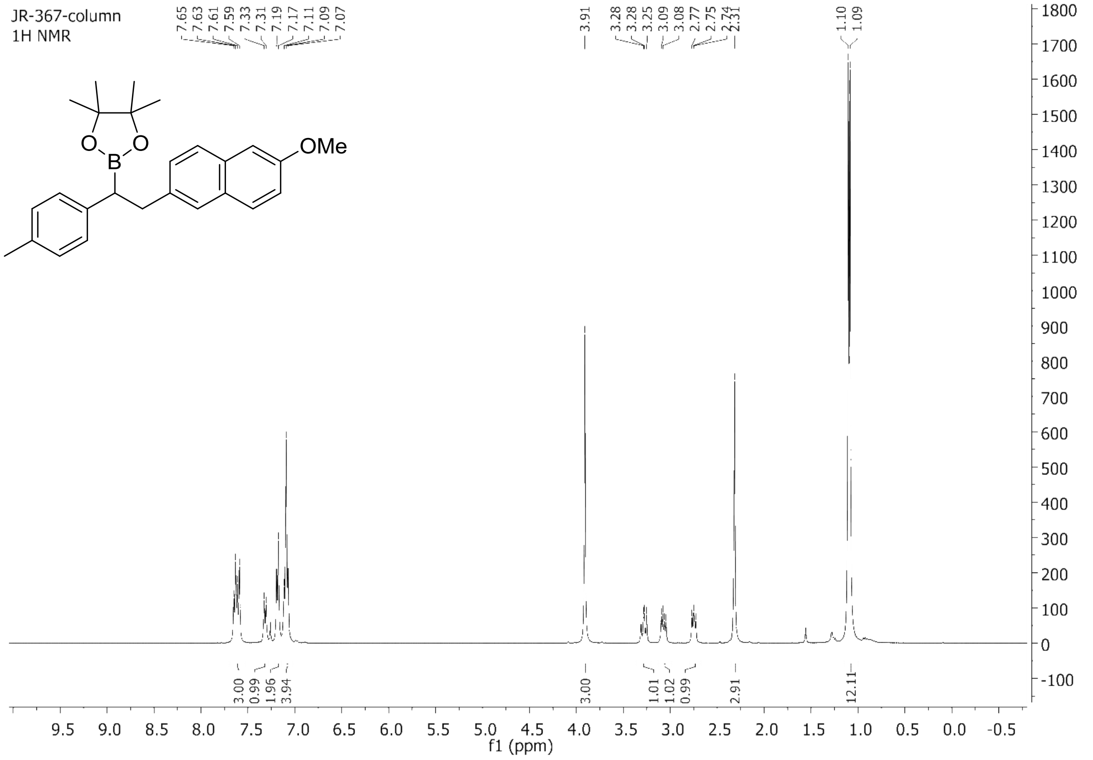

b)

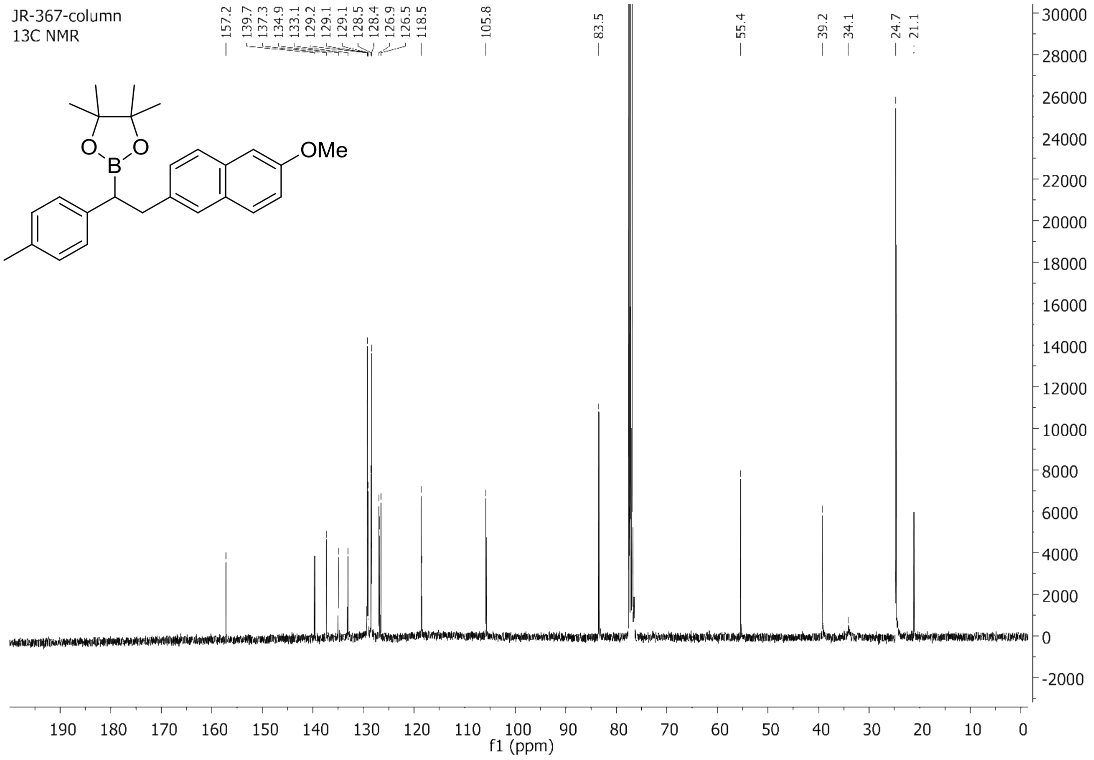

Supplementary Figure 22 | NMR spectra of 6bC. a) ^{1}H NMR spectrum. b) ^{13}C NMR spectrum.

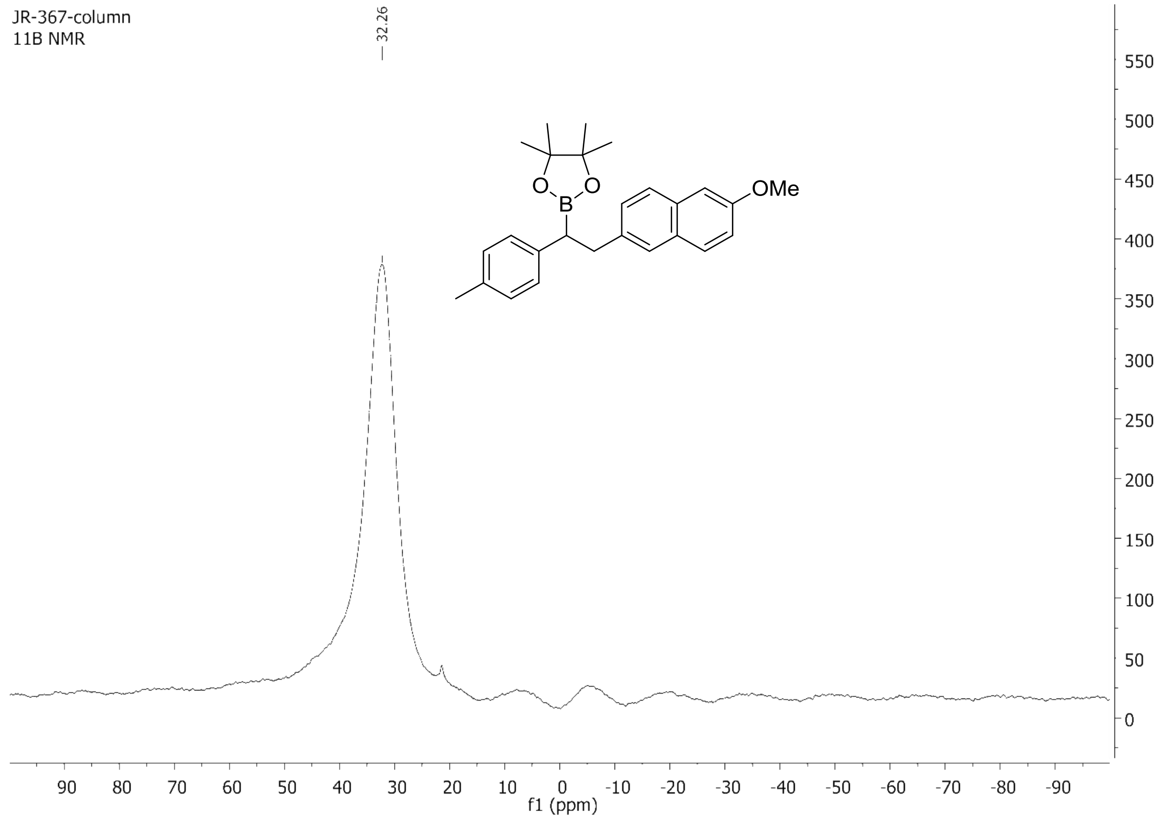

Supplementary Figure 23 | ^{11}B NMR spectrum of 6bC.

a)

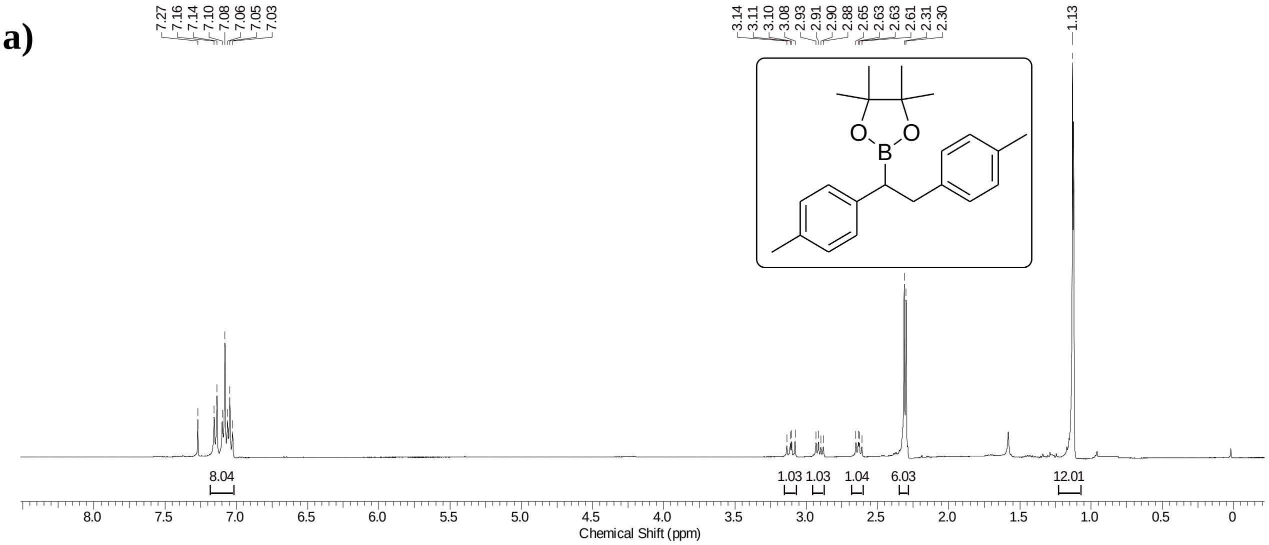

b)

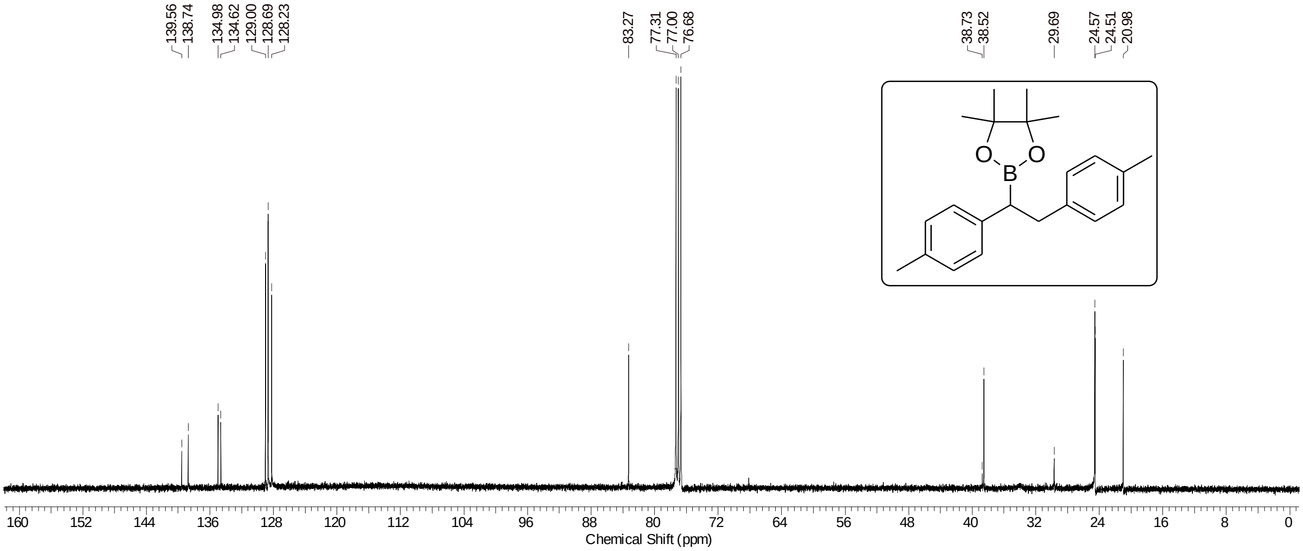

Supplementary Figure 24 | NMR spectra of 6bB. a) ^{1}H NMR spectrum. b) ^{13}C NMR spectrum.

a)

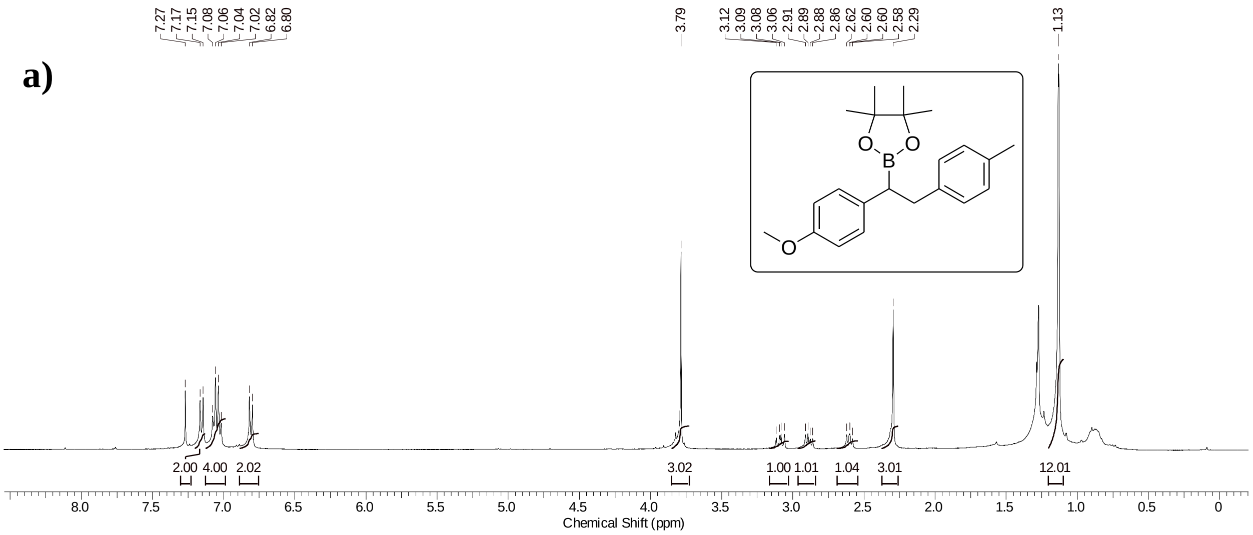

b)

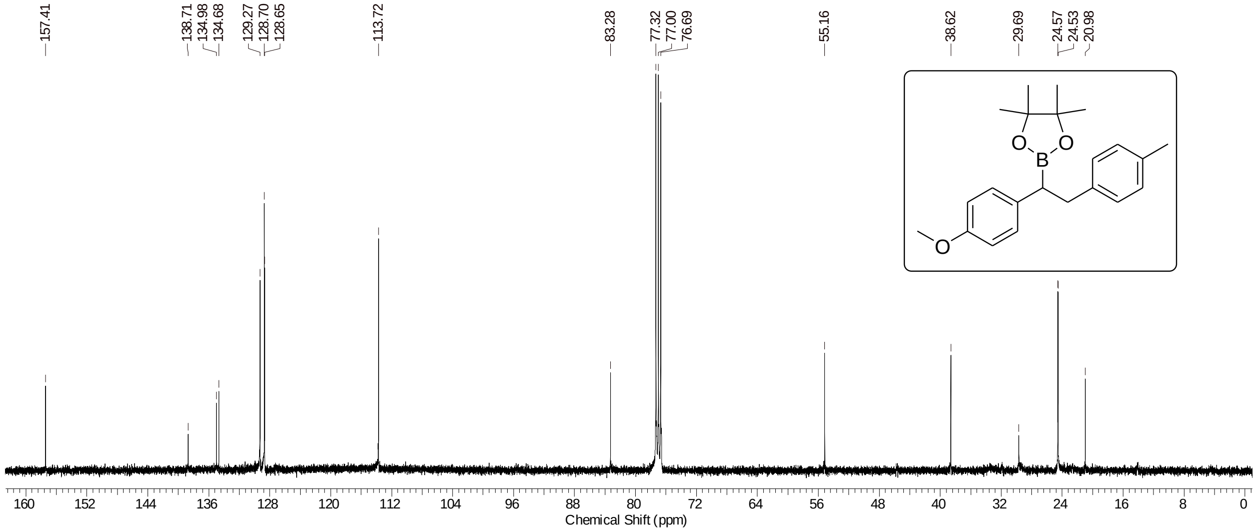

Supplementary Figure 25 | NMR spectra of 6fB. a) ^{1}H NMR spectrum. b) ^{13}C NMR spectrum.

a)

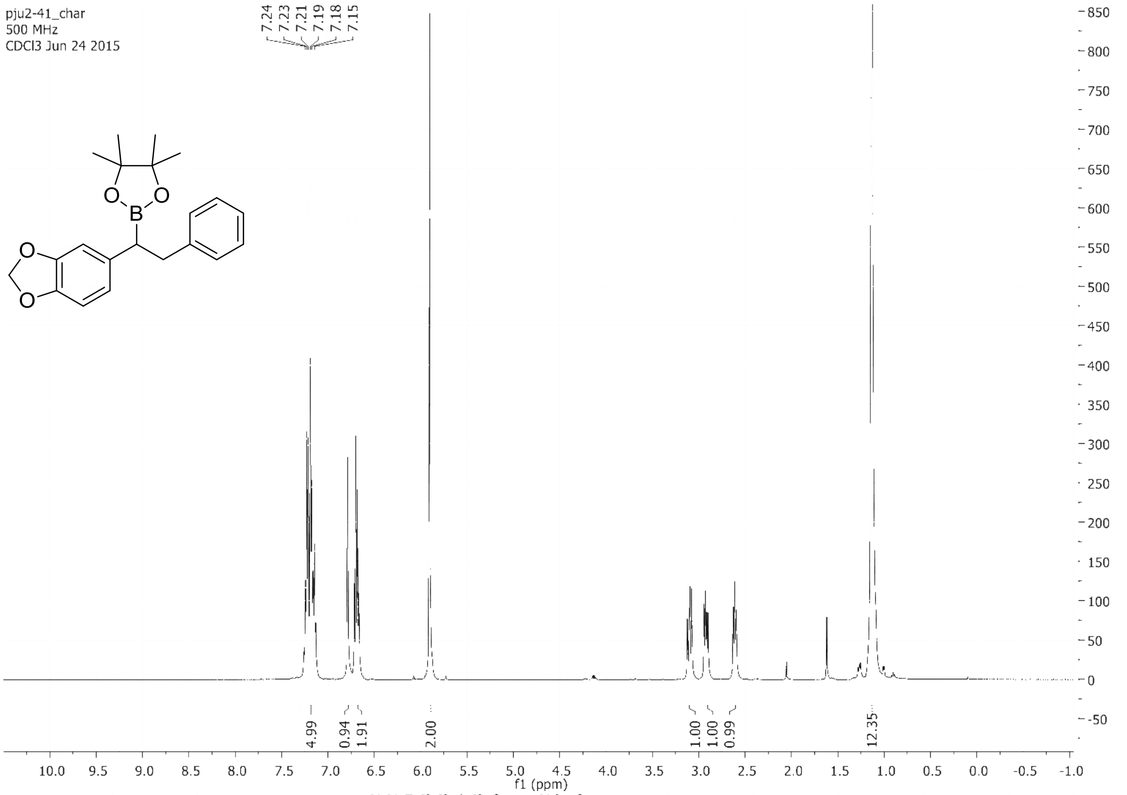

b)

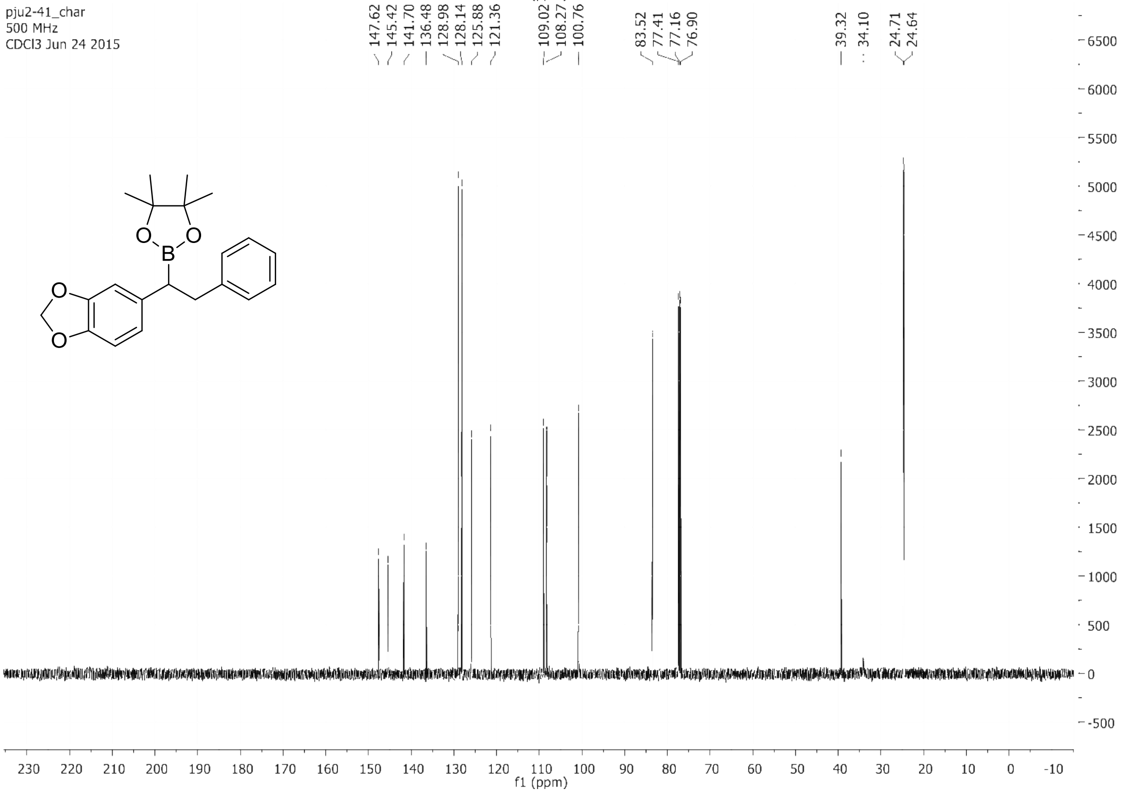

Supplementary Figure 26 | NMR spectra of 6gA. a) ^{1}H NMR spectrum. b) ^{13}C NMR spectrum.

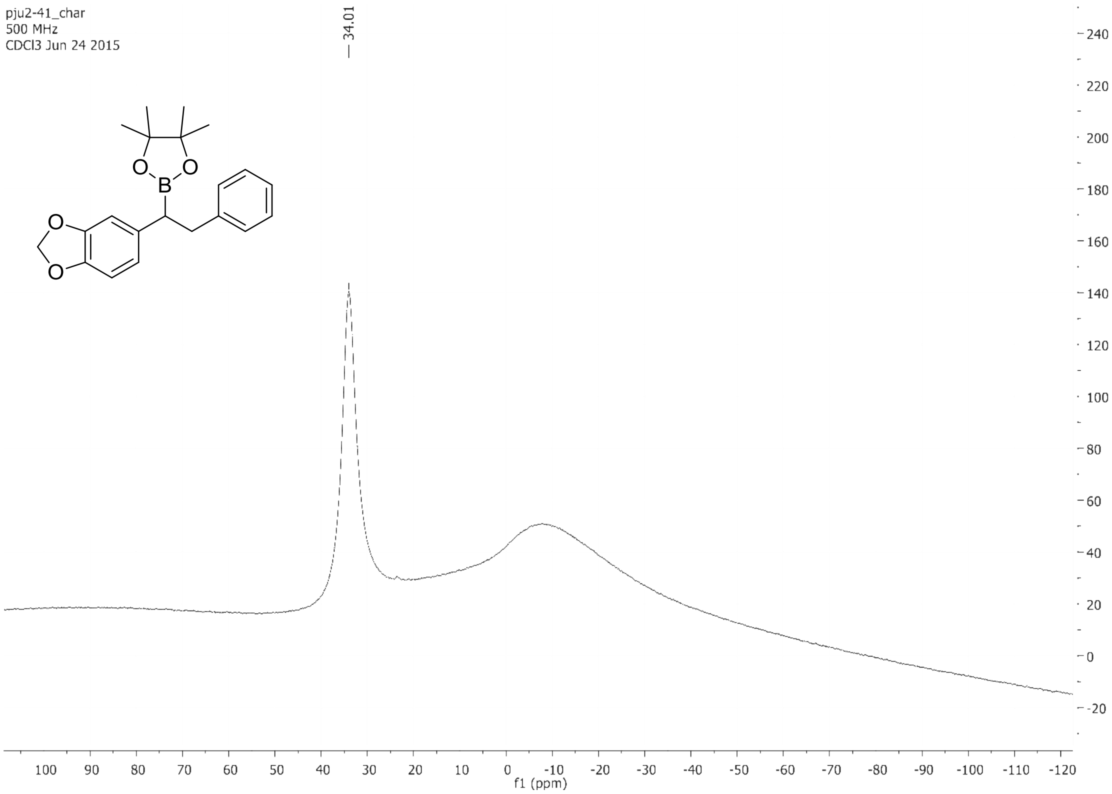

Supplementary Figure 27 | ^{11}B NMR spectrum of 6gA.

a)

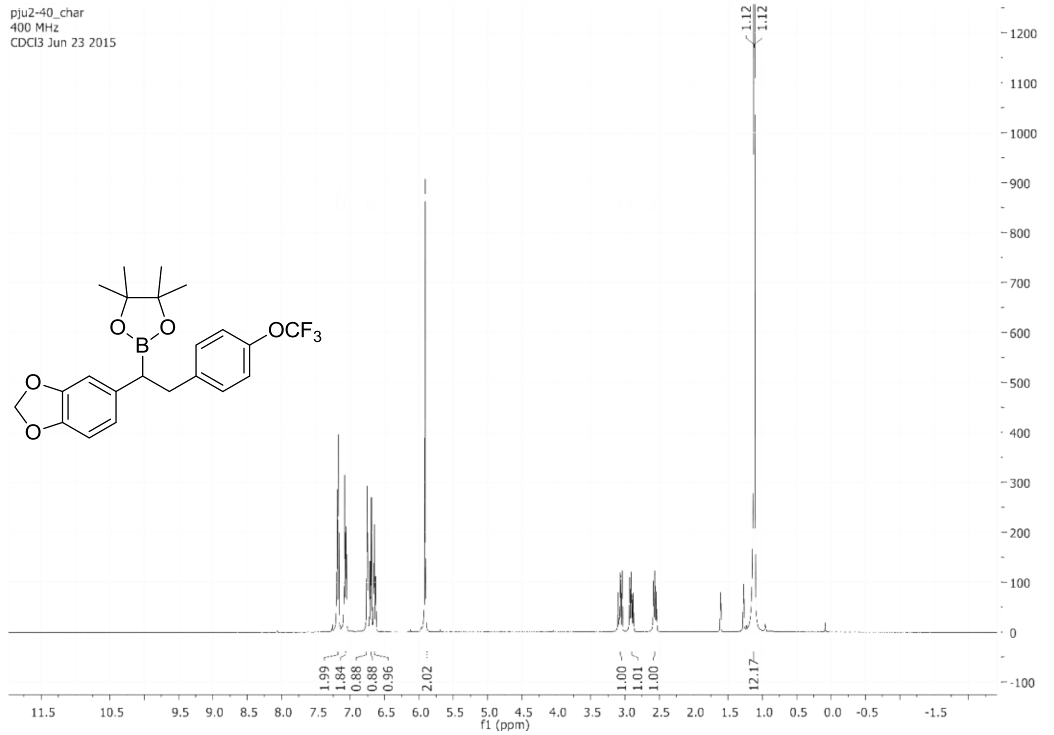

b)

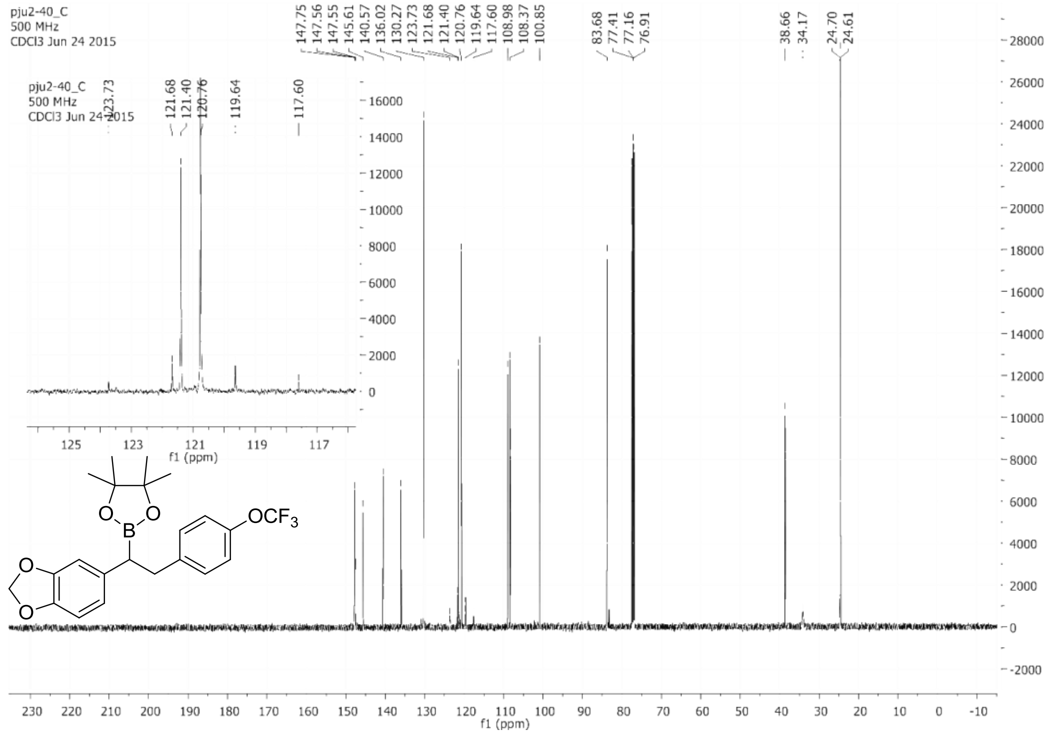

Supplementary Figure 28 │ NMR spectra of 6gE. a) ^{1}H NMR spectrum. b) ^{13}C NMR spectrum.

a)

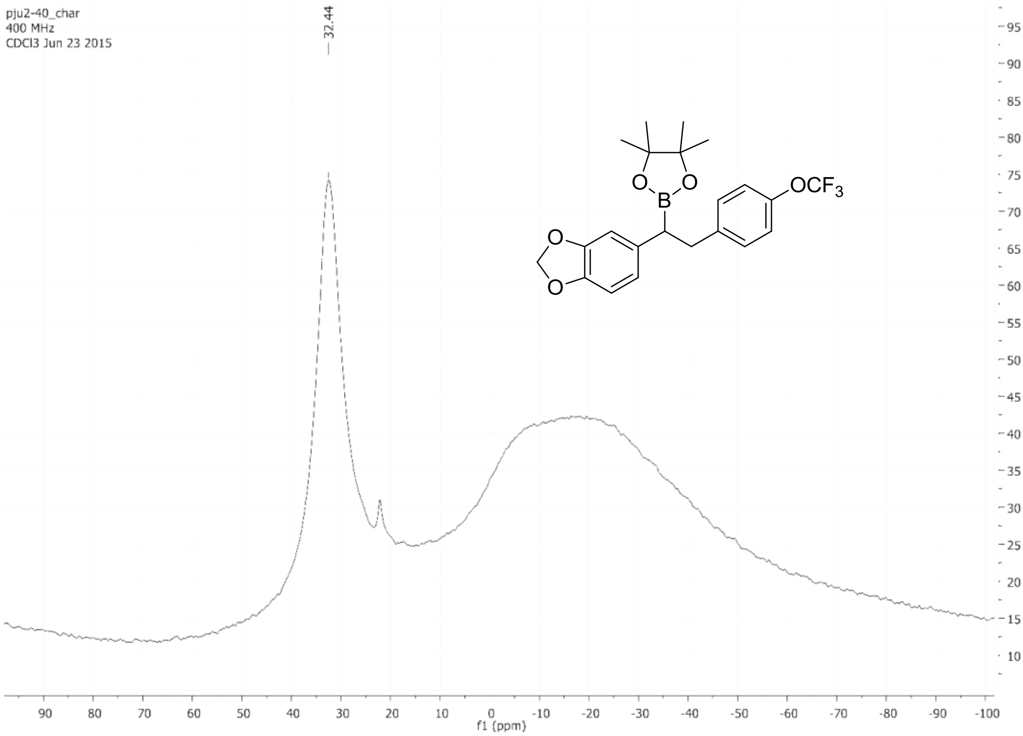

b)

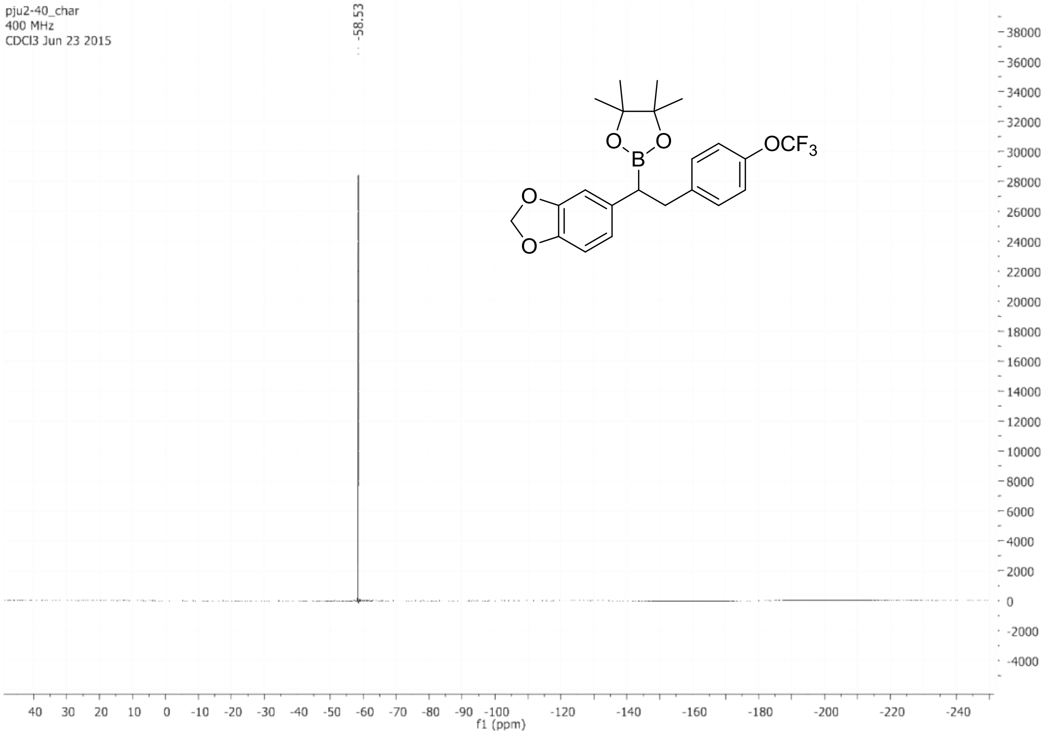

Supplementary Figure 29 | NMR spectra of 6gE. a) ^{11}B NMR spectrum. b) ^{19}F NMR spectrum.

a)

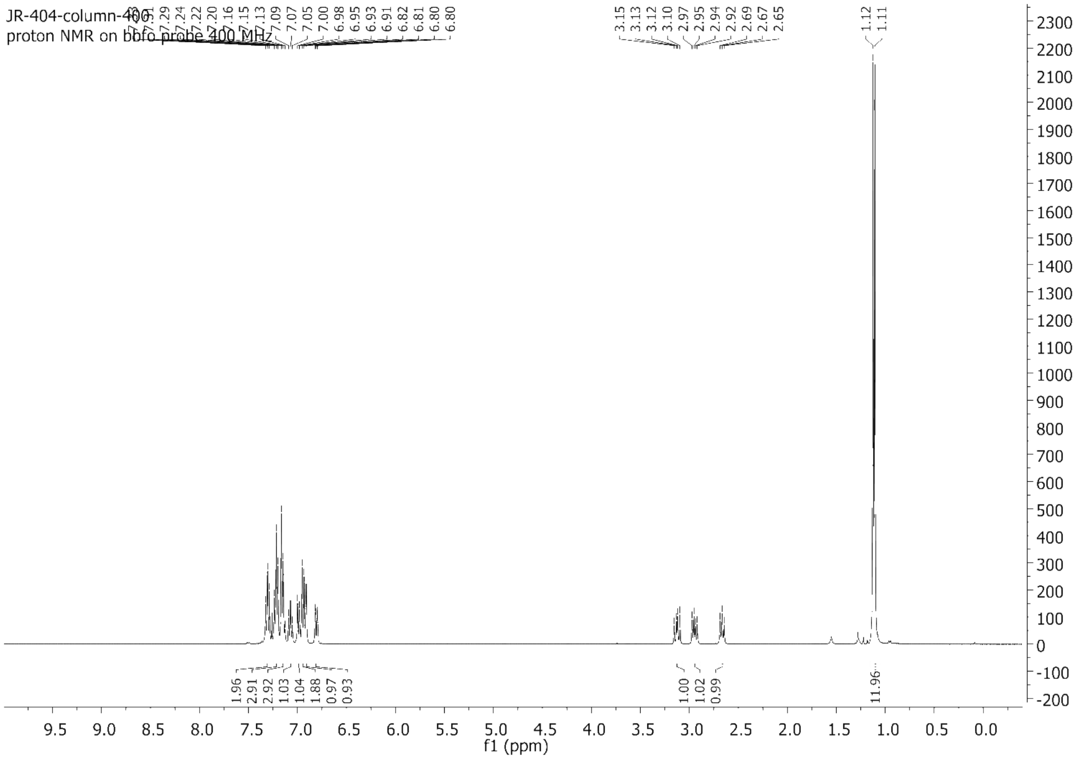

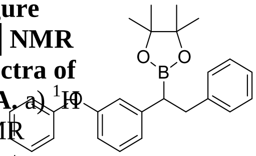

Supplementary Figure 30 | NMR spectra of 6hA. a) ^{1}H NMR spectrum. b) ^{13}C NMR spectrum.

b)

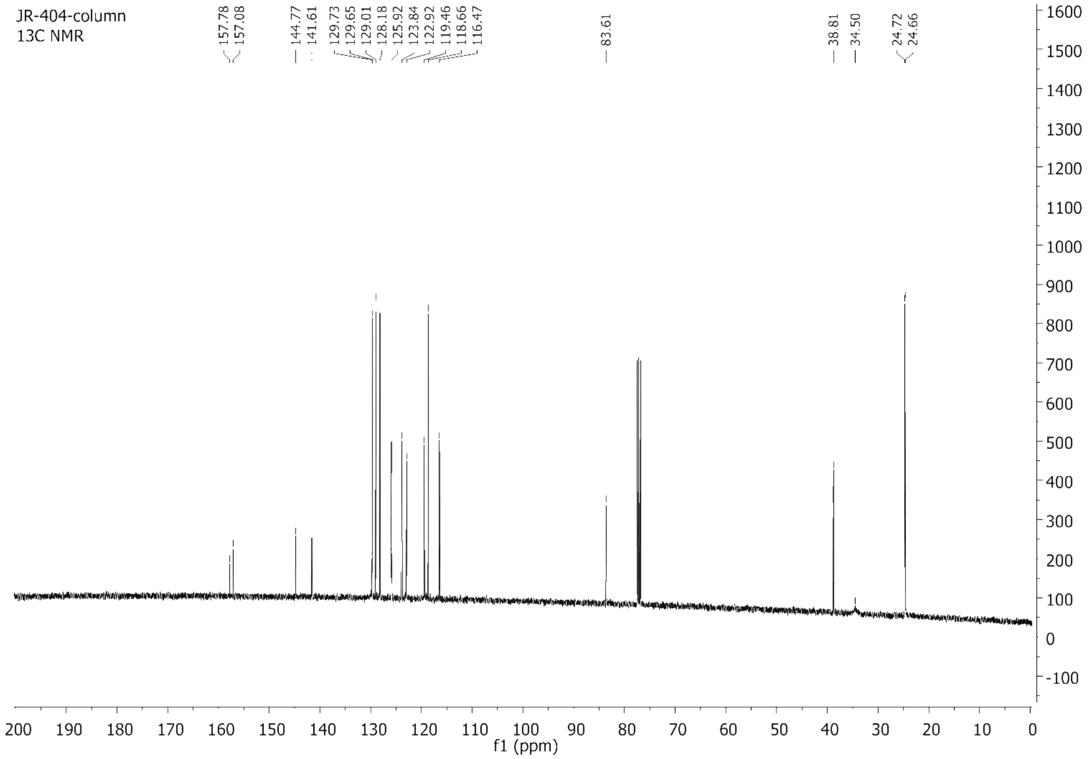

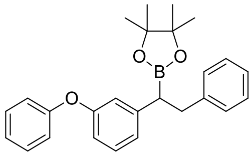

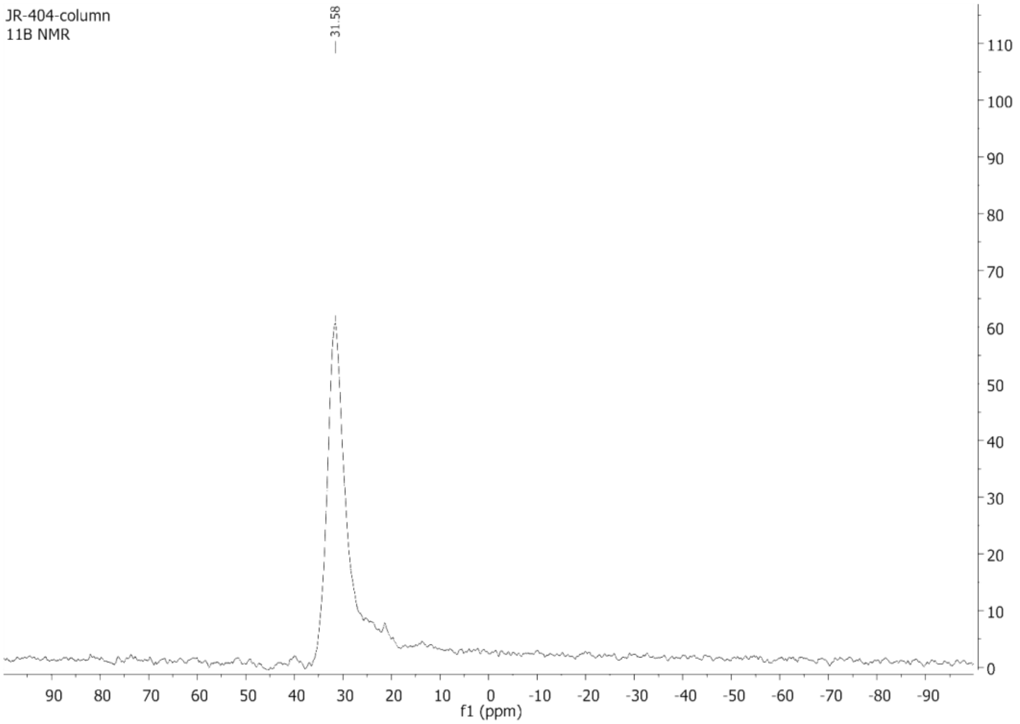

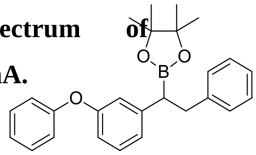

Supplementary Figure 31 | ^{11}B NMR spectrum of 6hA.

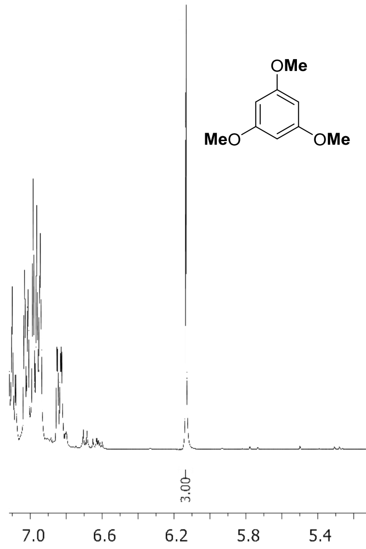

Supplementary Figure 32 | ^{1}H NMR spectrum used to determine NMR yield of 6hA. 1,3,5-Trimethoxybenzene used as internal standard.

a)

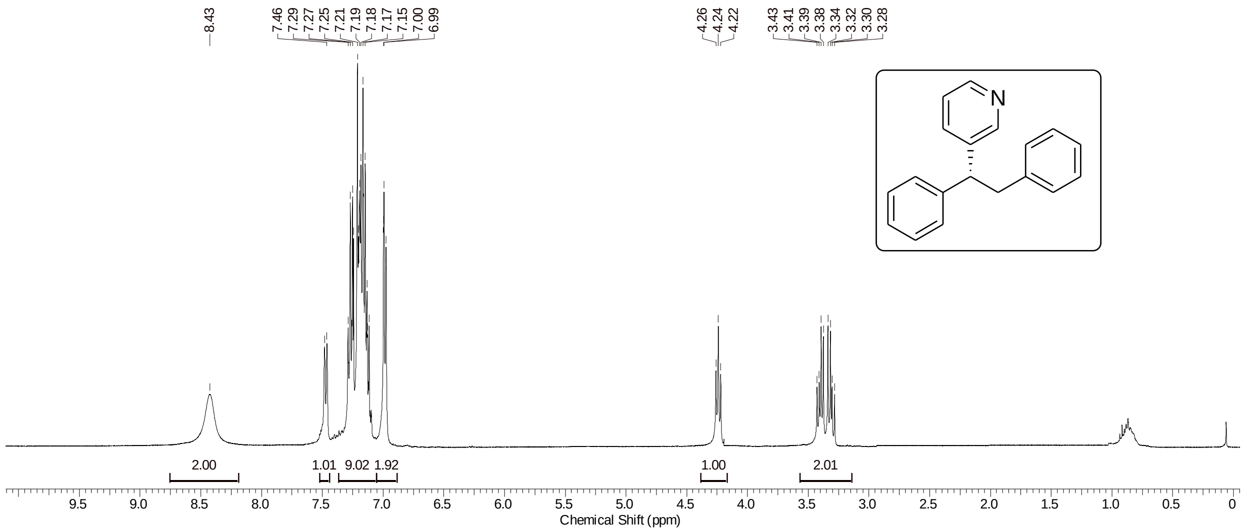

b)

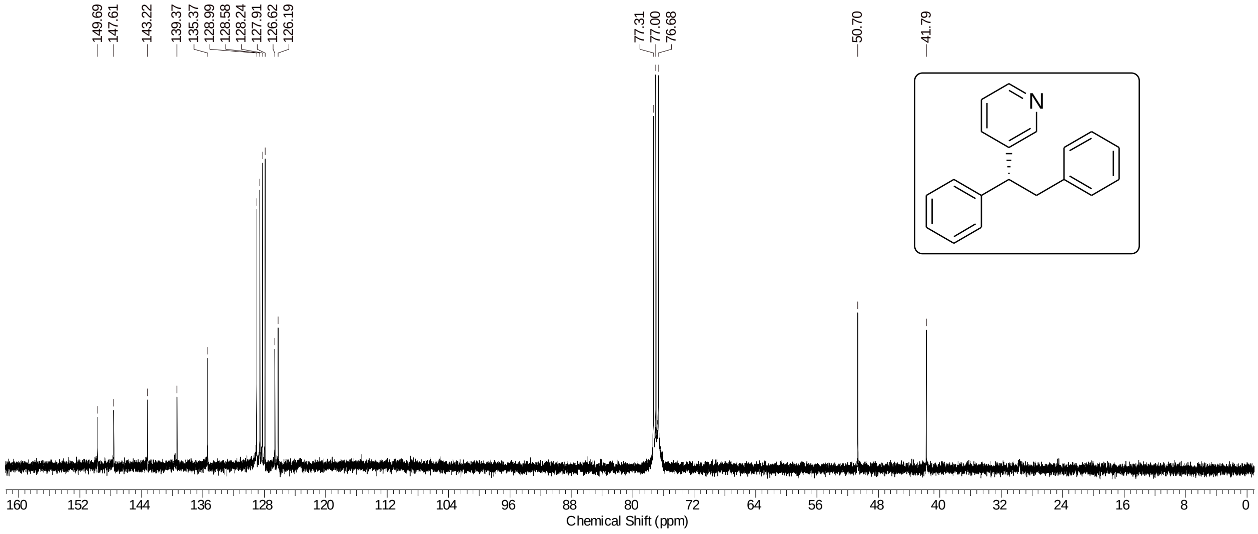

Supplementary Figure 33 | NMR spectra of 7aAi. a) ^{1}H NMR spectrum. b) ^{13}C NMR spectrum.

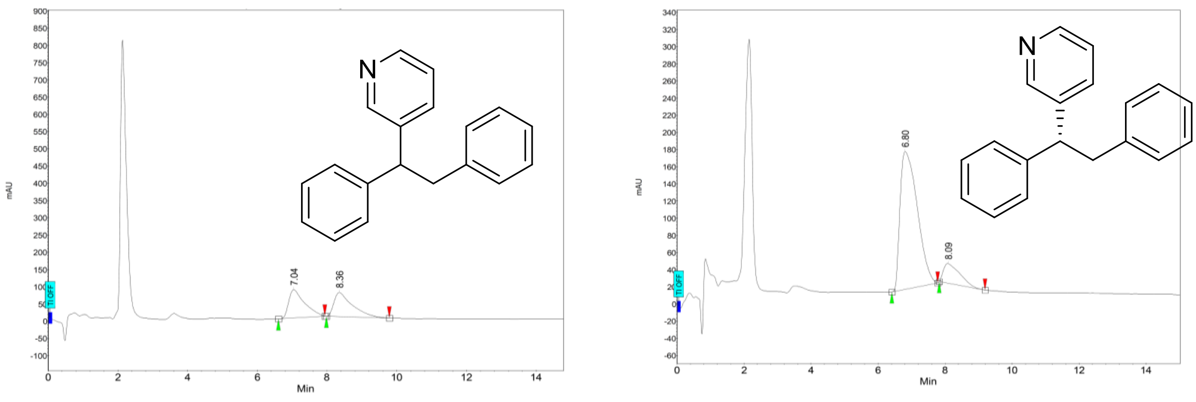

Supplementary Figure 34 | SFC traces of 7aAi.

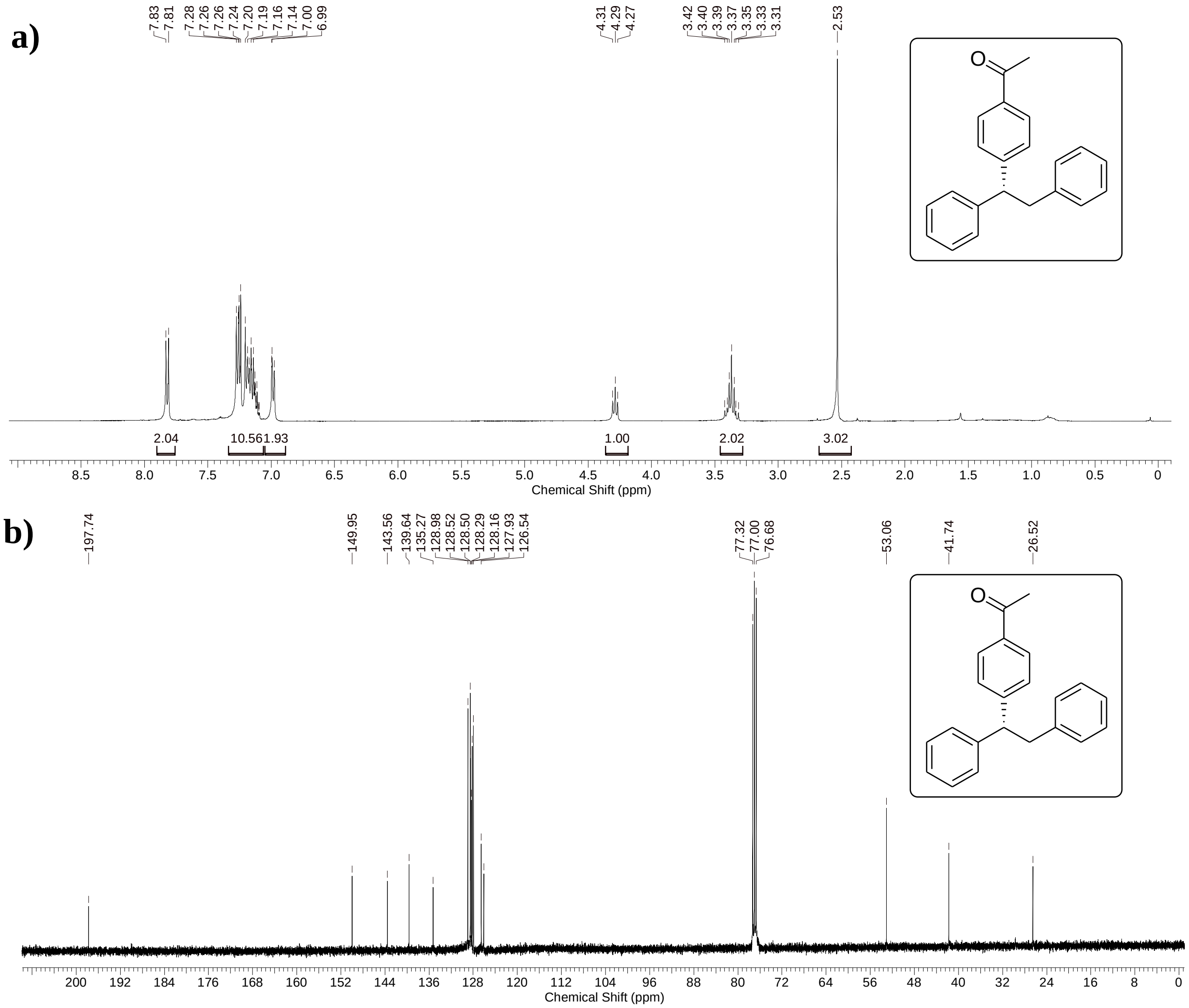

Supplementary Figure 35 │ NMR spectra of 7aAj. a) ^{1}H NMR spectrum. b) ^{13}C NMR spectrum.

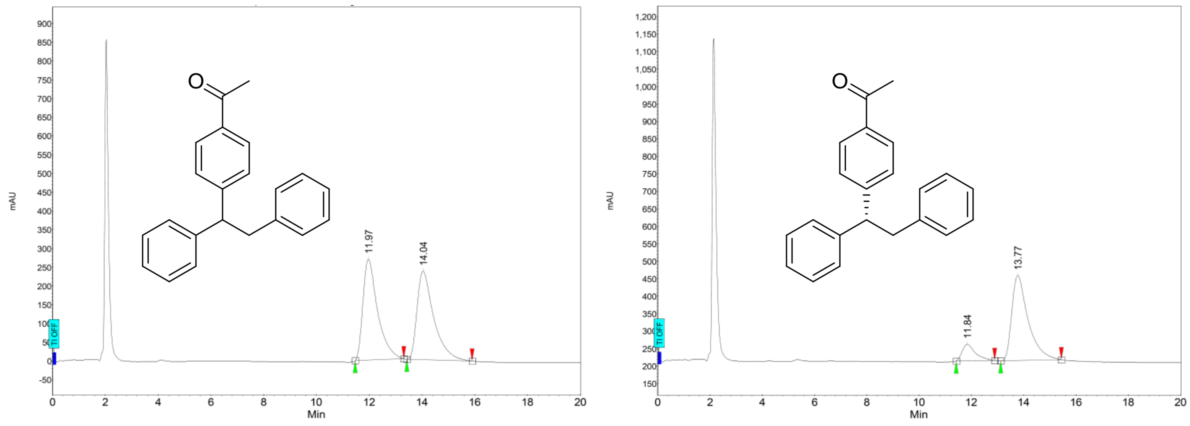

Supplementary Figure 36 │ SFC traces of 7aAj.

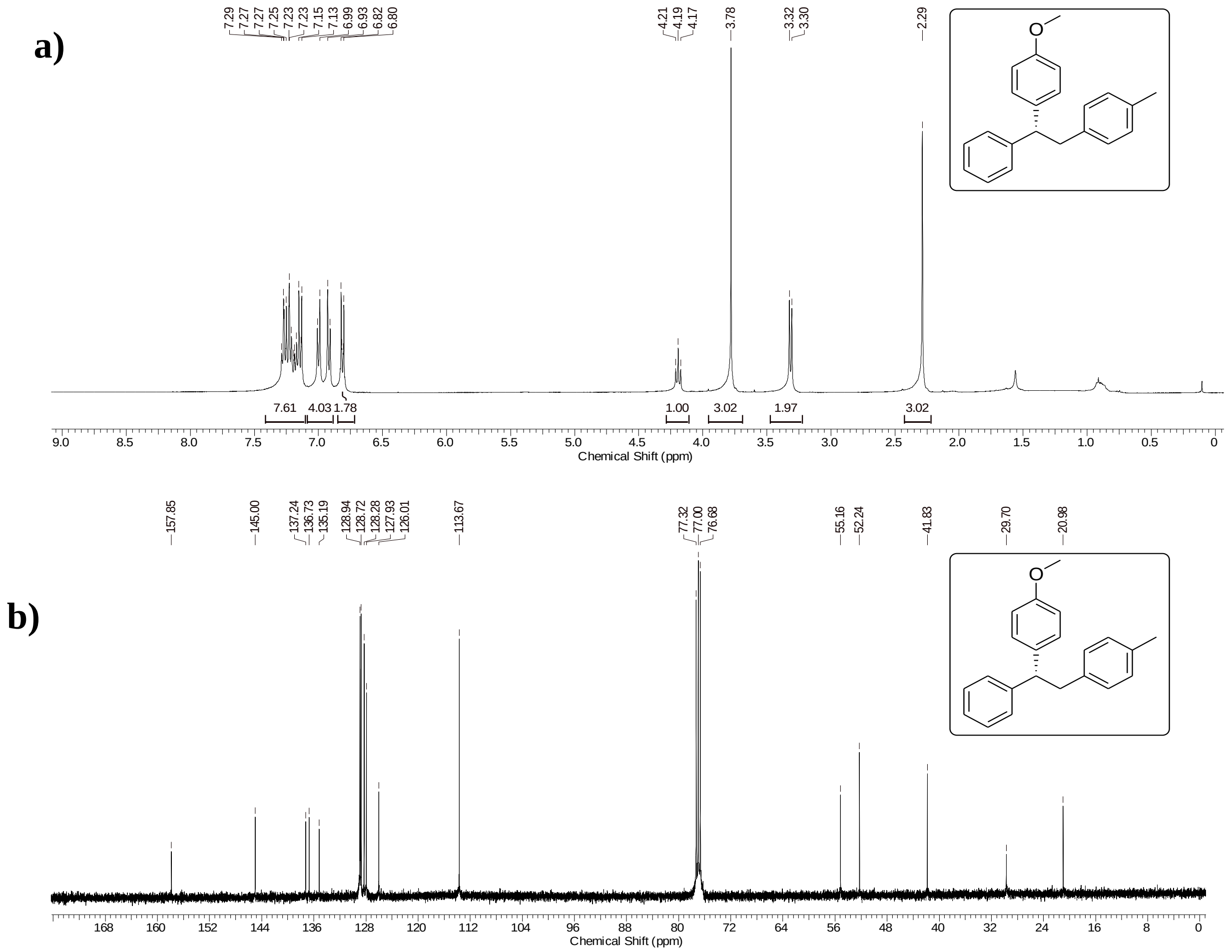

Supplementary Figure 37│NMR spectra of 7aBj. a) ^{1}H NMR spectrum. b) ^{13}C NMR spectrum.

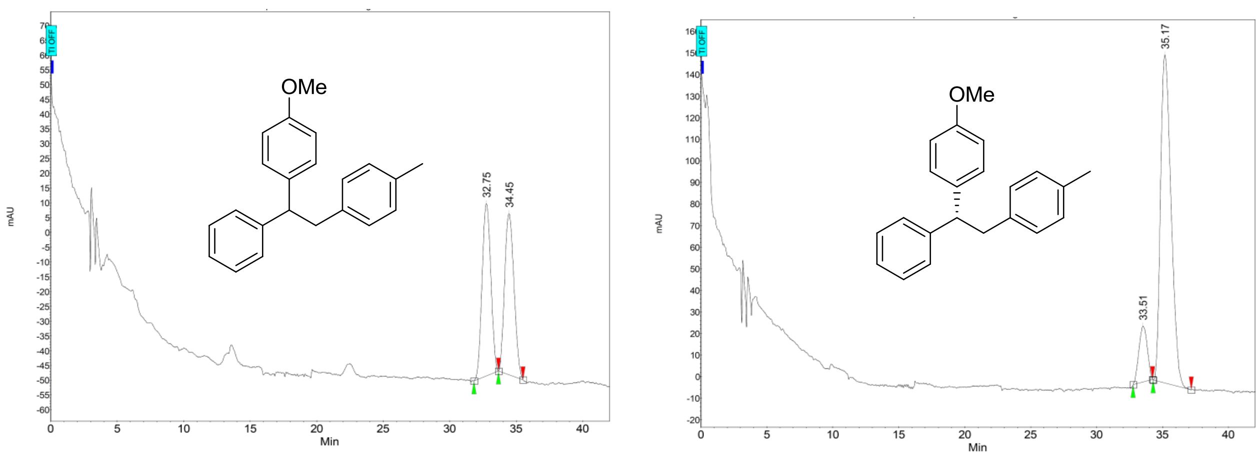

Supplementary Figure 38│SFC traces of 7aBj.

a)

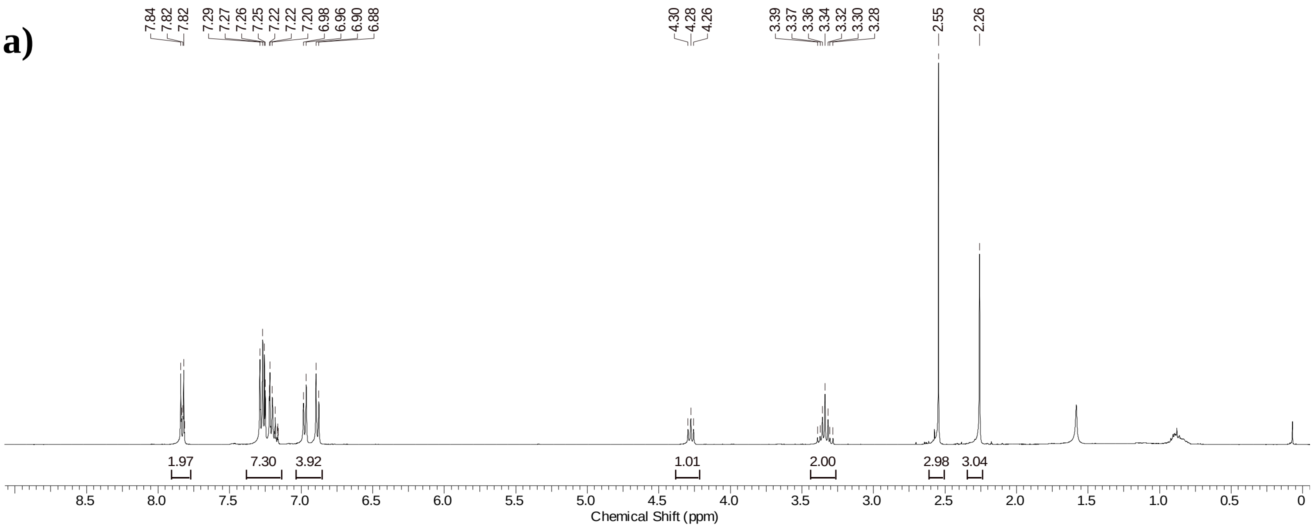

b)

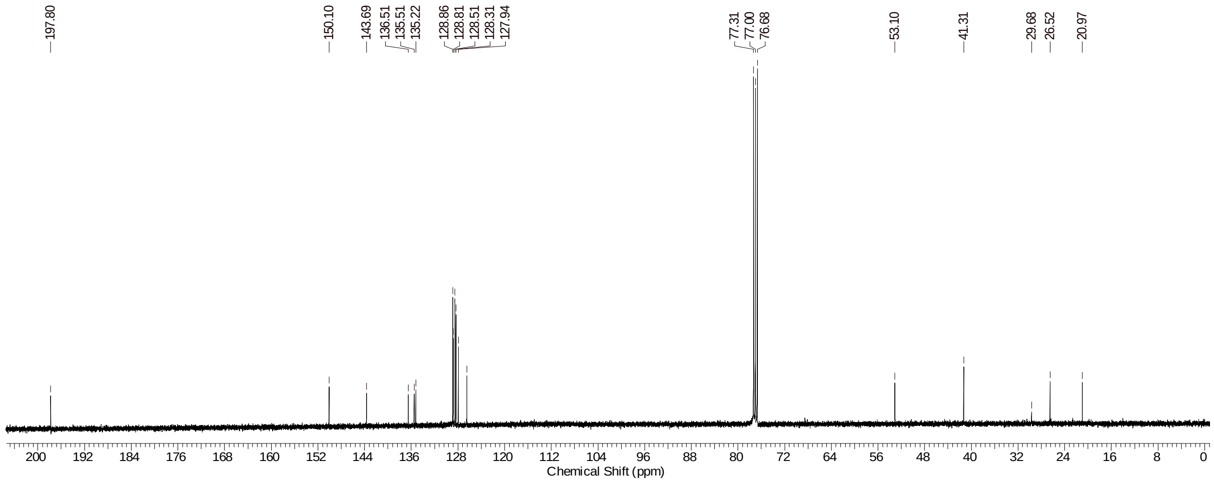

Supplementary Figure 39 | NMR spectra of 7aBj. a) ^{1}H NMR spectrum. b) ^{13}C NMR spectrum.

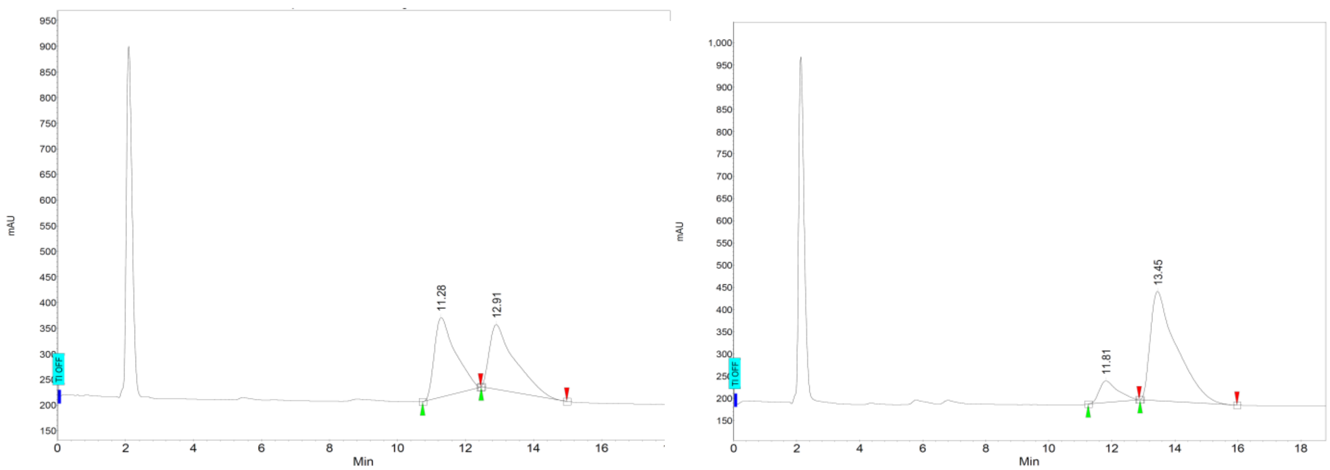

Supplementary Figure 40 | SFC traces of 7aBj.

a)

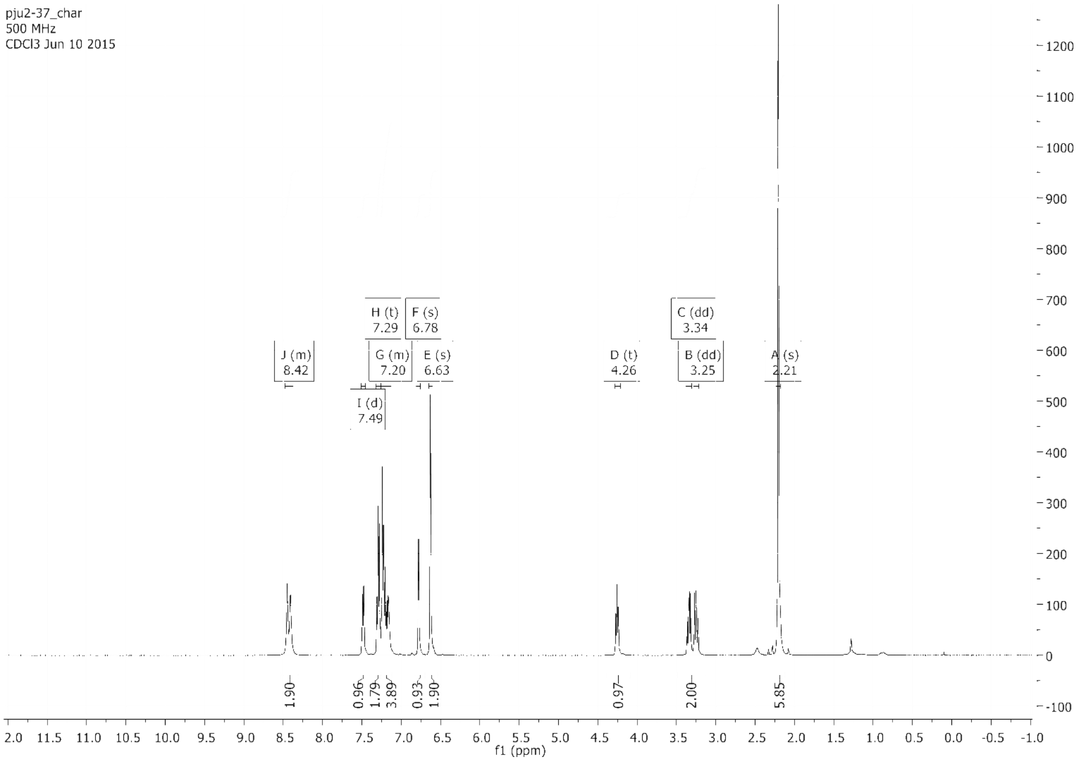

b)

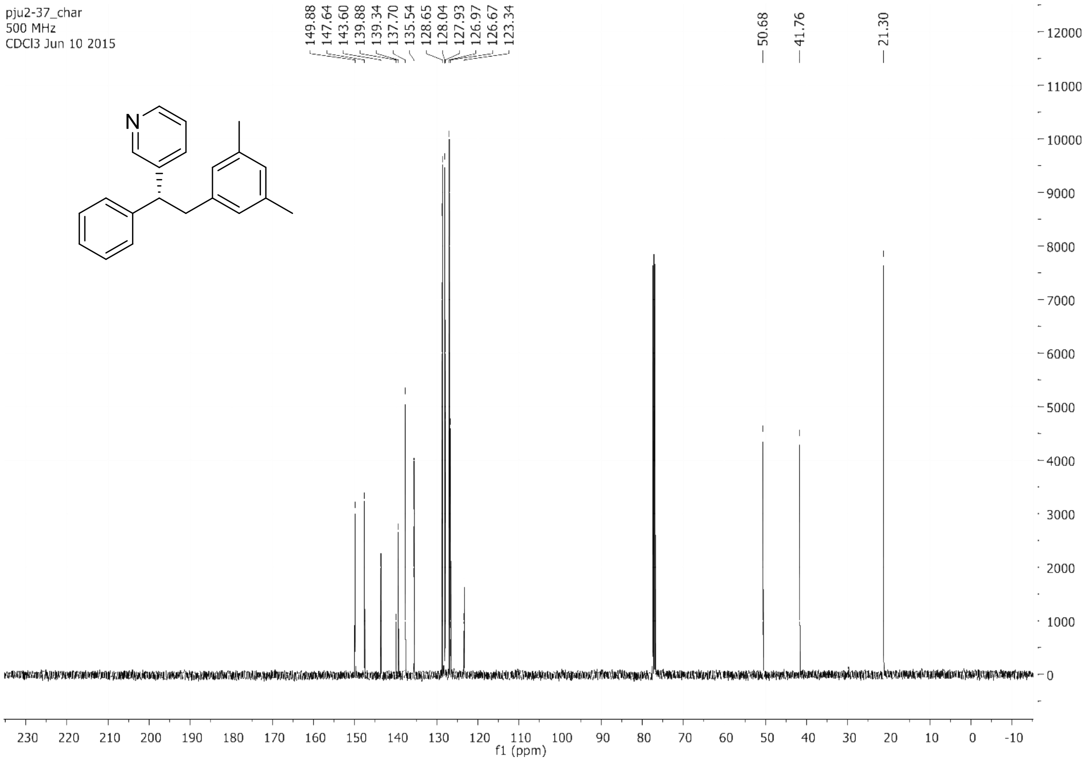

Supplementary Figure 41 | NMR spectra of 7aDi. a) ^{1}H NMR spectrum. b) ^{13}C NMR spectrum.

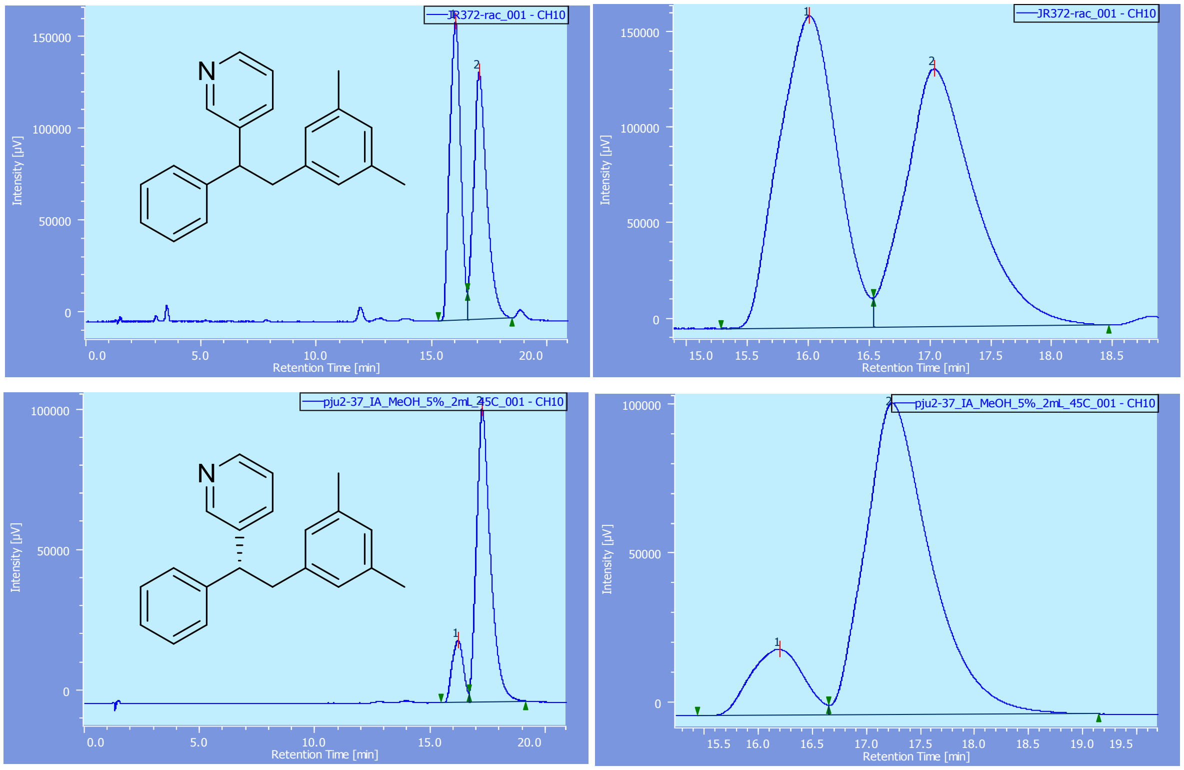

Supplementary Figure 42 | SFC traces of 7aDi.

a)

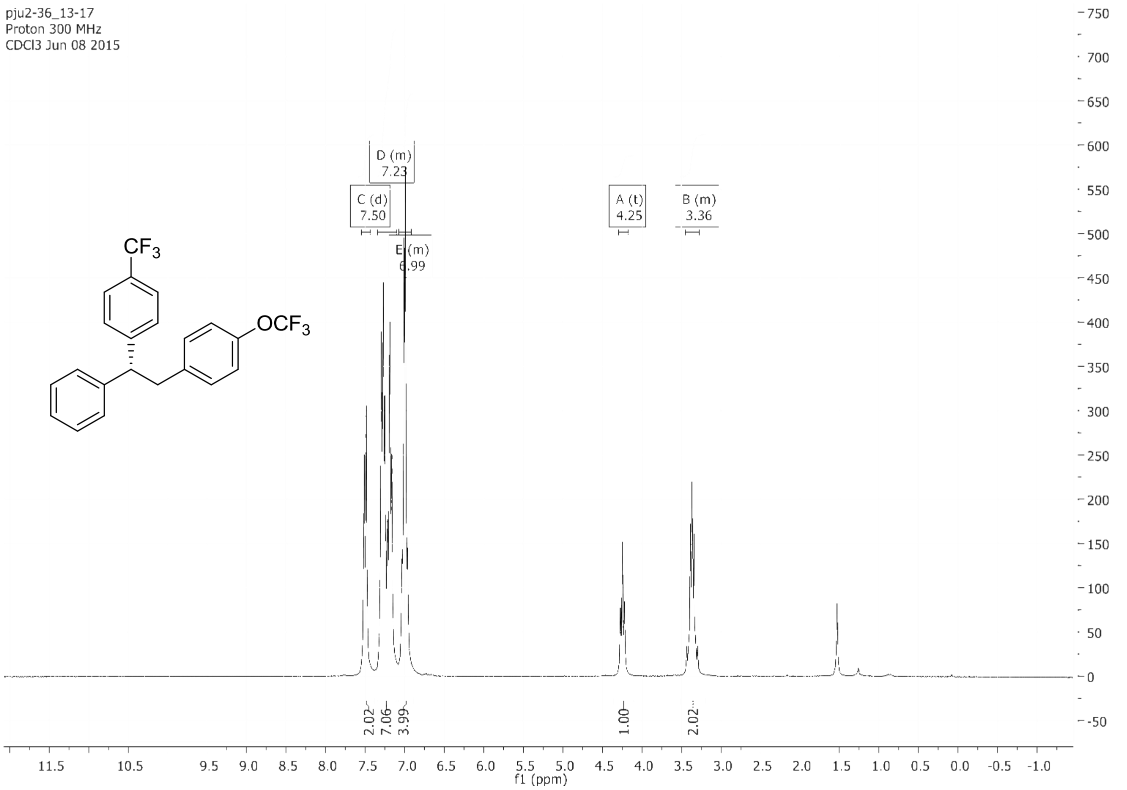

b)

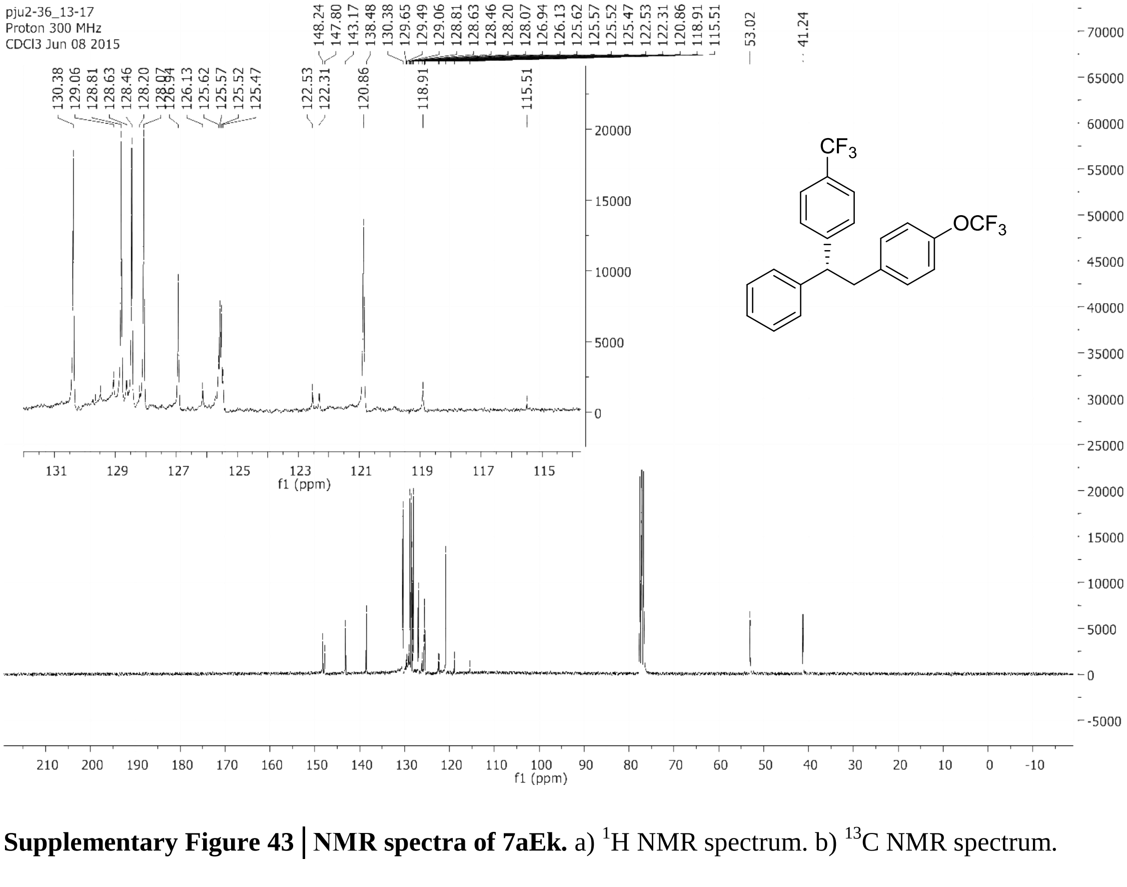

Supplementary Figure 43 | NMR spectra of 7aEk. a) ^{1}H NMR spectrum. b) ^{13}C NMR spectrum.

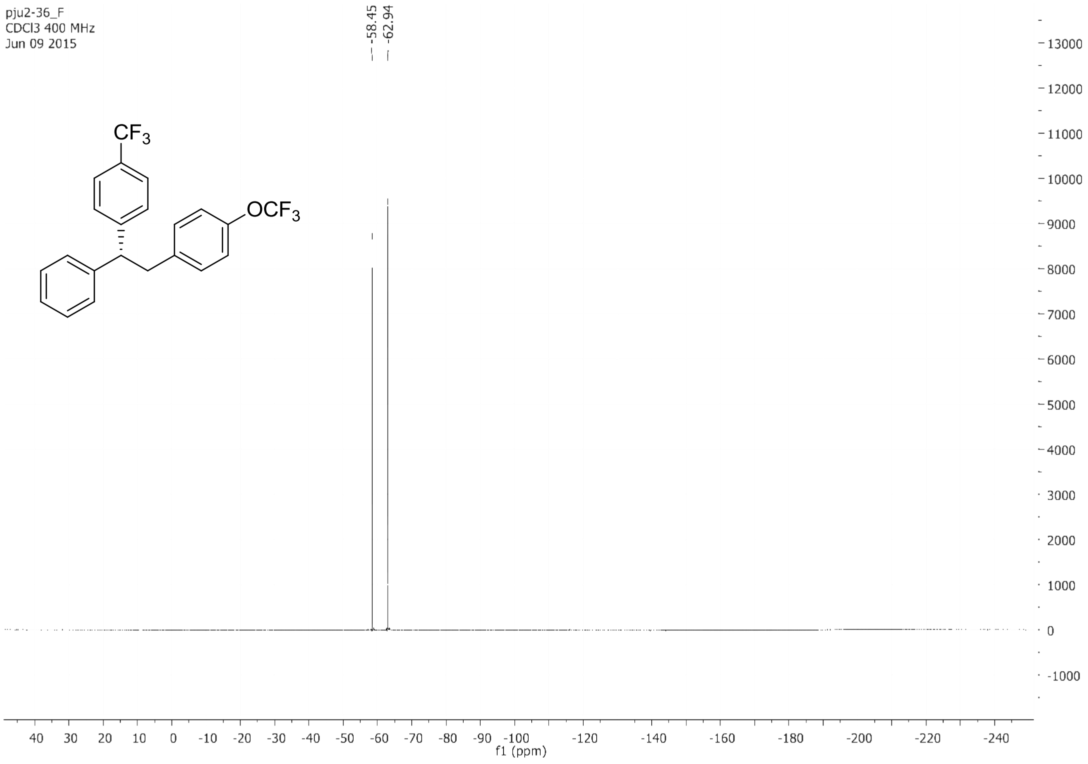

Supplementary Figure 44 | ^{19}F sprectrum of 7aEk.

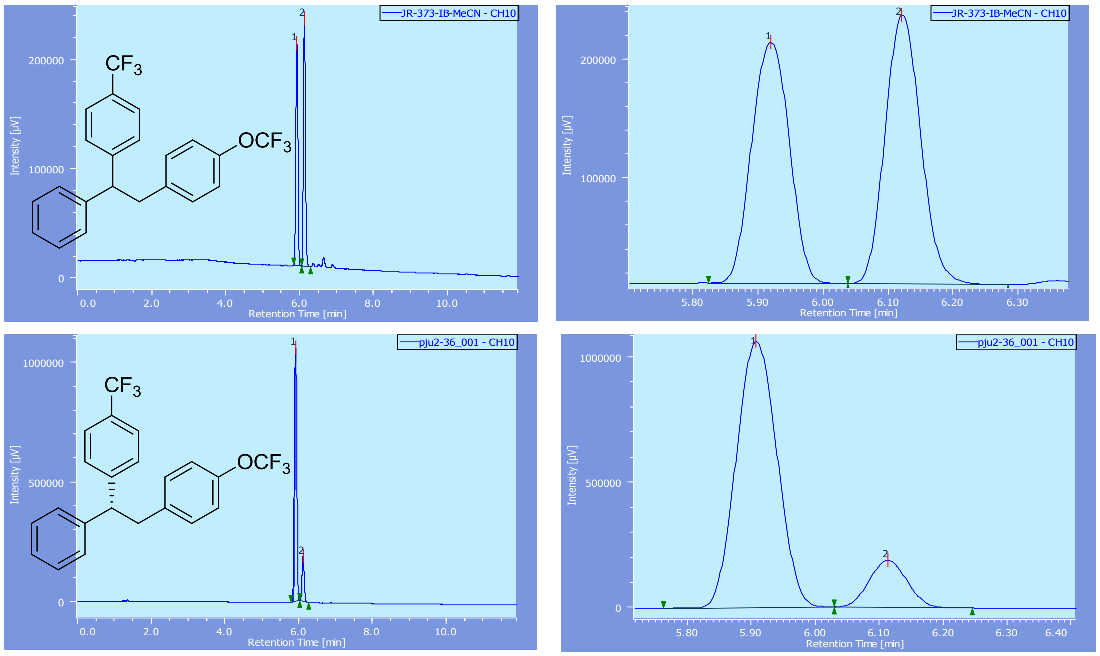

Supplementary Figure 45 | SFC traces of 7aEk.

a)

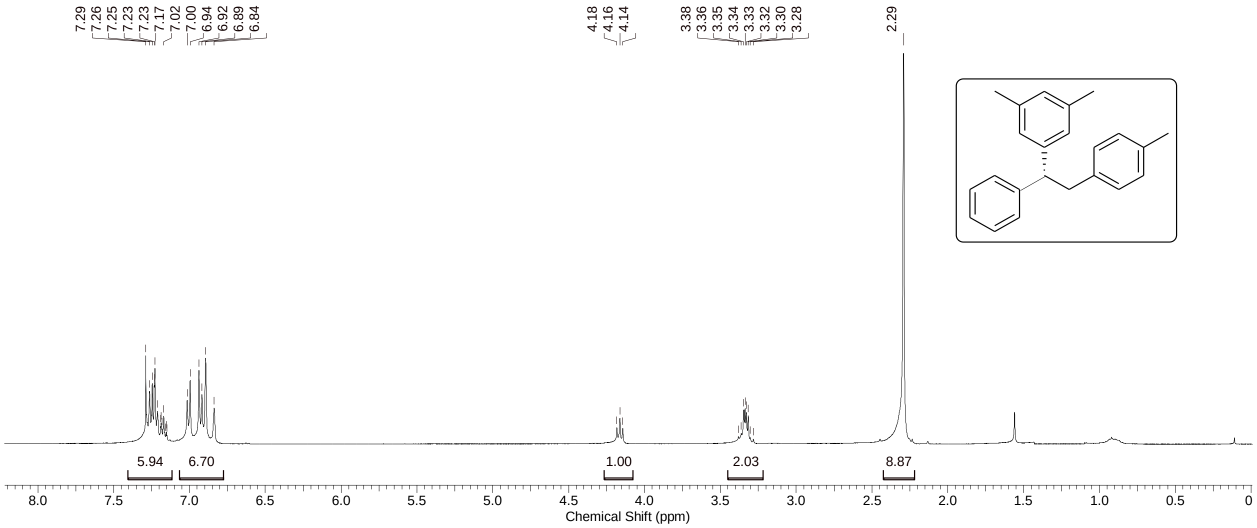

b)

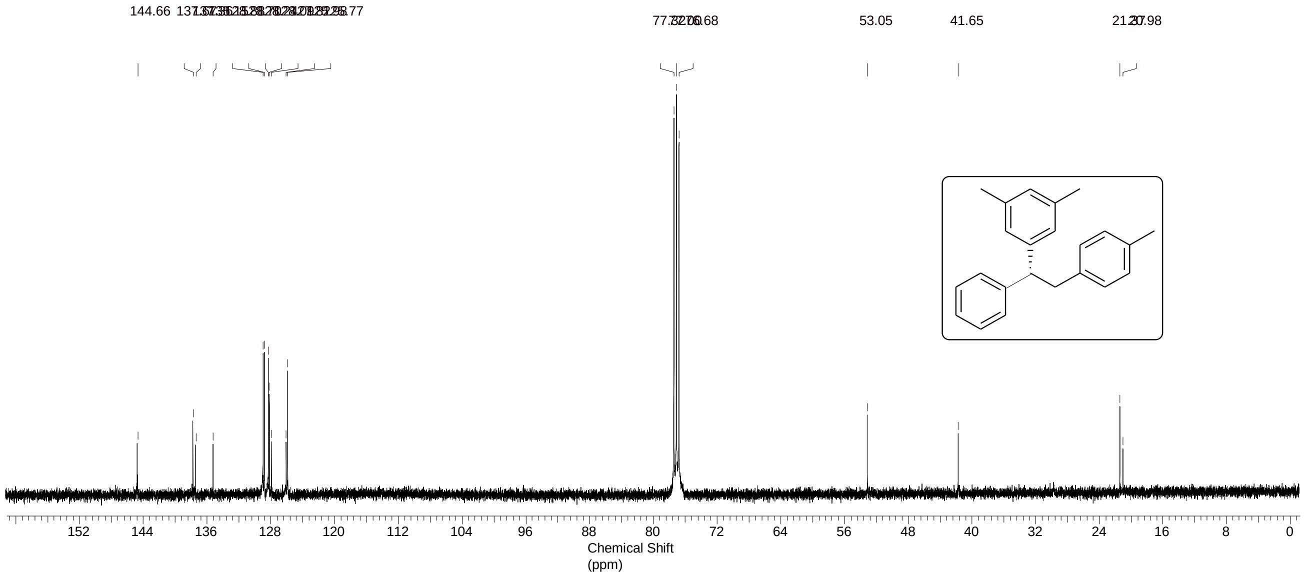

Supplementary Figure 46 | NMR spectra of 7aBd. a) ^{1}H NMR spectrum. b) ^{13}C NMR spectrum.

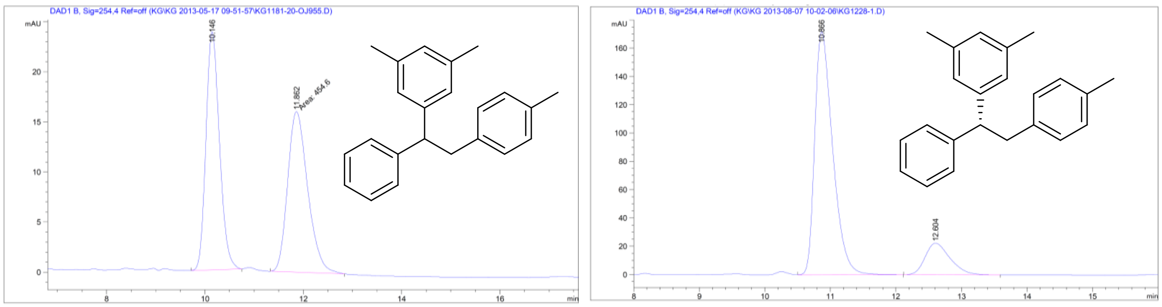

Supplementary Figure 47 | SFC spectra of 7aBd.

a)

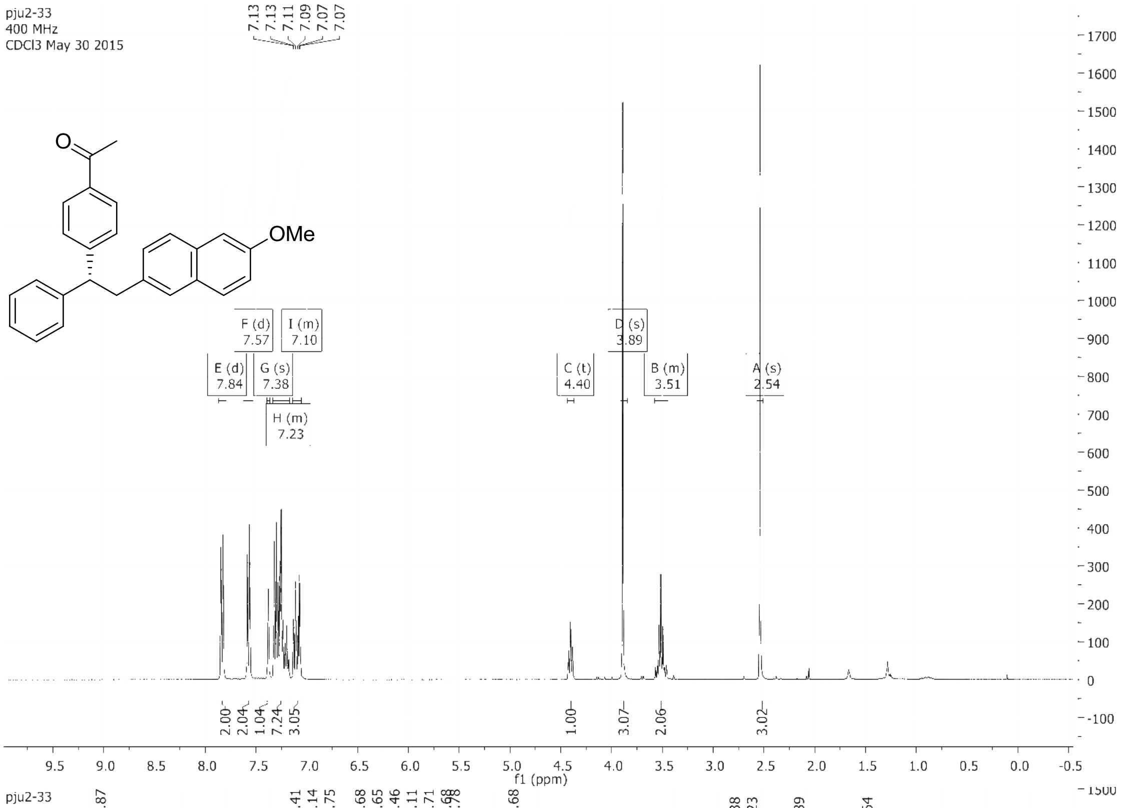

b)

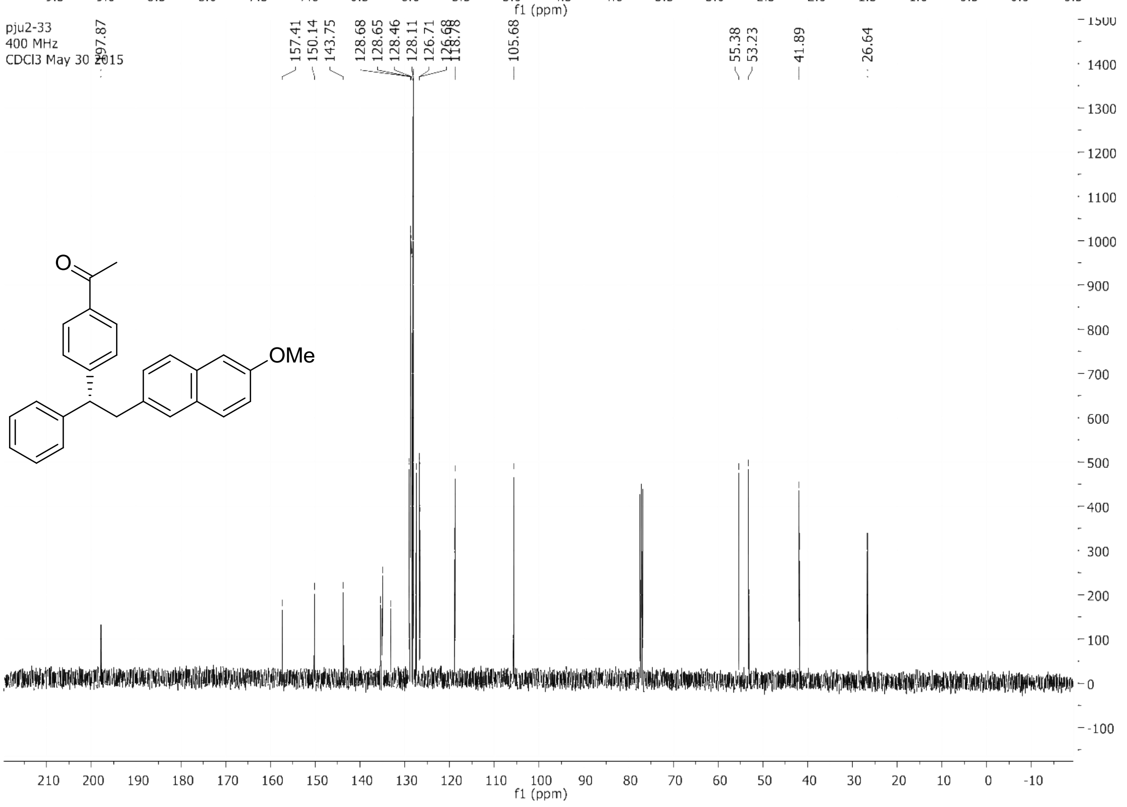

Supplementary Figure 48 | NMR spectra of 7aCj. a) ^{1}H NMR spectrum. b) ^{13}C NMR spectrum.

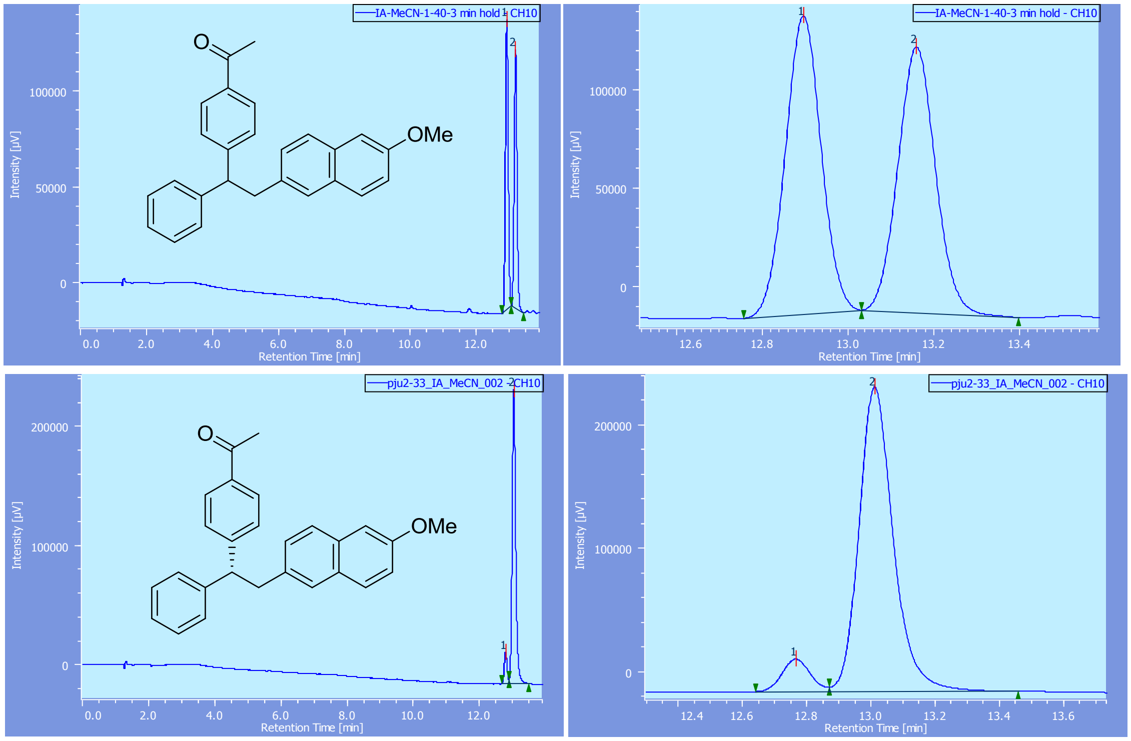

Supplementary Figure 49 | SFC traces of 7aCj.

a)

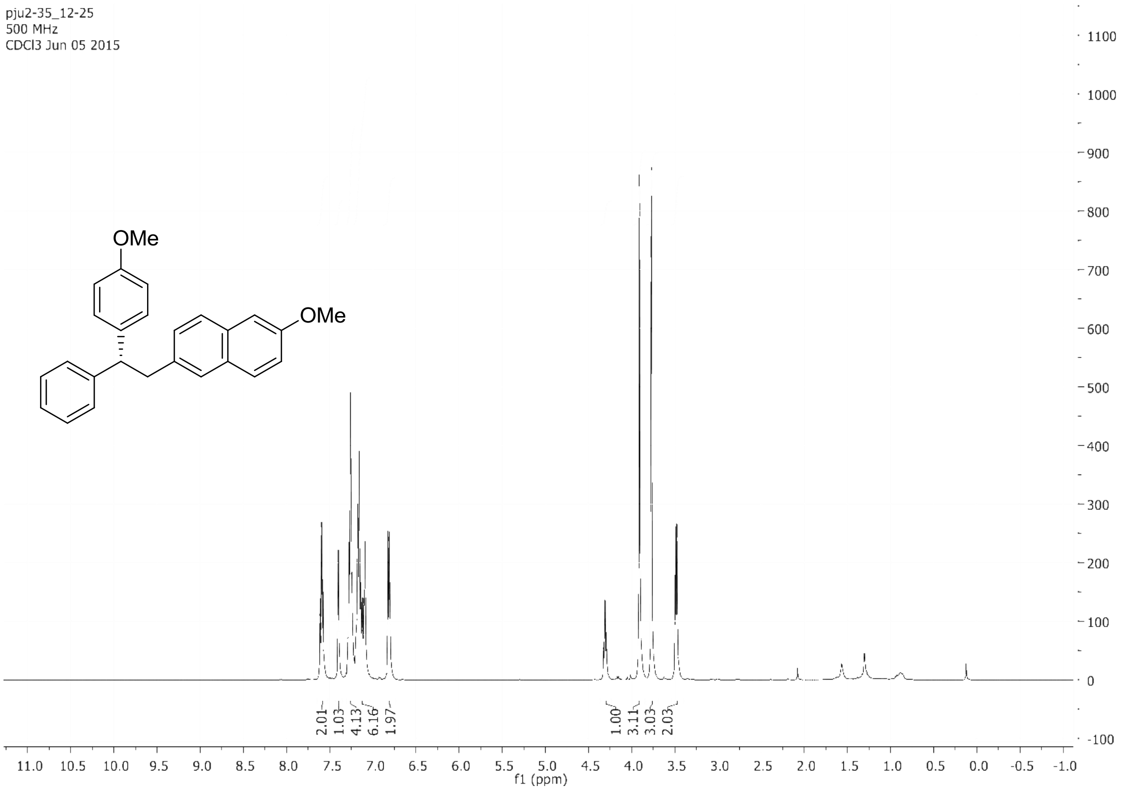

b)

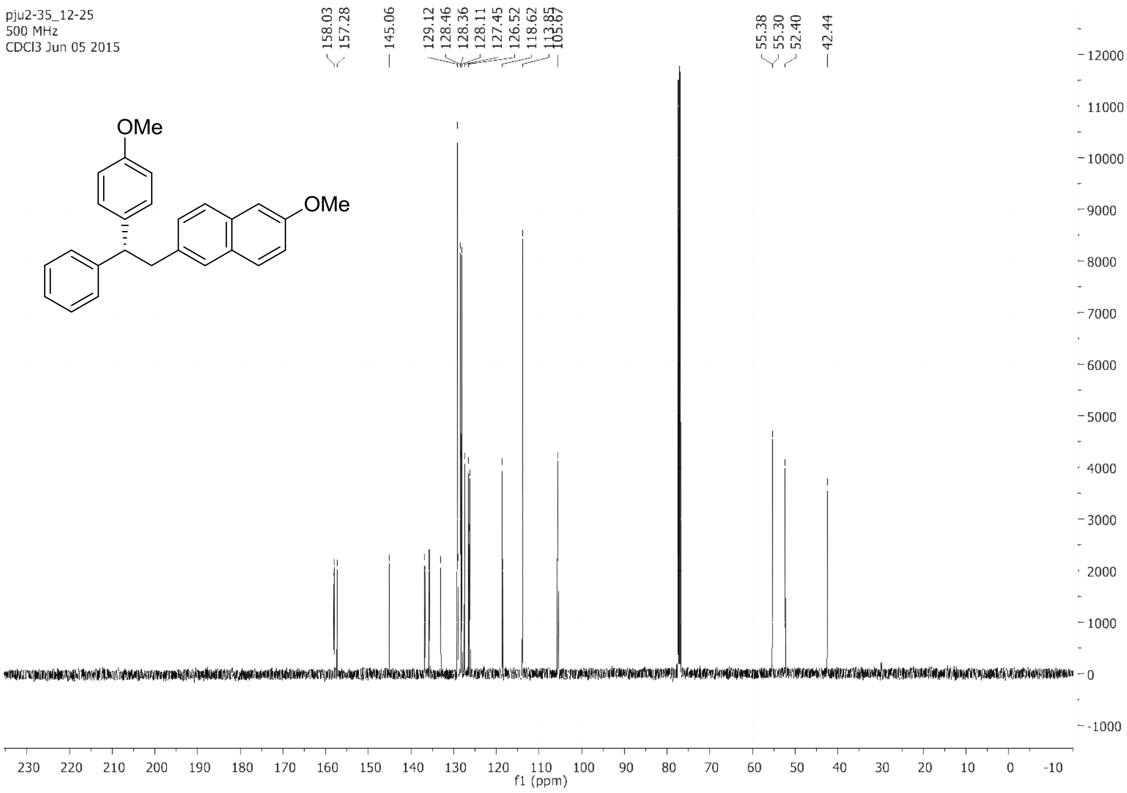

Supplementary Figure 50 | NMR spectra of 7aCf. a) ^{1}H NMR spectrum. b) ^{13}C NMR spectrum.

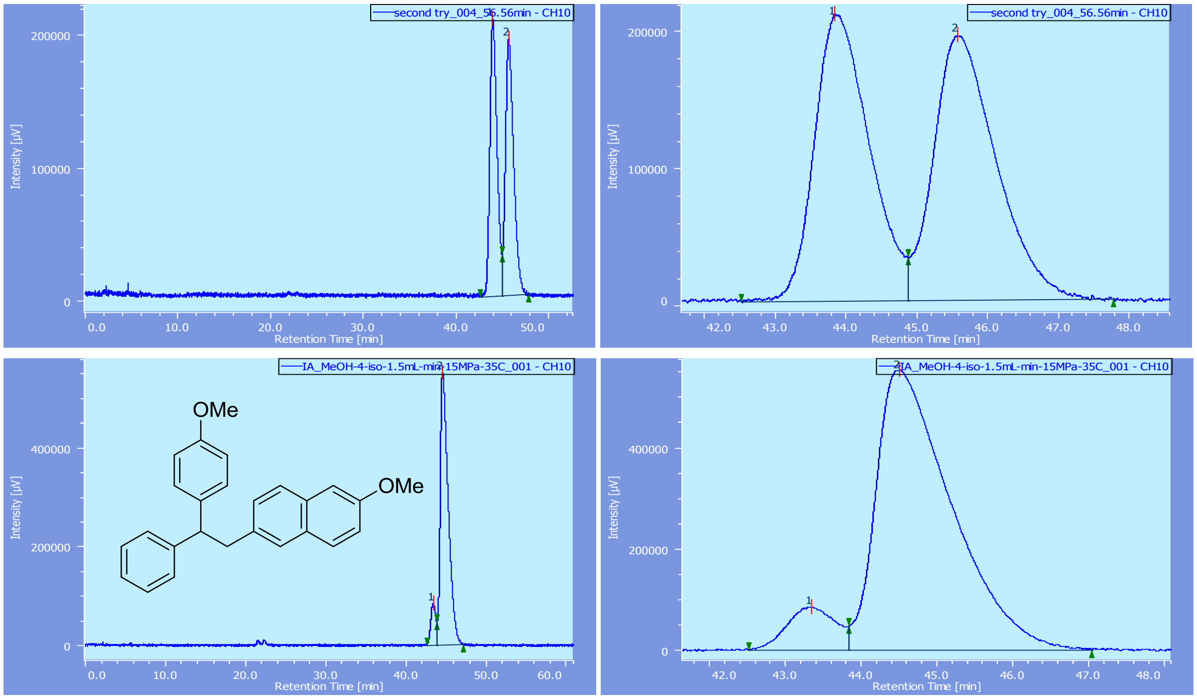

Supplementary Figure 51 | SFC traces of 7aCf

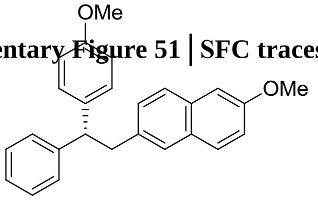

a)

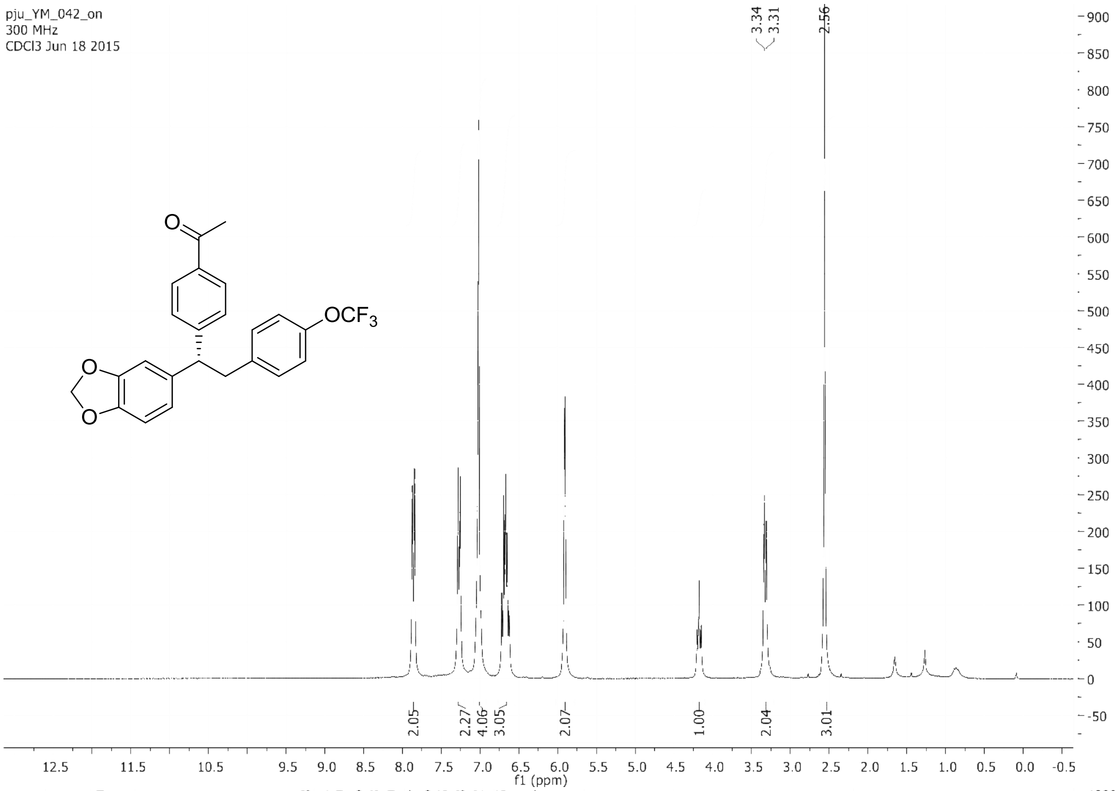

b)

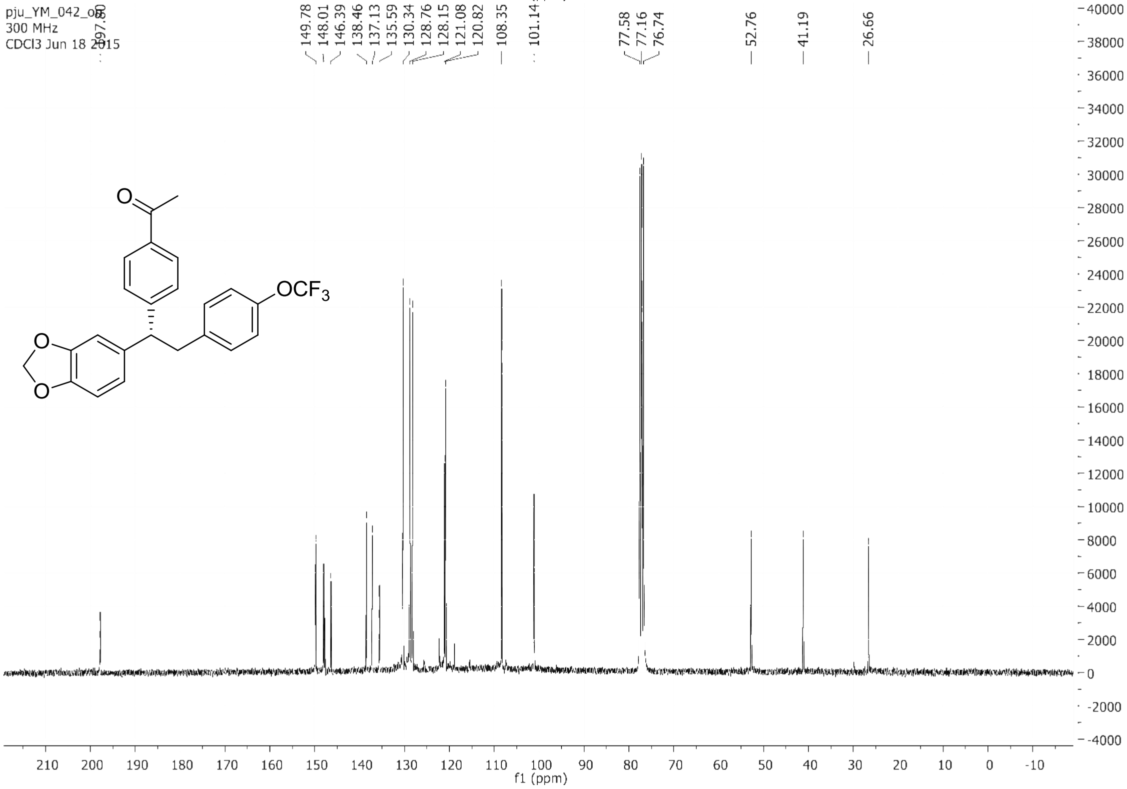

Supplementary Figure 52 | NMR spectra of 7hEj. a) ^{1}H NMR spectrum. b) ^{13}C NMR spectrum.

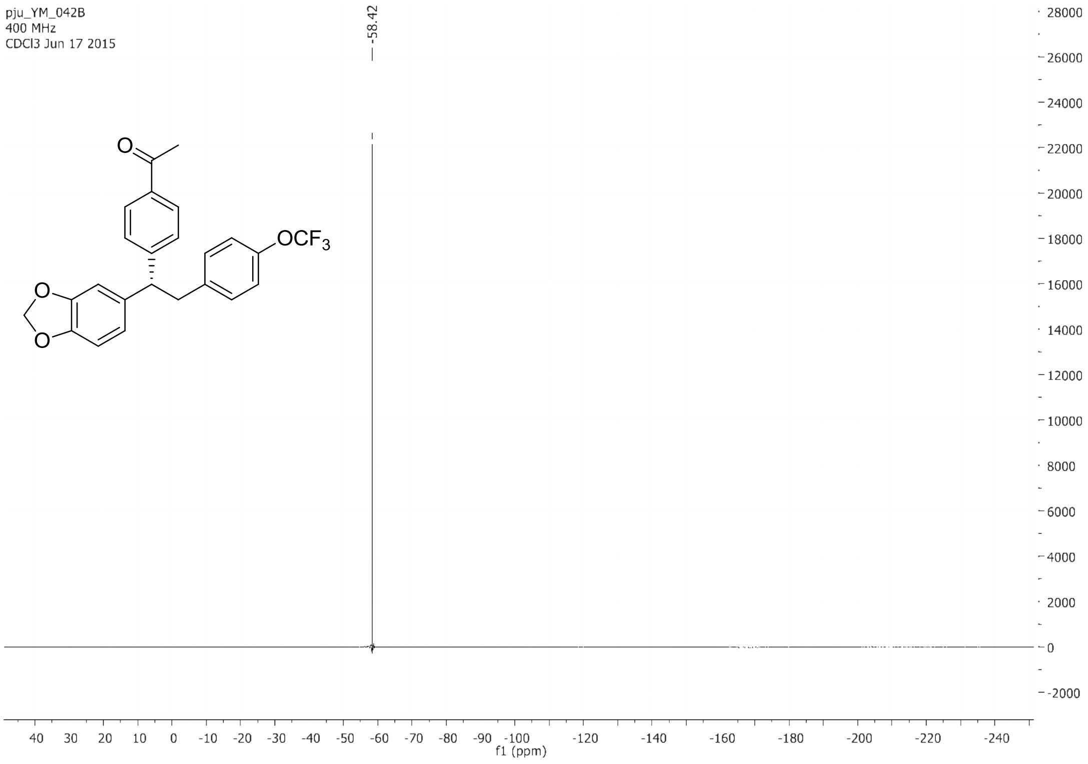

Supplementary Figure 53 | ^{19}F NMR spectrum of 7hEj.

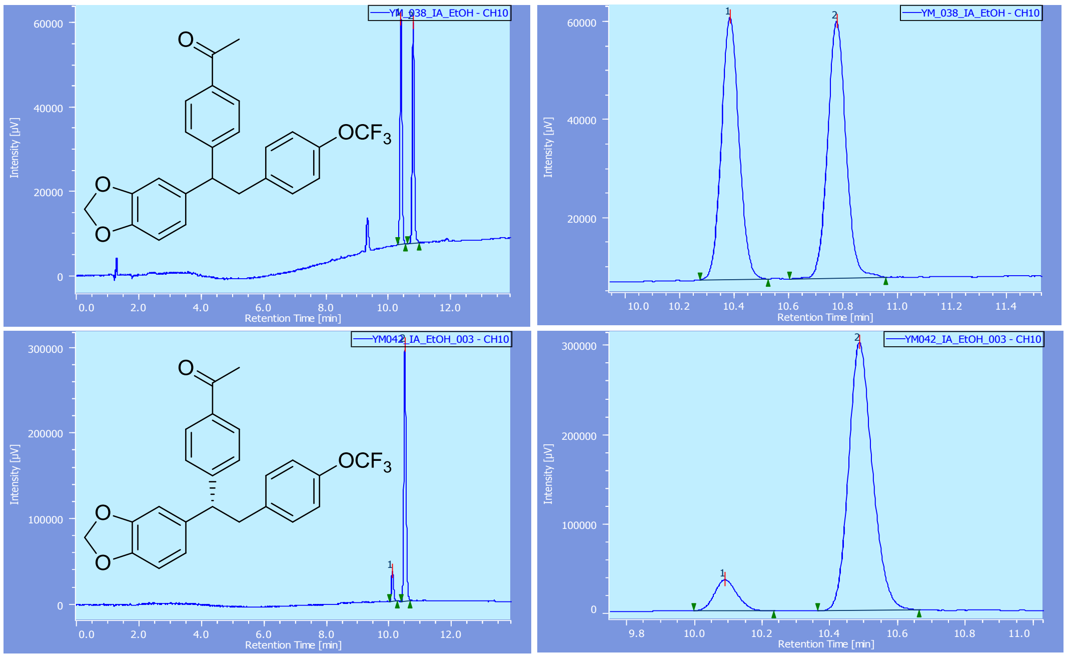

Supplementary Figure 54 | SFC traces of 7hEj.

a)

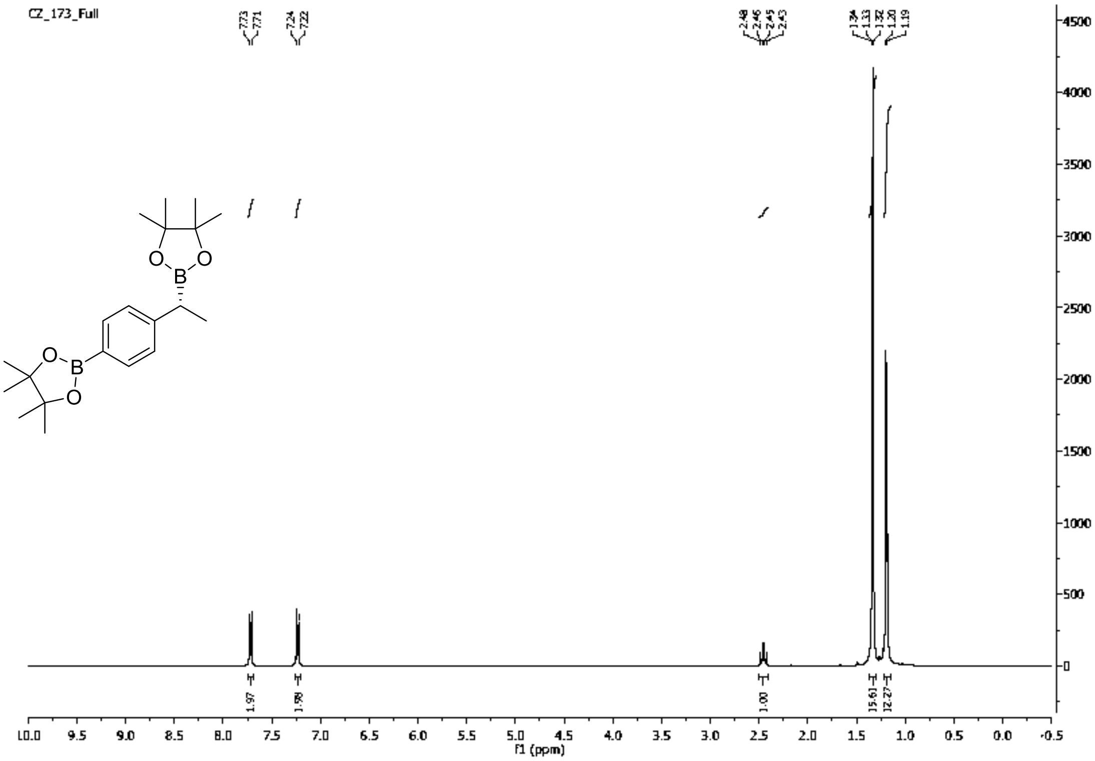

b)

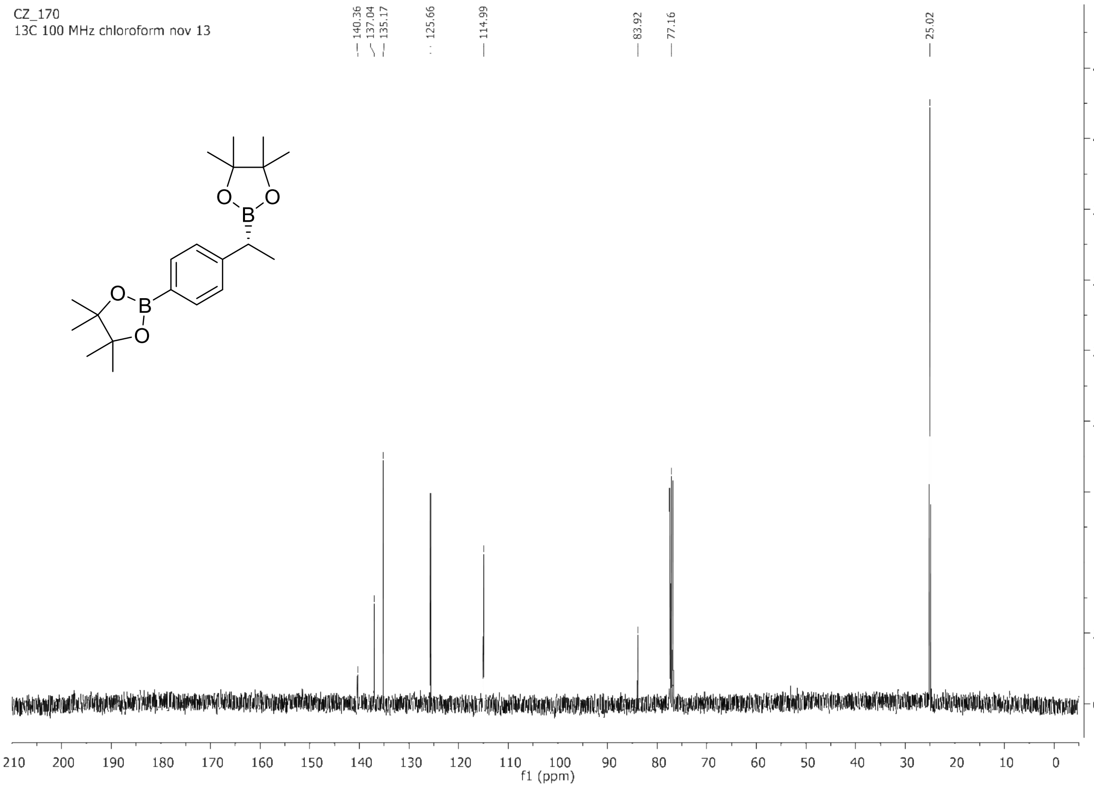

Supplementary Figure 55 | NMR spectra of 8. a) ^{1}H NMR spectrum. b) ^{13}C NMR spectrum.

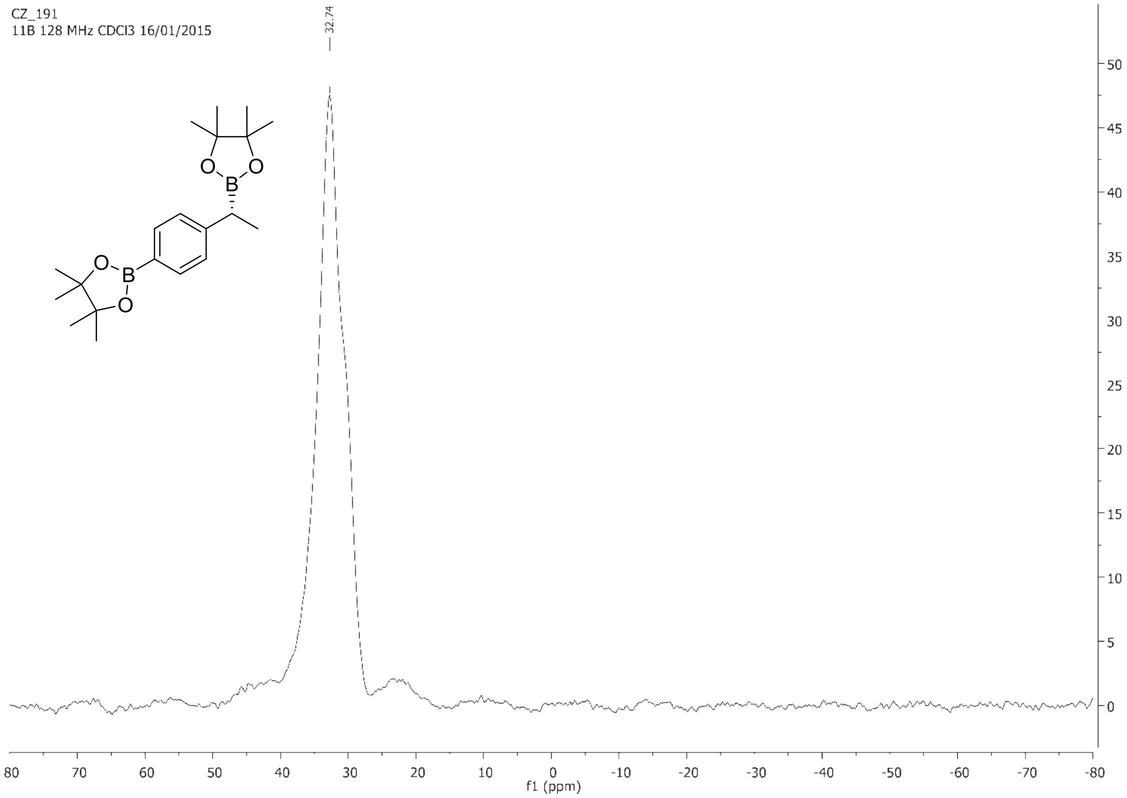

Supplementary Figure 56 | ^{11}B NMR spectrum of 8.

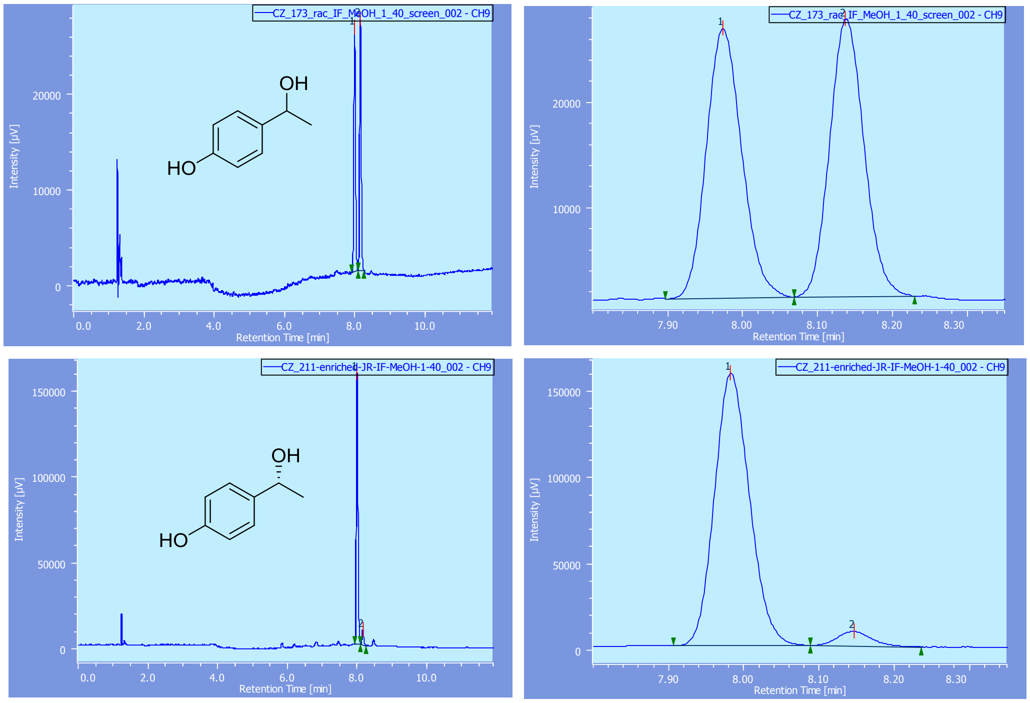

Supplementary Figure 57 | SFC spectra of oxidized 8.

a)

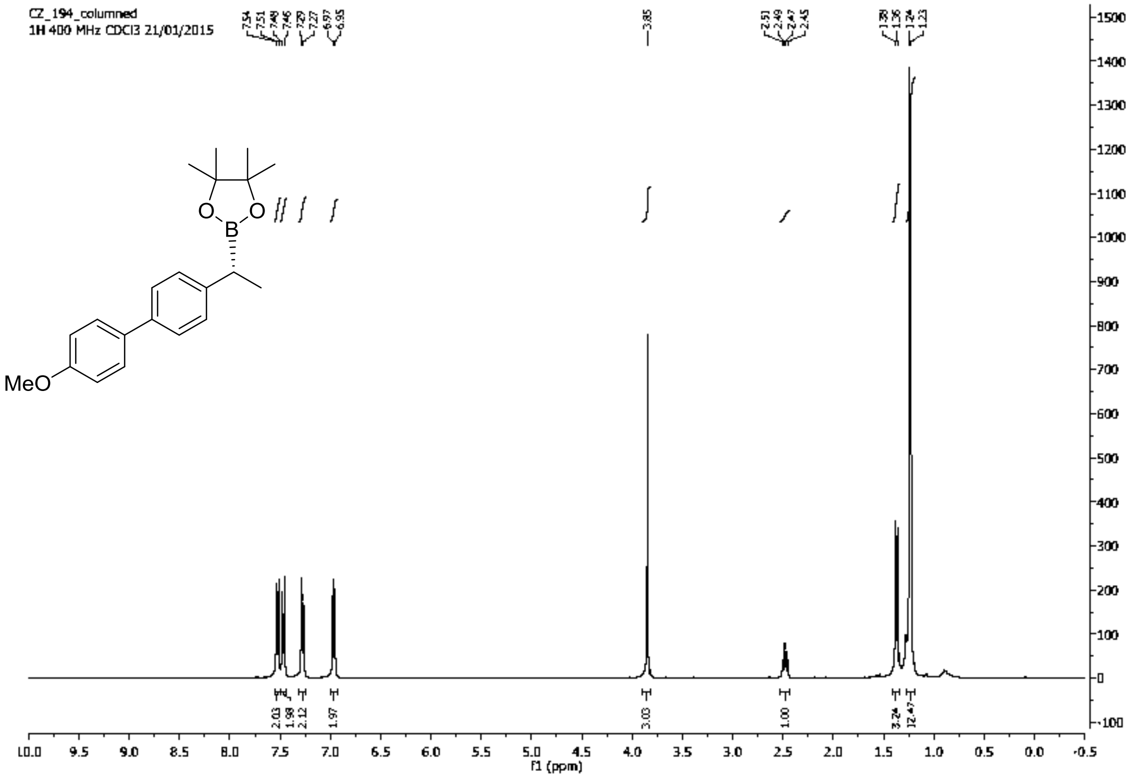

b)

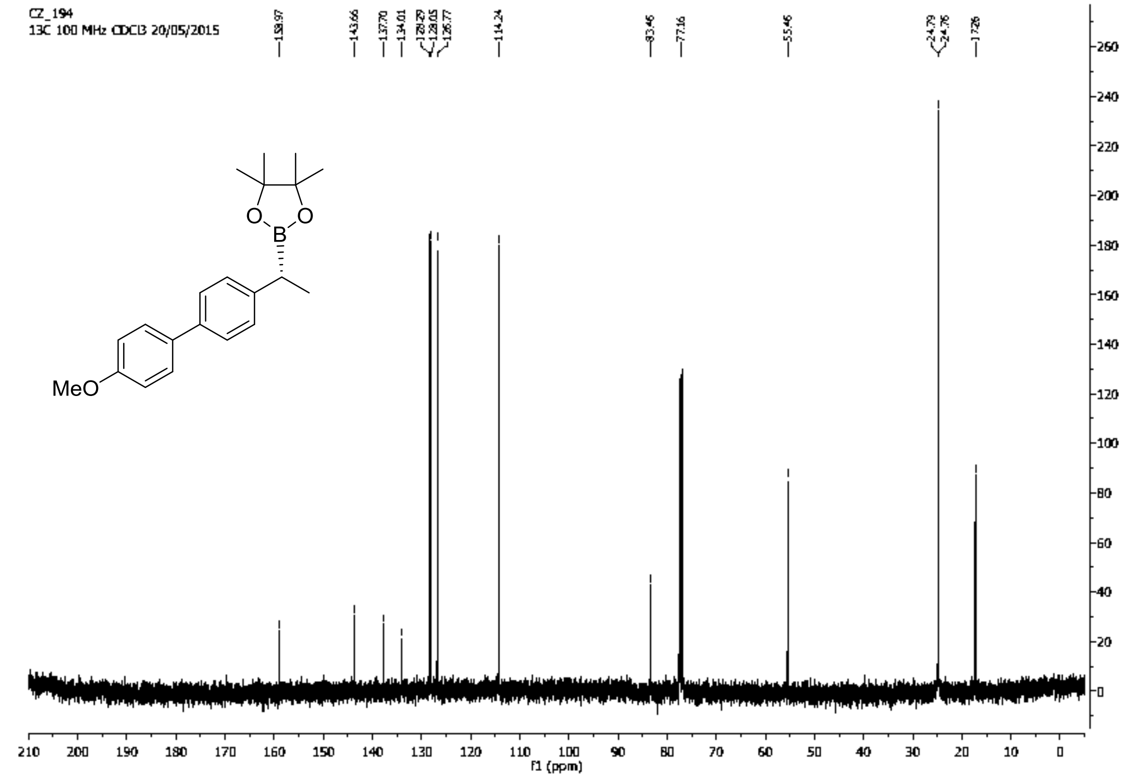

Supplementary Figure 58 | NMR spectra of 9aF. a) ^{1}H NMR spectrum. b) ^{13}C NMR spectrum.

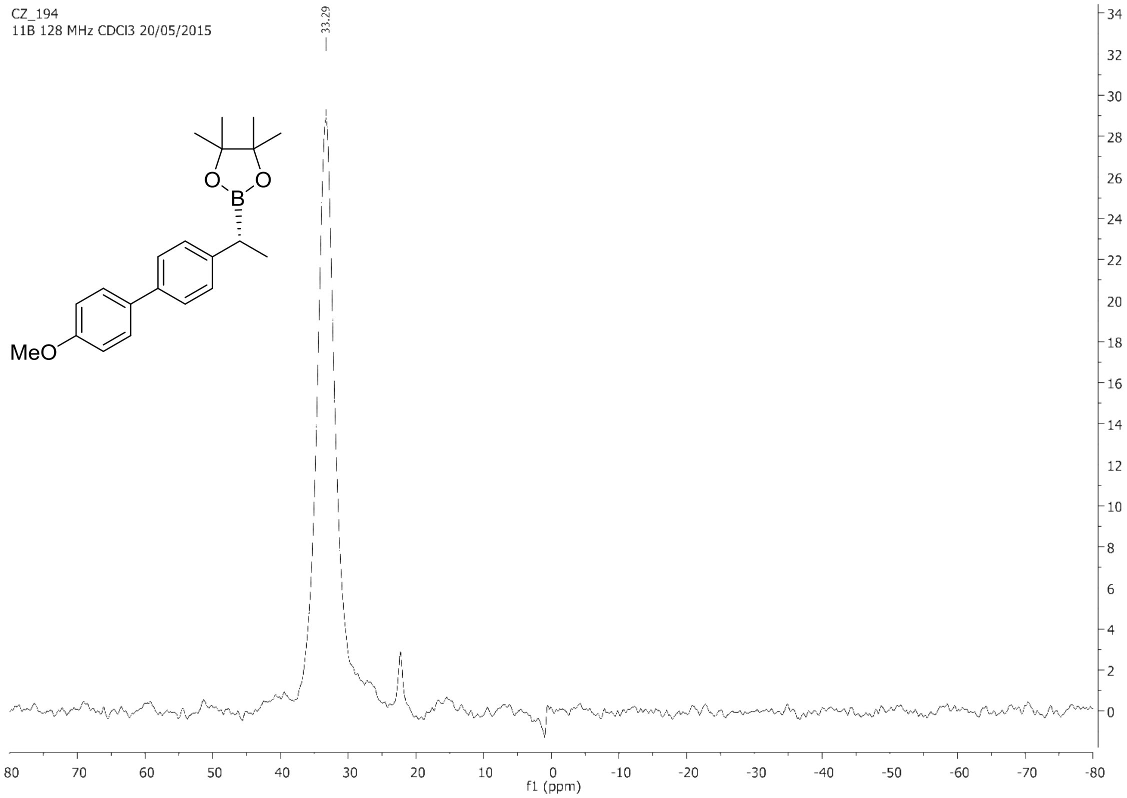

Supplementary Figure 59 | ^{11}B NMR spectrum of 9aF.

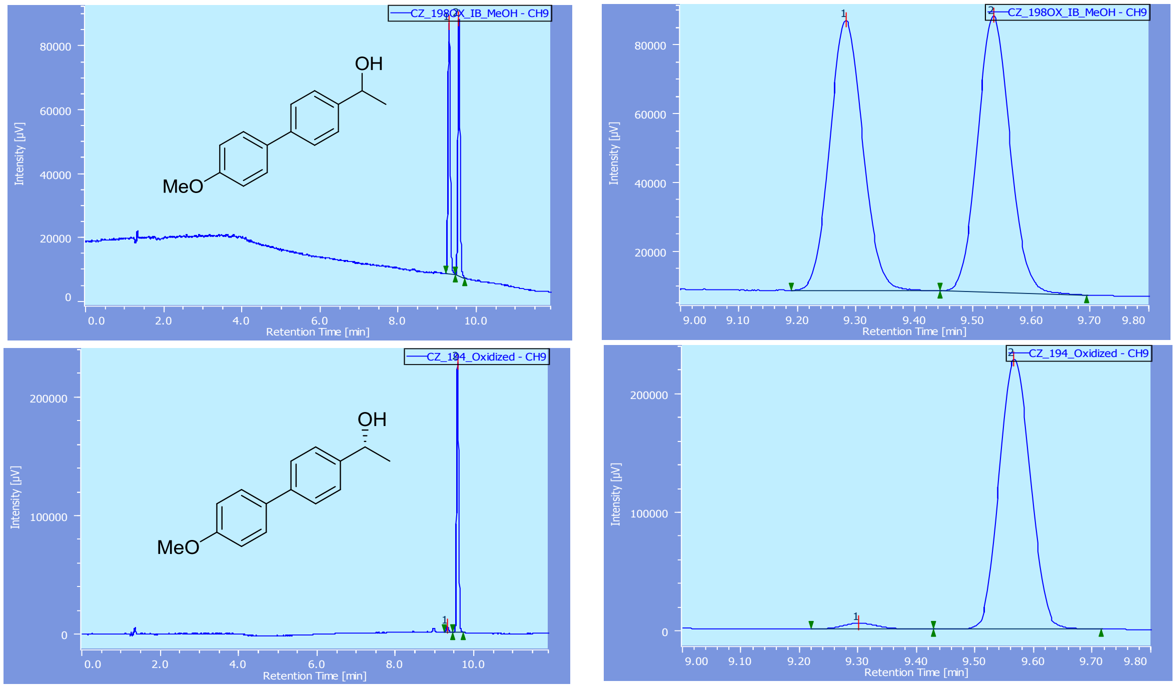

Supplementary Figure 60 | SFC traces of oxidized 9aF.

a)

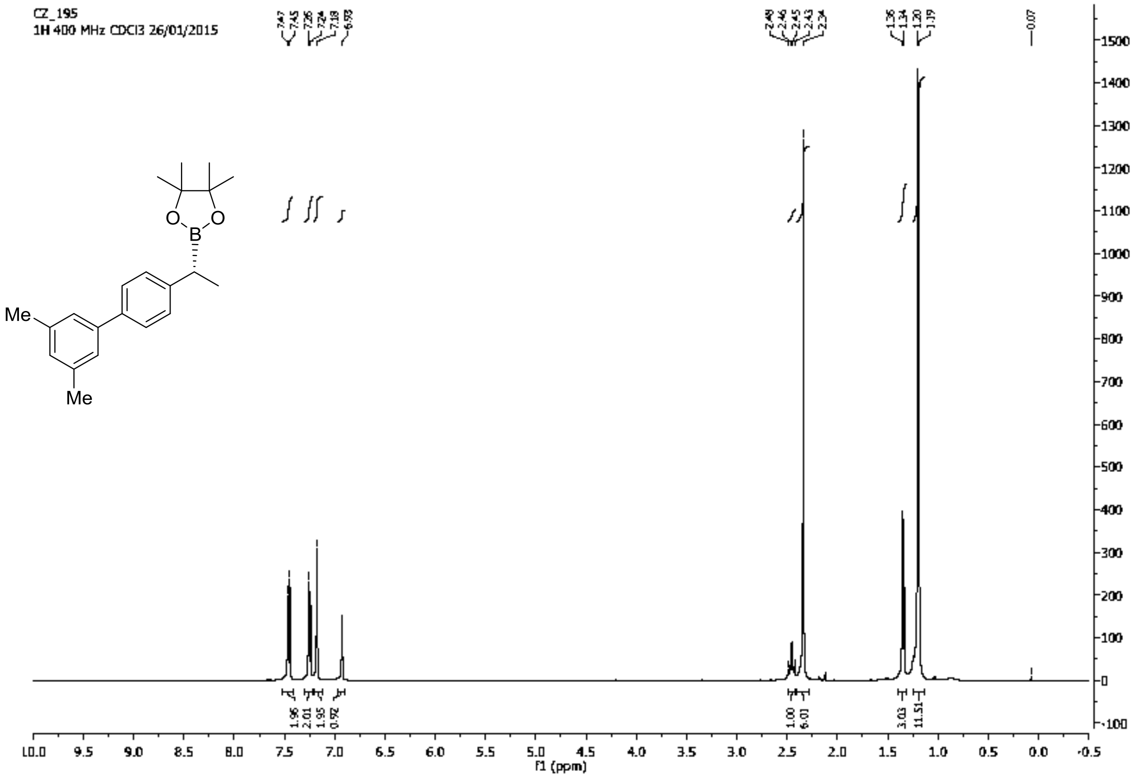

b)

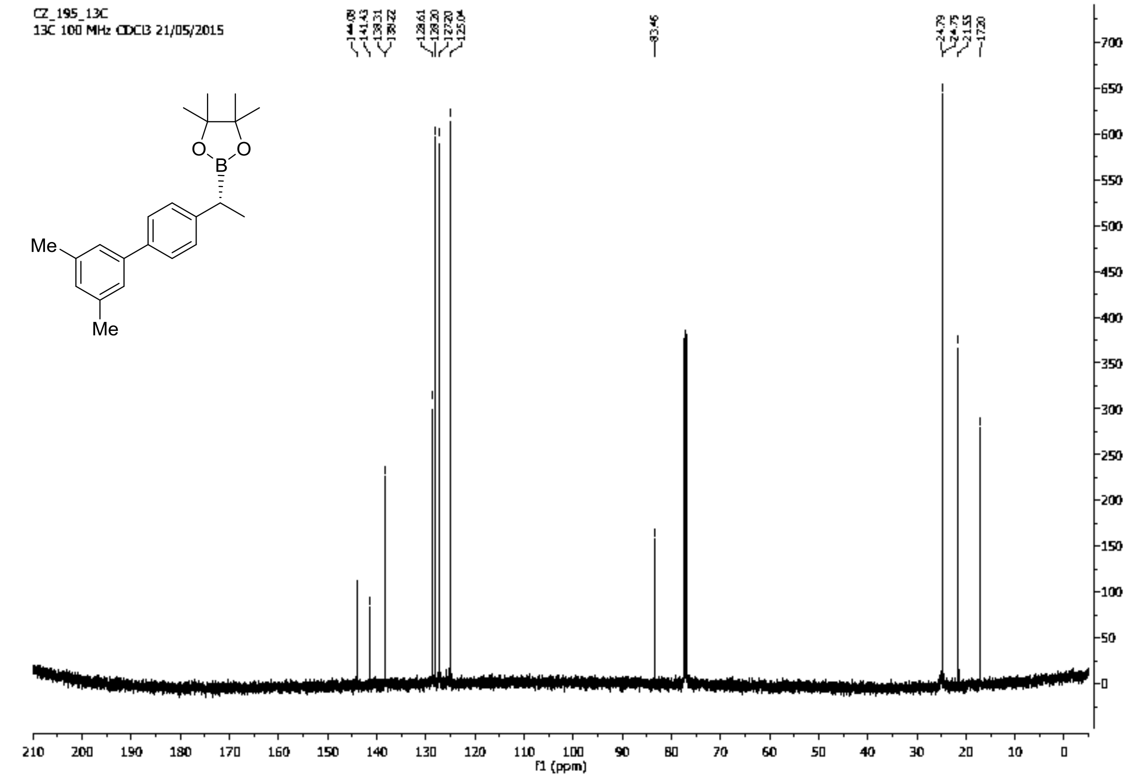

Supplementary Figure 61 | NMR spectra of 9aD. a) ^{1}H NMR spectrum. b) ^{13}C NMR spectrum.

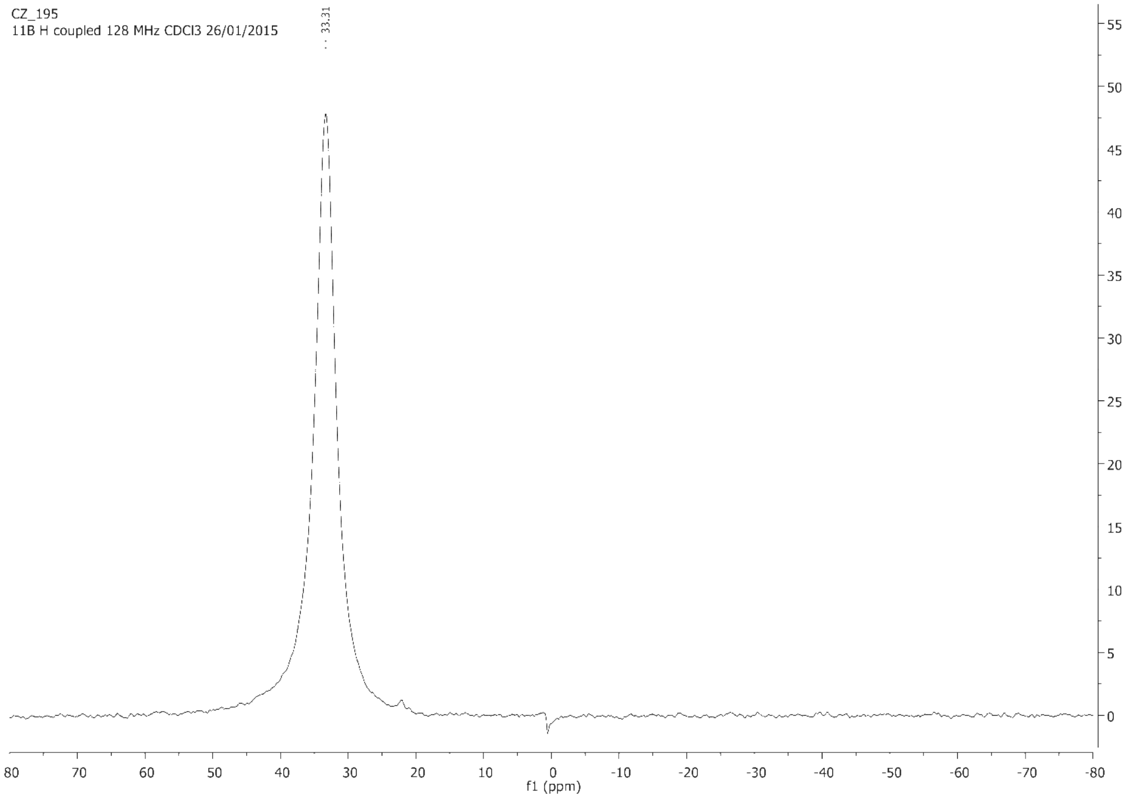

Supplementary Figure 62│ ^{11}B NMR spectrum of 9aD

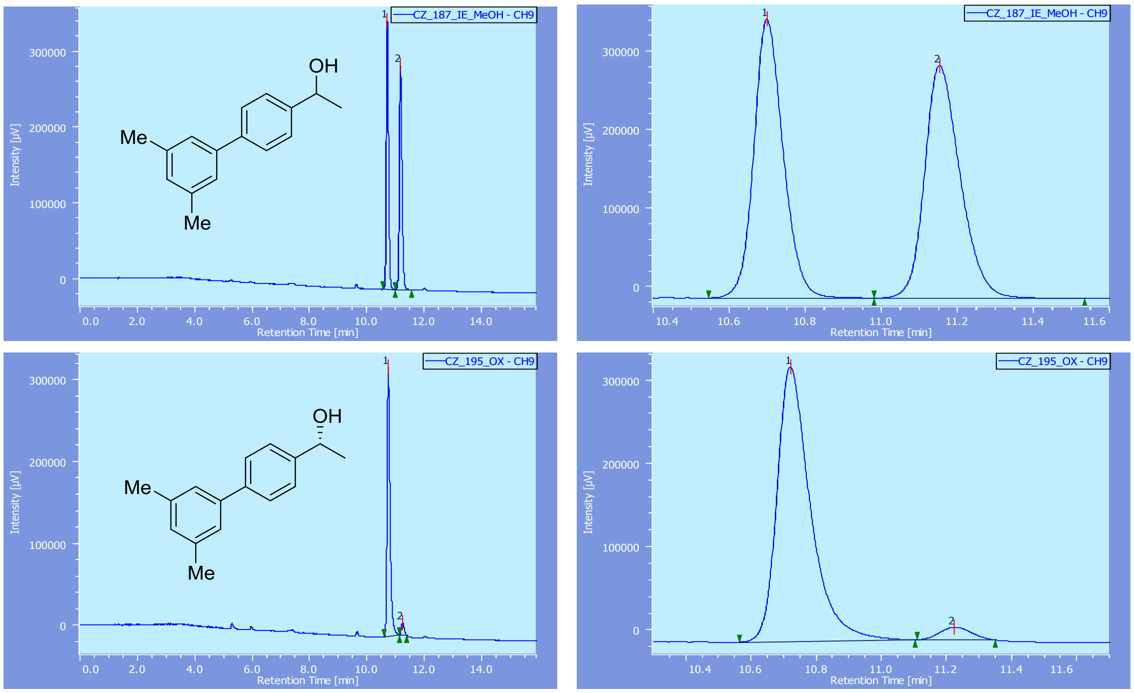

Supplementary Figure 63│SFC traces of oxidized 9aD.

a)

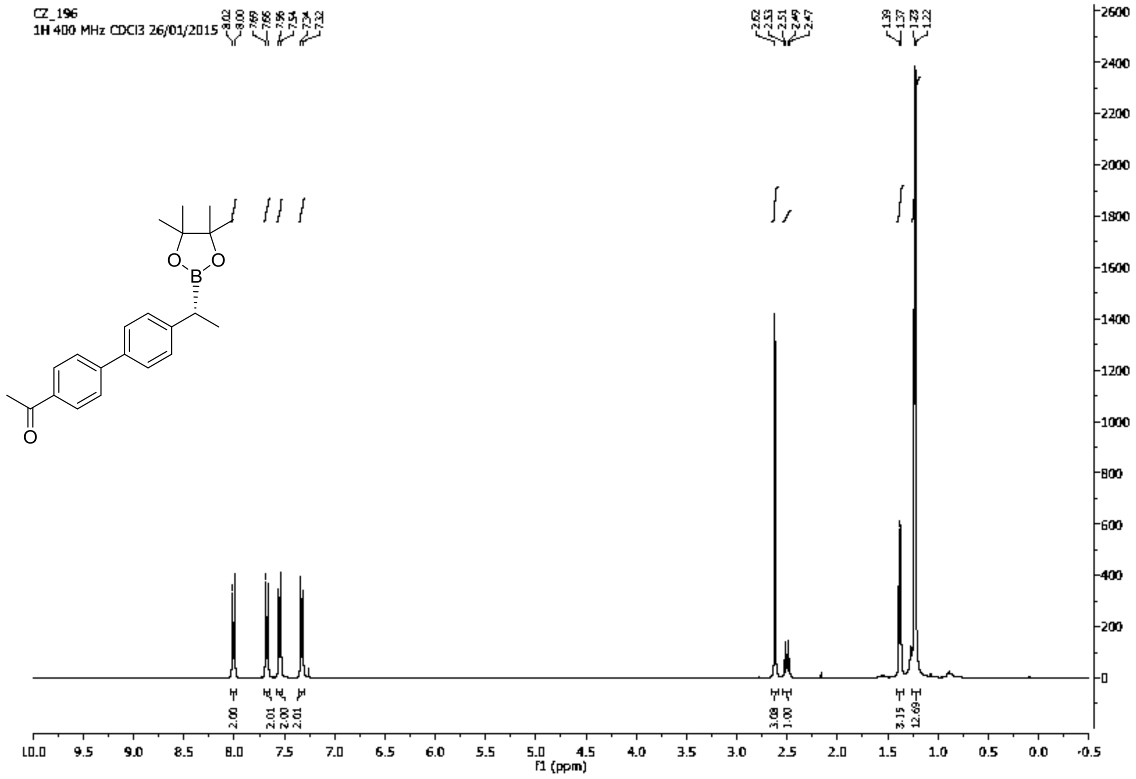

b)

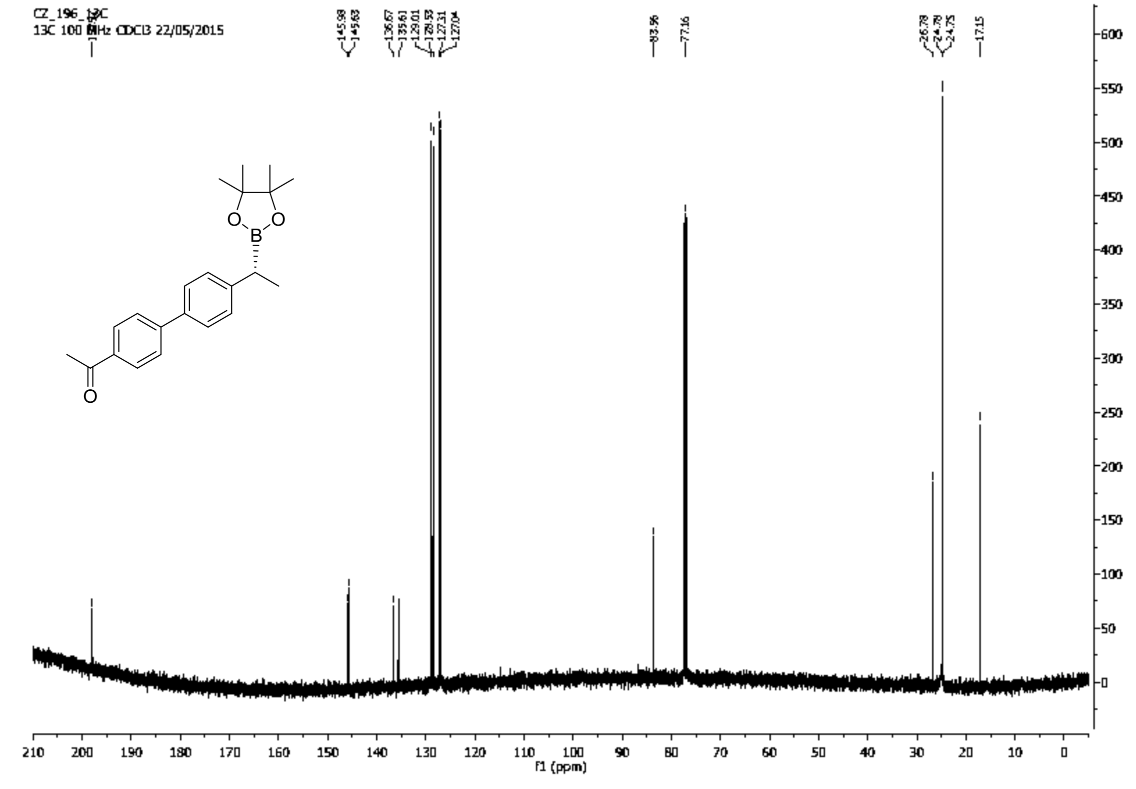

Supplementary Figure 64 | NMR spectra of 9aJ. a) ^{1}H NMR spectrum. b) ^{13}C NMR spectrum.

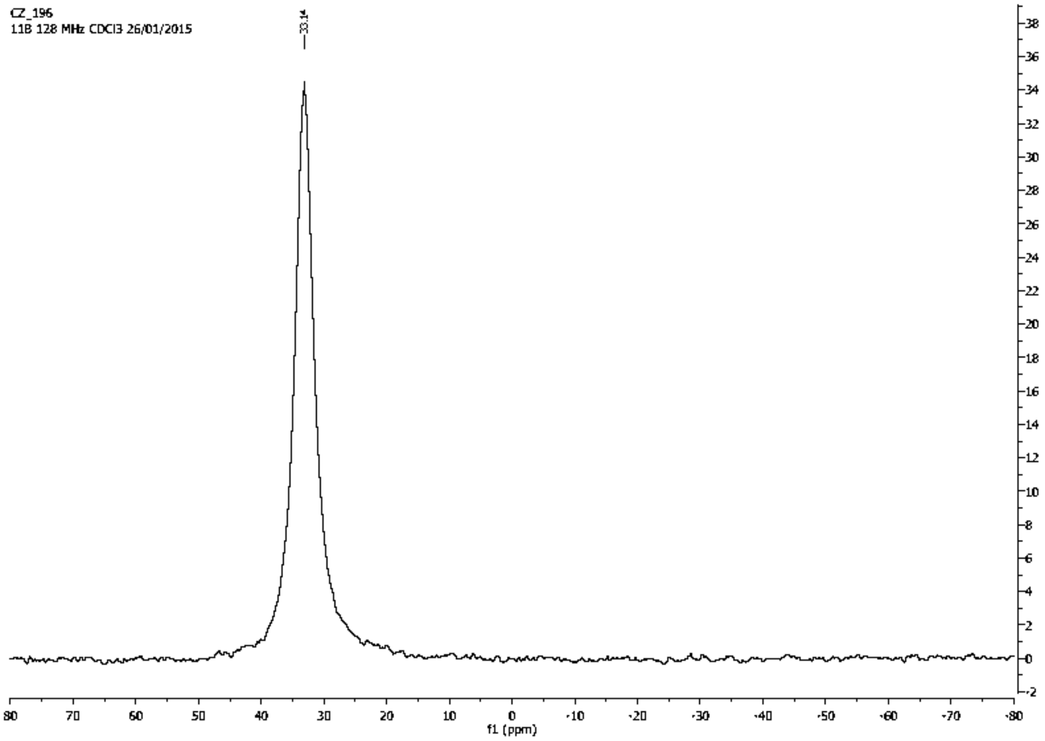

Supplementary Figure 65 | ^{11}B NMR spectrum of 9aJ.

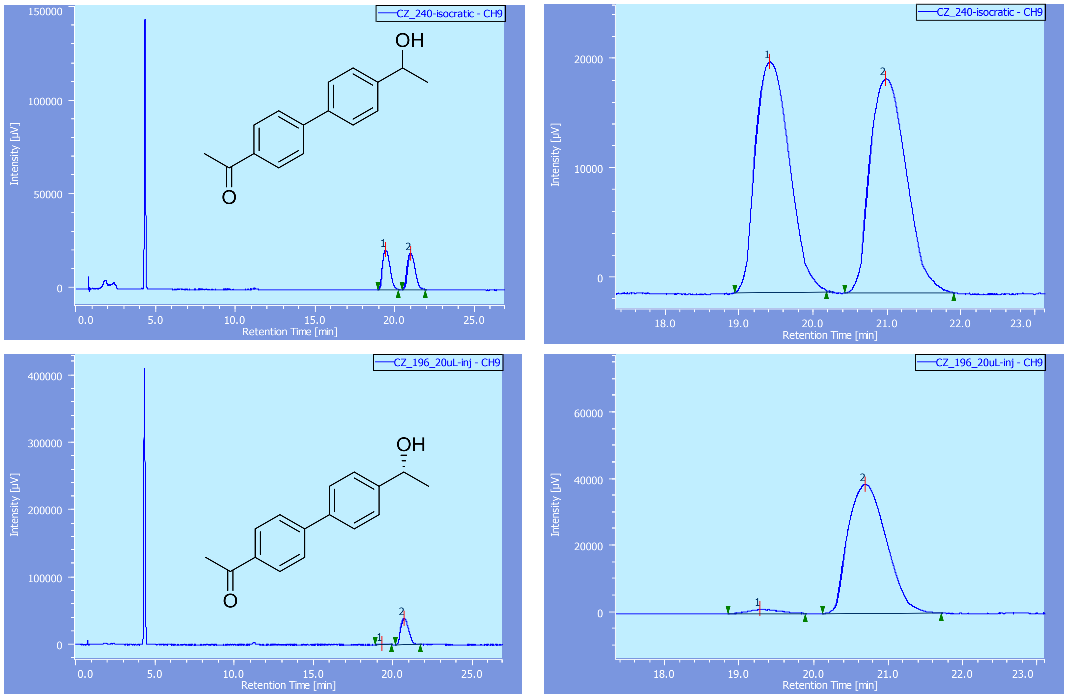

Supplementary Figure 66 | SFC traces of oxidized 9aJ.

a)

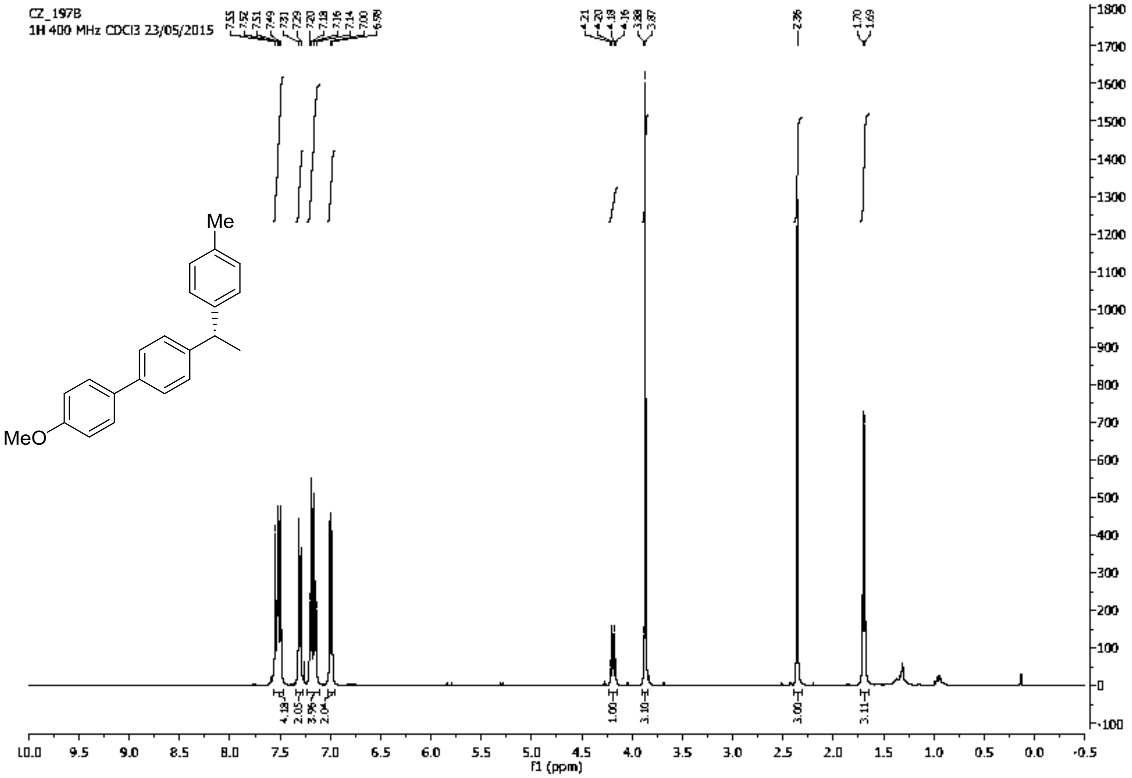

b)

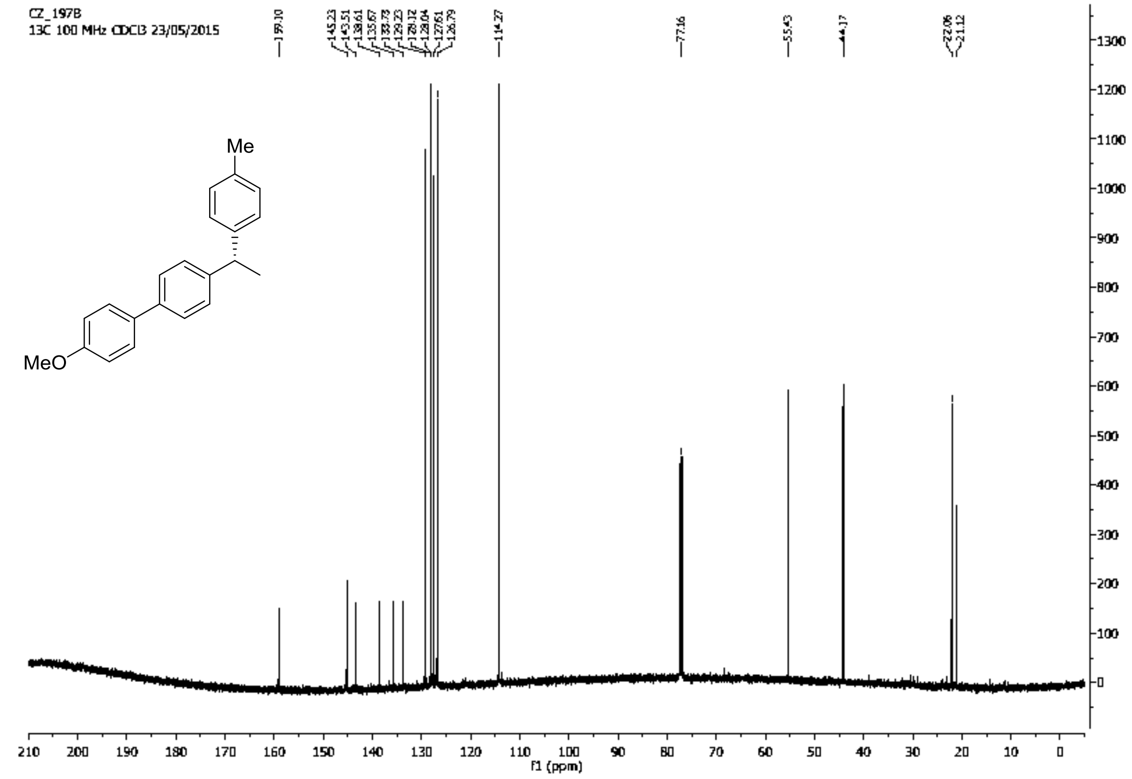

Supplementary Figure 67 | NMR spectra of 10aFb. a) ^{1}H NMR spectrum. b) ^{13}C NMR spectrum.

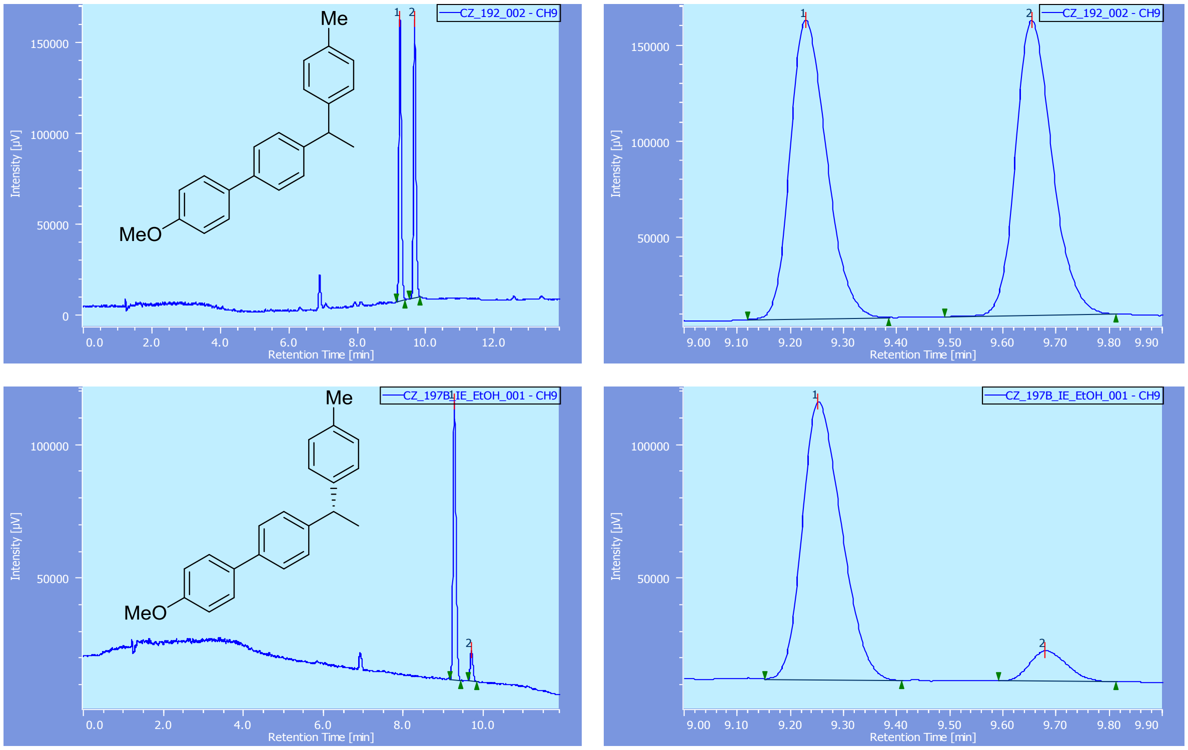

Supplementary Figure 68 | SFC traces of 10aFb.

a)

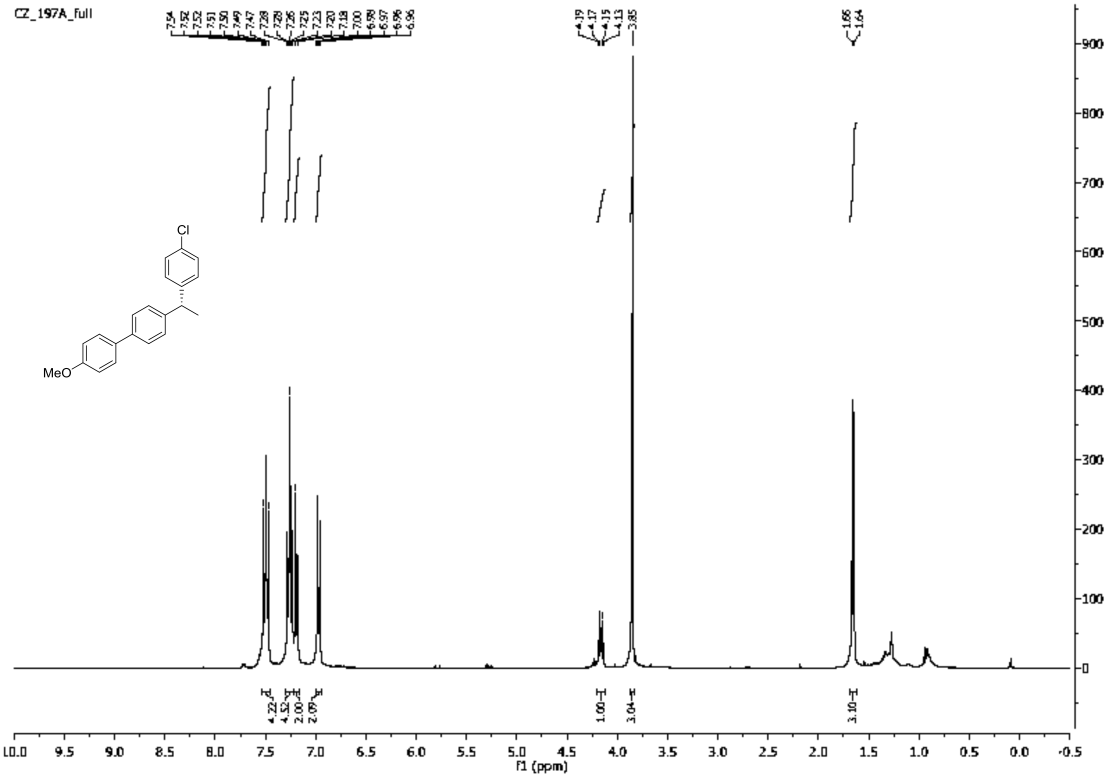

b)

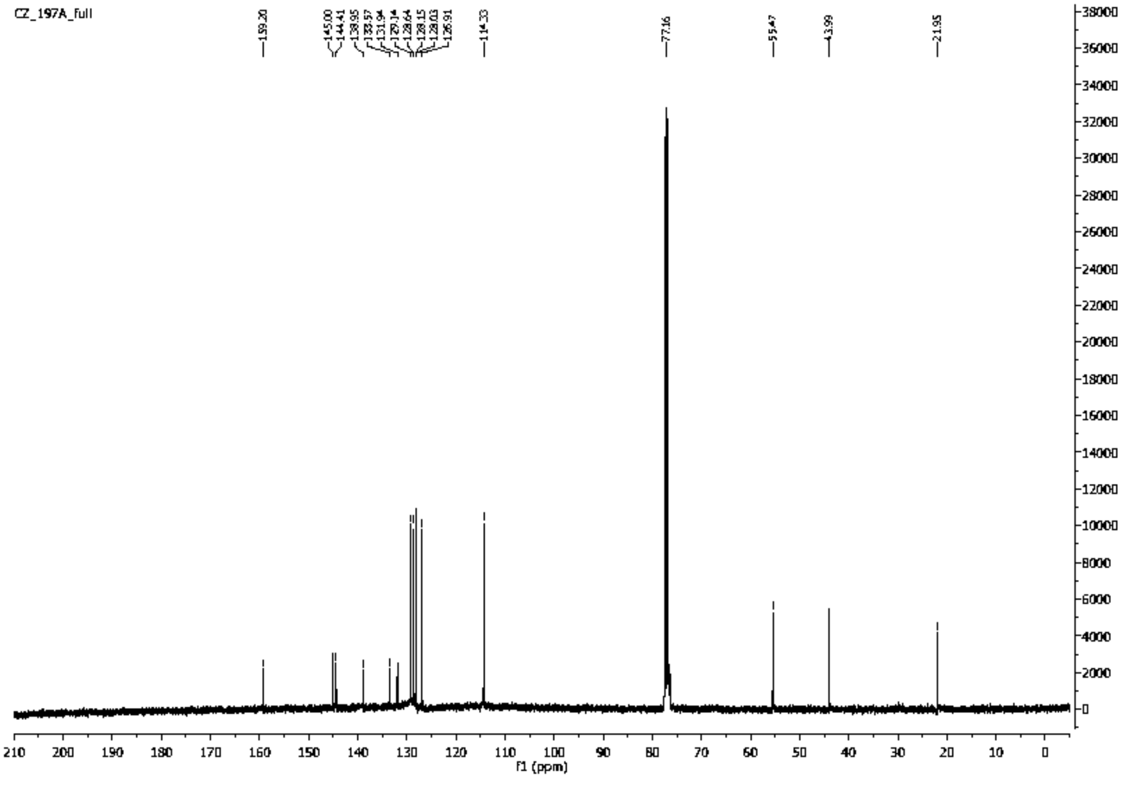

Supplementary Figure 69 | NMR spectra of 10aFl. a) ^{1}H NMR spectrum. b) ^{13}C NMR spectrum.

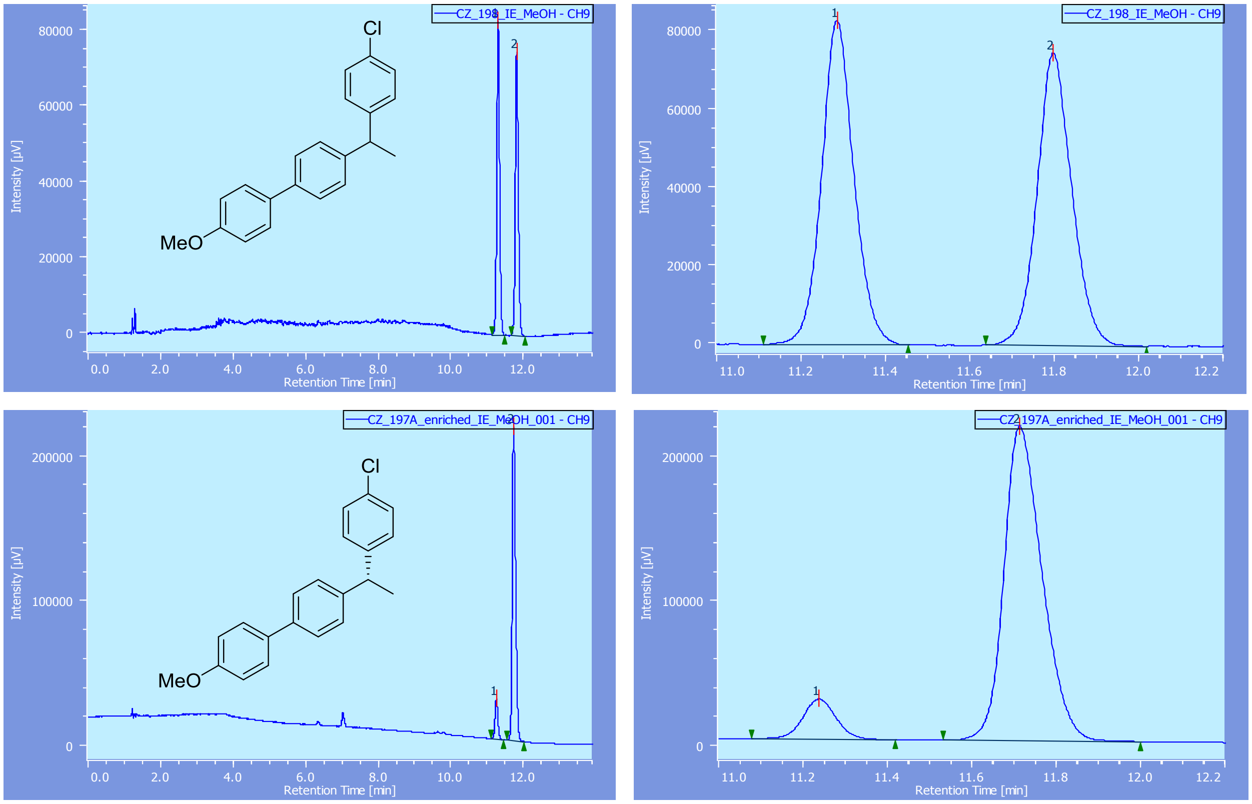

Supplementary Figure 70 | SFC traces of 10aFl.

a)

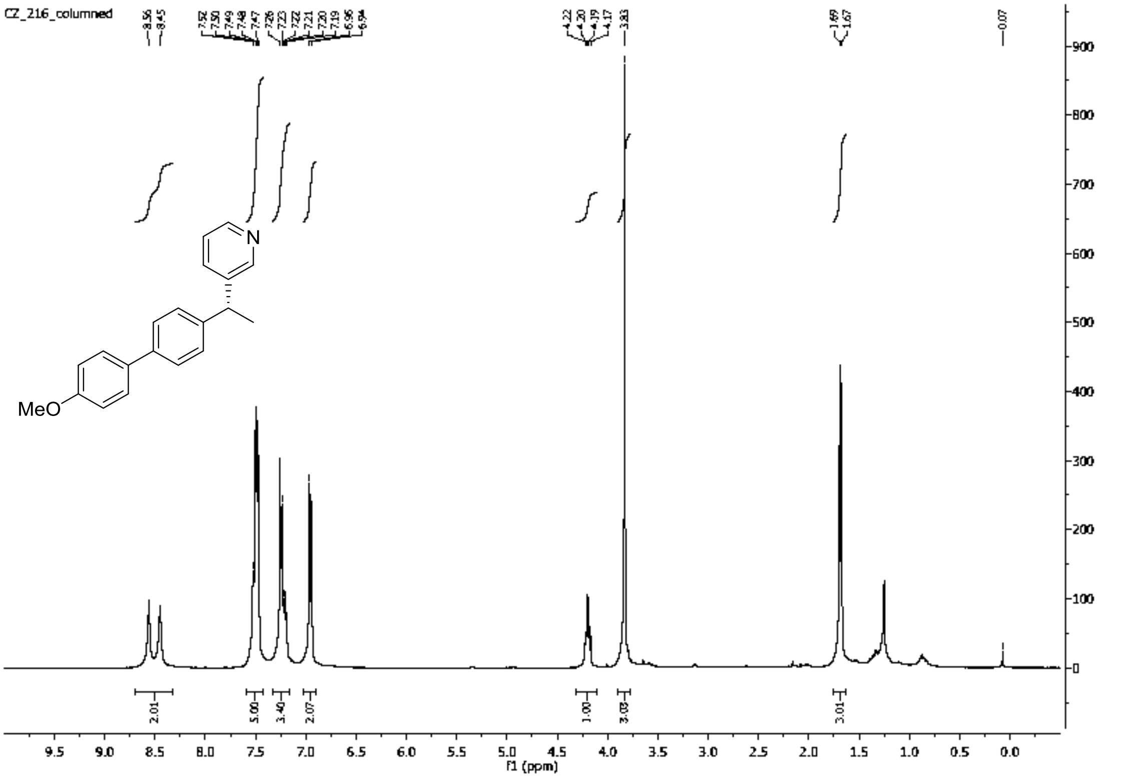

b)

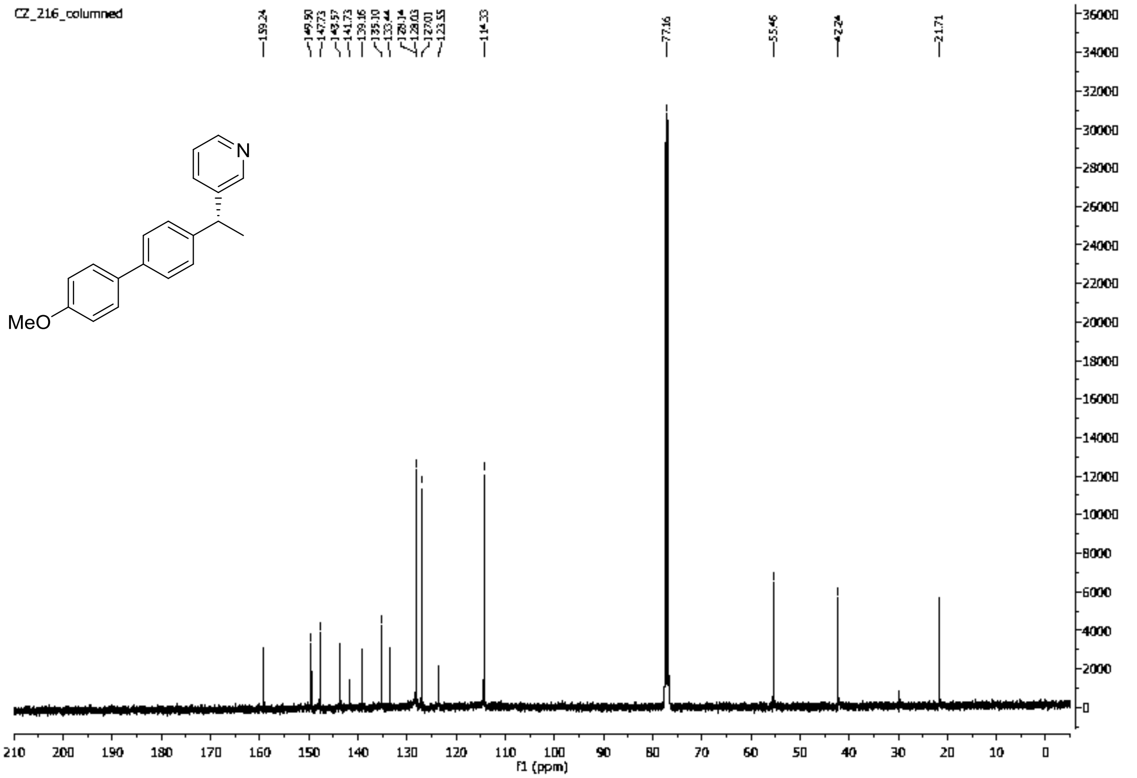

Supplementary Figure 71 | NMR spectra of 10aFi. a) ^{1}H NMR spectrum. b) ^{13}C NMR spectrum.

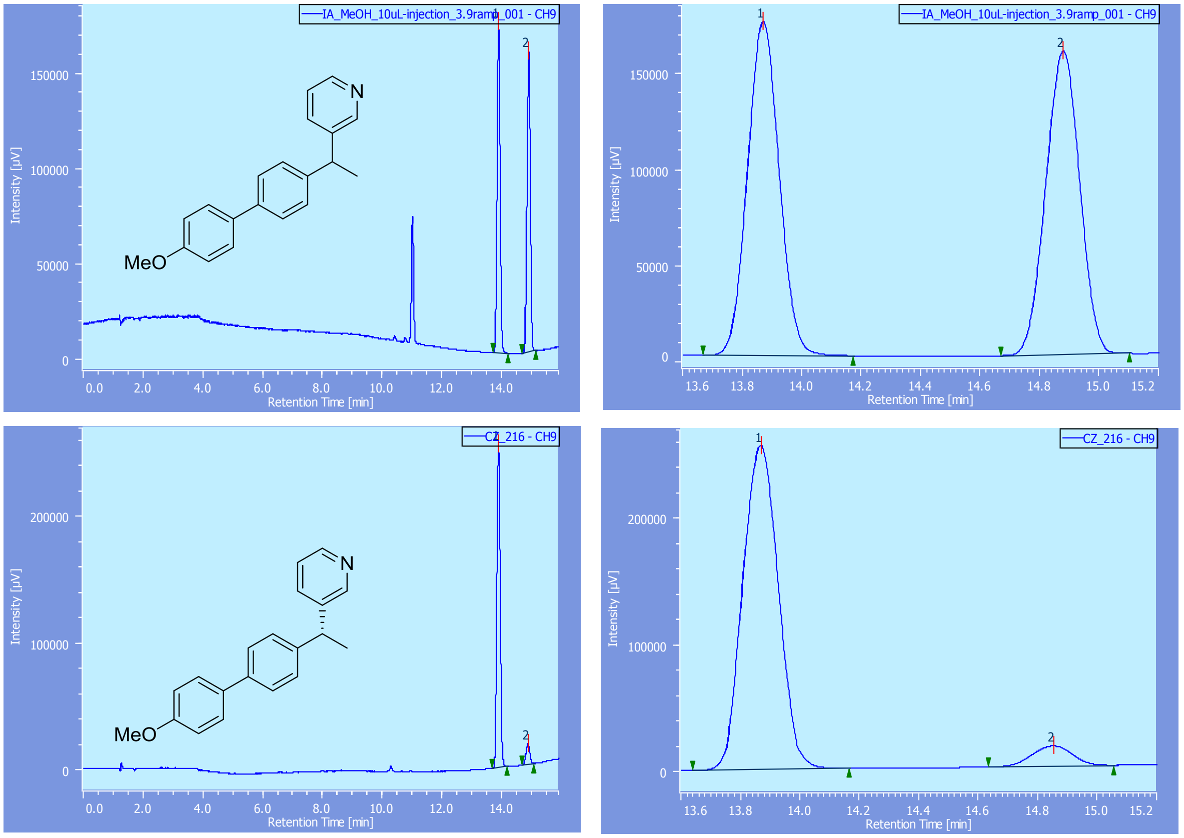

Supplementary Figure 72 | SFC traces of 10aFi.

a)

b)

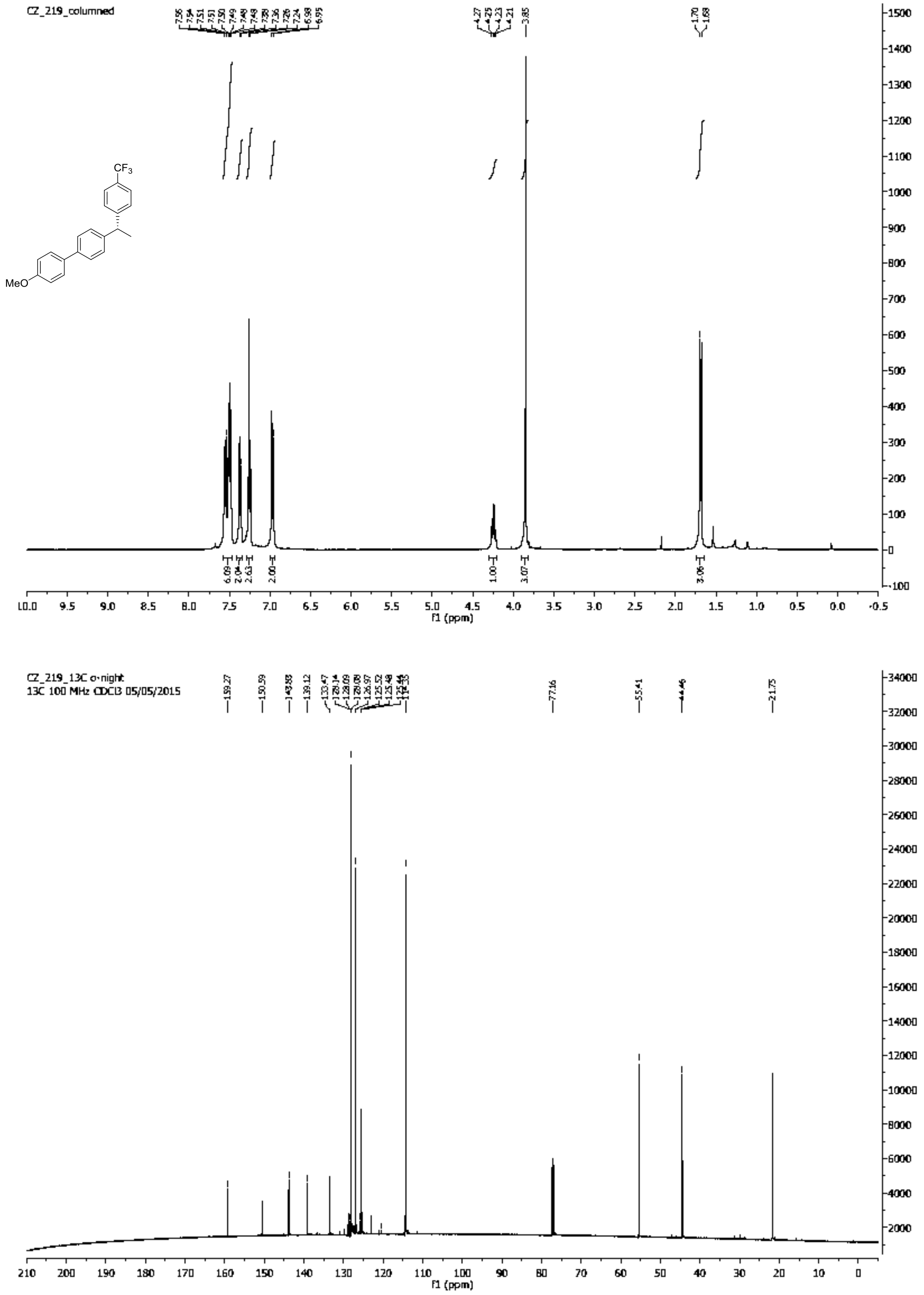

Supplementary Figure 73 | NMR spectra of 10aFk. a) ^{1}H NMR spectrum. b) ^{13}C NMR spectrum.

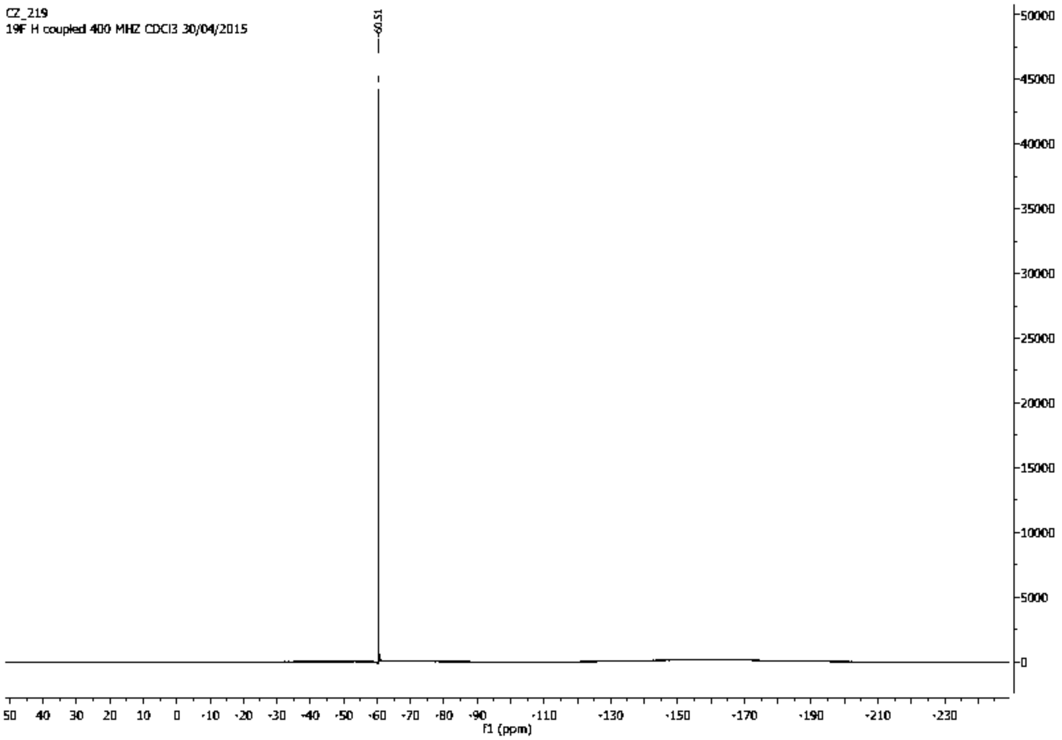

Supplementary Figure 74 | ^{19}F NMR spectrum of 10aFk.

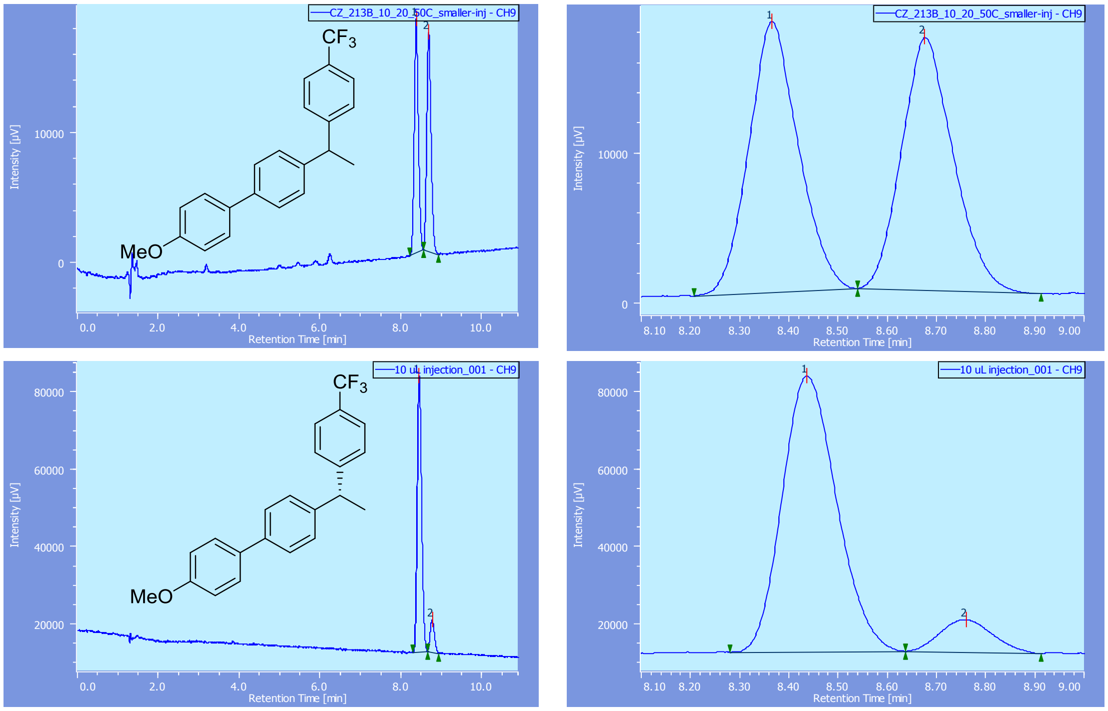

Supplementary Figure 75 | SFC traces of 10aFk.

a)

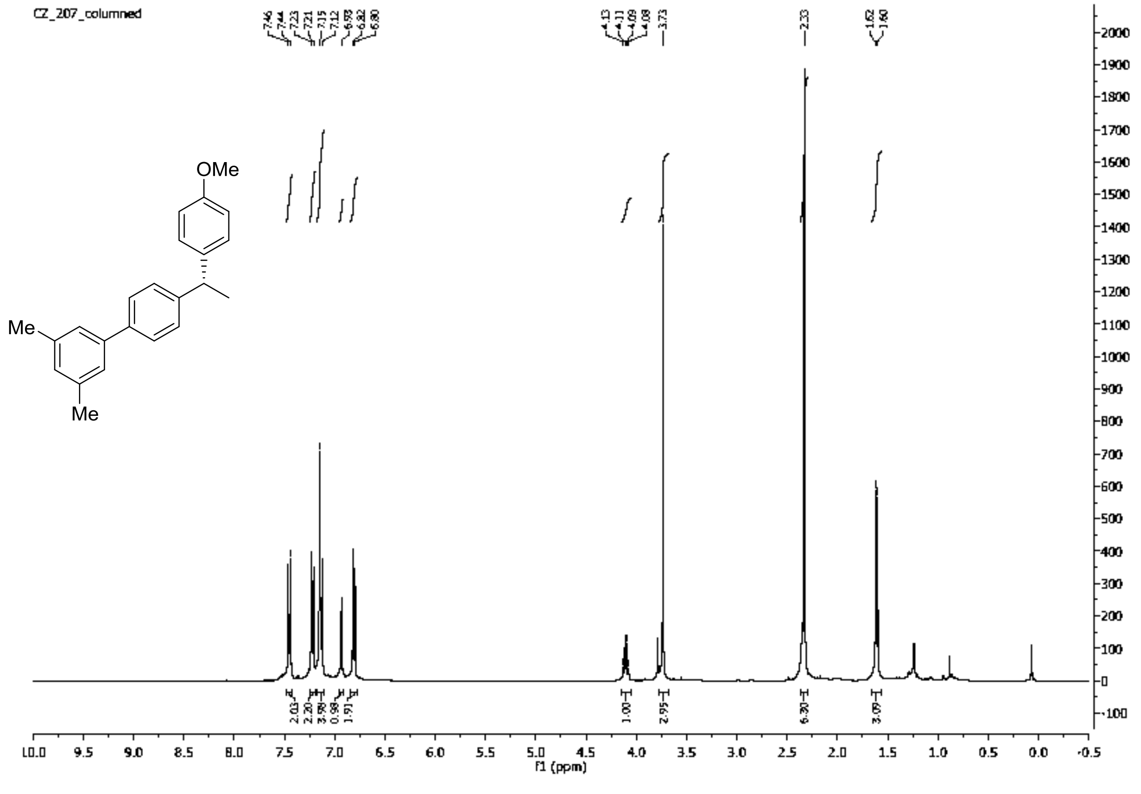

b)

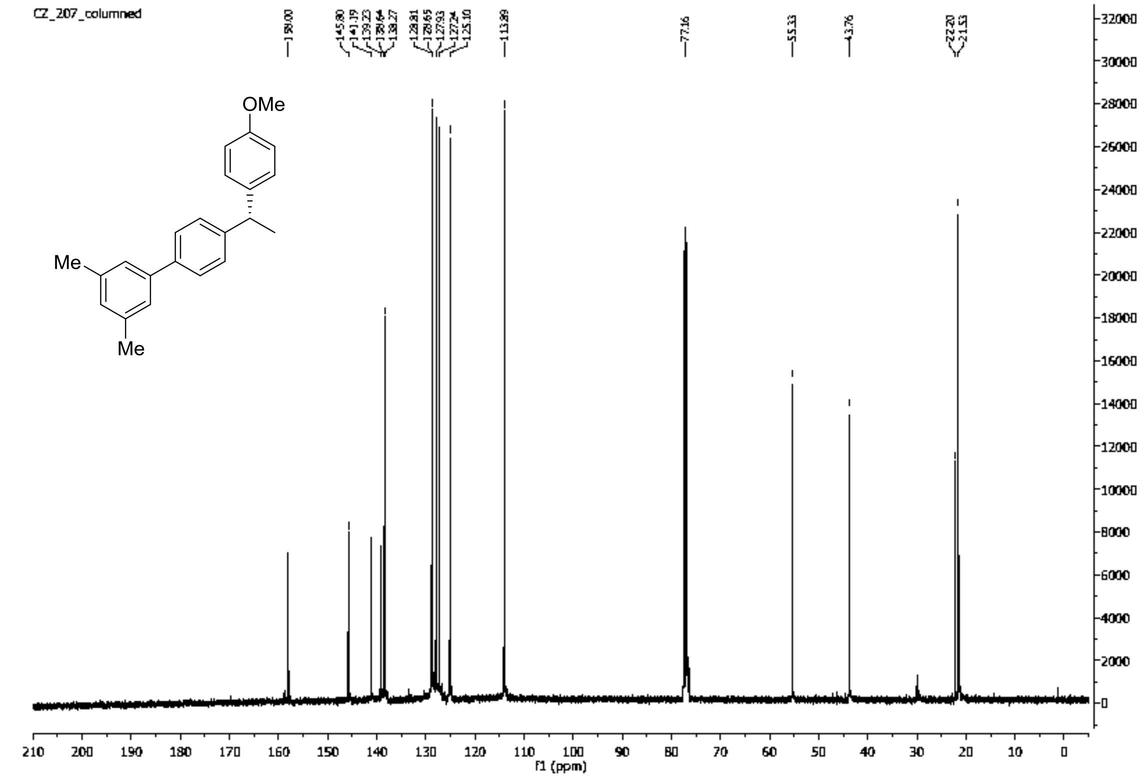

Supplementary Figure 76 | NMR spectra of 10aDf. a) ^{1}H NMR spectrum. b) ^{13}C NMR spectrum.

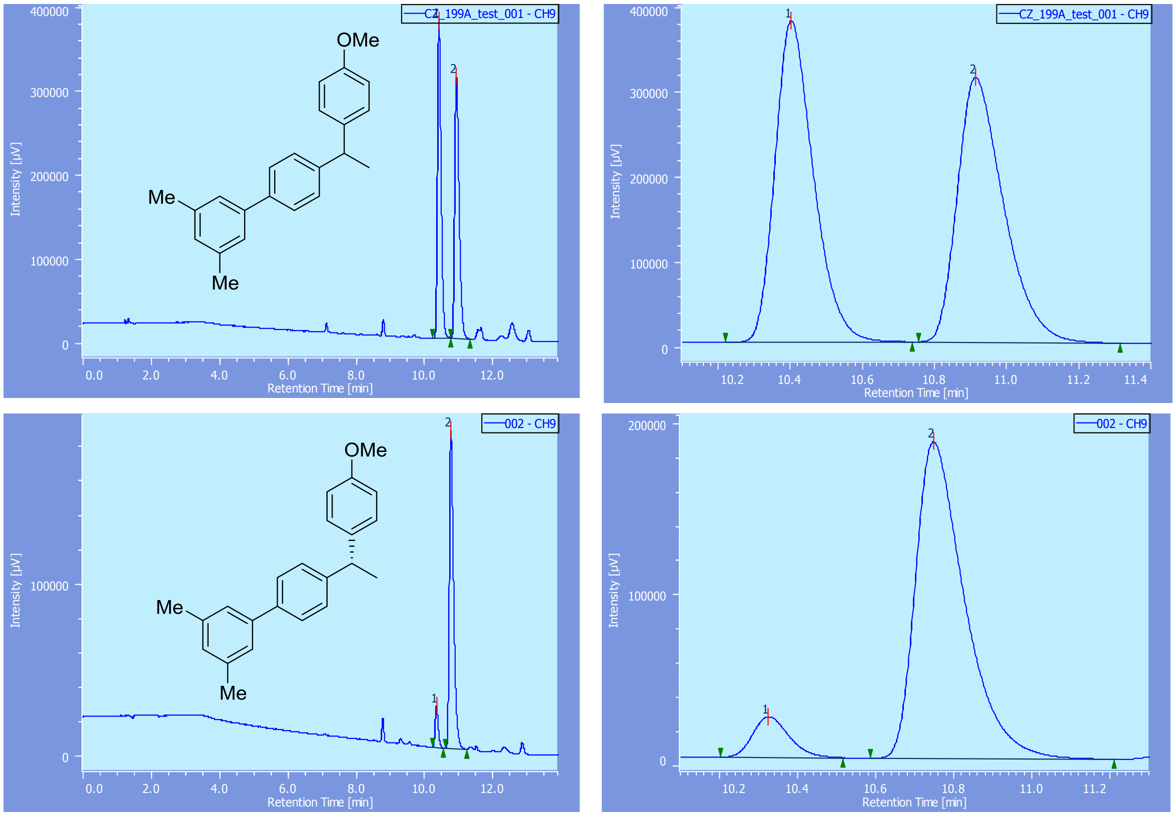

Supplementary Figure 77 | SFC traces of 10aDf.

a)

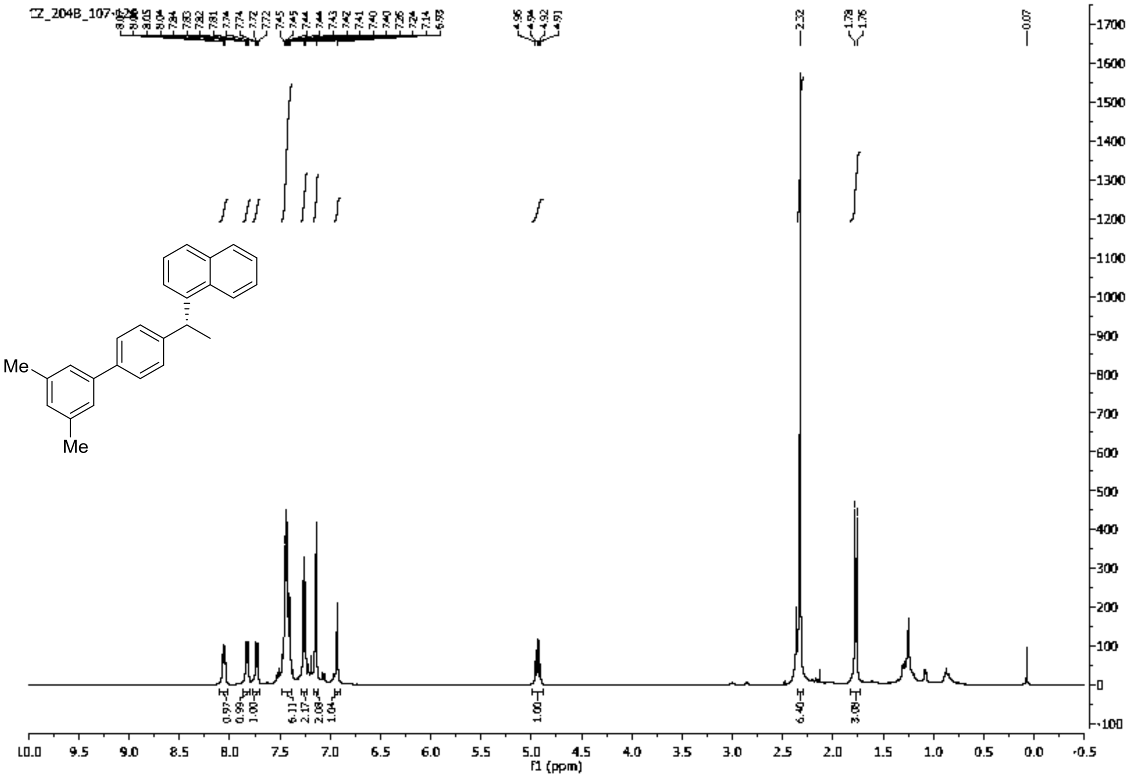

b)

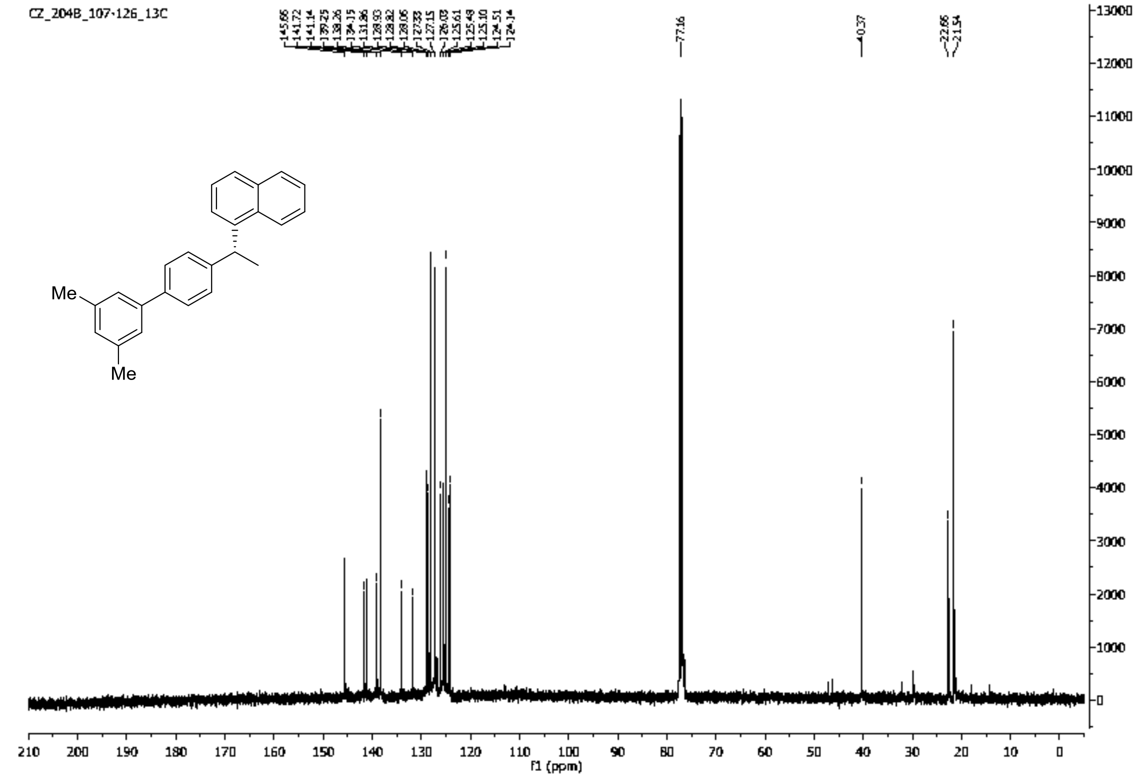

Supplementary Figure 78 | NMR spectra of 10aDm. a) ^{1}H NMR spectrum. b) ^{13}C NMR spectrum.

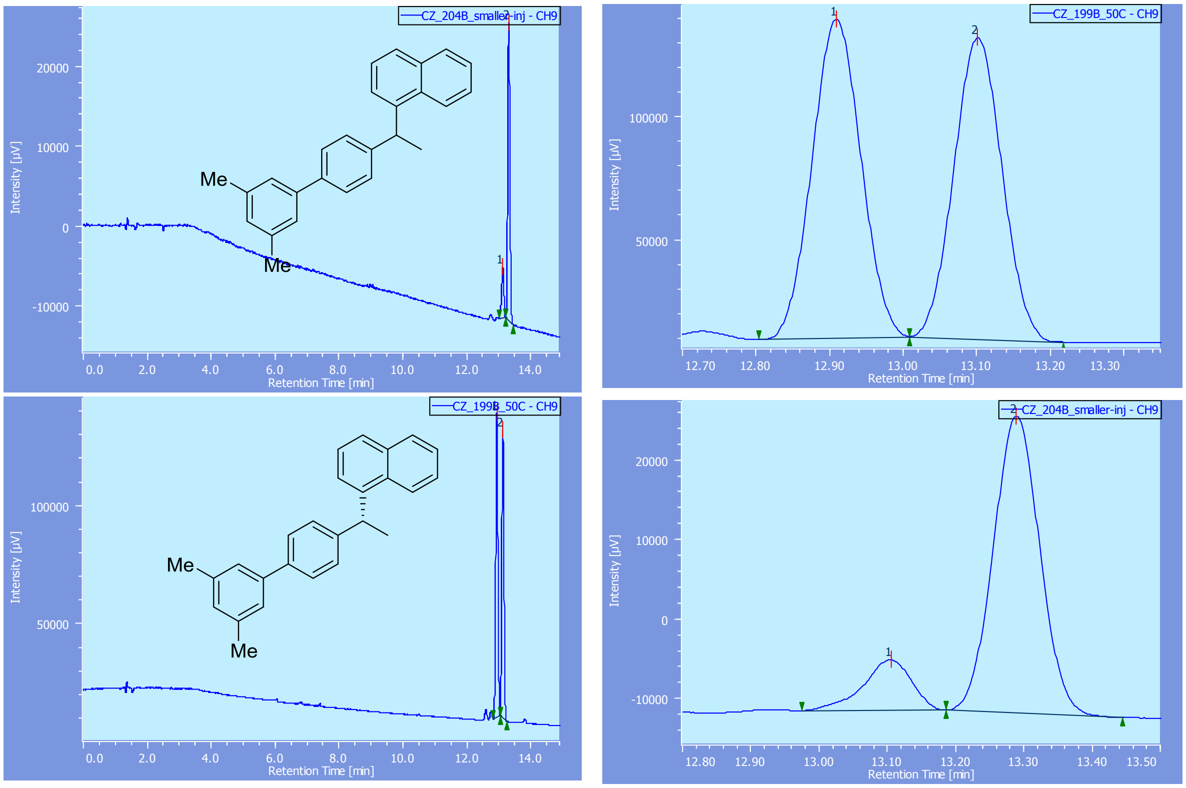

Supplementary Figure 79 | SFC traces of 10aDm.

a)

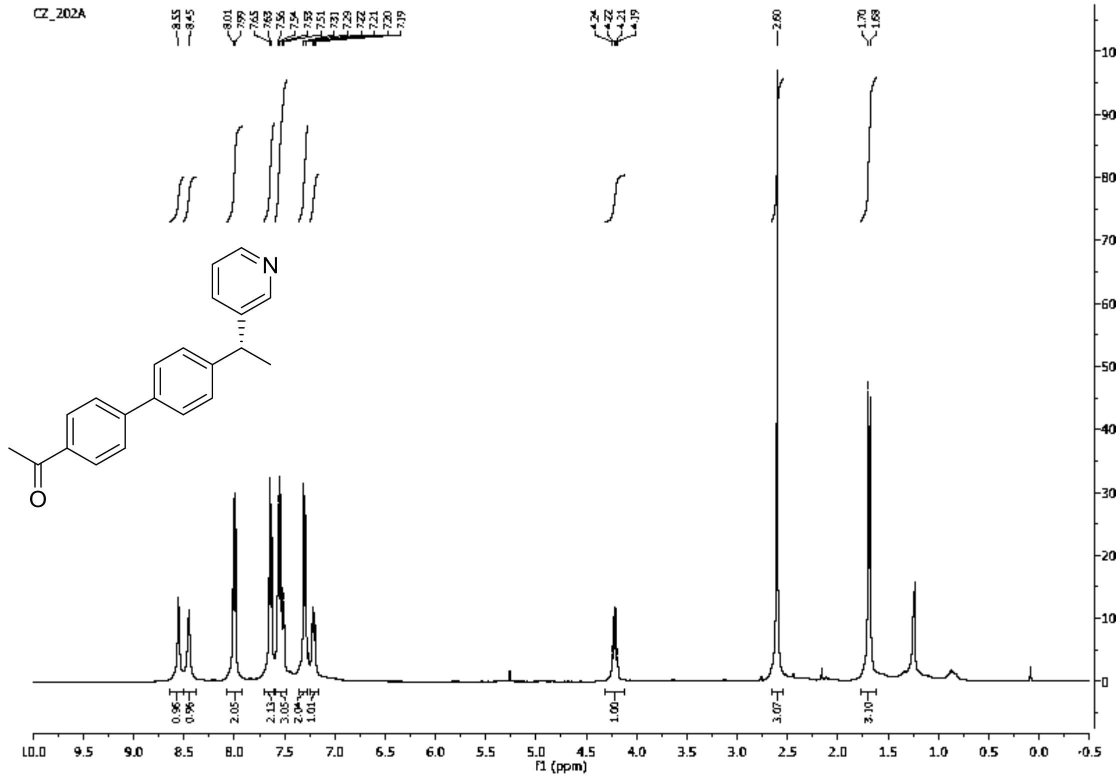

b)

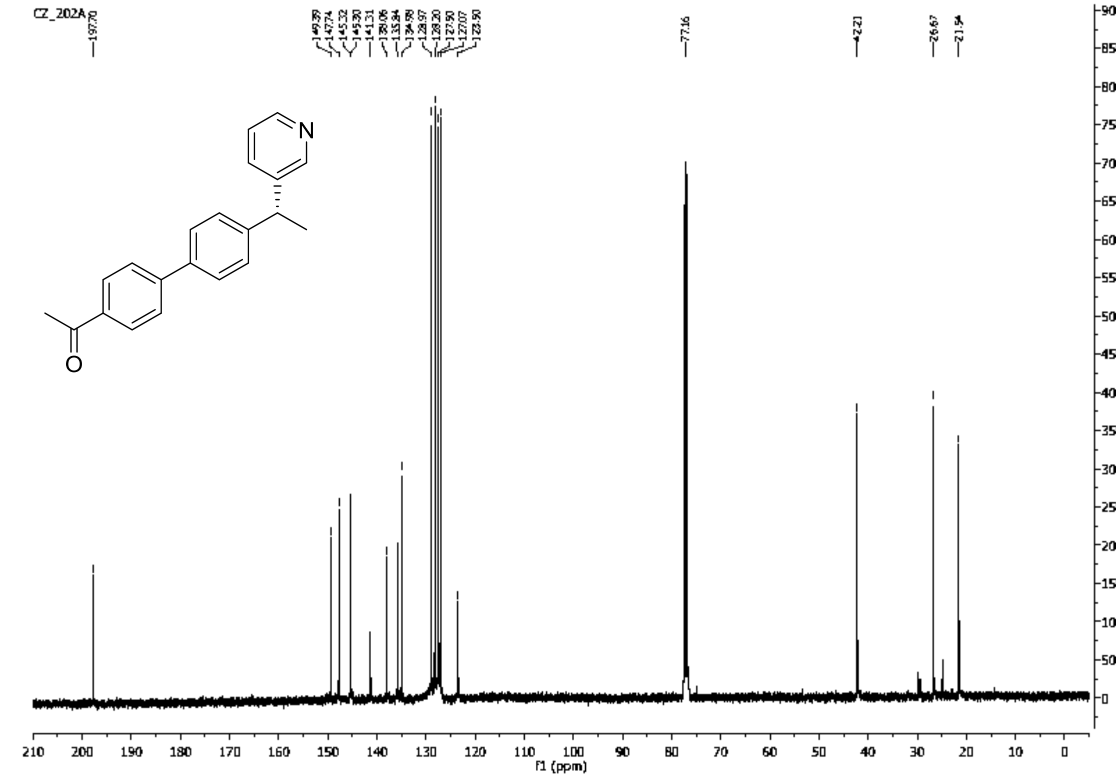

Supplementary Figure 80 | NMR spectra of 10aJi. a) ^{1}H NMR spectrum. b) ^{13}C NMR spectrum.

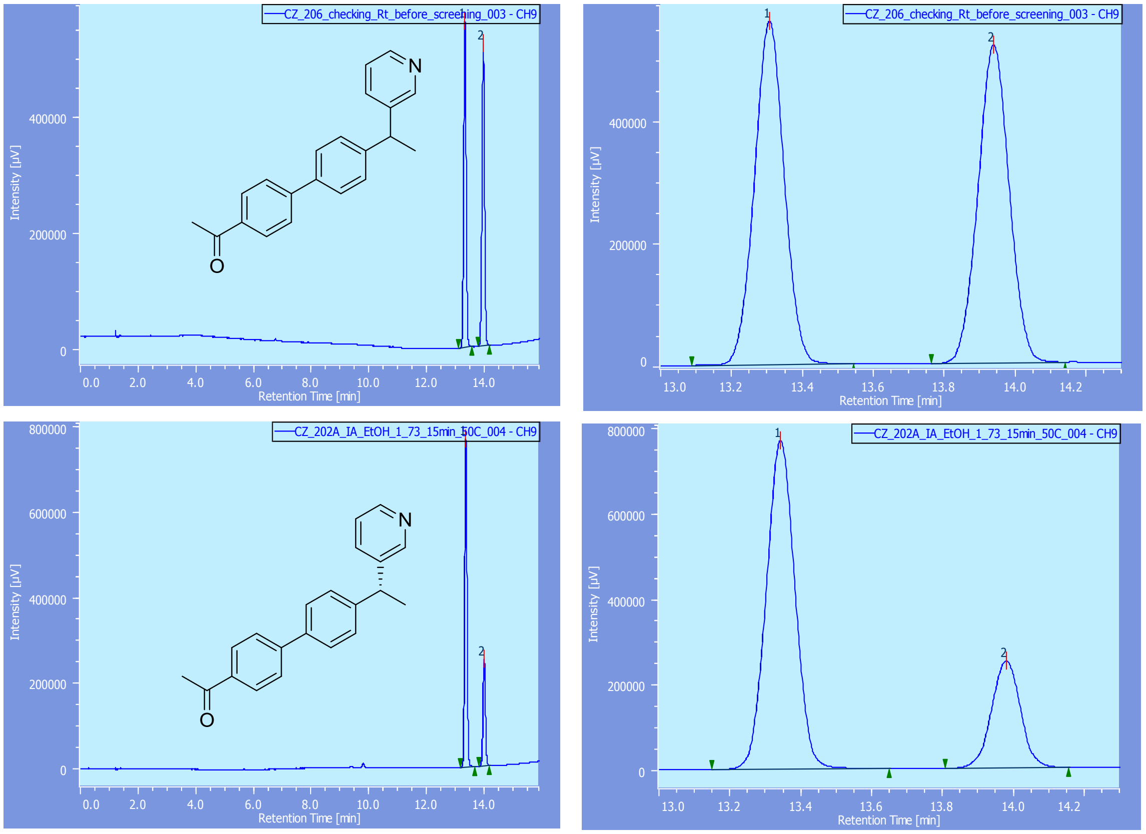

Supplementary Figure 81 | SFC traces of 10aJi.

a)

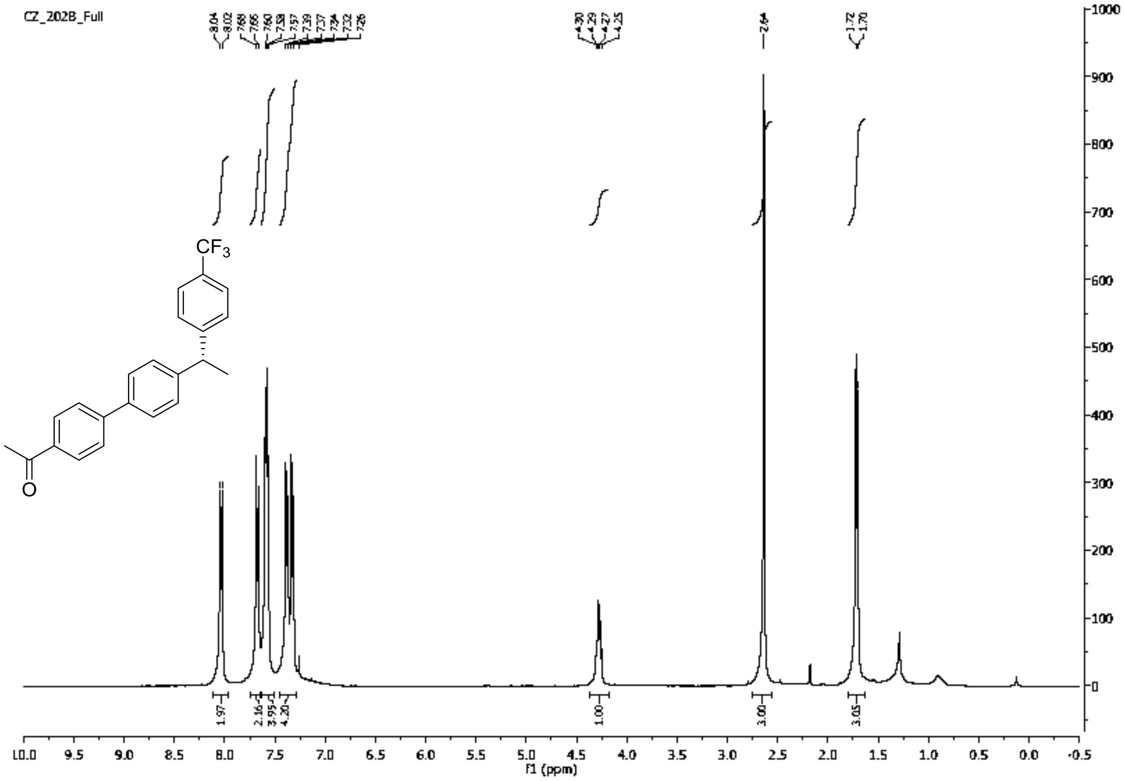

b)

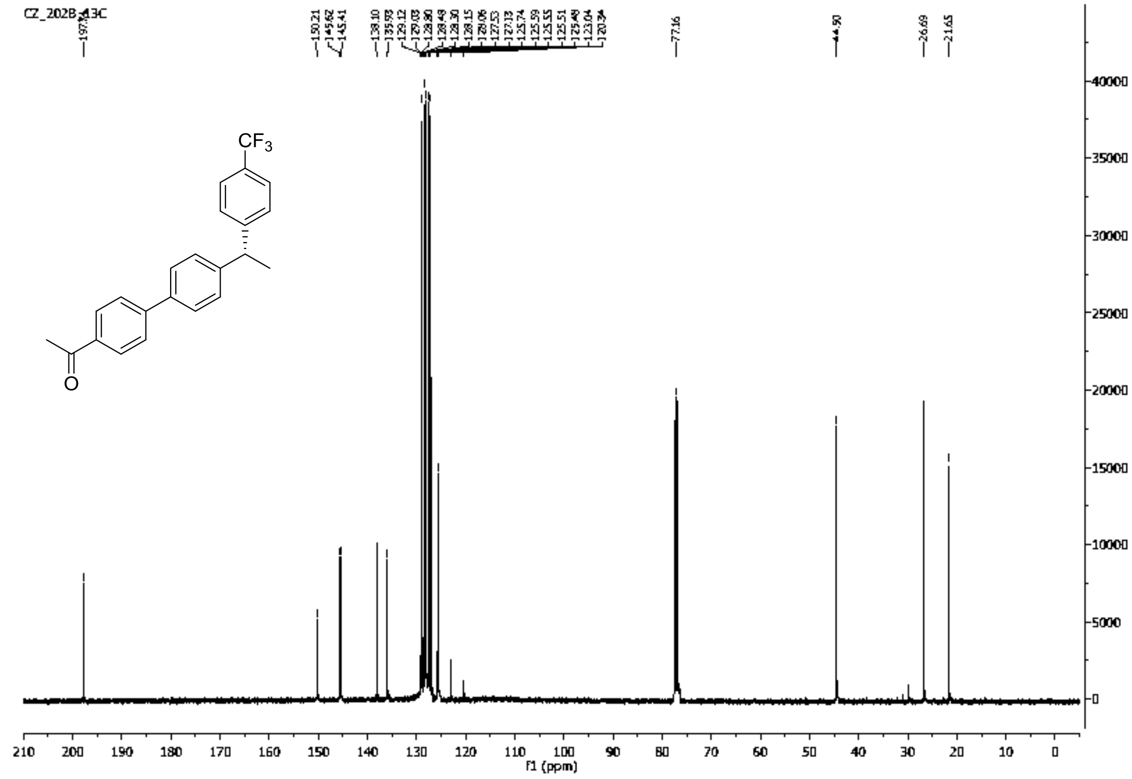

Supplementary Figure 82 | NMR spectra of 10aJk. a) ^{1}H NMR spectrum. b) ^{13}C NMR spectrum.

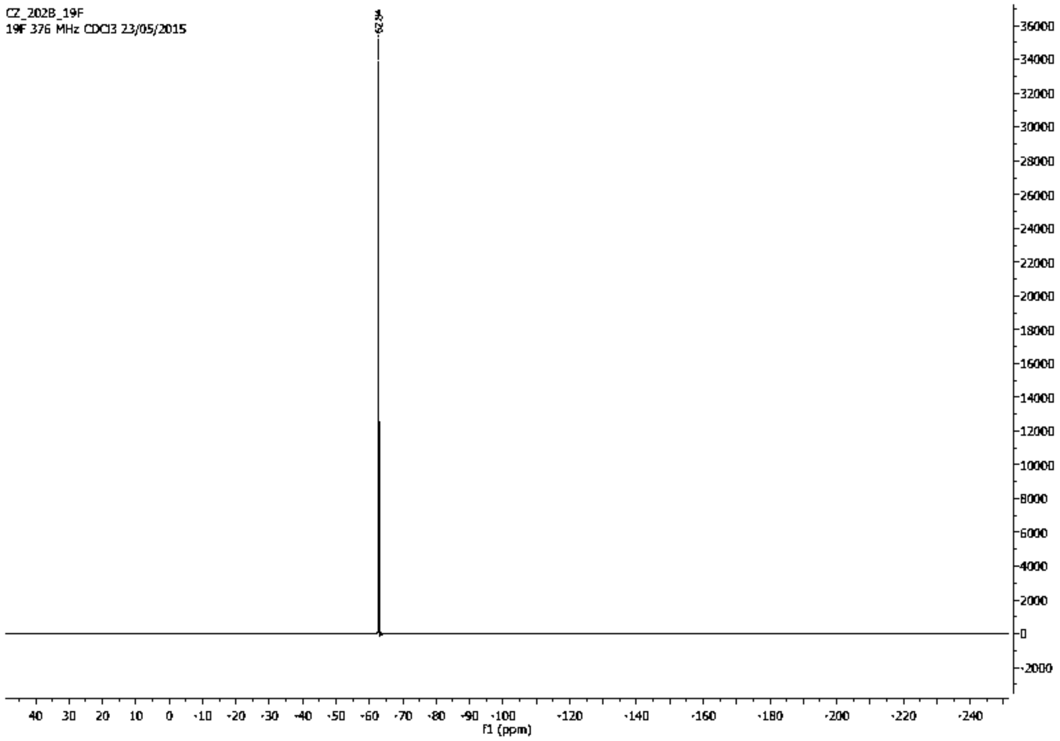

Supplementary Figure 83 | ^{19}F NMR spectrum of 10aJk

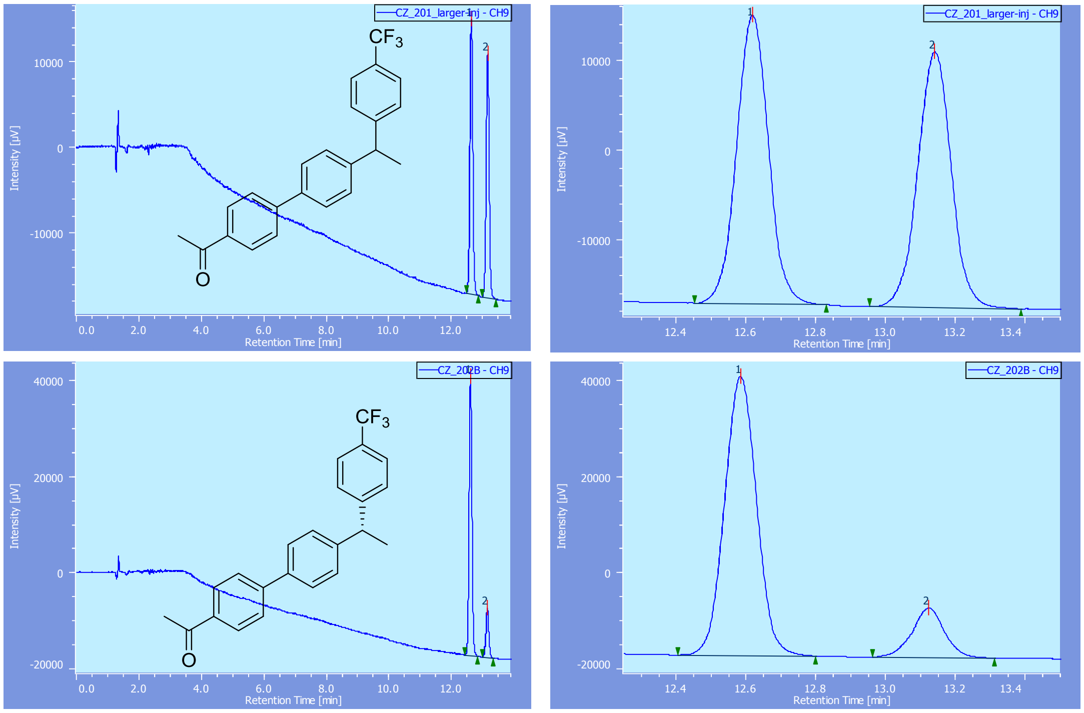

Supplementary Figure 84 | SFC traces of 10aJk.

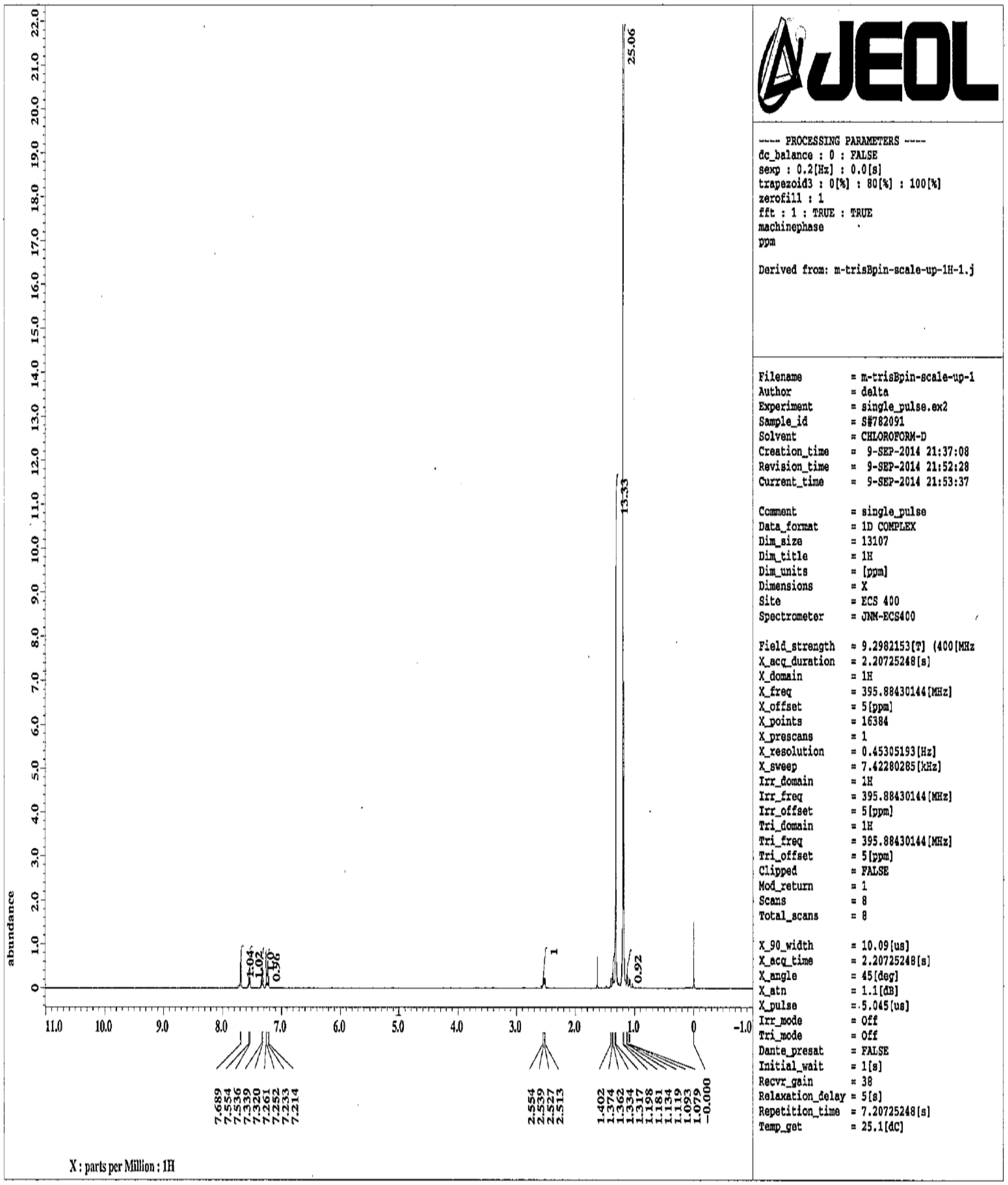

Supplementary Figure 85 | ^{1}H NMR spectrum of 11a.

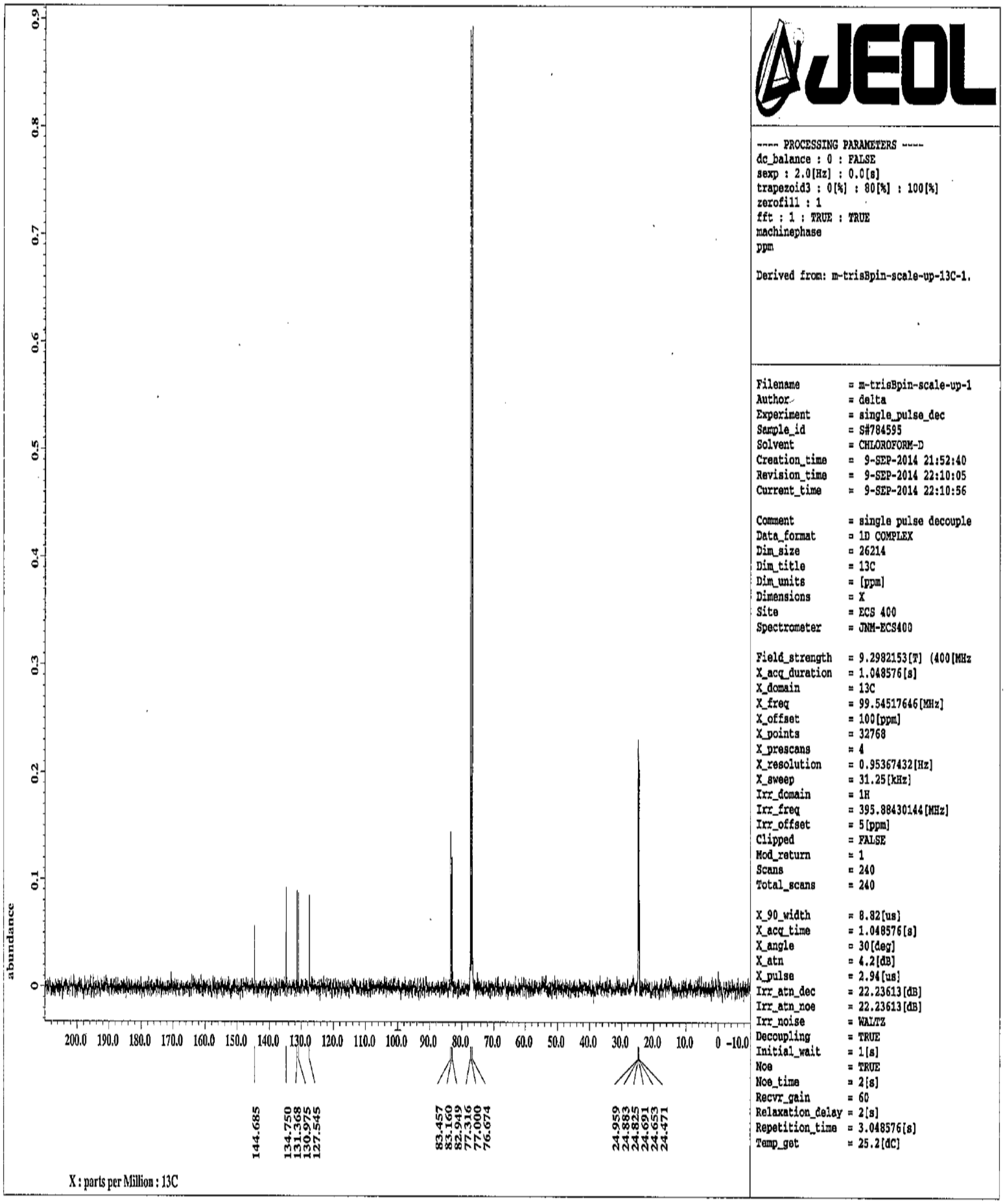

Supplementary Figure 86 | ^{13}C NMR spectrum of 11a.

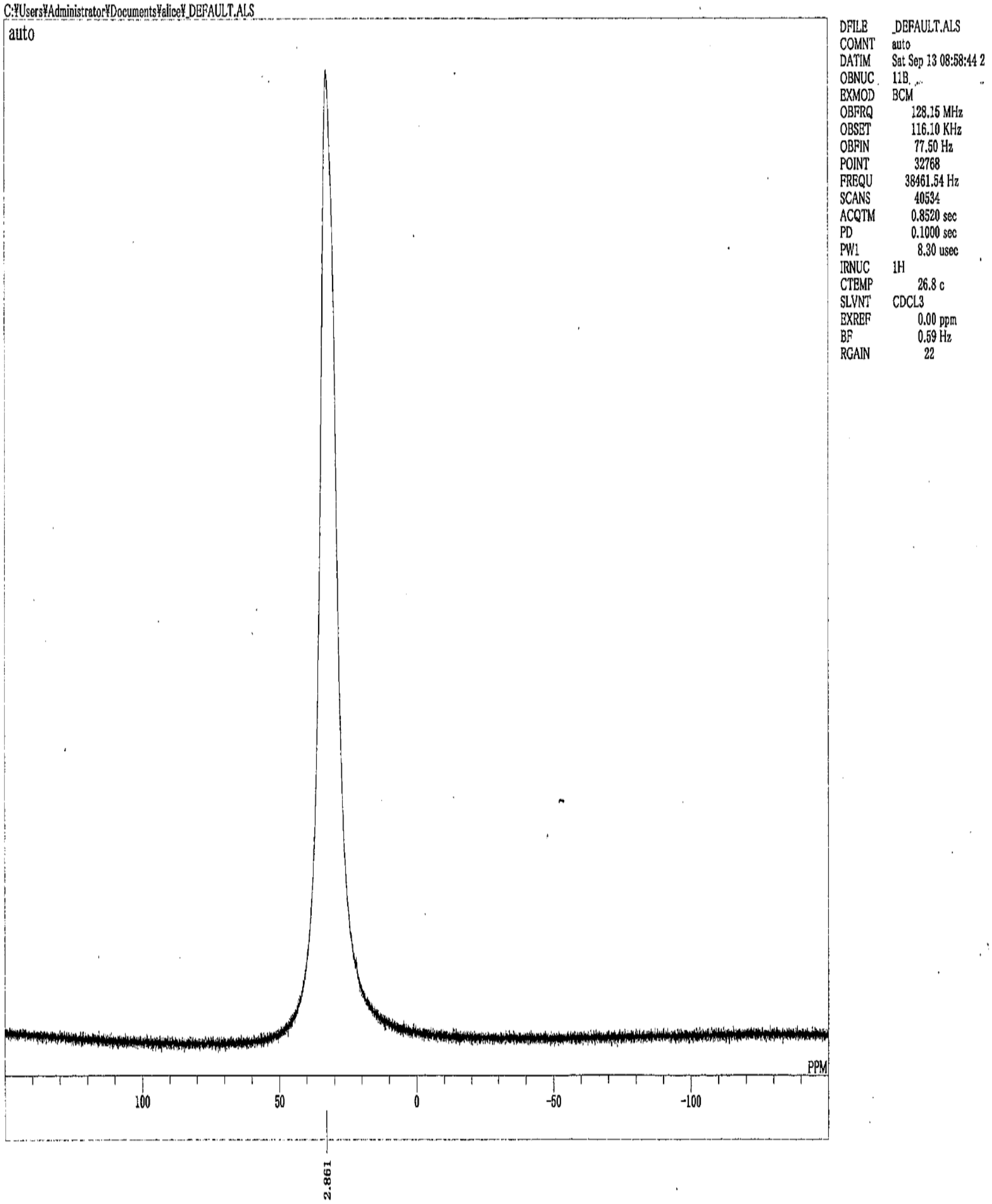

Supplementary Figure 87 | ^{11}B NMR spectrum of 11a.

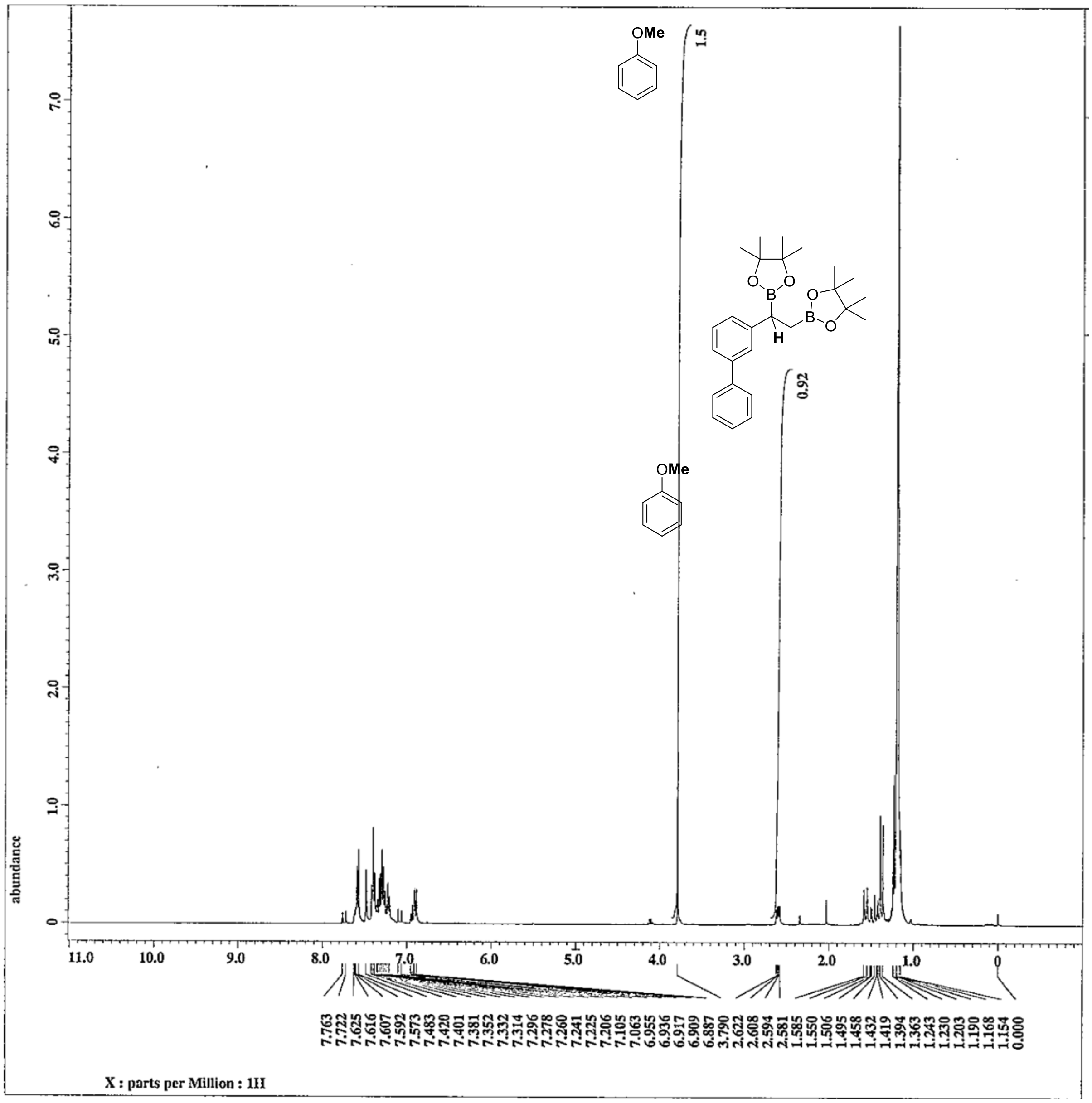

Supplementary Figure 88 | ^{1}H NMR spectrum used to determine NMR yield of sp^{2} arylation.

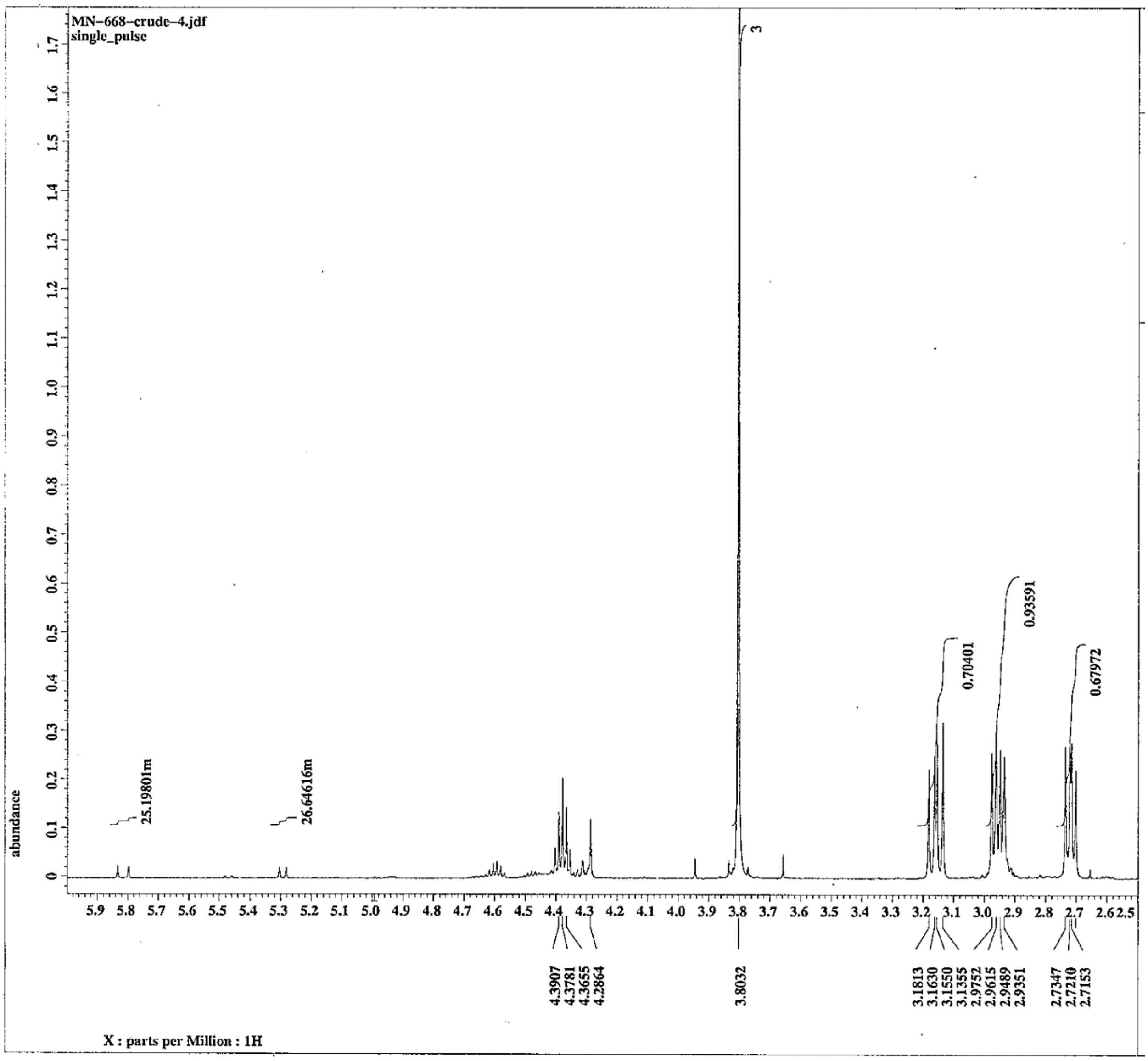

Supplementary Figure 89 | ^{1}H NMR spectrum used to determine NMR yield of primary sp^{3} arylation.

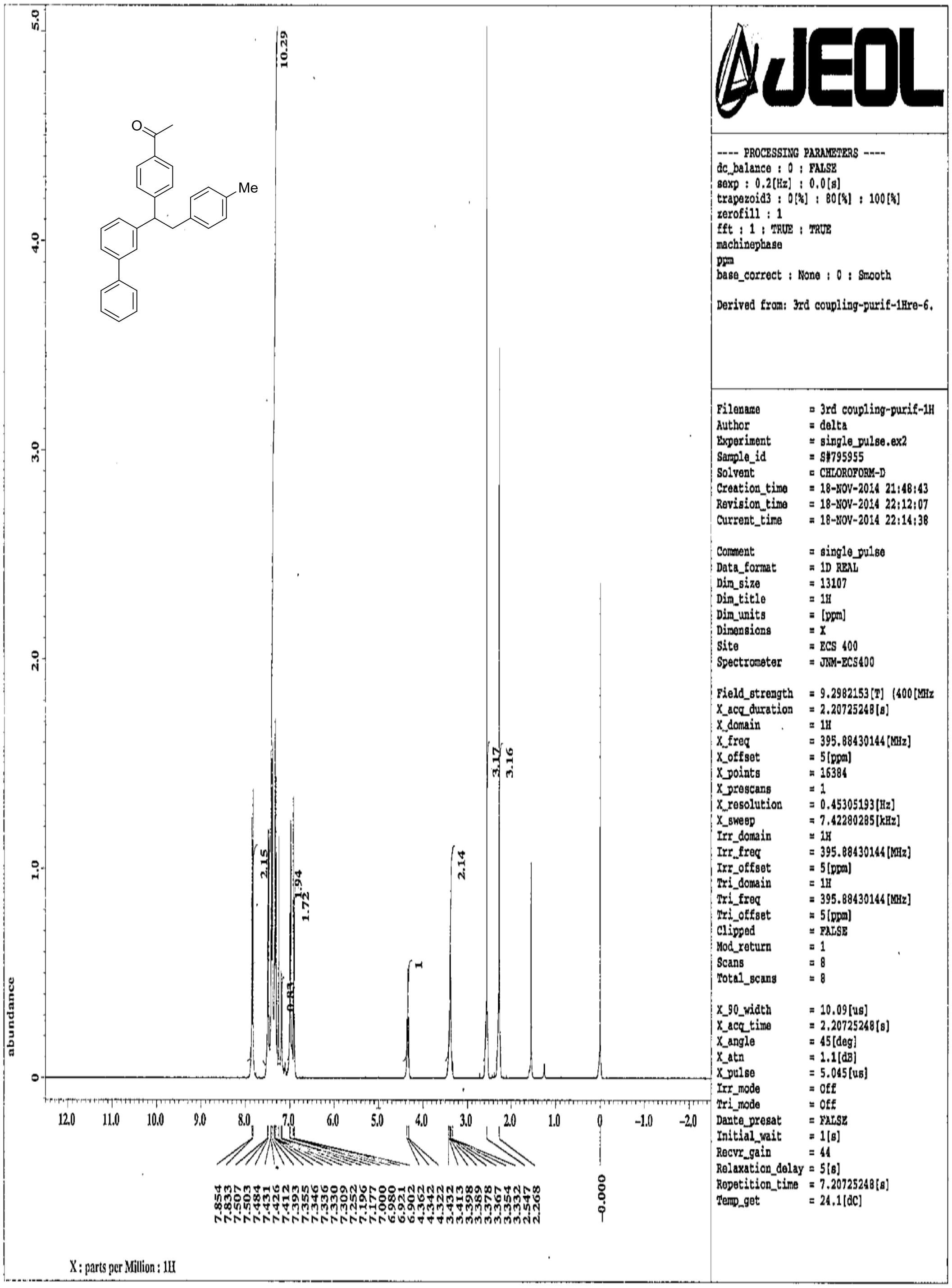

Supplementary Figure 90 | ^{1}H NMR spectrum of 12ABaJ.

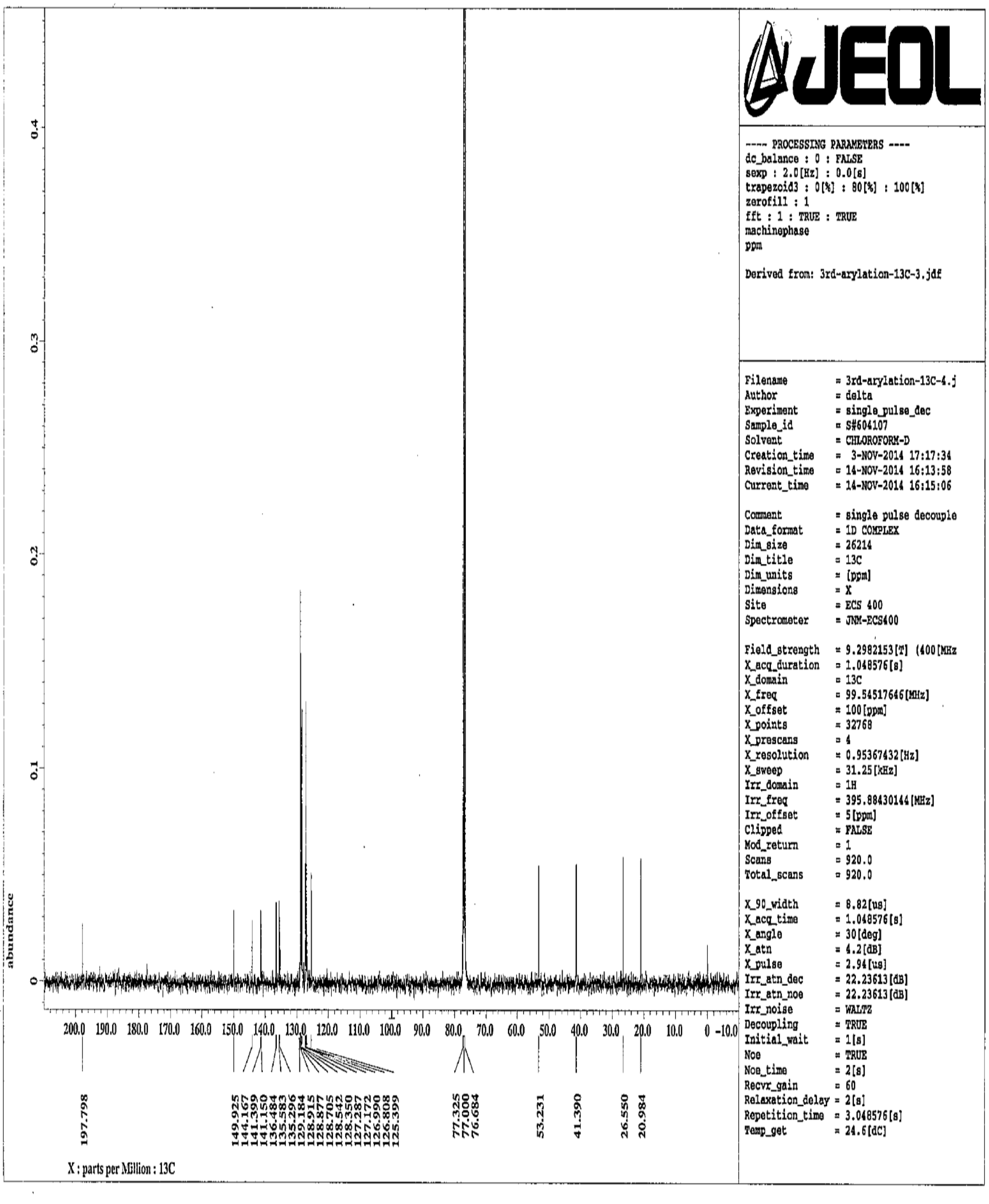

Supplementary Figure 91 | ^{13}C NMR spectrum of 12ABaJ.

a)

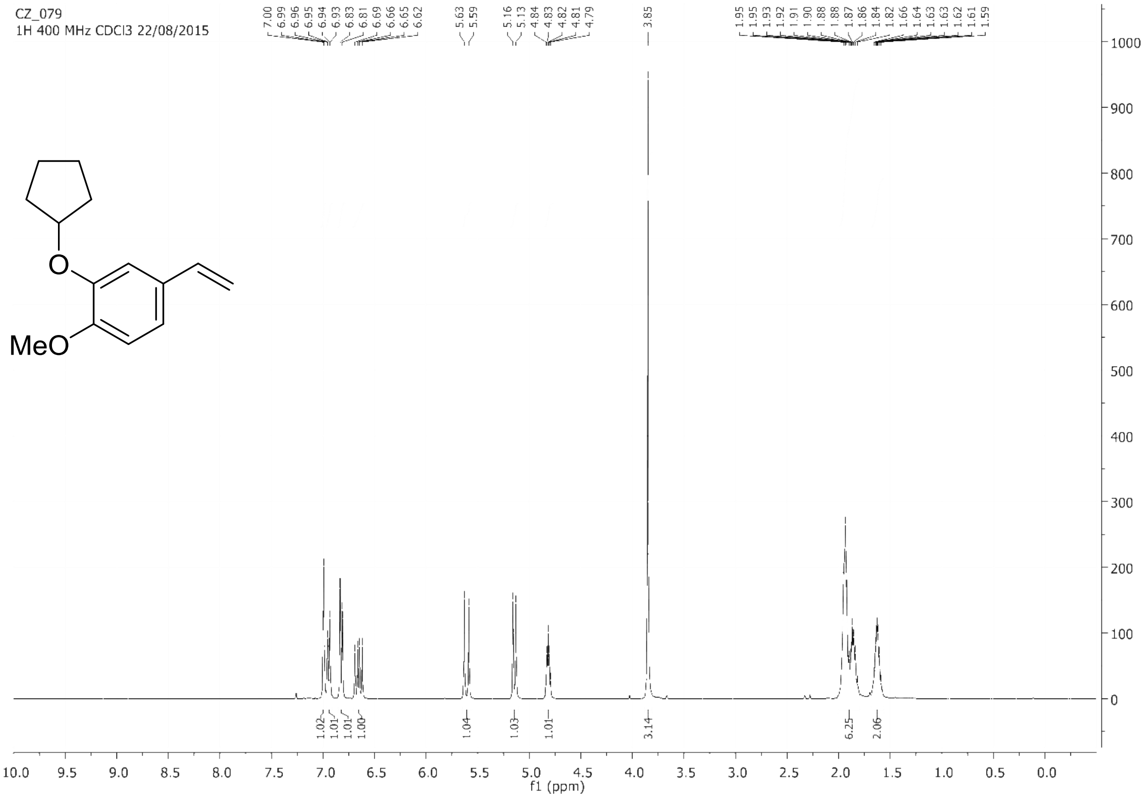

b)

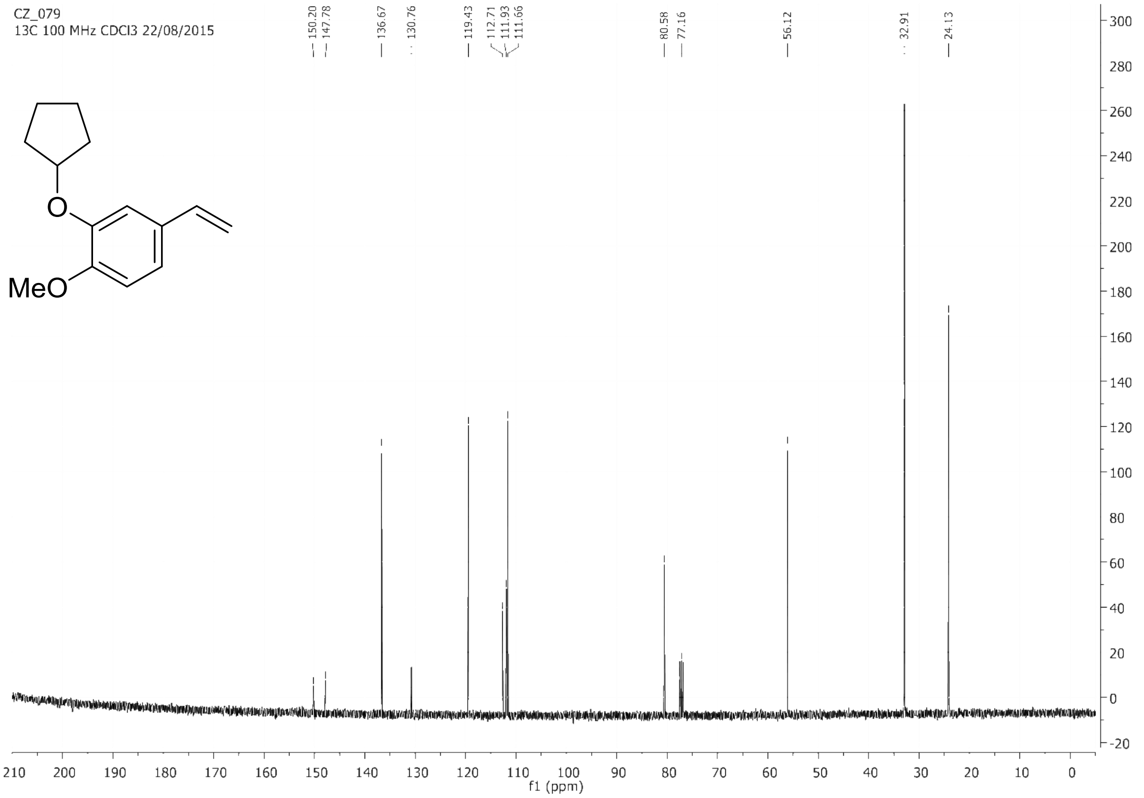

Supplementary Figure 92 │NMR spectra of 13. a) ^{1}H NMR spectrum. b) ^{13}C NMR spectrum.

a)

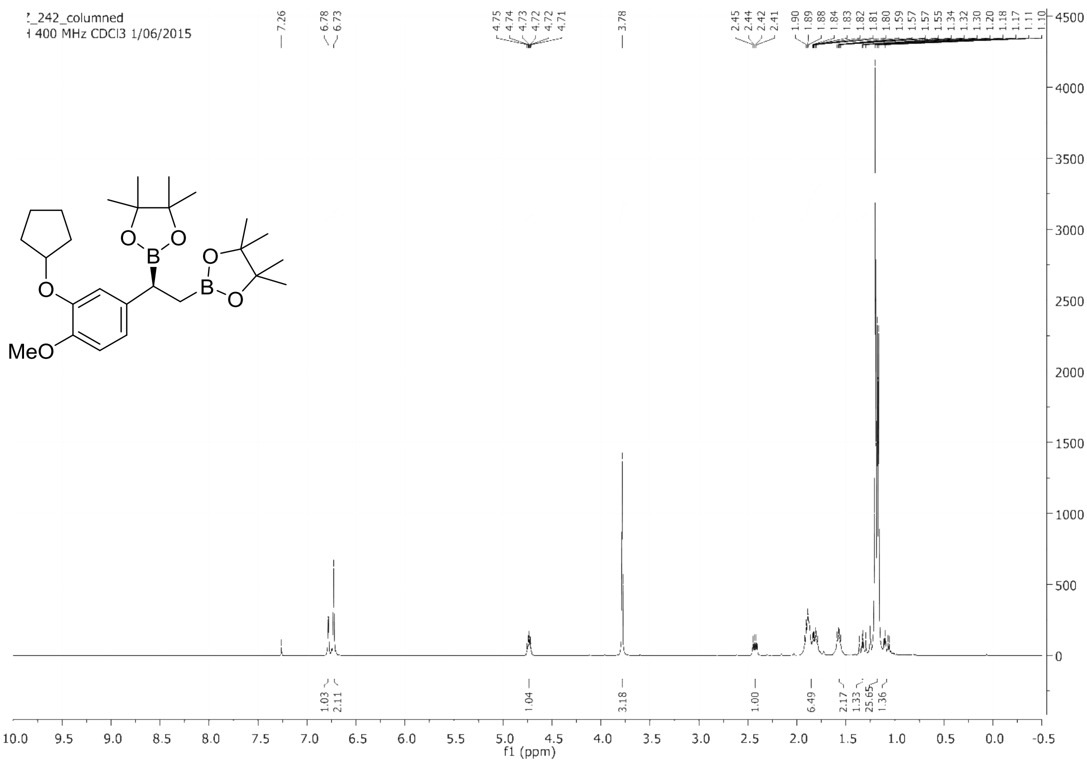

b)

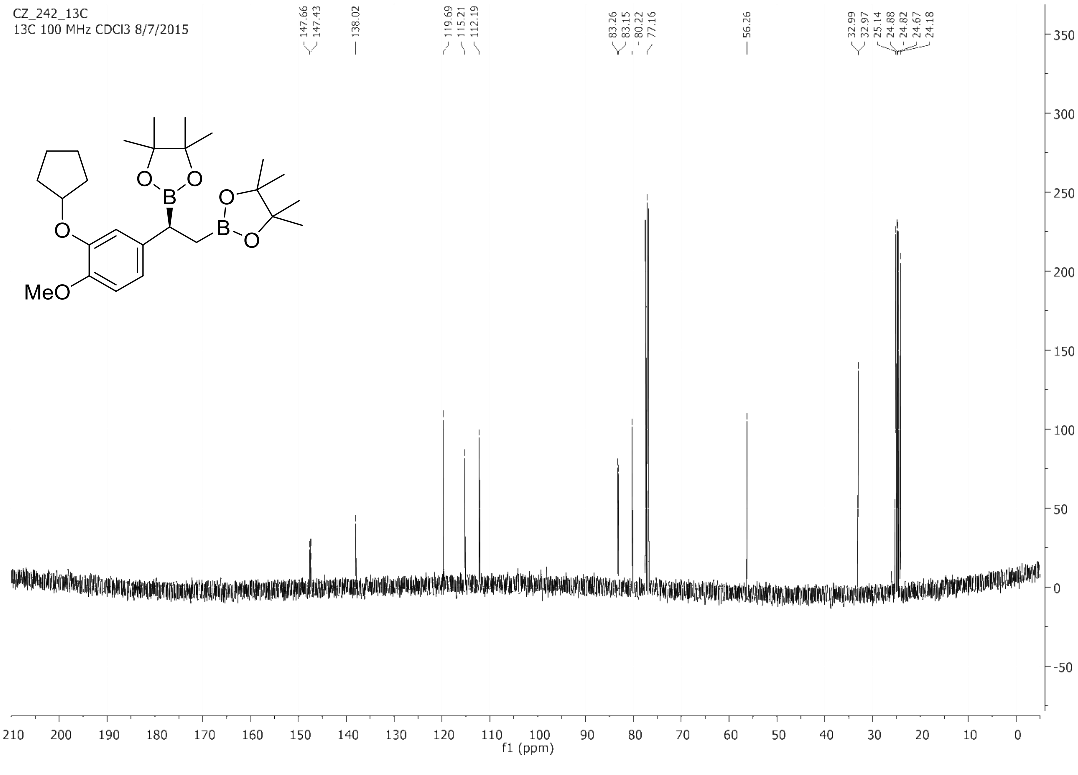

Supplementary Figure 93 | NMR spectra of 16. a) ^{1}H NMR spectrum. b) ^{13}C NMR spectrum.

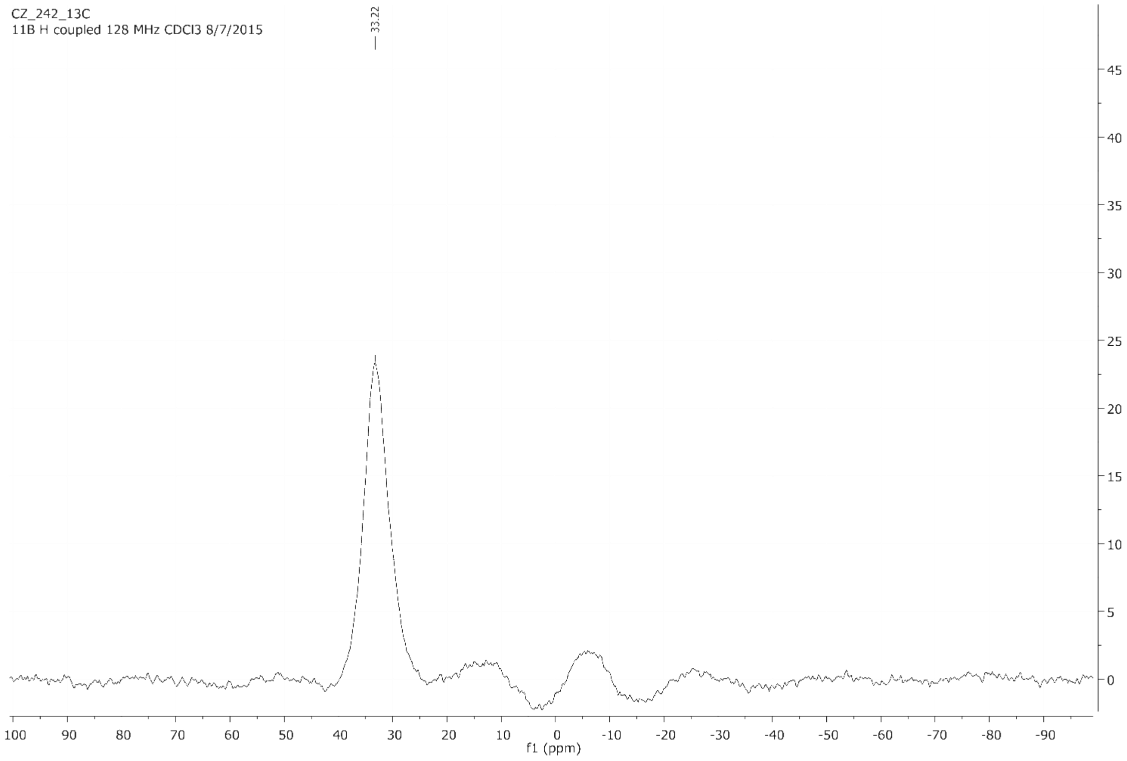

Supplementary Figure 94 | ^{11}B NMR spectrum of 16.

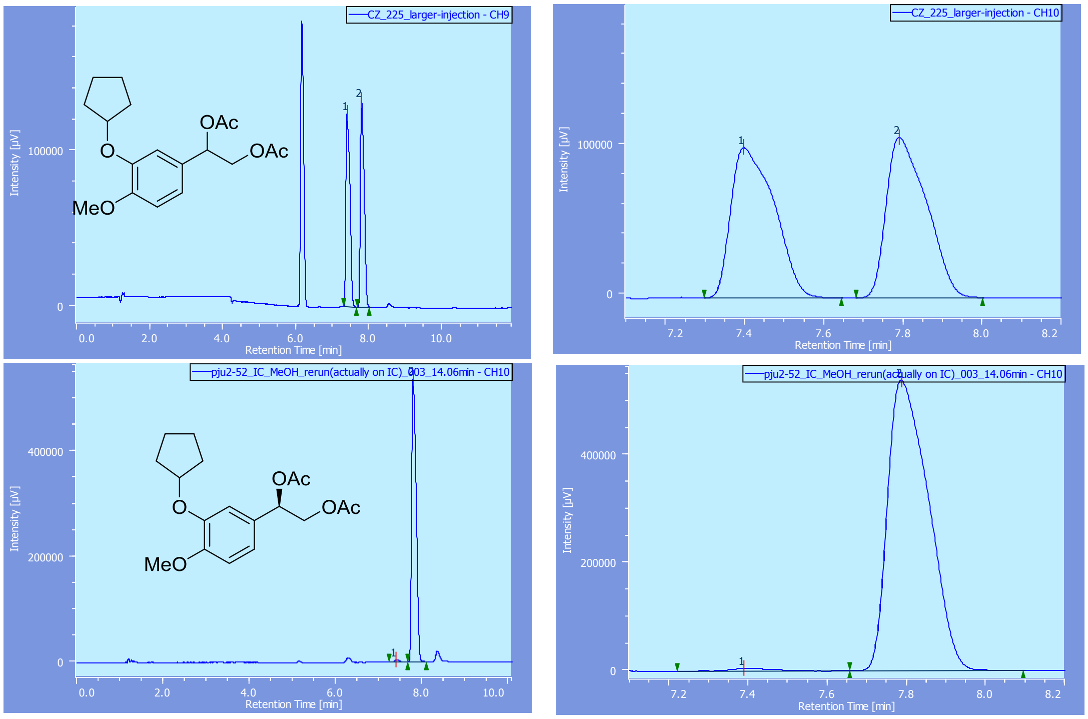

Supplementary Figure 95 | SFC spectra of oxidized and acylated 16.

a)

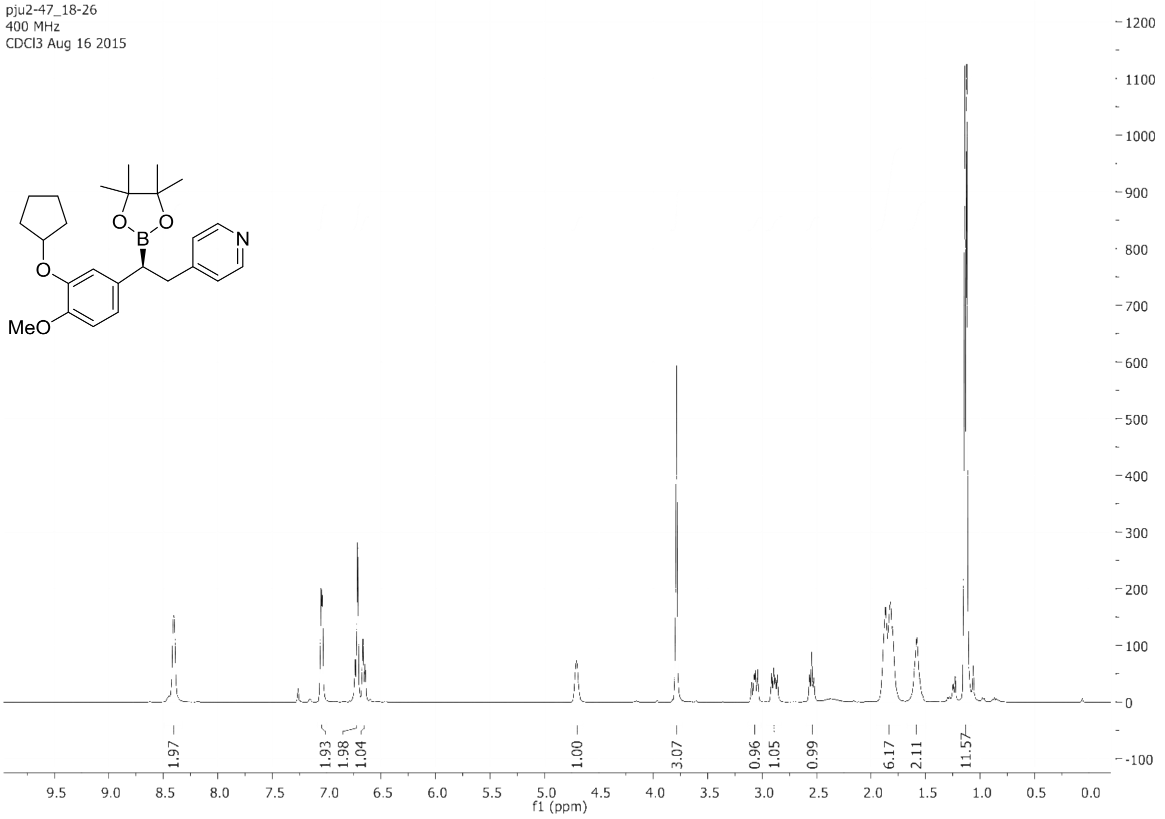

b)

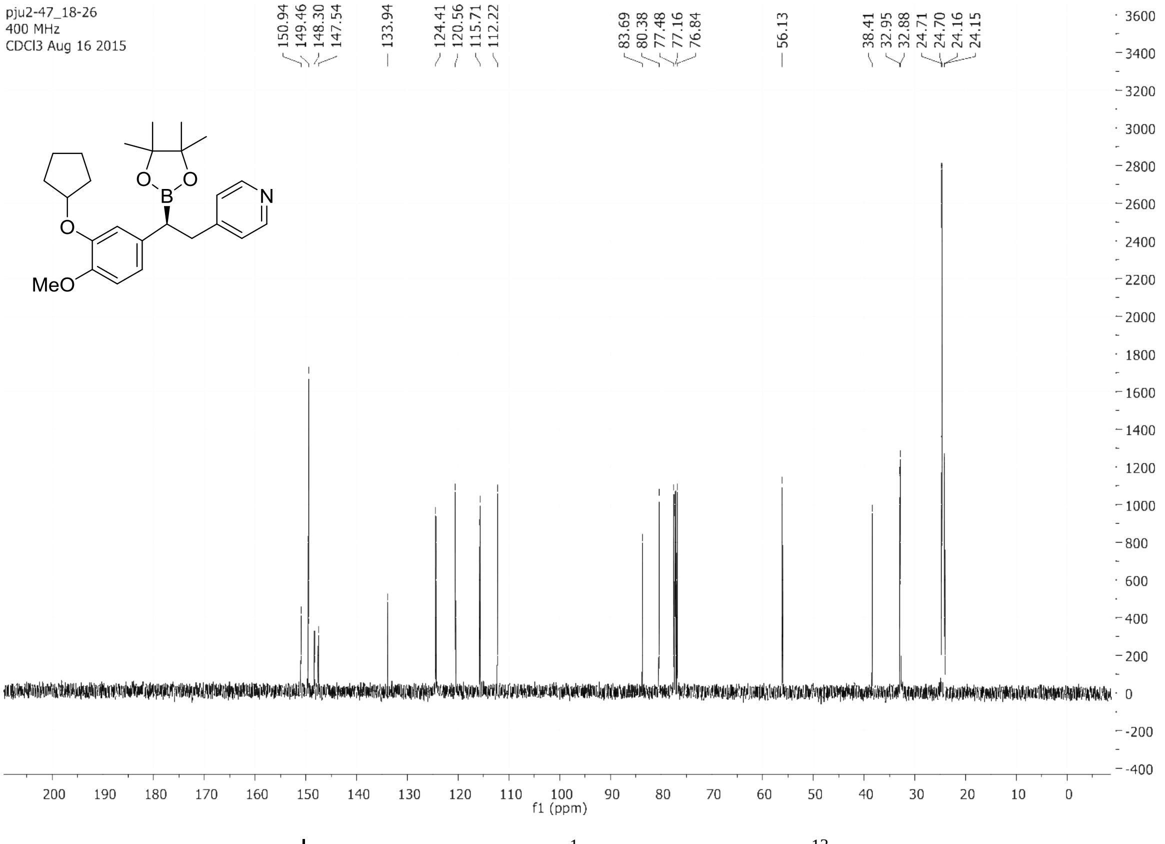

Supplementary Figure 96 | NMR spectra of 17. a) ^{1}H NMR spectrum. b) ^{13}C NMR spectrum.

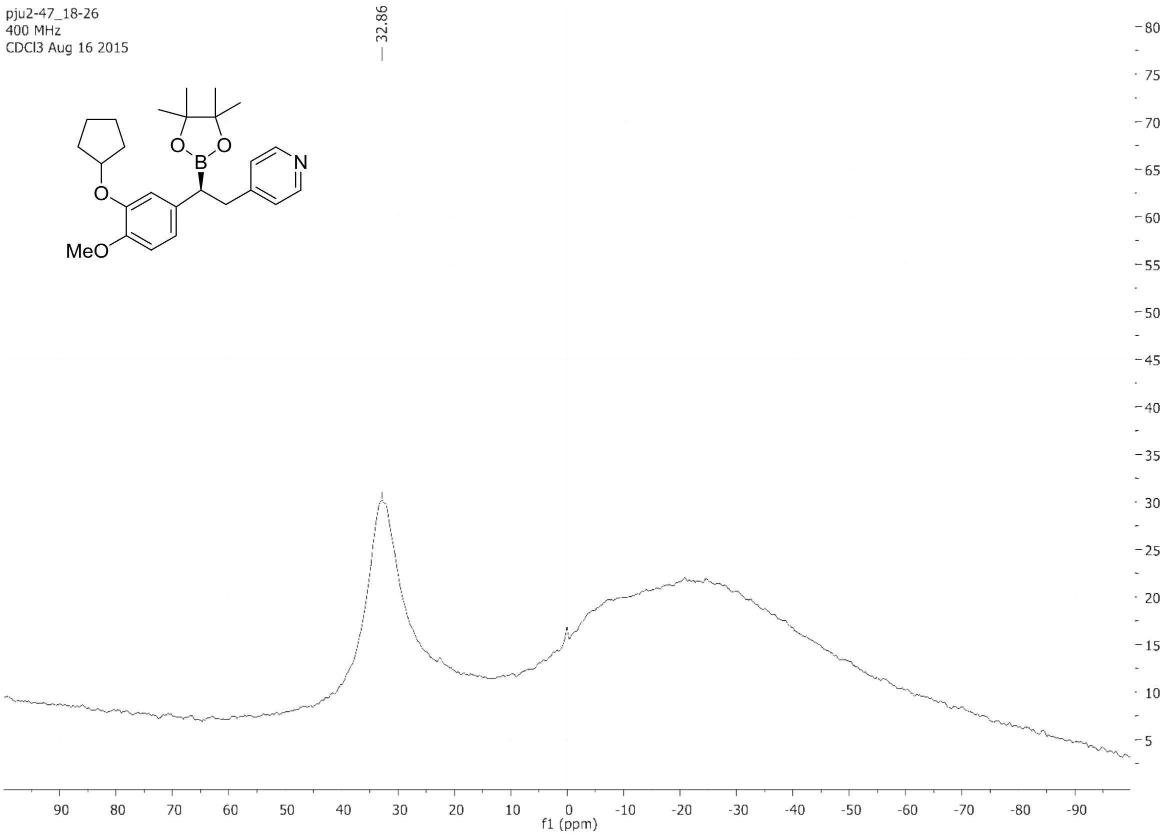

Supplementary Figure 97 | ^{11}B NMR spectrum of 17

a)

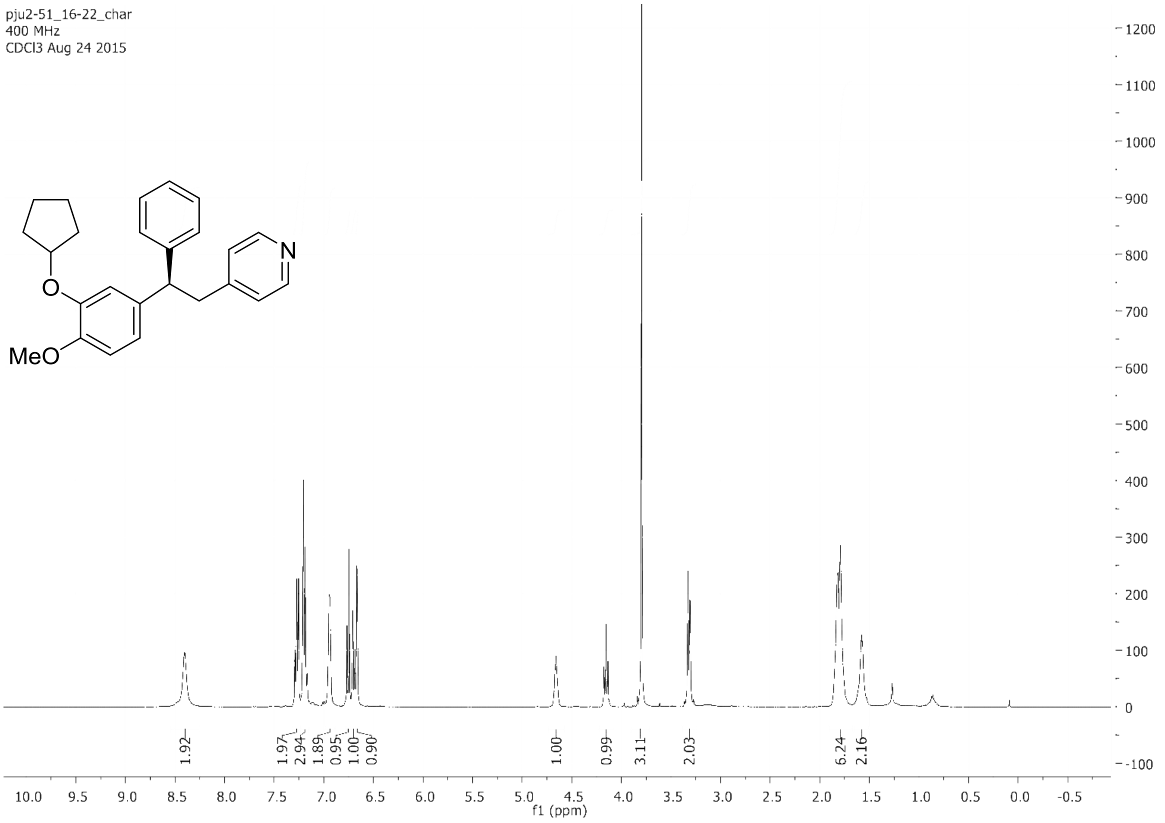

b)

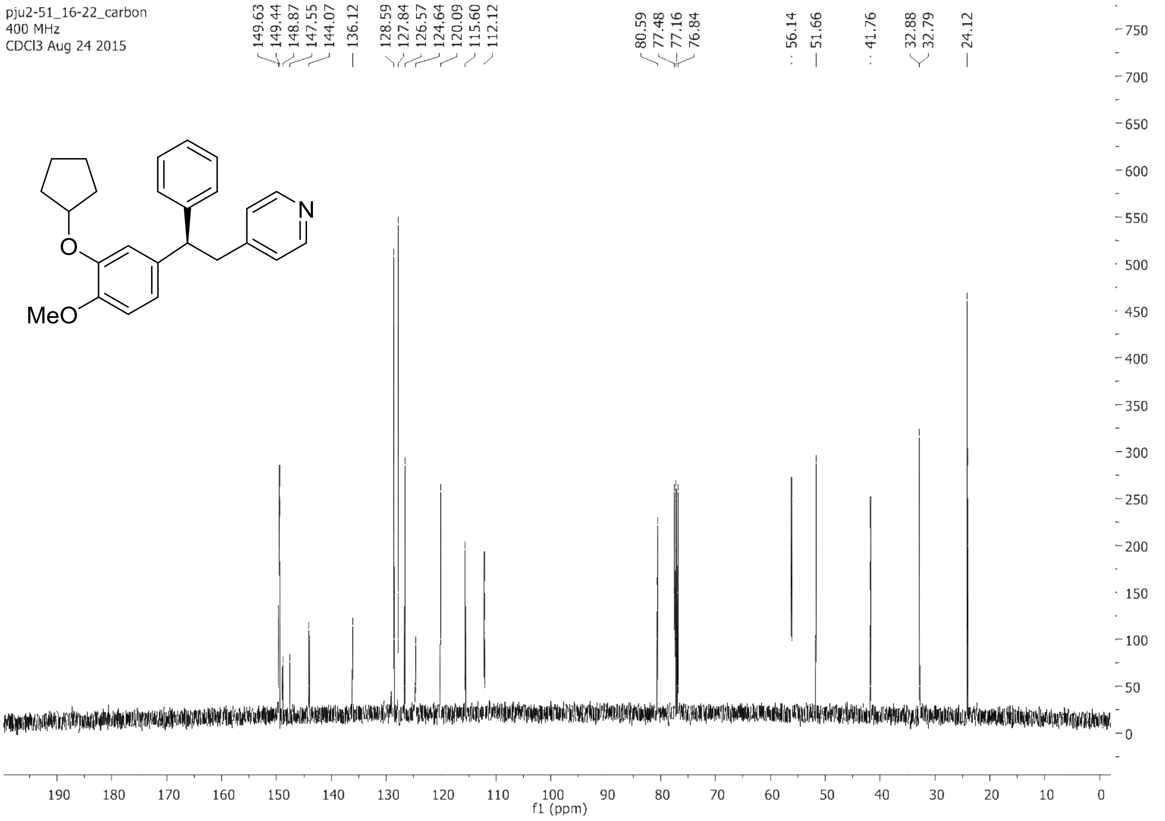

Supplementary Figure 98 | NMR spectra of CDP840 (14). a) ^{1}H NMR spectrum. b) ^{13}C NMR spectrum.

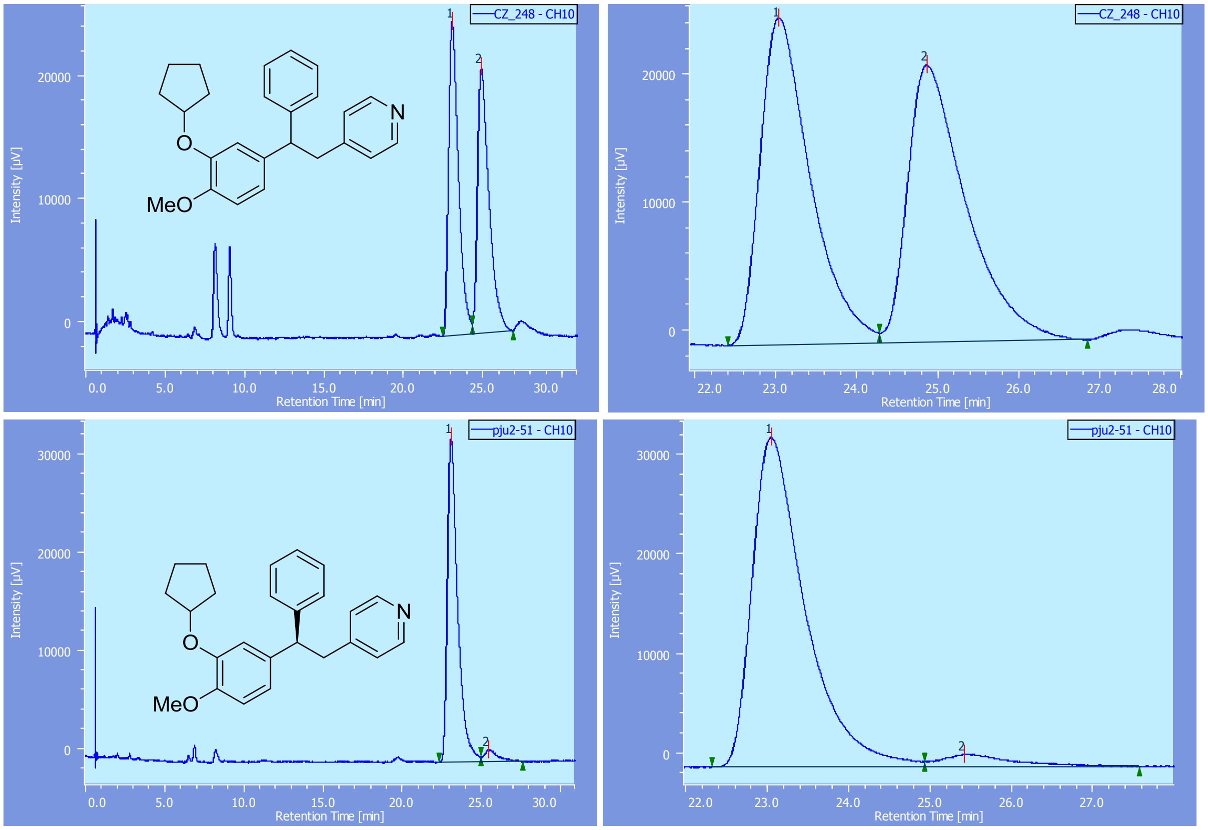

Supplementary Figure 99 | SFC traces of CDP840 (14).

Supplementary Methods

General Experimental Conditions

Unless otherwise specified, all manipulations were carried out under at atmosphere of dry argon in oven-dried glassware or under a nitrogen atmosphere in an M. Braun glovebox with oxygen and water levels <2 ppm. Toluene, THF, and 1,2-dimethoxyethane (DME) were distilled from either calcium hydride (CaH_2) or sodium metal (Na), deoxygenated with a minimum of three freeze-pump-thaw cycles and stored under N_2 or Ar over molecular sieves (4Å) prior to use. Anhydrous methanol (MeOH) was purchased from Avantor Performance Materials and used without further purification. Catechol borane was purified by distillation under reduced pressure and stored at -20 °C under nitrogen.

Silver (I) oxide was synthesized according to a literature procedure.[1] Triphenylphosphine and 1,4-bis(diphenylphosphino)butane were recrystallized from hot ethanol and stored in the glovebox. $Pd(dba)_2$, $Pd_2(dba)_3$, and $[Rh(cod)_2][BF_4]$ were store and used in an M. Braun glovebox as purchased from either Aldrich or Johnson Matthey. Distilled water was deoxygenated by sparging with argon for a minimum of 30 minutes prior to use

IR spectra were collected on a Bruker ALPHA Platinum ATR as neat solids and absorption bands ($\tilde{\nu}$, s = strong, m = medium, w = weak) are given in cm^{-1}. Melting points were recorded on an electrothermal MEL-TEMP apparatus connected to a Fluke 51 II Thermometer. Temperatures are given in degree Celsius (°C) and are uncorrected. Thin Layer Chromatography was performed on aluminum-backed silica plates and visualized by UV (254, 365 nm). The indicators utilized to stain were phosphomolybdic acid or potassium permanganate. Preparative thin-layer chromatography (PTLC) was performed using Wakogel B5-F silica coated plates (0.75 mm) prepared in our laboratory. Preparative recycling HPLC (GPC) was performed with a JAI LC-9204 instrument equipped with JAIGEL-1H/JAIGEL-2H columns using chloroform as an eluent. Column chromatography was performed by using flash grade silica (Silicycle, 40-63 µm particle size, 60 Å porosity) and reagent-grade solvents. All GC-MS spectra were obtained using an Agilent Technologies 5975CVL-MSD (triple axis detector) with a capillary measuring 30 m by 250 µm by 0.25 µm nominal, 250 inlet, splitless detector or Shimadzu GCMS-QP2010 instrument equipped with a HP-5 column (30 m × 0.25 mm,

Hewlett-Packard). High Resolution Mass Spectroscopy (HRMS) was performed on the Voyager DE STR MALDI TOF instrument or a JMS-T100TD instrument (DART) and Thermo Fisher Scientific Exactive.

NMR spectra were recorded on Bruker Avance 300 (^{1}H: 300.13 ^{13}C: 75.47), Bruker Avance 400 (^{1}H: 400.13, ^{11}B: 128.38, ^{13}C: 100.62), Bruker Avance 500 (^{1}H: 500.19, ^{11}B: 160.27, ^{13}C: 125.62) or JEOL ECS-400 and JEOL AL-400 (^{1}H: 400.13, ^{11}B: 128.38, ^{13}C: 100.62) instruments operating at the denoted spectrometer frequency given in mega Hertz (MHz) for the specified nucleus. All NMR samples were prepared using $CDCl_3$. To specify the signal multiplicity, the following abbreviations are used: s = singlet, d = doublet, t = triplet, q = quartet, and m = multiplet; br indicates a broad resonance; app = apparent. Shifts are reported in parts per million (ppm) relative to tetramethylsilane (TMS) as an external standard for ^{1}H- and ^{13}C NMR spectra and calibrated against the solvent residual peak or in case of proteo-solvents against known solvent resonances.[2] SFC (supercritical fluid chromatography) traces for the assessment of enantiopurity were obtained from a JASCO Instruments SFC HPLC equipped with HPLC columns (CHIRALPAK IA, IB, IC, ID, IE, IF: length 250 mm, ø 4.6 mm, particle size 5 μm), operating at the stated flow-rate and pressure of supercritical CO_2 with the indicated amount of admixed modifier solvent or on a Berger SFC HPLC using the specified chiracel Berger Silica column and specified conditions of co-eluent, flow rate and pressure. Retention times are t_R are given in minutes (min). Optical rotations $[\alpha]^{22}_{D}$ were measured on a Perkin-Elmer 241MC polarimeter as solutions in dichloromethane at concentration of 0.01 g mL^{-1} at 22 °C at 589 nm in a 0.5 dm cell, and given as specific rotations (° cm^{2} g^{-1}).

(*R*)-2,2'-(1-Phenylethane-1,2-diyl)bis(4,4,5,5-tetramethyl-1,3,2-dioxaborolane) 5a

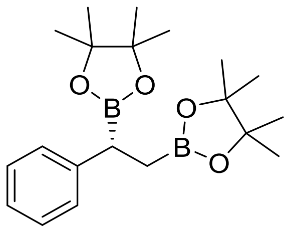

Prepared according to the procedure by Morken and co-workers.[3] To a flame-dried, round bottom flask with magnetic stir bar in a nitrogen filled glove box was added Pt(dba)$_3$ (218.6 mg, 0.24 mmol), (*R*,*R*)-3,5-diethylphenyl-TADDOLPPh (382.5 mg, 0.48 mmol), B_2pin$_2$ (2.13 g, 8.4 mmol) and THF (80 mL). The flask was sealed with a septum, removed from the glove box, and heated to 80 °C in an oil bath for 30 min. The flask was cooled to room temperature, returned to the glove box and charged with styrene (833 mg, 8 mmol). The flask was sealed, removed from the glove box, and stirred at 60 °C for 12 h. The reaction mixture was cooled and filtered through a plug of silica, eluted with EtOAc and concentrated *in vacuo*. Purification by column chromatography (hexane/acetone (97:3)) gave **5a** as a white solid (1.52 g, 52% yield); e.r. 92:8. The e.r. of the diol was determined by analysis by chiral SFC:

IF, 2mL/min, 100 bar, 50 °C, 1% → 40% MeOH over 10 min then 40% MeOH for 1 min; t_R 8.19 (major), 8.42 (minor).

Data were consistent with those reported in the literature.[4]

A racemic sample was prepared according to the procedure by Fernández and co-workers.[4]

In order to determine the e.r. by SFC analysis the diboronate was oxidised to the diol by the following general procedure (**GP1**): To a solution of diboronate (1 mmol) in THF (5 mL) was added a 1:1 mixture of 2 M aq. NaOH/30% aq. H_2O_2 (1 mL) at 0 °C. The mixture was warmed to RT and stirred until the reaction was complete by TLC analysis (usually less than 1 h). Water (5 mL) and Et_2O (5 mL) were added and the layers were separated. The aqueous phase was extracted with Et_2O (2 × 5 mL). The combined organics were washed with brine (10 mL), dried ($MgSO_4$), filtered and concentrated *in vacuo*.

Diboronate **5a** was also prepared by a procedure according to Nishiyama and co-workers.[5] B_2pin_2 (1.86 g, 7.32 mmol, 1.2 eq), NaO^{t}Bu (29.3 mg, 0.305 mmol, 0.05 eq) and [Rh{(*R*,*R*)-Phebox-*i*Pr}$OAc_2(H_2O)$][6] (32.8 mg, 0.061 mmol, 0.01 eq) were placed in a dry Schlenk flask and placed under argon. THF (12 mL) and styrene (700 µL, 6.10 mmol, 1 eq) were added and the reaction was immediately placed into a preheated oil bath at 60 °C and stirred for 1 h. The reaction mixture was cooled and aq. sat. NH_4Cl (50 mL) and Et_2O (50 mL) were added. The layers were separated and the aqueous phase was extracted with Et_2O (2 × 50 mL). The combined organics were washed with brine (50 mL), dried ($MgSO_4$), filtered and concentrated *in vacuo*. Purification by column chromatography (pentane/EtOAc (20:1)) gave **5a** as a white solid (1.45 g, 66% yield); e.r. 98:2. The e.r. was determined by oxidation to the diol (by **GP1**) followed by analysis by chiral SFC:

IF, 2mL/min, 100 bar, 50 °C, 1% → 40% MeOH over 10 min then 40% MeOH for 1 min; t_R 8.19 (major), 8.42 (minor).

$[\alpha]_D^{20}$ = –28.0 (*c* 1.0, $CHCl_3$) {Lit.[7] $[\alpha]_D^{20}$ = –24.2 (*c* 2.8, $CHCl_3$) for e.r. 94.5:5.5}.

Diboronate **5a** was also prepared using the same procedure as above but with 0.2 mol% of [Rh{(*R*,*R*)-Phebox-*i*Pr}$OAc_2(H_2O)$] catalyst in 52% yield (1.44 g) and 93.5:6.5 e.r. The e.r. was determined by oxidation to the diol (by **GP1**) followed by analysis by chiral SFC:

IF, 2mL/min, 100 bar, 50 °C, 1% → 40% MeOH over 10 min then 40% MeOH for 1 min; t_R 8.19 (major), 8.42 (minor).

2,2'-(1-(*p*-Tolyl)ethane-1,2-diyl)bis(4,4,5,5-tetramethyl-1,3,2-dioxaborolane) 5b

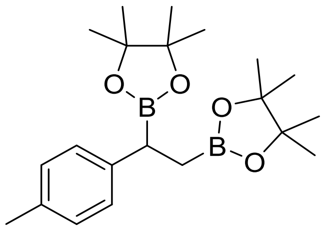

In a nitrogen filled glovebox, 4-methylstyrene (0.34 g, 2.9 mmol, 1.9 eq) and B_2pin_2 (0.38 g, 1.5 mmol, 1 eq) were added to a round bottom flask and dissolved in toluene (6 mL). The reaction was sealed with a septum and taken out of the glove box. Platinum(0)-1,3-divinyl-1,1,3,3-tetramethyldisiloxane complex solution (in xylene, Pt ~2 %) (0.45 mL) was added *via* syringe. The reaction mixture was stirred at 50 °C for 3 h. The reaction mixture was cooled and concentrated *in vacuo.* Purification by column chromatography (hexane/EtOAc (93:7)) gave **5b** as a white solid (440 mg, 79% yield).

^{1}H NMR ($CDCl_3$, 400 MHz): 7.12 (d, 8.0 Hz, Ar**H**), 7.04 (d, *J* = 7.9 Hz, Ar**H**), 2.48 (dd, *J* = 11.0 Hz, 5.8 Hz, C**H**BCHH'), 2.29 (s, ArC**H**$_3$), 1.36 (dd, *J* = 16.0 Hz, 11.0 Hz, CHBC**H**H'), 1.22 (s, 12H, C(C**H**$_3$)$_2$C(C**H**$_3$)$_2$), 1.20 (s, 6H, C(C**H**$_3$)(CH$_3$)'C(C**H**$_3$)(CH$_3$)')), 1.19 (s, 6H, C(CH$_3$)(C**H**$_3$)'C(CH$_3$)(C**H**$_3$)')), 1.09 (dd, *J* = 16.0 Hz, 5.8 Hz, CHBCH**H'**); **^{13}C NMR (100 MHz, $CDCl_3$)**: 142.3 (**C**), 134.1 (**C**), 128.8 (**C**H), 127.7 (**C**H), 83.1 (**C**-OB), 82.9 (**C**-OB), 24.9 (C**C**H$_3$), 24.7 (C**C**H$_3$), 24.6 (C**C**H$_3$), 24.5 (C**C**H$_3$), 20.9 (Ar**C**H$_3$), peaks not observed for **C**'s bound to B's; **^{11}B NMR (128 MHz, $CDCl_3$)**: 33.6; **HRMS (ESI$^+$)**: $C_{21}H_{35}B_2O_4$ (M + H$^+$) requires 373.2722; found 373.2710.

2,2'-(1-(4-Methoxyphenyl)ethane-1,2-diyl)bis(4,4,5,5-tetramethyl-1,3,2-dioxaborolane) 5f

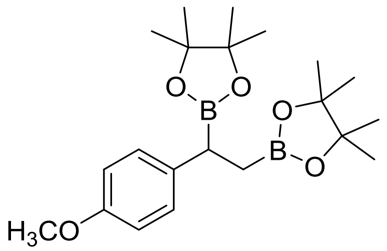

In a nitrogen filled glovebox, 4-vinylanisole (0.23 mL, 1.74 mmol) and B_2pin_2 (0.254 g, 1 mmol) were added to a round bottom flask and dissolved in toluene (4 mL). The reaction was sealed with a septum and taken out of the glove box. Platinum(0)-1,3-divinyl-1,1,3,3-tetramethyldisiloxane complex solution (in xylene, Pt ~2 %) (0.3 mL) was added *via* syringe. The reaction mixture was stirred at 50 °C for 3 h. The reaction mixture was cooled and concentrated *in vacuo.* Purification by column chromatography (hexane/EtOAc (93:7)) gave **5f** as a white solid (105 mg, 27% yield).

^{1}H NMR ($CDCl_3$, 400 MHz): 7.15 (d, *J* = 8.7 Hz, Ar**H**), 6.79 (d, *J* = 8.7 Hz, Ar**H**), 3.77 (s, OC**H**$_3$), 2.45 (dd, *J* = 11.0 Hz, 5.8 Hz, C**H**BCHH'), 1.33 (dd, *J* = 16.0 Hz, 11.0 Hz, CHBC**H**H'), 1.21 (s, 12H, C(C**H**$_3$)$_2$C(C**H**$_3$)$_2$), 1.20 (s, 6H, C(C**H**$_3$)(CH$_3$)'C(C**H**$_3$)(CH$_3$)')), 1.18 (s, 6H, C(CH$_3$)(C**H**$_3$)'C(CH$_3$)(C**H**$_3$)')), 1.09 (dd, *J* = 16.0 Hz, 5.8 Hz, CHBCH**H'**); **^{13}C NMR (100 MHz, $CDCl_3$)**: 157.1 (**C**), 137.5 (**C**), 128.7 (**C**H), 113.6 (**C**H), 83.1 (**C**-OB), 82.9 (**C**-OB), 55.2 (O**C**H$_3$), 24.9 (C**C**H$_3$), 24.7 (C**C**H$_3$), 24.6 (C**C**H$_3$), 24.5 (C**C**H$_3$), peaks not observed for **C**'s bound to B's; **^{11}B NMR (128 MHz, $CDCl_3$)**: 33.6; **HRMS (ESI$^+$)**: $C_{21}H_{35}B_2O_5$ (M + H^+) requires 389.2665; found 389.2676.

(*R*)-2,2'-(1-(Benzo[d][1,3]dioxol-5-yl)ethane-1,2-diyl)bis(4,4,5,5-tetramethyl-1,3,2-dioxaborolane) 5g

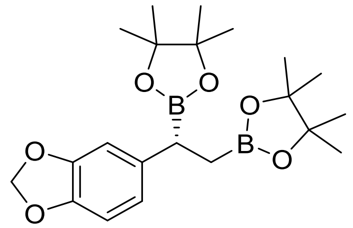

Prepared according to the procedure by Morken and co-workers.[3] To a flame-dried, round bottom flask with magnetic stir bar in a nitrogen filled glove box was added Pt(dba)$_3$ (53.9 mg, 0.06 mmol), (*R,R*)-3,5-diethylphenyl-TADDOLPPh (57.4 mg, 0.072 mmol), B$_2$pin$_2$ (533 mg, 2.1 mmol) and tetrahydrofuran (2.4 mL). The flask was sealed with a septum, removed from the glove box, and heated to 80 °C in an oil bath for 30 min. The flask was cooled to room temperature, returned to the glove box and a solution of 5-vinylbenzo[d][1,3]dioxole[8] (296 mg, 2 mmol) in THF (1.2 mL) was added. The flask was sealed, removed from the glove box, and stirred at 60 °C for 12 h. The reaction mixture was cooled and filtered through a plug of silica, eluted with EtOAc and concentrated *in vacuo*. Purification by column chromatography (hexane/EtOAc (99:1 → 96:4)) gave **5g** as a yellow solid (453 mg, 54% yield); e.r. 93.5:6.5.

^{1}H NMR (CDCl$_3$, 400 MHz): 6.74 (s, 1H, Ar**H**), 6.70-6.64 (brs, 2H, Ar**H**), 5.88 (s, 2H, OC**H**$_2$O), 2.44 (dd, J = 10.8 Hz, 5.7 Hz, 1H, C**H**BCHH’), 1.31 (dd, J = 16.0 Hz, 10.8 Hz, 1H, CHBC**H**H'), 1.21 (s, 12H, C(C**H**$_3$)$_2$C(C**H**$_3$)$_2$), 1.20 (s, 6H, C(C**H**$_3$)(CH$_3$)'C(C**H**$_3$)(CH$_3$)')), 1.19 (s, 6H, C(CH$_3$)(C**H**$_3$)'C(CH$_3$)(C**H**$_3$)')), 1.06 (dd, J = 16.0 Hz, 5.7 Hz, CHBCH**H**'); **^{13}C NMR (100 MHz, CDCl$_3$)**: 147.4 (**C**), 145.1 (**C**), 139.5 (**C**), 120.7 (**C**H), 108.7 (**C**H), 108.1 (**C**H), 100.6 (**C**H$_2$), 83.4 (**C**-OB), 83.2 (**C**-OB), 26.3 (br, **C**HB), 25.1 (**C**H), 24.84 (**C**H), 24.80 (**C**H), 24.6 (**C**H), 15.1 (br, **C**H$_2$B); **^{11}B NMR (160 MHz, CDCl$_3$)**: 33.4; **HRMS (ESI$^+$)**: $C_{21}H_{33}B_2O_6$ (M + H$^+$) requires 403.2463; found 403.2477; mp 78-80 °C. The e.r. was determined by oxidation to the diol (by **GP1**) followed by analysis by chiral SFC: IA, 2mL/min, 100 bar, 50 °C, 1% → 40% EtOH over 10 min then 40% EtOH for 3 min; t_R 9.28 (major), 9.77 (minor).

2,2'-(1-(3-phenoxyphenyl)ethane-1,2-diyl)bis(4,4,5,5-tetramethyl-1,3,2-dioxaborolane) 5h

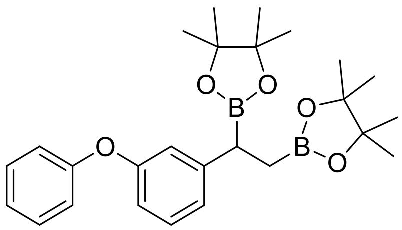

Prepared according to the procedure by Fernandez and co-workers.[4] To a flame-dried 15 mL pressure tube with magnetic stir bar in a nitrogen filled glove box was added 3-phenoxystyrene (368.95 mg, 1.88 mmol), NaO^{t}Bu (28.4 mg, 0.296 mmol), B_2pin_2 (531.75 mg, 2.09 mmol) and THF (5 mL). The tube was sealed with a septum, removed from the glove box, and placed under a flow of argon, where dry MeOH (190µL, 4.7mmol) was added via syringe. The tube was sealed with a screw cap and heated to 52 °C in an oil bath for 36 hours. The reaction was cooled to room temperature, transferred to a round bottom flask and concentrated *in vacuo*. Purification by column chromatography (hexane/ethyl acetate (gradient 50:1 to 15:1)) gave **5h** as a colorless oil (445 mg, 52% yield).

^{1}H NMR (400MHz, $CDCl_3$): 7.29-7.24 (m, 2H, Ar**H**), 7.17 (t, *J* = 7.9 Hz, 1H, Ar**H**), 7.03 (t, *J* = 7.9 Hz, 1H, Ar**H**), 6.97-6.96 (m, 3H, Ar**H**), 6.89 (m, 1H, Ar**H**), 6.75 (dd, *J* = 8.0 Hz, 1.6 Hz, 1H, Ar**H**) 2.48 (dd, *J* = 10.9 Hz, 5.5 Hz, 1H, C**H**BCHH'), 1.30 (dd, *J* = 16.0 Hz, 11.1 Hz, 1H, CHBC**H**H'), 1.18 (s, 12H, $C(C\mathbf{H}_3)_2C(C\mathbf{H}_3)_2$), 1.15 (s, 6H, $C(C\mathbf{H}_3)(CH_3)'C(C\mathbf{H}_3)(CH_3)'$), 1.14 (s, 6H, $C(CH_3)(C\mathbf{H}_3)'C(CH_3)(C\mathbf{H}_3)'$) 1.07 (dd, *J* = 16.0 Hz, 5.6 Hz, CHBCH**H'**) **^{13}C NMR (100 MHz, $CDCl_3$)**: 157.8 (**C**), 157.1 (**C**), 147.7 (**C**), 129.7 (**C**H), 129.5 (**C**H), 123.2 (**C**H), 122.9 (**C**H), 118.8 (**C**H), 118.8 (**C**H), 115.9 (**C**H), 83.4 (**C**-OB), 83.2 (**C**-OB), 26.7 (br, **C**HB) 25.1 (**C**H_3), 24.8 (**C**H_3), 24.6 (**C**H_3); 14.4 (br, **C**H_2B), **^{11}B NMR (128 MHz, $CDCl_3$)**: 32.31; **IR (film) cm^{-1}**: 3067m, 2929w, 1580w, 1483w, 1367m, 1315m, 1241m, 1138s **HRMS (ESI$^+$)**: $C_{26}H_{37}B_2O_5$ (M+H$^+$) requires 451.2822; found 451.2816.

(*R*)-2-(1,2-Diphenylethyl)-4,4,5,5-tetramethyl-1,3,2-dioxaborolane (compound **6aA**, Figure 3)

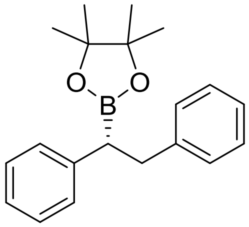

In a nitrogen filled glovebox, diboronate **5a** (200 mg, 0.56 mmol, 1 eq, e.r. 92:8), bromobenzene (105 mg, 0.67 mmol, 1.2 eq), $Pd(OAc)_2$ (12.5 mg, 0.0556 mmol, 0.1 eq), RuPhos (65.2 mg, 0.14 mmol, 0.25 eq) and K_2CO_3 (147 mg, 1.06 mmol, 1.9 eq) were weighed into a vial and THF (11.2 mL) was added. The reaction was sealed with a septum and removed from the glove box and placed under a flow of argon. Degassed water (0.56 mL) was added and the septum was replaced with a Teflon cap. The reaction mixture was sonicated for about 2 minutes before being stirred at 80 °C for 15 h. The reaction mixture was cooled and filtered through a plug of silica, eluted with EtOAc and concentrated *in vacuo*. Purification by column chromatography (hexane/EtOAc (97:3)) gave **6aA** (109 mg, 63% yield), e.r. was determined over two steps (see compounds **7aAi**, **7aAj**).

Data were consistent with those reported in the literature.[9]

(*R*)-4,4,5,5-Tetramethyl-2-(1-phenyl-2-(*p*-tolyl)ethyl)-1,3,2-dioxaborolane (compound **6aB**, Figure 3)

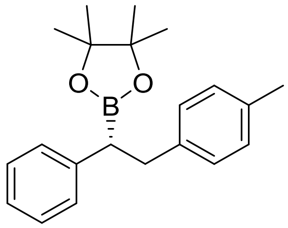

In a nitrogen filled glovebox, diboronate **5a** (100 mg, 0.28 mmol, 1 eq, e.r. 92:8), 4-bromotoluene (57 mg, 0.33 mmol, 1.2 eq), $Pd(OAc)_2$ (6.3 mg, 0.028 mmol, 0.1 eq), RuPhos (32.6 mg, 0.07 mmol, 0.25 eq), K_2CO_3 (73 mg, 0.53 mmol, 1.9 eq) were weighed into a vial and THF (5.6 mL) was added. The reaction was sealed with a septum and removed from the glove box and placed under a flow of argon. Degassed water (0.28 mL) was added and the septum was replaced with a Teflon cap. The reaction mixture was sonicated for about 2 minutes before being stirred at 80 °C for 15 h. The reaction mixture was cooled and filtered through a plug of silica, eluted with EtOAc and concentrated *in vacuo*. Purification by column chromatography (hexane/EtOAc (97:3)) gave **6aB** (62 mg, 69% yield), e.r. 92:8. The e.r. was determined by oxidation to the alcohol (by **GP1**) followed by analysis by chiral SFC:
IB, 2mL/min, 2% MeOH, 100 bar, T = 50 °C; t_R 16.5 (major), 17.7 (minor).

^{1}H NMR (400 MHz, $CDCl_3$): 7.29-7.27 (m, 4H, Ar**H**), 7.17 (m, 1H, Ar**H**), 7.10 (d, *J* = 8.1 Hz, 2H, Ar**H**), 7.05 (d, *J* = 8.1 Hz, 2H, Ar**H**), 3.16 (dd, *J* = 13.4 Hz, 9.6 Hz, 1H, CHBC**H**H'), 2.95 (dd, *J* = 13.4 Hz, 6.8 Hz,1H CHBCH**H**'), 2.69 (dd, *J* = 9.6 Hz, 6.8 Hz, 1H, C**H**BCHH'), 2.32 (s, 3H, C**H**$_3$), 1.15 (s, 6H, C(C**H**$_3$)(CH$_3$)'C(C**H**$_3$)(CH$_3$)')), 1.15 (s, 6H, C(CH$_3$)(C**H**$_3$)'C(CH$_3$)(C**H**$_3$)'); **^{13}C NMR (125 MHz, $CDCl_3$)**: 142.7 (Ar**C**), 138.6 (Ar**C**), 135.1 (Ar**C**), 128.7 (Ar**C**), 128.4 (Ar**C**), 128.3 (Ar**C**), 125.3 (Ar**C**), 83.4 (**C**-OB), 38.3 (**C**H$_2$), 24.6 (**C**H$_3$), 24.5 (**C**H$_3$), 21.0 (**C**H$_3$), peak not observed for **C** bound to B; **^{11}B NMR (160 MHz, $CDCl_3$)**: 32.6; **HRMS (EI$^+$)**: $C_{21}H_{27}BO_2$ requires 322.2104; found 322.2112.

(*R*)-2-(2-(6-Methoxynaphthalen-2-yl)-1-phenylethyl)-4,4,5,5-tetramethyl-1,3,2-dioxaborolane (compound **6aC**, Figure 3)

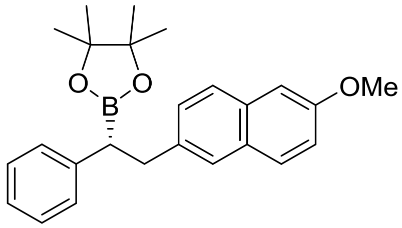

Diboronate **5a** (615 mg, 1.72 mmol, 1 eq, 98:2 e.r.), 2-bromo-6-methoxynaphthalene (611 mg, 2.58 mmol, 1.5 eq), $Pd(OAc)_2$ (19.3 mg, 0.086 mmol, 0.05 eq), SPhos (70.5 mg, 0.172 mmol, 0.1 eq) and K_2CO_3 (475 mg, 3.44 mmol, 2 eq) were weighed into a flask and placed under argon. THF (1.7 mL) followed by degassed water (3.4 mL) were added and the reaction mixture was heated at 60 °C for 24 h. The reaction mixture was cooled to room temperature, water (20 mL) and EtOAc (20 mL) were added and the layers were separated. The aqueous portion was extracted with EtOAc (2 × 20 mL) and the combined organics were washed with brine (20 mL), dried ($MgSO_4$), filtered and concentrated *in vacuo*. Purification by column chromatography (pentane/CH_2Cl_2 (1:1) → pentane/EtOAc (20:1)) gave **6aC** as a white solid (423 mg, 63%); e. r. 97:3. The e.r. was determined by oxidation to the alcohol (by **GP1**) followed by analysis by chiral SFC:

IB, 2mL/min, 100 bar, 50 °C, 1% → 40% MeOH over 10 min then 40% MeOH for 1 min; t_R 10.82 (major), 11.10 (minor).

^{1}H NMR (300 MHz, $CDCl_3$): 7.71-7.52 (m, 3H, Ar**H**), 7.37-7.21 (m, 5H, Ar**H**), 7.21-7.03 (m, 3H, Ar**H**), 3.92 (s, 3H, PhOC**H**$_3$), 3.33 (dd, *J* = 13.5 Hz, 10.0 Hz, 1H, CHBC**H**H'), 3.11 (dd, *J* = 13.5 Hz, 6.6 Hz, 1H, CHBCH**H'**), 2.80 (dd, *J* = 10.0 Hz, 6.6 Hz, 1H, C**H**BCHH'), 1.12 (s, 6H, C(C**H**$_3$)(CH$_3$)'C(C**H**$_3$)(CH$_3$)')), 1.12 (s, 6H, C(CH$_3$)(C**H**$_3$)'C(CH$_3$)(C**H**$_3$)')); **^{13}C NMR (125 MHz, $CDCl_3$)**: 157.2 (**C**), 142.8 (**C**), 137.2 (**C**), 133.1 (**C**), 129.12 (**C**), 129.05 (**C**H), 128.6 (**C**H), 128.5 (**C**H), 128.4 (**C**H), 126.9 (**C**H), 126.6 (**C**H), 125.5 (**C**H), 118.6 (**C**H), 105.8 (**C**H), 83.5 (**C**-OB), 55.4 (O**C**H$_3$), 39.0 (**C**H$_2$), 34.5 (br, **C**HB), 24.7 (C**C**H$_3$); **^{11}B NMR (128 MHz, $CDCl_3$)**: 33.0; **IR (film) cm^{-1}**: 2980w, 1981w, 1632w, 1602w; **HRMS (EI$^+$)**: $C_{25}H_{29}BO_3$ requires 388.2210; found 388.2209; $[\alpha]_D^{20}$ = –46.0 (*c* 1.0, $CHCl_3$); mp 101-103 °C.

(*R*)-2-(2-(3,5-Dimethylphenyl)-1-phenylethyl)-4,4,5,5-tetramethyl-1,3,2-dioxaborolane (compound **6aD**, Figure 3)

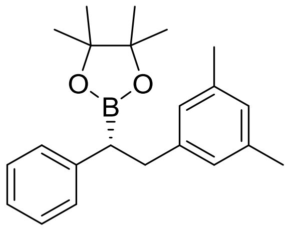

In a nitrogen filled glove box, diboronate **5a** (361 mg, 1.01 mmol, 1 eq, 93.5:6.5 e.r.), 1-bromo-3,5-dimethylbenzene (287.5 mg, 1.55 mmol, 1.5 eq), $Pd(OAc)_2$ (11.9 mg, 0.053 mmol, 0.05 eq), RuPhos (49.5 mg, 0.106 mmol, 0.1 eq) and K_2CO_3 (278.5 mg, 2.02 mmol, 2 eq) were weighed into a pressure tube and DME (10 mL) was added. The pressure tube was sealed with a septum and removed from the glove box and placed under a flow of argon. Degassed water (0.5 mL) was added, the reaction vessel was sealed and then heated at 80 °C for 16 h. The reaction mixture was cooled to room temperature, water (15 mL) and EtOAc (15 mL) were added and the layers were separated. The aqueous portion was extracted with EtOAc (2 × 15 mL) and the combined organics were washed with brine (15 mL), dried ($MgSO_4$), filtered and concentrated *in vacuo*. Purification by column chromatography (hexanes/CH_2Cl_2 (9:1 → 7:3)) gave **6aD** as a colourless oil (193 mg, 57%); e. r. 93:7. The e.r. was determined by oxidation to the alcohol (by **GP1**) followed by analysis by chiral SFC:
IB, 2mL/min, 100 bar, 50 °C, 1% → 40% MeOH over 10 min then 40% MeOH for 1 min; t_R 7.40 (major), 7.61 (minor).

^{1}H NMR (300 MHz, $CDCl_3$): 7.31-7.21 (m, 4H, Ar**H**, overlapping with residual $CHCl_3$), 7.15 (m, 1H, Ar**H**), 6.84 (s, 2H, Ar**H)**, 6.79 (s, 1H, Ar**H**), 3.09 (dd, *J* = 13.1 Hz, 10.6 Hz, 1H, CHBC**H**H'), 2.88 (dd, *J* = 13.1 Hz, 5.9 Hz, 1H, CHBCH**H'**), 2.65 (dd, *J* = 10.6 Hz, 5.9 Hz, 1H, C**H**BCHH'), 2.25 (s, 6H, 2 x ArC**H**$_3$), 1.11 (s, 6H, C(C**H**$_3$)(CH$_3$)'C(C**H**$_3$)(CH$_3$)'), 1.10 (s, 6H, C(CH$_3$)(C**H**$_3$)'C(CH$_3$)(C**H**$_3$)'); **^{13}C NMR (125 MHz, $CDCl_3$)**: 143.0 (**C**), 141.8 (**C**), 137.4 (**C**), 128.4 (**C**H), 127.4 (**C**H), 126.9 (**C**H), 125.5 (**C**H), 83.4 (**C**-OB), 39.0 (**C**H$_2$), 34.7 (br, **C**HB), 24.7 (C**C**H$_3$), 24.6 (C**C**H$_3$), 21.3 (Ar**C**H$_3$); **^{11}B NMR (128 MHz, $CDCl_3$)**: 32.6; **IR (film) cm^{-1}**: 2973w, 2918w, 1604w, 1493w; **HRMS (EI$^+$)**: $C_{22}H_{29}BO_3$ requires 336.2261; found 336.2261; $[\alpha]_D^{20}$ = –32.0 (*c* 1.0, $CHCl_3$).

(*R*)-4,4,5,5-Tetramethyl-2-(1-phenyl-2-(4-(trifluoromethoxy)phenyl)ethyl)-1,3,2-dioxaborolane (compound **6aE**, Figure 3)

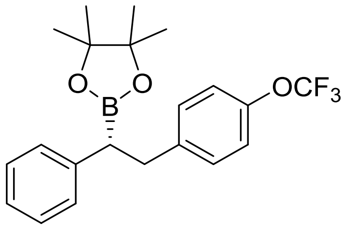

In a nitrogen filled glove box, diboronate **5a** (359 mg, 1.00 mmol, 1 eq, 93.5:6.5 e.r.), 1-bromo-4-(trifluoromethoxy)benzene (358 mg, 1.49 mmol, 1.5 eq), $Pd(OAc)_2$ (12.9 mg, 0.057 mmol, 0.06 eq), RuPhos (54.3 mg, 0.116 mmol, 0.12 eq) and K_2CO_3 (277 mg, 2.00 mmol, 2 eq) were weighed into a pressure tube and DME (10 mL) was added. The pressure tube was sealed with a septum and removed from the glove box and placed under a flow of argon. Degassed water (0.5 mL) was added, the reaction vessel was sealed and then heated at 80 °C for 16 h. The reaction mixture was cooled to room temperature, water (15 mL) and EtOAc (15 mL) were added and the layers were separated. The aqueous portion was extracted with EtOAc (2 × 15 mL) and the combined organics were washed with brine (15 mL), dried ($MgSO_4$), filtered and concentrated *in vacuo*. Purification by column chromatography (hexanes/CH_2Cl_2 (9:1 → 7:3)) gave **6aE** as a pale yellow solid (202 mg, 51%); e. r. 94:6. The e.r. was determined by oxidation to the alcohol (by **GP1**) followed by analysis by chiral SFC:
IB, 2mL/min, 100 bar, 40 °C, 1% → 30% MeOH over 10 min then 30% MeOH for 3 min; t_R 8.53 (major), 8.70 (minor).

^{1}H NMR (400 MHz, $CDCl_3$): 7.28-7.12 (m, 7H, Ar**H**), 7.09-7.04 (m, 2H, Ar**H**), 3.13 (dd, J = 13.6 Hz, 9.7 Hz, 1H, CHBC**H**H'), 2.96 (dd, J = 13.6 Hz, 7.0 Hz, 1H, CHBCH**H'**), 2.65 (dd, J = 9.7 Hz, 7.0 Hz, 1H, C**H**BCHH'), 1.103 (s, 6H, C(C**H**$_3$)(CH$_3$)'C(C**H**$_3$)(CH$_3$)')), 1.100 (s, 6H, C(CH$_3$)(C**H**$_3$)'C(CH$_3$)(C**H**$_3$)')); **^{13}C NMR (75 MHz, $CDCl_3$)**: 147.5 (**C**), 142.2 (**C**), 140.7 (**C**), 130.3 (**C**H), 128.6 (**C**H), 128.5 (**C**H), 125.7 (**C**H), 120.8 (**C**H), 120.7 (q, J = 256 Hz, **C**F$_3$), 83.7 (**C**-OB), 38.3 (**C**H$_2$), 34.5 (br, **C**HB), 24.7 (**C**H$_3$), 24.6 (**C**H$_3$); **^{11}B NMR (128 MHz, $CDCl_3$)**: 32.8; **^{19}F NMR (377 MHz, $CDCl_3$)**: –58.5; **IR (film) cm^{-1}**: 2979w, 2932w, 1600w, 1508w; **HRMS (EI$^+$)**: $C_{21}H_{24}BO_3F_3$ requires 392.1771; found 392.1771; $[\alpha]_D^{20}$ = –34.7 (*c* 1.5, $CHCl_3$); mp 39.9-41.3 °C.

2-(2-(6-methoxynaphthalen-2-yl)-1-(p-tolyl)ethyl)-4,4,5,5-tetramethyl-1,3,2-dioxaborolane (compound **6bC**, Figure 3)

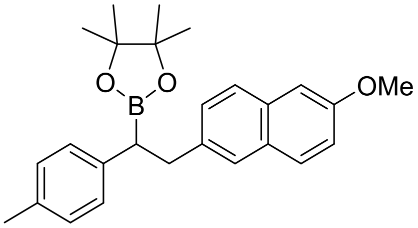

In a nitrogen filled glovebox, **5b** (369.0mg, 0.99 mmol, 1 eq), 6-methoxy-2-bromonapthalene (353.1 mg, 1.50 mmol, 1.5 eq), $Pd(OAc)_2$ (11.4 mg, 0.0509 mmol, 0.05 eq), SPhos (42.2 mg, 0.10 mmol, 0.1 eq), K_2CO_3 (276.4 mg, 2.0 mmol, 2 eq) were taken up in THF (1 mL) in a 15 mL pressure tube. The reaction was sealed with a septum and removed from the glove box and placed under a flow of argon. Degassed water (2.0 mL) was added and the septum was replaced with a screw cap. The reaction mixture was stirred at 60 °C for 24 h. The reaction mixture was then cooled to room temperature, diluted with water and ethyl acetate, and the aqueous layer washed three times with ethyl acetate. The combined organic layers were dried over Na_2SO_4 filtered and concentrated *in vacuo.* Purification by column chromatography (hexanes/CH_2Cl_2 8:2 → 6.5:3.5) gave **6bC** as a white solid (239 mg, 60%).

^{1}H NMR (400 MHz, $CDCl_3$): 7.65-7.59 (m, 3H, Ar**H**), 7.32 (dd, *J* = 8.4 Hz, 1.6 Hz, 1H, Ar**H**), 7.18 (d, *J* = 8.0 Hz, 2H, Ar**H**), 7.11-7.07 (m 4H, Ar**H**), 3.91 (s, 3H, OC**H**$_3$), 3.28 (dd, *J* = 13.5 Hz, 10.0 Hz, 1H, CHBC**H**H'), 3.07 (dd, *J* = 13.5 Hz, 6.5 Hz, 1H. CHBCH**H'**), 2.75 (dd, *J* = 10.0 Hz, 6.6 Hz, 1H, C**H**BCHH'), 2.32 (s, 3H, ArC**H**$_3$) 1.10 (s, 6H, C(C**H**$_3$)(CH$_3$)'C(C**H**$_3$)(CH$_3$)')), 1.09 (s, 6H, C(CH$_3$)(C**H**$_3$)'C(CH$_3$)(C**H**$_3$)')); **^{13}C NMR (100 MHz, $CDCl_3$)**: 157.2 (**C**), 139.7 (**C**), 137.3 (**C**), 134.9 (**C**), 133.1 (**C**), 129.2 (**C**H), 129.13 (**C**H), 129.06 (**C**H), 128.5 (**C**H), 128.4 (**C**H), 126.9 (**C**H), 126.6 (**C**H), 118.5 (**C**H), 105.8 (**C**H), 83.5 (**C**-OB), 55.4 (O**C**H$_3$), 39.2 (**C**H$_2$), 34.1 (br, **C**HB), 24.7 (**C**H$_3$), 21.1 (Ar**C**H$_3$); **^{11}B NMR (128 MHz, $CDCl_3$)**: 32.3; **IR (film) cm^{-1}**: 3067w, 2920m, 1605m, 1325s, 1135s; **HRMS (EI$^+$)**: $C_{26}H_{21}BO_3$ requires 402.2371; found 402.2379; mp: 55.6-56.9 °C.

2-(1,2-Di-*p*-tolylethyl)-4,4,5,5-tetramethyl-1,3,2-dioxaborolane (compound **6bB**, Figure 3)

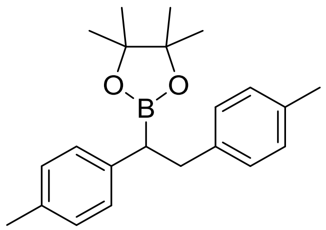

In a nitrogen filled glovebox, diboronate **5b** (20.8 mg, 0.056 mmol, 1 eq), 4-bromotoluene (11.5 mg, 0.067 mmol, 1.2 eq), $Pd(OAc)_2$ (1.2 mg, 0.0053 mmol, 0.1 eq), RuPhos (6.5 mg, 0.014 mmol, 0.25 eq) and K_2CO_3 (14.7 mg, 0.106 mmol, 1.9 eq) were weighed into a vial and THF (1.1 mL) was added. The reaction was sealed with a septum and removed from the glove box and placed under a flow of argon. Degassed water (0.06 mL) was added and the septum was replaced with a Teflon cap. The reaction mixture was sonicated for about 2 minutes before being stirred at 80 °C for 15 h. The reaction mixture was cooled and filtered through a plug of silica, eluted with EtOAc and concentrated *in vacuo*. Purification by column chromatography (hexane/EtOAc (97:3)) gave **6bB** (13.9 mg, 74% yield).

^{1}H NMR (400 MHz, $CDCl_3$): 7.19-6.97 (m, 8H), 3.15-3.07 (dd, *J* = 13.0 Hz, 9.7 Hz 1H, CHBCH**H**'), 2.91 (dd, *J* = 13.0 Hz, 7.3 Hz, 1H, CHBCH**H**'), 2.63 (dd, *J* = 9.7 Hz, 7.3 Hz, 1H, C**H**BCHH'), 2.31 (s, 3H, ArC**H**$_3$), 2.30 (s, 3H, ArC**H**$_3$'), 1.13 (s, 12H, C(C**H**$_3$)$_2$C(C**H**$_3$)$_2$); **^{13}C NMR (100 MHz, $CDCl_3$)**: 139.6 (**C**), 138.7 (**C**), 135.0 (**C**), 134.6 (**C**), 129.0 (**C**H), 128.7 (**C**H), 128.6 (**C**H), 128.2 (**C**H), 83.3 (**C**-OB), 38.5 (**C**H$_2$), 24.6 (**C**H$_3$), 24.5 (**C**H$_3$), 21.0 (**C**H$_3$), peak not observed for **C** bound to B; **^{11}B NMR (128 MHz, $CDCl_3$)**: 33.3.

2-(1-(4-Methoxyphenyl)-2-(*p*-tolyl)ethyl)-4,4,5,5-tetramethyl-1,3,2-dioxaborolane (compound **6fB**, Figure 3)

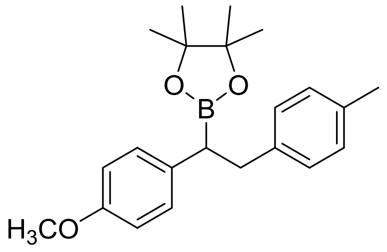

In a nitrogen filled glovebox, diboronate **5f** (21.7 mg, 0.056 mmol, 1 eq), 4-bromotoluene (11.5 mg, 0.067 mmol, 1.2 eq), $Pd(OAc)_2$ (1.2 mg, 0.0053 mmol, 0.1 eq), RuPhos (6.5 mg, 0.014 mmol, 0.25 eq), K_2CO_3 (14.7 mg, 0.106 mmol, 1.9 eq) were weighed into a vial and THF (1.1 mL) was added. The reaction was sealed with a septum and removed from the glove box and placed under a flow of argon. Degassed water (0.06 mL) was added and the septum was replaced with a Teflon cap. The reaction mixture was sonicated for about 2 minutes before being stirred at 80 °C for 15 h. The reaction mixture was cooled and filtered through a plug of silica, eluted with EtOAc and concentrated *in vacuo*. Purification by column chromatography (hexane/EtOAc (97:3)) gave **6fB** (13.2 mg, 67% yield).

^{1}H NMR (400 MHz, $CDCl_3$): 7.16 (d, *J* = 8.5, 2H, Ar**H**), 7.11-7.00 (m, 4H), 6.81 (d, *J* = 8.9, 2H, Ar**H**), 3.79 (s, 3H, OC**H**$_3$), 3.15-3.03 (m, 1H, CHBC**H**H'), 2.89 (dd, *J* = 13.0, 7.3 Hz, 1H, CHBCH**H**'), 2.60 (dd, *J* = 9.7, 7.3 Hz, 1H, C**H**BCHH'), 2.29 (s, 3H, ArC**H**$_3$), 1.13 (s, 12H, C(C**H**$_3$)$_2$C(C**H**$_3$)$_2$); **^{13}C NMR (100 MHz, $CDCl_3$)**: 157.4 (**C**), 138.7 (**C**), 134.9 (**C**), 134.7 (**C**), 129.3 (**C**H), 128.70 (**C**H), 128.65 (**C**H), 113.7 (**C**H), 83.3 (**C**-OB), 55.2 (O**C**H$_3$), 38.6 (**C**H$_2$), 24.57 (**C**H$_3$), 24.53 (**C**H$_3$), 21.0 (**C**H$_3$), peak not observed for **C** bound to B; **^{11}B NMR (128 MHz, $CDCl_3$)**: 33.3; **HRMS (EI$^+$)**: $C_{22}H_{29}BO_3$ requires 352.2210; found 352.2223.

2-(1-(Benzo[d][1,3]dioxol-5-yl)-2-phenylethyl)-4,4,5,5-tetramethyl-1,3,2-dioxaborolane (compound **6gA**, Figure 3)

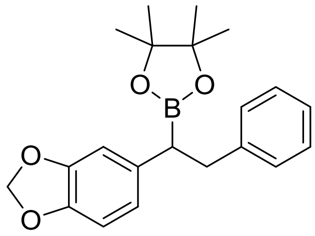

In a nitrogen filled glove box, diboronate **5g** (125 mg, 0.311 mmol, 1 eq), bromobenzene (73 mg, 0.466 mmol, 1.5 eq), $Pd(OAc)_2$ (3.5 mg, 0.016 mmol, 0.05 eq), SPhos (12.8 mg, 0.031 mmol, 0.1 eq) and K_2CO_3 (86 mg, 0.62 mmol, 2 eq) were weighed into a pressure tube and THF (0.3 mL) was added. The pressure tube was sealed with a septum and removed from the glove box and placed under a flow of argon. Degassed water (0.6 mL) was added, the reaction vessel was sealed and then heated at 60 °C for 24 h. The reaction mixture was cooled to room temperature, water (10 mL) and EtOAc (10 mL) were added and the layers were separated. The aqueous portion was extracted with EtOAc (2 × 10 mL) and the combined organics were washed with brine (10 mL), dried ($MgSO_4$), and concentrated *in vacuo*. Purification by column chromatography (pentane/EtOAc (30:1)) gave **6gA** as a colourless oil (72 mg, 66%):

^{1}H NMR (500 MHz, $CDCl_3$): 7.30-7.08 (m, 5H, Ar**H**), 6.79 (d, *J* = 1.1 Hz, 1H, Ar**H**), 6.73-6.62 (m, 2H, Ar**H**), 3.10 (dd, *J* = 13.4 Hz, 9.5 Hz, 1H, CHBC**H**H'), 2.92 (dd, *J* = 13.4 Hz, 7.2 Hz, 1H, CHBCH**H'**), 2.61 (dd, *J* 9.5 Hz, 7.2 Hz, 1H, C**H**B), 1.14 (s, 6H, C(C**H**$_3$)(CH$_3$)'C(C**H**$_3$)(CH$_3$)'), 1.13 (s, 6H, C(CH$_3$)(C**H**$_3$)'C(CH$_3$)(C**H**$_3$)'); **^{13}C NMR (125 MHz, $CDCl_3$)**: 147.6 (**C**), 145.4 (**C**), 141.7 (**C**), 136.5 (**C**), 129.0 (**C**H), 128.1 (**C**H), 125.9 (**C**H), 121.4 (**C**H), 109.0 (**C**H), 108.3 (**C**H), 100.8 (**C**H$_2$), 83.5 (**C**-OB), 39.3 (**C**H$_2$), 34.1 (br, **C**HB), 24.71 (**C**H$_3$), 24.64 (**C**H$_3$); **^{11}B NMR (160 MHz, $CDCl_3$)**: 34.0; **IR (film) cm^{-1}**: 2979w, 2895w, 1506w, 1487w; **LRMS (EI$^+$)**: $C_{21}H_{25}BO_4$ requires 352.2; found 352.2.

(*R*)-2-(1-(Benzo[d][1,3]dioxol-5-yl)-2-(4-(trifluoromethoxy)phenyl)ethyl)-4,4,5,5-tetramethyl-1,3,2-dioxaborolane (compound **6gE**, Figure 3)

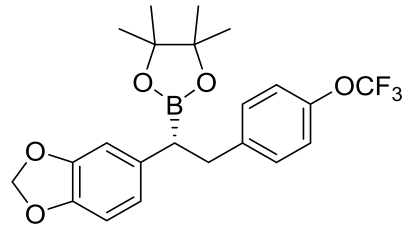

In a nitrogen filled glove box, diboronate **5g** (121 mg, 0.301 mmol, 1 eq, e.r. 93.5:6.5), 1-bromo-4-(trifluoromethoxy)benzene (109 mg, 0.451 mmol, 1.5 eq), $Pd(OAc)_2$ (3.4 mg, 0.015 mmol, 0.05 eq), SPhos (12.4 mg, 0.030 mmol, 0.1 eq) and K_2CO_3 (83 mg, 0.602 mmol, 2 eq) were weighed into a pressure tube and THF (0.3 mL) was added. The pressure tube was sealed with a septum and removed from the glove box and placed under a flow of argon. Degassed water (0.6 mL) was added, the reaction vessel was sealed and then heated at 60 °C for 24 h. The reaction mixture was cooled to room temperature, water (10 mL) and EtOAc (10 mL) were added and the layers were separated. The aqueous portion was extracted with EtOAc (2 × 10 mL) and the combined organics were washed with brine (10 mL), dried ($MgSO_4$), and concentrated *in vacuo*. Purification by column chromatography (pentane/EtOAc (30:1)) gave **6gE** as a colourless oil (69 mg, 53%), e.r. was determined over two steps (see compound **7hEj**).

^{1}H NMR (400 MHz, $CDCl_3$): 7.18 (d, *J* = 8.4 Hz, 2H, Ar**H**), 7.07 (d, *J* = 8.4 Hz, 2H, Ar**H**), 6.75 (s, 1H, Ar**H**), 6.70 (d, *J* = 8.0 Hz, 1H, Ar**H**), 6.64 (d, *J* = 8.0 Hz, 1H, Ar**H**), 5.91 (s, 2H, OC**H**$_2$O), 3.07 (dd, *J* = 13.5 Hz, 9.6 Hz, 1H, CHBC**H**H'), 2.91 (dd, *J* = 13.5 Hz, 7.3 Hz, 1H, CHBCH**H'**), 2.56 (dd, *J* = 9.6 Hz, 7.3 Hz, 1H, C**H**B), 1.120 (s, 6H, C(C**H**$_3$)(CH$_3$)'C(C**H**$_3$)(CH$_3$)'), 1.117 (s, 6H, C(CH$_3$)(C**H**$_3$)'C(CH$_3$)(C**H**$_3$)'); **^{13}C NMR (125 MHz, $CDCl_3$)**: 147.8 (**C**), 147.6 (q, *J* = 1.7 Hz, **C**OCF$_3$), 145.6 (**C**), 140.6 (**C**), 136.0 (**C**), 130.3 (**C**H), 121.4 (**C**H), 120.8 (**C**H), 120.7 (q, *J* = 256 Hz, **C**F$_3$), 109.0 (**C**H), 108.4 (**C**H), 100.9 (**C**H$_2$), 83.7 (**C**-OB), 38.7 (**C**H$_2$), 34.2 (br, CHB), 24.7 (**C**H$_3$), 24.6 (**C**H$_3$); **^{11}B NMR (128 MHz, $CDCl_3$)**: 32.4; **^{19}F NMR (377 MHz, $CDCl_3$)**: –58.5; **IR (film) cm^{-1}**: 2978w, 2930w, 2894w, 1503w. **LRMS (EI$^+$)**: $C_{22}H_{24}BF_3O_5$ requires 436.2; found 436.1.

4,4,5,5-tetramethyl-2-(1-(3-phenoxyphenyl)-2-phenylethyl)-1,3,2-dioxaborolane (compound **6hA**, Figure 3)

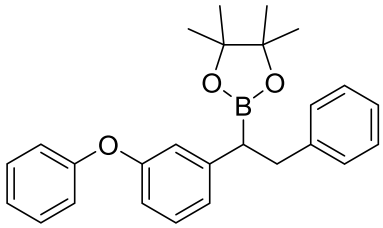

In a nitrogen filled glovebox, diboronate **5h** (151 mg, 0.34 mmol, 1 eq), bromobenzene (80.9 mg, 0.52 mmol, 1.5 eq), $Pd(OAc)_2$ (4.00 mg, 0.018 mmol, 0.05 eq), SPhos (14.01 mg, 0.034 mmol, 0.10 eq) and K_2CO_3 (94.20 mg, 0.68 mmol, 2.0 eq) were taken up in THF (0.34 mL) in a 1 dram vial. The reaction was sealed with a PTFE septa screw cap and removed from the glove box and placed under a flow of argon. Degassed water (0.68 mL) was added and the reaction mixture was stirred at 60 °C for 24 h. The reaction mixture was then cooled to room temperature, diluted with water and ethyl acetate, and the aqueous layer washed three times with ethyl acetate. The combined organic layers were dried over Na_2SO_4, filtered and concentrated *in vacuo.* Purification by column chromatography (hexanes/ethyl acetate 99:1 → 96:4) gave **6hA** as a colorless oil (48 mg, 36%).

^{1}H NMR (400 MHz, $CDCl_3$): 7.31 (t, *J* = 8.0 Hz, 2H, Ar**H**), 7.22 (t, *J*= 7.0 Hz, 3H, Ar**H**), 7.16 (d, *J* = 7.3 Hz, 3H, Ar**H**), 7.07 (t, *J* = 7.4 Hz, 1H, Ar**H**), 6.99 (d, *J* = 7.6 Hz, 1H, Ar**H**), 6.94 (d, *J* = 7.8 Hz, 2H, Ar**H**), 6.91 (m, 1H, Ar**H**), 6.81 (dd, *J* = 7.7 Hz, 1.9 Hz, 1H, Ar**H**), 3.13 (dd, *J* = 13.5 Hz, 9.2 Hz, 1H, CHC**H**H'), 2.95 (dd, *J* = 13.5 Hz, 7.4 Hz0, 1H, CHCH**H'**), 2.67 (m, 2H, C**H**CH'), 1.12 (s, 6H, C(C**H**$_3$)(CH$_3$)'C(C**H**$_3$)(CH$_3$)')), 1.11 (s, 6H, C(CH$_3$)(C**H**$_3$)'C(CH$_3$)(C**H**$_3$)')) **^{13}C NMR (100 MHz, $CDCl_3$)**: 157.78 (**C**), 157.08 (**C**), 144.77 (**C**), 141.61 (**C**), 129.73 (**C**H), 129.65 (**C**H), 129.01 (**C**H), 128.18 (**C**H), 125.92 (**C**H), 123.84 (**C**H), 122.92 (**C**H), 119.46 (**C**H), 118.66 (**C**H), 116.47 (**C**H), 83.61 (**C**-OB), 38.8 (**C**H$_2$), 34.50 (br, **C**H), 24.72 (**C**H$_3$), 24.66 (**C**H$_3$) **^{11}B NMR (128 MHz, $CDCl_3$)**: 31.58 ; **IR (film) cm^{-1}**: 3061w, 2976m, 1579m, 1482s, 1359m, 1327m, 1243s, 1214m, 1140m; **HRMS (EI^{+})**: $C_{26}H_{29}BO_3$ requires 400.2215; found 400.2219.

Determination of NMR yield of 4,4,5,5-tetramethyl-2-(1-(3-phenoxyphenyl)-2-phenylethyl)-1,3,2-dioxaborolane (compound **6hA**, Figure 3)

In a nitrogen filled glovebox, diboronate **5h** (60.75 mg, 0.135 mmol, 1 eq), bromobenzene (37.3 mg, 0.24 mmol, 1.8 eq), $Pd(OAc)_2$ (1.57 mg, 0.0070 mmol, 0.05 eq), SPhos (5.53 mg, 0.013 mmol, 0.10 eq) and K_2CO_3 (37.30 mg, 0.27 mmol, 2.0 eq) were taken up in THF (0.14 mL) in a 1 dram vial. The reaction was sealed with a PTFE septa screw cap, removed from the glove box and placed under a flow of argon. Degassed water (0.28 mL) was added and the reaction mixture was stirred at 60 °C for 24 h. The reaction mixture was then cooled to room temperature, diluted with 1 mL $CHCl_3$ and internal standard (200 µL of a solution of 26.2 mg 1,3,5-trimethoxybenzene in 1 mL $CHCl_3$) was added. An aliquot of the reaction was filtered through a pipette of silica gel with $CHCl_3$, concentrated *in vacuo* and subjected to ^{1}H NMR analysis to determine the yield (83%).

(*R*)-3-(1,2-Diphenylethyl)pyridine (compound **7aAi**, Figure 3)

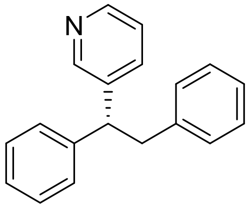

In a nitrogen filled glove box, boronic ester **6aA** (20 mg, 0.065 mmol, 1 eq, e.r. 92:8 (assumed based on e.r. of diboronate **5a**)), 3-iodopyridine (19.9 mg, 0.097 mmol, 1.5 eq), $Pd(dba)_2$ (3.0 mg, 0.005 mmol, 0.08 eq), PPh_3 (10.9 mg, 0.042 mmol, 0.64 eq) and Ag_2O (25.7 mg, 0.097 mmol, 1.5 eq) were weighed into a 1 dram vial and DME (1.3 mL) was added. The reaction vessel was sealed, removed from the glove box and heated at 70 °C for 16 h. The reaction mixture was cooled and filtered through a plug of silica, eluted with EtOAc (30 mL) and concentrated *in vacuo*. Purification by column chromatography (hexane/EtOAc (90:10 → 75:25)) gave **7aAi** as an oil (12.8 mg, 76%); e. r. 88:12, 90% e.s. (over 2 steps from 92:8 e.r. batch of diboronate **5a**). The e.r. was determined by chiral SFC:

AD-H, 2mL/min, 10% MeOH, 200 bar, T = 50 °C; t_R 6.8 (major), 8.1 (minor).

^{1}H NMR (400 MHz, $CDCl_3$): 8.43 (br s, 2H, Ar**H**), 7.46 (d, *J* = 7.7 Hz, 1H, Ar**H**), 7.35-7.07 (m, 9H, Ar**H**), 7.05-6.92 (m, 2 H, Ar**H**), 4.24 (t, *J* = 7.7 Hz, 1 H, C**H**CHH'), 3.47-3.23 (m, 2 H, CHC**HH'**); **^{13}C NMR (100 MHz, $CDCl_3$)**: 149.7 (Ar**C**), 147.6 (Ar**C**), 143.2 (Ar**C**), 139.4 (Ar**C**), 135.4 (Ar**C**), 129.0 (Ar**C**), 128.6 (Ar**C**), 128.2 (Ar**C**), 127.9 (Ar**C**), 126.6 (Ar**C**), 126.2 (Ar**C**), 50.7 (**C**H), 41.8 (**C**H_2); **IR (film) cm^{-1}**: 3027w, 2925w, 1602w, 1026w, 698w; **HRMS (EI$^+$)**: $C_{19}H_{17}N$ requires 259.1361; found 259.1368.

(*R*)-1-(4-(1,2-Diphenylethyl)phenyl)ethanone (compound **7aAj**, Figure 3)

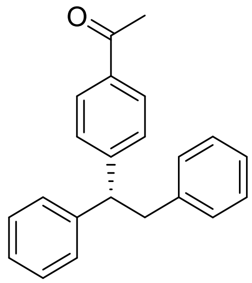

In a nitrogen filled glove box, boronic ester **6aA** (20 mg, 0.065 mmol, 1 eq, e.r. 92:8 (assumed based on e.r. of diboronate **5a**)), 4-iodoacetophenone (23.9 mg, 0.097 mmol, 1.5 eq), $Pd(dba)_2$ (3.0 mg, 0.005 mmol, 0.08 eq), PPh_3 (10.9 mg, 0.042 mmol, 0.64 eq) and Ag_2O (25.7 mg, 0.097 mmol, 1.5 eq) were weighed into a 1 dram vial and DME (1.3 mL) was added. The reaction vessel was sealed, removed from the glove box and heated at 70 °C for 16 h. The reaction mixture was cooled and filtered through a plug of silica, eluted with EtOAc (30 mL) and concentrated *in vacuo*. Purification by column chromatography (hexane/EtOAc (97.5:2.5)) gave **7aAj** (14.3 mg, 73%); e. r. 86.5:13.5, 87% e.s. (over 2 steps from 92:8 e.r. batch of diboronate **5a**). The e.r. was determined by chiral SFC:

AD-H, 2mL/min, 5% MeOH, 200 bar, T = 50 °C; t_R 11.8 (minor), 13.8 (major).

^{1}H NMR (400 MHz, $CDCl_3$): 7.87-7.78 (m, 2H), 7.33-7.06 (m, 10H), 7.03-6.95 (m, 2H), 4.29 (t, *J* = 8.0 Hz, 1H, C**H**CHH'), 3.44-3.29 (m, 2H, CHC**HH'**), 2.53 (s, 3H, C**H**$_3$); **^{13}C NMR (100 MHz, $CDCl_3$)**: 197.7 (**C**=O), 145.0 (**C**), 143.6 (**C**), 139.6 (**C**), 135.3 (**C**), 129.0 (**C**H), 128.52 (**C**H), 128.50 (**C**H), 128.3 (**C**H), 128.2 (**C**H), 127.9 (**C**H), 126.5 (**C**H), 126.1 (**C**H), 53.1 (**C**H), 41.7 (**C**H_2), 26.5 (**C**H_3); **IR (film) cm^{-1}**: 3027w, 2924w, 1679w, 1601w, 1266w, 697w; **HRMS (EI$^+$)**: $C_{22}H_{20}O$ requires 300.1514; found 300.1523.

(*R*)-1-Methoxy-4-(1-phenyl-2-(*p*-tolyl)ethyl)benzene (compound **7aBf**, Figure 3)

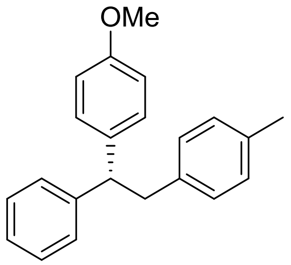

In a nitrogen filled glove box, boronic ester **6aB** (20 mg, 0.062 mmol, 1 eq, e.r. 92:8), 4-iodoanisole (21.9 mg, 0.093 mmol, 1.5 eq), $Pd(dba)_2$ (2.9 mg, 0.005 mmol, 0.08 eq), PPh_3 (10.4 mg, 0.039 mmol, 0.64 eq) and Ag_2O (24.5 mg, 0.093 mmol, 1.5 eq) were weighed into a 1 dram vial and DME (1.3 mL) was added. The reaction vessel was sealed, removed from the glove box and heated at 70 °C for 16 h. The reaction mixture was cooled and filtered through a plug of silica, eluted with EtOAc (30 mL) and concentrated *in vacuo*. Purification by column chromatography (pentane/CH_2Cl_2 (85:15)) gave **7aBf** (15 mg, 80%); e. r. 12:88, 90% e.s. (over 2 steps from 92:8 e.r. batch of diboronate **5a**). The e.r. was determined by chiral SFC:

IB, 1mL/min, 1% MeOH, 150 bar, T = 50 °C; t_R 32.7 (minor), 34.4 (major).

^{1}H NMR (400 MHz, $CDCl_3$): 7.34-7.10 (m, 7H, Ar**H**), 7.00 (d, *J* = 7.6 Hz, 2H, Ar**H**), 6.92 (d, *J* = 7.6 Hz, 2H, Ar**H**), 6.81 (d, *J* = 8.0 Hz, 2H, Ar**H**), 4.19 (t, *J* = 8.0 Hz, 1H C**H**CHH'), 3.78 (s, 3 H, OC**H**$_3$), 3.31 (d, *J* = 8.0 Hz, 2H, CHC**HH**'), 2.29 (s, 3H, ArC**H**$_3$); **^{13}C NMR (100 MHz, $CDCl_3$)**: 157.8 (**C**), 145.0 (**C**), 137.2 (**C**), 136.7 (**C**), 135.2 (**C**), 128.9 (**C**H), 128.7 (**C**H), 128.3 (**C**H), 127.9 (**C**H), 126.0 (**C**H), 113.7 (**C**H), 55.2 (O**C**H$_3$), 52.2 (**C**H), 41.8 (**C**H$_2$), 21.0 (**C**H$_3$); **IR (film) cm^{-1}**: 3025w, 2928w, 1609w, 1508w, 1244w, 1178w, 696w; **HRMS (EI$^+$)**: $C_{22}H_{22}O$ requires 302.1671; found 302.1675.

(*R*)-1-(4-(1-Phenyl-2-(*p*-tolyl)ethyl)phenyl)ethanone (compound **7aBj**, Figure 3)

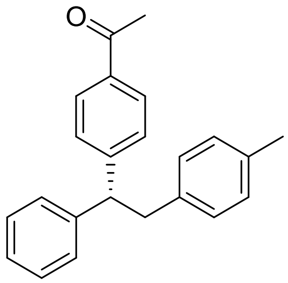

In a nitrogen filled glove box, boronic ester **6aB** (20 mg, 0.062 mmol, 1 eq, e.r. 92:8), 4-iodoacetophenone (22.9 mg, 0.093 mmol, 1.5 eq), $Pd(dba)_2$ (2.9 mg, 0.005 mmol, 0.08 eq), PPh_3 (10.4 mg, 0.039 mmol, 0.64 eq) and Ag_2O (24.5 mg, 0.093 mmol, 1.5 eq) were weighed into a 1 dram vial and DME (1.3 mL) was added. The reaction vessel was sealed, removed from the glove box and heated at 70 °C for 16 h. The reaction mixture was cooled and filtered through a plug of silica, eluted with EtOAc (30 mL) and concentrated *in vacuo*. Purification by column chromatography (pentane/EtOAc (96.5:3.5)) gave **7aBj** (13.0 mg, 66%); e. r. 12:88, 90% e.s. (over steps from 92:8 e.r. batch of diboronate **5a**). The e.r. was determined by chiral SFC:

AD-H, 2mL/min, 5% MeOH, 200 bar, T = 50 °C; t_R 11.8 (minor), 13.4 (major).

^{1}H NMR (400 MHz, $CDCl_3$): 7.83 (d, *J* = 8.0 Hz, 2H, Ar**H**), 7.33-7.15 (m, 7H, Ar**H**), 6.97 (d, *J* = 8.1 Hz, 2H, Ar**H**), 6.89 (d, *J* = 8.1 Hz, 2H, Ar**H**), 4.28 (t, *J* = 7.6 Hz, 1H, C**H**CHH'), 3.41-3.27 (m, 2H, CHC**HH'**), 2.55 (s, 3H, COC$\mathbf{H}_3$), 2.26 (s, 3H, ArC$\mathbf{H}_3$); **^{13}C NMR (100 MHz, $CDCl_3$)**: 197.8 (**C**=O), 150.1 (**C**), 143.7 (**C**), 136.5 (**C**), 135.5 (**C**), 135.2 (**C**), 128.9 (**C**H), 128.8 (**C**H), 128.5 (**C**H), 128.3 (**C**H), 127.9 (**C**H), 126.5 (**C**H), 53.1 (**C**H), 41.3 (**C**H_2), 26.5 (**C**H_3), 21.0 (**C**H_3); **IR (film) cm^{-1}**: 3025w, 2922w, 1679w, 1604w, 1266w, 699w; **HRMS (EI$^+$)**: $C_{23}H_{22}O$ requires 314.1671; found 314.1675.

(*R*)-3-(2-(3,5-dimethylphenyl)-1-phenylethyl)pyridine (compound **7aDi**, Figure 3)

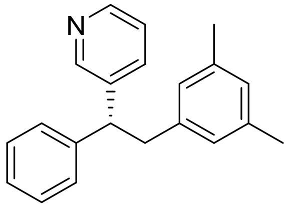

In a nitrogen filled glove box, boronic ester **6aD** (132 mg, 0.393 mmol, 1 eq, 93:7 e.r.), 3-iodopyridine (121 mg, 0.59 mmol, 1.5 eq), $Pd(dba)_2$ (18.1 mg, 0.0314 mmol, 0.08 eq), PPh_3 (49.4 mg, 0.188 mmol, 0.48 eq) and Ag_2O (136 mg, 0.59 mmol, 1.5 eq) were weighed into a pressure tube and DME (7.9 mL) was added. The reaction vessel was sealed, removed from the glove box and heated at 72 °C for 16 h. The reaction mixture was cooled and filtered through a plug of silica, eluted with EtOAc (30 mL) and concentrated *in vacuo*. Purification by column chromatography (pentane/EtOAc (7:1)) gave **7aDi** as a pale yellow oil (57 mg, 51%); e. r. 85.5:14.5, 82% e.s. (over 2 steps from 93.5:6.5 e.r. batch of diboronate **5a**). The e.r. was determined by analysis by chiral SFC:

IA, 2mL/min, 5% MeOH, 100 bar, T = 45 °C; t_R 16.0 (minor), 17.0 (major).

^{1}H NMR (500 MHz, $CDCl_3$): 8.51-8.34 (m, 2H, Ar**H**), 7.49 (d, *J* = 7.9 Hz, 1H, Ar**H**), 7.33-7.26 (m, 2H, Ar**H**), 7.26-7.12 (m, 4H, Ar**H**), 6.78 (s, 1H, Ar**H**), 6.63 (s, 2H, Ar**H**), 4.26 (dd. *J* = 8.7 Hz, 7.0 Hz, 1H, C**H**CHH'), 3.34 (dd, *J* = 13.6 Hz, 7.0 Hz, 1H, CHC**H**H'), 3.25 (dd, *J* = 13.6 Hz, 8.7 Hz, 1H, CHCH**H**'), 2.21 (s, 6H, 2 × CH_3); **^{13}C NMR (125 MHz, $CDCl_3$)**: 149.9 (**C**H), 147.6 (**C**H), 143.6 (**C**), 139.9 (**C**), 139.3 (**C**), 137.7 (**C**), 135.5 (**C**H), 128.7 (**C**H), 128.0 (**C**H), 127.9 (**C**H), 127.0 (**C**H), 126.7 (**C**), 123.3 (**C**H), 50.7 (**C**H), 41.8 (**C**H_2), 21.3 (**C**H_3); **IR (film) cm^{-1}**: 3025w, 2917w, 1603w, 1574w; **HRMS (EI$^+$)**: $C_{21}H_{21}N$ requires 287.1674; found 287.1679; $[\alpha]_D^{20}$ = +20.0 (*c* 0.5, $CHCl_3$).

(*R*)-1-(1-Phenyl-2-(4-(trifluoromethoxy)phenyl)ethyl)-4-(trifluoromethyl)benzene (compound **7aEk**, Figure 3)

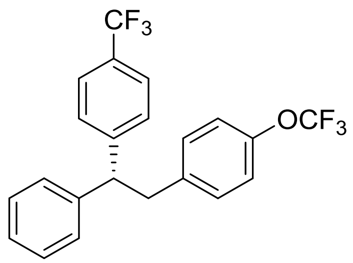

In a nitrogen filled glove box, boronic ester **6aE** (73 mg, 0.186 mmol, 1 eq, e.r. 94:6), 4-iodobenzotrifluoride (76 mg, 0.279 mmol, 1.5 eq), $Pd(dba)_2$ (8.6 mg, 0.0149 mmol, 0.08 eq), PPh_3 (23.4 mg, 0.089 mmol, 0.48 eq) and Ag_2O (65 mg, 0.279 mmol, 1.5 eq) were weighed into a pressure tube and DME (3.7 mL) was added. The reaction vessel was sealed, removed from the glove box and heated at 72 °C for 16 h. The reaction mixture was cooled and filtered through a plug of silica, eluted with EtOAc (30 mL) and concentrated *in vacuo*. Purification by column chromatography (pentane/EtOAc (100:1)) gave **7aEk** as an oil (42 mg, 55%); e. r. 86:14, 83% e.s. (over 2 steps from 93.5:6.5 e.r. batch of diboronate **5a**). The e.r. was determined by analysis by chiral SFC:

IB, 2mL/min, 100 bar, 50 °C, 1% → 40% MeCN over 10 min then 40% MeCN for 1 min; t_R 5.91 (major), 6.11 (minor).

^{1}H NMR (300 MHz, $CDCl_3$): 7.50 (d, *J* = 8.1 Hz, 2H, Ar**H**), 7.36-7.10 (m, 7H, Ar**H**), 7.08-6.90 (m, 4H, Ar**H**), 4.25 (t, *J* = 7.8, 1H, C**H**CHH’), 3.46-3.24 (m, 2H, CHC**HH**’); **^{13}C NMR (75 MHz, $CDCl_3$)**: 148.2 (**C**), 147.8 (**C**), 134.2 (**C**), 138.5 (**C**), 130.4 (**C**H), 128.9 (q, *J* = 32 Hz, **C**CF_3), 128.8 (**C**H), 128.5 (**C**H), 128.1 (**C**H), 126.9 (**C**H), 125.5 (q, *J* = 4 Hz, C**H**), 124.3 (q, *J* = 272 Hz, **C**F_3), 120.9 (**C**H), 120.6 (q, *J* = 257 Hz, **C**F_3), 53.0 (**C**H), 41.2 (**C**H_2); **^{19}F NMR (377 MHz, $CDCl_3$)**: –58.5, –62.9; **IR (film) cm^{-1}**: 3031w, 2938w, 1619w, 1509w; **HRMS (EI$^+$)**: $C_{22}H_{16}OF_6$ requires 410.1105; found 410.1101; $[\alpha]_D^{20}$ = +13.3 (*c* 0.6, $CHCl_3$).

(*R*)-1,3-Dimethyl-5-(1-phenyl-2-(*p*-tolyl)ethyl)benzene (compound **7aBd**, Figure 3)

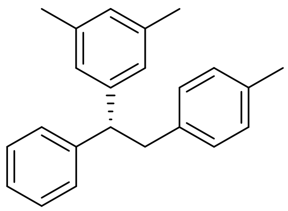

In a nitrogen filled glove box, boronic ester **6aB** (20 mg, 0.062 mmol, 1 eq, e.r. 92:8), 3,5-dimethyliodobenzene (21.6 mg, 0.093 mmol, 1.5 eq), $Pd(dba)_2$ (2.9 mg, 0.005 mmol, 0.08 eq), PPh_3 (10.4 mg, 0.039 mmol, 0.64 eq) and Ag_2O (24.5 mg, 0.093 mmol, 1.5 eq) were weighed into a 1 dram vial and DME (1.3 mL) was added. The reaction vessel was sealed, removed from the glove box and heated at 70 °C for 16 h. The reaction mixture was cooled and filtered through a plug of silica, eluted with EtOAc (30 mL) and concentrated *in vacuo*. Purification by column chromatography (pentane/CH_2Cl_2 (96.5:3.5)) gave **7aBd** (14 mg, 75%); e. r. 85:15, 83% e.s. (over 2 steps from 92:8 e.r. batch of diboronate **5a**). The e.r. was determined by analysis by chiral HPLC:

AD-H, hexane/2-propanol (95:5), 0.5 mL/min; t_R 10.9 (major), 12.6 (minor).

^{1}H NMR (400 MHz, $CDCl_3$): 7.34-7.12 (m, 6H, Ar**H**), 7.04-6.81 (m, 6H, Ar**H**), 4.16 (t, *J* = 7.6 Hz, 1H, C**H**CHH'), 3.41-3.27 (m, 2H, CHC**HH'**), 2.29 (s, 9H, C**H**$_3$); **^{13}C NMR (100 MHz, $CDCl_3$)**: 144.7 (Ar**C**), 137.7 (Ar**C**), 137.4 (Ar**C**), 135.1 (Ar**C**), 128.9 (Ar**C**), 128.7 (Ar**C**), 128.2 (Ar**C**), 128.1 (Ar**C**), 127.8 (Ar**C**), 126.0 (Ar**C**), 125.8 (Ar**C**), 53.1 (**C**H), 41.6 (**C**H_2), 21.4 (**C**H_3), 21.0 (**C**H_3); **HRMS (EI$^+$)**: $C_{23}H_{24}$ requires 300.1878; found 300.1892.

(*R*)-1-(4-(2-(6-Methoxynaphthalen-2-yl)-1-phenylethyl)phenyl)ethanone (compound **7aCj**, Figure 3)

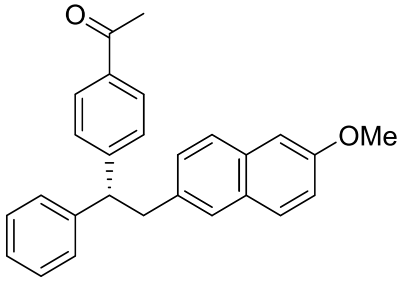

In a nitrogen filled glove box, boronic ester **6aC** (100 mg, 0.26 mmol, 1 eq, e.r. 97:3), 4-iodoacetophenone (95 mg, 0.39 mmol, 1.5 eq), $Pd(dba)_2$ (11.8 mg, 0.0206 mmol, 0.08 eq), PPh_3 (32.4 mg, 0.124 mmol, 0.48 eq) and Ag_2O (90 mg, 0.39 mmol, 1.5 eq) were weighed into a pressure tube and DME (5.1 mL) was added. The reaction vessel was sealed, removed from the glove box and heated at 72 °C for 16 h. The reaction mixture was cooled and filtered through a plug of silica, eluted with EtOAc (30 mL) and concentrated *in vacuo*. Purification by column chromatography (pentane/EtOAc (10:1)) gave **7aCj** as a pale yellow solid (60 mg, 61%); e. r. 92:8, 88% e.s. (over 2 steps from 98:2 e.r. batch of diboronate **5a**). The e.r. was determined by analysis by chiral SFC:

IA, 2mL/min, 100 bar, 50 °C, 1% → 40% MeCN over 10 min then 40% MeCN for 3 min; t_R 12.77 (minor), 13.01 (major).

^{1}H NMR (400 MHz, $CDCl_3$): 7.84 (d, *J* = 8.4 Hz, 2H, Ar**H**), 7.57 (d, *J* = 8.4 Hz, 2H, Ar**H**), 7.38 (s, 1H, Ar**H**), 7.34-7.17 (m, 7H, Ar**H**), 7.15-7.05 (m, 3H, Ar**H**), 4.40 (t, *J* = 7.8 Hz, 1H, C**H**CHH’), 3.89 (s, 3H, OC**H**3), 3.58-3.44 (m, 2H, CHC**HH**’), 2.54 (s, 3H, COC**H**3); **^{13}C NMR (100 MHz, $CDCl_3$)**: 197.9 (**C**=O), 157.4 (**C**), 150.1 (**C**), 143.8 (**C**), 135.4 (**C**), 135.0 (**C**), 133.2 (**C**), 129.1 (**C**H), 129.0 (**C**), 128.68 (**C**H), 128.65 (**C**H), 128.5 (**C**H), 128.1 (**C**H), 127.4 (**C**H), 126.71 (**C**H), 126.68 (**C**H), 118.8 (**C**H), 105.7 (**C**H), 55.4 (**C**H3), 53.2 (**C**H), 41.9 (**C**H2), 26.6 (**C**H3); **IR (film) cm^{-1}**: 2921w, 1673s, 1603; **HRMS (EI$^+$)**: $C_{27}H_{24}O_2$ requires 380.1776; found 380.1779; $[\alpha]_D^{20}$ = +57.5 (*c* 0.8, $CHCl_3$); mp 113-115 °C.

(*R*)-2-Methoxy-6-(2-(4-methoxyphenyl)-2-phenylethyl)naphthalene (compound **7aCf**, Figure 3)

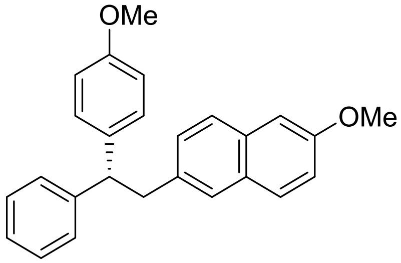

In a nitrogen filled glove box, boronic ester **6aC** (114 mg, 0.294 mmol, 1 eq, e.r. 97:3), 4-iodoanisole (103 mg, 0.44 mmol, 1.5 eq), $Pd(dba)_2$ (13.5 mg, 0.0235 mmol, 0.08 eq), PPh_3 (37.0 mg, 0.141 mmol, 0.48 eq) and Ag_2O (102 mg, 0.44 mmol, 1.5 eq) were weighed into a pressure tube and DME (5.9 mL) was added. The reaction vessel was sealed, removed from the glove box and heated at 72 °C for 16 h. The reaction mixture was cooled and filtered through a plug of silica, eluted with EtOAc (30 mL) and concentrated *in vacuo*. Purification by column chromatography (pentane/CH_2Cl_2 (2:1)) gave **7aCf** as a white solid (61 mg, 56%); e. r. 91:9, 85% e.s. (over 2 steps from 98:2 e.r. batch of diboronate **5a**). The e.r. was determined by analysis by chiral SFC:

IA, 1.5mL/min, 4% MeOH, 150 bar, T = 35 °C; t_R 43.8 (minor), 45.6 (major).

^{1}H NMR (500 MHz, $CDCl_3$): 7.60 (t, *J* = 8.0 Hz, 2H, Ar**H**), 7.40 (s, 1H, Ar**H**), 7.30-7.21 (m, 4H, Ar**H**), 7.20-7.05 (m, 6H, Ar**H**), 6.81 (d, *J* = 8.2 Hz, 2H, Ar**H**), 4.31 (t, *J* = 7.7 Hz, 1H, C**H**CHH’), 3.91 (s, 3H, OC**H**3), 3.77 (s, 3H, OC**H**3), 3.48 (d, *J* = 7.7 Hz, 2H, CHC**HH’**); **^{13}C NMR (125 MHz, $CDCl_3$)**: 158.0 (**C**), 157.3 (**C**), 145.1 (**C**), 136.8 (**C**), 135.7 (**C**), 133.1 (**C**), 129.1 (**C**H), 129.0 (**C**), 128.5 (**C**H), 128.4 (**C**H), 128.1 (**C**H), 127.4 (**C**H), 126.5 (**C**H), 126.2 (**C**), 118.6 (**C**H), 113.9 (**C**H), 105.7 (**C**H), 55.4 (O**C**H3), 55.3 (O**C**H3), 52.4 (**C**H), 42.4 (**C**H2); **IR (film) cm^{-1}**: 2954w, 2936w, 1632w, 1604w; **HRMS (ESI$^+$)**: $C_{25}H_{25}O_2$ requires 369.1849; found 136.1838; $[\alpha]_D^{20}$ = +23.3 (*c* 0.6, $CHCl_3$); mp 170-171 °C.

(*S*)-1-(4-(1-(Benzo[d][1,3]dioxol-5-yl)-2-(4-(trifluoromethoxy)phenyl)ethyl)phenyl)ethanone (Compound **7hEj**, Figure 3)

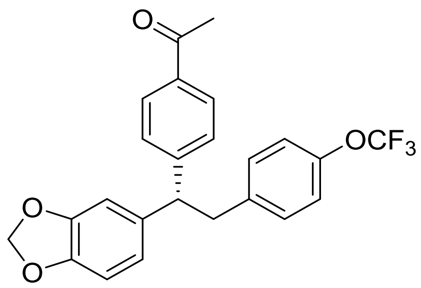

In a nitrogen filled glove box, boronic ester **6gE** (131 mg, 0.30 mmol, 1 eq, 93.5:6.5 e.r. (assumed based on e.r. of diboronate **5g**)), 4-iodoacetophenone (92 mg, 0.45 mmol, 1.5 eq), $Pd(dba)_2$ (13.8 mg, 0.024 mmol, 0.08 eq), PPh_3 (37.8 mg, 0.144 mmol, 0.48 eq) and Ag_2O (104 mg, 0.45 mmol, 1.5 eq) were weighed into a pressure tube and DME (6 mL) was added. The reaction vessel was sealed, removed from the glove box and heated at 72 °C for 16 h. The reaction mixture was cooled and filtered through a plug of silica, eluted with EtOAc (30 mL) and concentrated *in vacuo*. Purification by column chromatography (hexanes/EtOAc (96:4 → 90:10)) gave **7hEj** as a yellow oil (57 mg, 45%); e. r. 90:10, 92% e.s. (over 2 steps from 93.5:6.5 batch of diboronate **5g**). The e.r. was determined by analysis by chiral SFC:

IA, 2mL/min, 100 bar, 50 °C, 1% → 40% EtOH over 10 min then 40% EtOH for 3 min; t_R 10.09 (minor), 10.49 (major).

^{1}H NMR (300 MHz, $CDCl_3$): 7.86 (d, *J* = 8.2 Hz, 2H, Ar**H**), 7.27 (d, *J* = 8.2 Hz, 2H, Ar**H**), 7.08-6.96 (m, 4H, Ar**H**), 6.75-6.60 (m, 3H, Ar**H**), 5.91 (d, *J* = 1.7 Hz, 2H, OC**H**$_2$O), 4.18 (t, *J* = 7.8 Hz, 1H, C**H**CHH'), 3.32 (d, *J* = 7.8 Hz, 2H, CHC**HH**'), 2.56 (s, 3H, C**H**$_3$); **^{13}C NMR (75 MHz, $CDCl_3$)**: 197.8 (**C**=O), 149.8 (**C**), 148.0 (**C**), 147.7 (q, *J* = 1.4 Hz, **C**OCF_3), 146.4 (**C**), 138.5 (**C**), 137.1 (**C**), 135.6 (**C**), 130.3 (**C**H), 128.8 (**C**H), 128.2 (**C**H), 121.1 (**C**H), 120.8 (**C**H), 120.6 (q, *J* = 257 Hz, **C**F_3), 108.4 (**C**H), 101.1 (**C**H_2), 52.8 (**C**H), 41.2 (**C**H_2), 26.7 (**C**H_3); **^{19}F NMR (377 MHz, $CDCl_3$)**: –58.4; **IR (film) cm^{-1}**: 2894w, 1680s, 1605w; **HRMS (EI$^+$)**: $C_{24}H_{19}O_4F_3$ requires 428.1235; found 428.1241; $[\alpha]_D^{20}$ = +13.3 (*c* 0.6, $CHCl_3$).

Synthesis of Diboronate Used in Figure 4a

4,4,5,5-tetramethyl-2-(4-vinylphenyl)-1,3,2-dioxaborolane

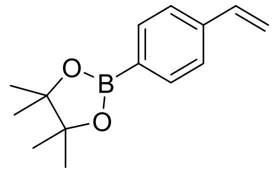

The title compound was prepared according to a modified literature procedure.[10] In a round bottom flask with a stir bar, 4-vinylphenylboronic acid (396.9 mg, 2.50 mmol, 1 equiv.), $MgSO_4$ (60.2 mg, 0.50 mmol, 0.2 equiv.), and pinacol (301.4 mg, 2.55, 1.02 equiv.) were taken up in tetrahydrofuran (12.5 mL). The heterogeneous mixture was stirred at room temperature for 2 hours. After concentration under reduced pressure, the remaining solids were washed repeatedly with 3 x ~15 mL diethyl ether, which was filtered through a plug of silica gel and a PROMAX™ 0.22 µm PTFE syringe filter. The combined washes were concentrated under reduced pressure to afford the product (575.3 mg, 2.5 mmol, quant.) as a clear colourless oil. Spectroscopic data were in agreement with the literature.[10]

4,4,5,5-tetramethyl-2-(4-(1-(4,4,5,5-tetramethyl-1,3,2-dioxaborolan-2-yl)ethyl)phenyl)-1,3,2-dioxaborolane (compound **8**, Figure 4a)

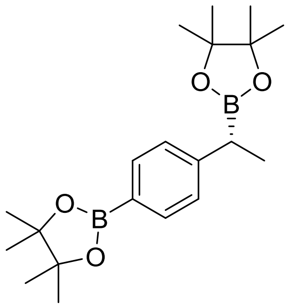

The title compound was prepared according to a modified literature procedure.[11] In a nitrogen filled glove box, to an oven dried schlenk tube was added $[Rh(COD)_2]BF_4$ (16.3mg, 0.04 mmol, 0.02 equiv.), (*R*)-BINAP (28.1 mg, 0.045 mmol, 0.22 equiv.), and 1,2-dimethoxyethane (1.0 mL). The resultant solution was stirred at glovebox temperature for 15 minutes after which time 4,4,5,5-tetramethyl-2-(4-vinylphenyl)-1,3,2-dioxaborolane (471.6 mg, 2.05 mmol, 1 equiv.) was added using an additional 1.5 mL of 1,2-dimethoxyethane. The resultant solution was removed from the glovebox, placed under argon, and cooled to −68 °C. A solution of catecholborane (293.3 mg, 2.45 mmol, 1.19 equiv.) in 1,2-dimethoxyethane (1 mL) was then added dropwise down the wall of the schlenk tube over 40 minutes. After an additional 4 hours at −68 °C, pinacol (613.0 mg, 5.19 mmol, 2.53 equiv.) was added in one portion and the reaction mixture was warmed to room temperature overnight. After concentration under reduced pressure, the crude material was purified by column chromatography (4% ethyl acetate/hexanes) to afford **8** (546 mg, 1.52 mmol, 74%) as a white solid. The e.r. was determined by oxidizing a portion of the product to the diol (by **GP1**) followed by analysis by chiral SFC:
IF, 2mL/min, 100 bar, 50 °C, 1% → 40% MeOH over 10 min then 40% MeOH for 1 min; t_R 8.11 (major), 8.23 (minor).

^{1}H NMR (400 MHz; CDCl$_3$): 7.72 (d, *J* = 7.9 Hz, 2H, Ar**H**), 7.23 (d, *J* = 8.0 Hz, 2H, Ar**H**), 2.45 (q, *J* = 7.4 Hz, 1H, C**H**-CH$_3$), 1.33 (m, 15H, CHC**H**$_3$ & C(C**H**$_3$)$_2$C(C**H**$_3$)$_2$), 1.20 (s, 6H, C(C**H**$_3$)(CH$_3$)'C(C**H**$_3$)(CH$_3$)')), 1.19 (s, 6H, C(CH$_3$)(C**H**$_3$)'C(CH$_3$)(C**H**$_3$)')); **^{13}C NMR (100 MHz; CDCl$_3$)**: 149.7 (**C**), 135.0 (**C**H), 127.4 (**C**H), 83.7 (**C**-OB), 83.4 (**C**-OB), 25.0 (**C**H), 24.73 (**C**H$_3$), 24.70 (**C**H$_3$), 16.9 (**C**H$_3$), peaks not observed for **C**'s bound to B's; **^{11}B NMR (128

MHz; $CDCl_3$): 32.7; **IR (film) cm^{-1}:** 2978w, 1606w, 1321, 1138w, 845w; **HRMS (EI^+)**: $C_{20}H_{32}B_2O_4$ requires 358.2487; found 358.2491; $[\alpha]_D^{20} = -5.7$ (*c* 1.4, CH_2Cl_2); mp 128.6-130.0 °C.

Orthogonal Coupling of Primary B–C$^{(sp2)}$ Bond in Presence of Secondary Shown in Figure 4a

General Procedure 2: An oven dried pressure tube with a magnetic stir bar in a glovebox was charged with diboronate **8** (615.5 mg, 1.72 mmol, 1 equiv.), $Pd_2(dba)_3$ (77.1 mg, 0.084 mmol, 0.05 equiv.), $[(t\text{Bu})_3PH]BF_4$ (98.2 mg, 0.34 mmol, 0.2 equiv.), K_2CO_3 (703.9 mg, 5.09 mmol, 2.96 equiv.), 4-bromoanisole (380.8 mg, 2.04 mmol, 1.2 equiv.), and toluene (3.4 mL). The pressure tube was sealed with a rubber septum, removed from the glovebox, and placed under argon. Water sparged with argon (95 µL) was added and the rubber septum was replaced with a lid. The reaction was heated to 60 °C for 24 hours. After cooling to room temperature, the reaction mixture was filtered through a plug of silica gel (ca. 2 mL) and a PROMAX™ 0.22 µm PTFE syringe filter using copious ethyl acetate (~30 mL).

2-(1-(4'-methoxy-[1,1'-biphenyl]-4-yl)ethyl)-4,4,5,5-tetramethyl-1,3,2-dioxaborolane (compound **9aF**, Figure 4a)

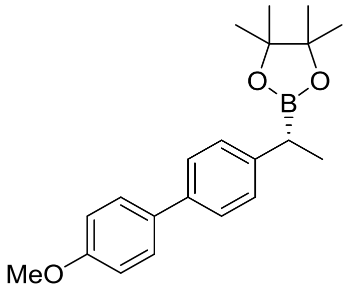

The title compound was prepared according to **general procedure 2**. After filtration, the crude material was purified by column chromatography (2% to 3% ethyl acetate/hexanes) to afford **9aF** (451 mg, 1.33 mmol, 78%) as an ocher solid; e.r. 96:4. The e.r. was determined by oxidizing a portion of the product to the alcohol (by **GP1**) followed by analysis by chiral SFC:

IB, 2mL/min, 100 bar, 50 °C, 1% → 40% MeOH over 10 min then 40% MeOH for 1 min; t_R 9.30 (minor), 9.57 (major).

^{1}H NMR (400 MHz; $CDCl_3$): 7.53 (d, J = 8.7 Hz, 2H, Ar**H**), 7.47 (d, J = 8.1 Hz, 2H, Ar**H**), 7.28 (d, J = 8.1 Hz, 2H, Ar**H**), 6.96 (d, J = 8.7 Hz, 2H, Ar**H**), 3.85 (s, 3H, OC**H**$_3$), 2.48 (q, J = 7.4 Hz, 1H, C**H**-CH_3), 1.37 (d, J = 7.5 Hz, 3H, CH-C**H**$_3$), 1.24 (s, 6H, C(C**H**$_3$)(CH_3)'C(C**H**$_3$)(CH_3)')), 1.23 (s, 6H, C(CH_3)(C**H**$_3$)'C(CH_3)(C**H**$_3$)')); **^{13}C NMR (100 MHz; $CDCl_3$)**: 159.0 (**C**), 143.7 (**C**), 137.7 (**C**), 134.0 (**C**), 128.3 (**C**H), 128.1 (**C**H), 126.8 (**C**H), 114.2 (**C**H), 83.5 (**C**-OB), 55.5 (**C**H$_3$), 24.8 (**C**H$_3$), 24.75 (**C**H$_3$), 17.26 (**C**H$_3$), peak not observed for **C** bound to B; **^{11}B NMR (128 MHz; $CDCl_3$)**: 33.3; **IR (film) cm^{-1}:** 2970w, 1607w, 1494w, 1322w, 818w; **HRMS (EI)**: $C_{21}H_{27}BO_3$ requires 338.2053; found 338.2046; $[\alpha]_D^{20}$ = −5.0 (c 2.0, CH_2Cl_2); mp 64.5-66.3 °C.

2-(1-(3',5'-dimethyl-[1,1'-biphenyl]-4-yl)ethyl)-4,4,5,5-tetramethyl-1,3,2-dioxaborolane (compound **9aD**, Figure 4a)

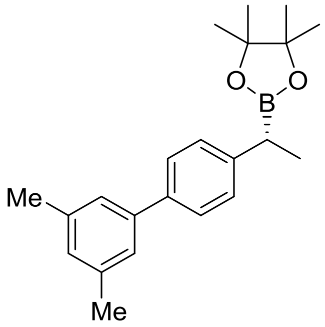

The title compound was prepared according to **general procedure 2** using diboronate **8** (609.2 mg, 1.70 mmol, 1 equiv.), $Pd_2(dba)_3$ (78.2 mg, 0.085 mmol, 0.05 equiv.), $[(t\text{Bu})_3PH]BF_4$ (100.2 mg, 0.35 mmol, 0.2 equiv.), K_2CO_3 (711.6 mg, 5.15 mmol, 3.0 equiv.), and 1-bromo-3,5-dimethylbenzene (385.0 mg, 2.1 mmol, 1.2 equiv.). After filtration, the crude material was purified by column chromatography (2% to 2.5% ethyl acetate/hexanes) to afford **9aD** (523 mg, 1.56 mmol, 91%) as an ocher solid; e.r. 96:4. The e.r. was determined by oxidizing a portion of the product to the alcohol (by **GP1**) followed by analysis by chiral SFC:

IE, 3.9mL/min, 100 bar, 50 °C, 1% → 59.5% MeOH over 15 min; t_R 10.72 (major), 11.22 (minor).

^{1}H NMR (400 MHz; CDCl$_3$): 7.46 (d, *J* = 8.2 Hz, 2H, Ar**H**), 7.25 (d, *J* = 8.2 Hz, 2H, Ar**H**), 7.18 (s, 2H, Ar**H**), 6.93 (s, 1H, Ar**H**), 2.45 (q, *J* = 7.4 Hz, 1H, C**H**-CH$_3$), 2.34 (s, 6H, C**H**$_3$), 1.35 (d, *J* = 7.5 Hz, 3H, CH-C**H**$_3$), 1.20 (s, 6H, C(C**H**$_3$)(CH$_3$)'C(C**H**$_3$)(CH$_3$)')), 1.19 (s, 6H, C(CH$_3$)(C**H**$_3$)'C(CH$_3$)(C**H**$_3$)')); **^{13}C NMR (100 MHz; CDCl$_3$)**: 144.1 (**C**), 141.4 (**C**), 138.3 (**C**), 138.2 (**C**H), 128.6 (**C**H), 128.2 (**C**H), 127.2 (**C**H), 125.0 (**C**H), 83.5 (**C**-OB), 24.8 (**C**H$_3$), 24.75 (**C**H$_3$), 21.6 (**C**H$_3$), 17.2 (**C**H$_3$), peak not observed for **C** bound to B; **^{11}B NMR (128 MHz; CDCl$_3$)**: 33.3; **IR (film) cm^{-1}:** 2980w, 2871w, 1601w, 1317w, 1140w, 829w; **HRMS (EI)**: $C_{22}H_{29}BO_2$ requires 336.2261; found 336.2253; $[\alpha]_D^{20} = -4.1$ (*c* 1.5, CH_2Cl_2); mp 65.8-67.7 °C.

1-(4'-(1-(4,4,5,5-tetramethyl-1,3,2-dioxaborolan-2-yl)ethyl)-[1,1'-biphenyl]-4-yl)ethan-1-one (compound **9aJ**, Figure 4a)

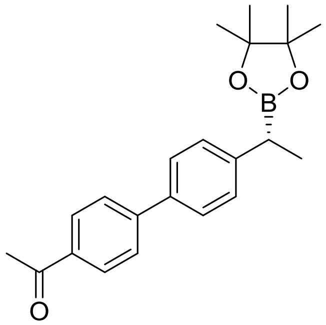

The title compound was prepared according to **general procedure 2** using diboronate **8** (616.6 mg, 1.72 mmol, 1 equiv.), $Pd_2(dba)_3$ (77.3 mg, 0.084 mmol, 0.05 equiv.), $[(t\text{Bu})_3PH]BF_4$ (105.6 mg, 0.36 mmol, 0.2 equiv.), K_2CO_3 (717.1 mg, 5.19 mmol, 3.0 equiv.), and 4-bromo-acetophenone (411.0 mg, 2.1 mmol, 1.2 equiv.). After filtration, the crude material was purified by column chromatography (4% ethyl acetate/hexanes) to afford **9aJ** (497 mg, 1.42 mmol, 82%) as an ocher solid; e.r. 96:4. The e.r. was determined by oxidizing a portion of the product to the alcohol (by **GP1**) followed by analysis by chiral SFC:

IA, 2mL/min, 100 bar, 50 °C, 10% MeOH; t_R 19.28 (minor), 20.68 (major).

^{1}H NMR (400 MHz; CDCl$_3$): 8.01 (d, *J* = 8.3 Hz, 2H, Ar**H**), 7.68 (d, *J* = 8.3 Hz, 2H, Ar**H**), 7.55 (d, *J* = 8.2 Hz, 2H, Ar**H**), 7.33 (d, *J* = 8.2 Hz, 2H, Ar**H**), 2.62 (s, 3H, C**H**$_3$), 2.50 (q, *J* = 7.4 Hz, 1H, C**H**-CH$_3$), 1.38 (d, *J* = 7.5 Hz, 3H, CH-C**H**$_3$), 1.23 (s, 6H, C(C**H**$_3$)(CH$_3$)'C(C**H**$_3$)(CH$_3$)')) 1.22 (s, 6H, C(CH$_3$)(C**H**$_3$)'C(CH$_3$)(C**H**$_3$)')); **^{13}C NMR (100 MHz; CDCl$_3$)**: 197.9 (**C**=O), 146.0 (**C**), 145.6 (**C**), 136.7(**C**), 135.6 (**C**), 129.0 (**C**H), 128.5 (**C**H), 127.3 (**C**H), 127.0 (**C**H), 83.6 (**C**-OB), 26.8 (**C**H$_3$), 24.8 (**C**H$_3$), 24.75 (**C**H$_3$), 17.2 (**C**H$_3$), peak not observed for **C** bound to B; **^{11}B NMR (128 MHz; CDCl$_3$)**: 33.1; **IR (film) cm^{-1}:** 2975w, 1678w, 1600w, 1322w, 818w; **HRMS (EI)**: $C_{22}H_{27}BO_3$ requires 350.2053; found 350.2066; $[\alpha]_D^{20}$ = −3.7 (*c* 1.1, CH_2Cl_2); mp 100-101.6 °C.

Stereoretentive Coupling of Remaining Secondary Benzylic B–C Bond in Enantioenriched Boronic Esters Shown in Figure 4a

General Procedure 3: An oven dried pressure tube with a stir bar in a glovebox was charged with benzylic boronic ester **9aF** (174.7 mg, 0.52 mmol, 1 equiv.), $Pd(dba)_2$ (23.5 mg, 0.041 mmol, 0.08 equiv.), PPh_3 (66.2 mg, 0.25 mmol, 0.49 equiv.), Ag_2O (178.0 mg, 0.77 mmol, 1.48 equiv.), 4-iodotoluene (105.3 mg, 0.81 mmol, 1.5 equiv.), and 1,2-dimethoxyethane (6.0 mL). The pressure tube was sealed, removed from the glovebox, and heated to 70 °C for 16 hours. After cooling to room temperature the reaction mixture was filtered through a plug of silica gel and a PROMAX™ 0.22 µm PTFE syringe filter using ethyl acetate (~30 mL).

4-methoxy-4'-(1-(p-tolyl)ethyl)-1,1'-biphenyl (compound **10aFb**, Figure 4a)

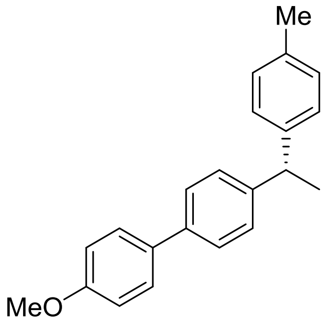

The title compound was prepared according to **general procedure 3**. After filtration, the crude material was purified by column chromatography (2% ethyl acetate/hexanes) to afford **10aFb** (101.0 mg, 0.33 mmol, 65%) as an ocher solid; e.r. 90.5:9.5, 88% e.s. (over 2 steps from 96:4 e.r. diboronate **8**). The e.r. was determined by analysis by chiral SFC:

IE, 2mL/min, 100 bar, 50 °C, 1% → 40% MeOH over 10 min then 40% MeOH for 3 min; t_R 9.25 (major), 9.68 (minor).

^{1}H NMR (400 MHz; $CDCl_3$): 7.54 (d, *J* = 8.7 Hz, 2H, Ar**H**), 7.50 (d, *J* = 8.2 Hz, 2H, Ar**H**), 7.30 (d, *J* = 8.1 Hz, 2H, Ar**H**), 7.19 (d, *J* = 8.1 Hz, 2H, Ar**H**), 7.15 (d, *J* = 8.0 Hz, 2H, Ar**H**), 6.99 (d, *J* = 8.7 Hz, 2H, Ar**H**), 4.19 (q, *J* = 7.2 Hz, 1H, C**H**-CH_3), 3.87 (s, 3H, OC**H**$_3$), 2.36 (s, 3H, C**H**$_3$), 1.69 (d, *J* = 7.2 Hz, 3H, CH-C**H**$_3$); **^{13}C NMR (100 MHz; $CDCl_3$)**: 159.1 (**C**), 145.2 (**C**), 143.5 (**C**), 138.6 (**C**), 135.6 (**C**), 133.7 (**C**), 129.2 (**C**H), 128.1 (**C**H), 128.0 (**C**H), 127.6 (**C**H), 126.8 (**C**H), 114.3 (**C**H), 55.4 (**C**H$_3$), 44.2 (**C**H), 22.1 (**C**H$_3$), 21.1 (**C**H$_3$); **IR (film) cm^{-1}:** 2960w, 1603w, 1493w, 1034w, 826w; **HRMS (EI)**: $C_{22}H_{22}O$ requires 302.1671; found 302.1659; $[\alpha]_D^{20}$ = +8.7 (*c* 1.4, CH_2Cl_2); mp 131-133 °C.

4-(1-(4-chlorophenyl)ethyl)-4'-methoxy-1,1'-biphenyl (compound **10aFl**, Figure 4a)

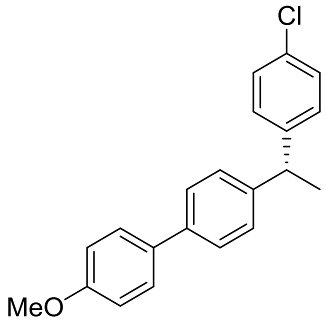

The title compound was prepared according to **general procedure 3** using benzylic boronic ester **9aF** (174.0 mg, 0.51 mmol, 1 equiv.), $Pd(dba)_2$ (23.4 mg, 0.041 mmol, 0.08 equiv.), PPh_3 (66.2 mg, 0.25 mmol, 0.49 equiv.), Ag_2O (174.2 mg, 0.75 mmol, 1.44 equiv.), 1-chloro-4-iodobenzene (179.8 mg, 0.75 mmol, 1.5 equiv.), and 1,2-dimethoxyethane (6.0 mL). After filtration, the crude material was purified by column chromatography (1.5% ethyl acetate/hexanes) to afford **10aFl** (97.0 mg, 0.30 mmol, 60%) as an ocher solid; e.r. 90:10, 87% e.s. (over 2 steps from 96:4 e.r. diboronate **8**). The e.r. was determined by analysis by chiral SFC:

IE, 2mL/min, 100 bar, 50 °C, 1% → 40% MeOH over 10 min then 40% MeOH for 3 min; t_R 11.24 (minor), 11.71 (major).

^{1}H NMR (400 MHz; $CDCl_3$): 7.51 (m, 4H, Ar**H**), 7.26 (m, 4H, Ar**H**), 7.19 (d, J = 8.5 Hz, 2H, Ar**H**), 6.97 (m, 2H, Ar**H**), 4.16 (q, J = 7.2 Hz, 1H, C**H**-CH_3), 3.85 (s, 3H. OC**H**$_3$), 1.65 (d, J = 7.2 Hz, 3H, CH-C**H**$_3$); **^{13}C NMR (100 MHz; $CDCl_3$)**: 159.2 (**C**), 145.0 (**C**), 144.4 (**C**), 139.0 (**C**), 133.6 (**C**), 131.9 (**C**), 129.1 (**C**H), 128.6 (**C**H), 128.2 (**C**H), 128.0 (**C**H), 126.9 (**C**H), 114.33 (**C**H), 55.47 (**C**H_3), 44.0 (**C**H), 22.0 (**C**H_3); **IR (film) cm^{-1}:** 2965w, 1603w, 1486w, 1206w, 823w; **HRMS (EI)**: $C_{22}H_{19}ClO$ requires 322.1124; found 322.1137; $[\alpha]_D^{20}$ = +6.0 (c 1.0, CH_2Cl_2); mp 156.2-158.2 °C.

3-(1-(4'-methoxy-[1,1'-biphenyl]-4-yl)ethyl)pyridine (compound **10aFi**, Figure 4a)

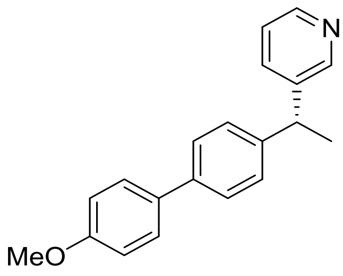

The title compound was prepared according to **general procedure 3** using benzylic boronic ester **9aF** (102.4 mg, 0.30 mmol, 1 equiv.), $Pd(dba)_2$ (13.6 mg, 0.024 mmol, 0.08 equiv.), PPh_3 (37.5 mg, 0.14 mmol, 0.48 equiv.), Ag_2O (108.2 mg, 0.47 mmol, 1.56 equiv.), 3-iodopyridine (93.3 mg, 0.46 mmol, 1.5 equiv.), and 1,2-dimethoxyethane (6.0 mL). After filtration, the crude material was purified by column chromatography (10% ethyl acetate/hexanes + 2 v/v% NEt_3) to afford **10aFi** (46.0 mg, 0.16 mmol, 53%) as an ocher solid; e.r. 93:7, 96% e.s. (over 2 steps from 95:5 e.r. diboronate **8**). The e.r. was determined by analysis by chiral SFC:

IE, 3.9mL/min, 100 bar, 50 °C, 1% → 59.5% MeOH over 15 min; t_R 13.87 (major), 14.85 (minor).

^{1}H NMR (400 MHz; $CDCl_3$): 8.56 (brs, 1H, Ar**H**), 8.45 (brs, 1H, Ar**H**), 7.49 (m, 5H, Ar**H**), 7.22 (m, 3H, Ar**H**), 6.95 (d, *J* = 8.6 Hz, 2H, Ar**H**), 4.20 (q, *J* = 7.1 Hz, 1H, C**H**-CH_3), 3.83 (s, 3H, OC**H**$_3$), 1.68 (d, *J* = 7.2 Hz, 3H, CH-C**H**$_3$); **^{13}C NMR (100 MHz; $CDCl_3$)**: 159.2 (**C**), 149.5 (**C**H), 147.7 (**C**H), 143.6 (**C**), 141.7 (**C**), 139.2 (**C**), 135.1 (**C**H), 133.4 (**C**), 128.1 (**C**H), 128.0 (**C**H), 127.0 (**C**H), 123.5 (**C**H), 114.3 (**C**H), 55.5 (**C**H$_3$), 42.2 (**C**H), 21.7 (**C**H$_3$); **IR (film) cm^{-1}:** 2966w, 1604w, 1494w, 1250w, 825w; **HRMS (EI)**: $C_{20}H_{19}NO$ requires 289.1467; found 289.1461; $[\alpha]_D^{20}$ = +8.7 (*c* 0.9, CH_2Cl_2); mp 105-107 °C.

4-methoxy-4'-(1-(4-(trifluoromethyl)phenyl)ethyl)-1,1'-biphenyl (compound **10aFk**, Figure 4a)

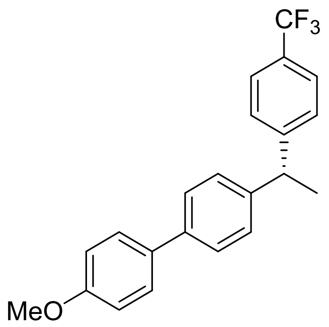

The title compound was prepared according to **general procedure 3** using benzylic boronic ester **9aF** (1.00 g, 2.96 mmol, 1 equiv.), Pd(dba)$_2$ (136.9 mg, 0.238 mmol, 0.08 equiv.), PPh_3 (377.7 mg, 1.44 mmol, 0.49 equiv.), Ag_2O (1.03 g, 4.45 mmol, 1.5 equiv.), 4-iodobenzotrifluoride (1.22 g, 4.48 mmol, 1.5 equiv.), and 1,2-dimethoxyethane (60.0 mL). After filtration, the crude material was purified by column chromatography (2.0% ethyl acetate/hexanes) to afford **10aFk** (695 mg, 1.95 mmol, 66%) as an ocher solid; e.r. 90:10, 89% e.s. (over 2 steps from 90:5 e.r. diboronate **8**). The e.r. was determined by analysis by chiral SFC:

IE, 2mL/min, 100 bar, 50 °C, 10% → 20% MeOH over 10 min; t_R 8.44 (major), 8.76 (minor).

^{1}H NMR (400 MHz; $CDCl_3$): 7.55 (d, *J* = 8.1 Hz, 2H, Ar**H**), 7.50 (m, 4H, Ar**H**), 7.37 (d, *J* = 8.0 Hz, 2H, Ar**H**), 7.25 (d, *J* = 7.8 Hz, 2H, Ar**H**), 6.97 (d, *J* = 8.8 Hz, 2H, Ar**H**), 4.24 (q, *J* = 7.1 Hz, 1H, C**H**-CH_3), 3.85 (s, 3H. OC**H**$_3$), 1.69 (d, *J* = 7.2 Hz, 3H, CH-C**H**$_3$); **^{13}C NMR (100 MHz; $CDCl_3$)**: 159.3 (**C**), 150.6 (**C**), 143.8 (**C**), 139.8 (**C**), 133.5 (**C**), 128.5 (q, *J* = 32.4 Hz, (**C**) 128.14 (**C**H), 128.09 (**C**H), 128.08 (**C**H), 127.0 (**C**H), 125.5 (q, *J* = 3.8 Hz, (**C**H), 124.5 (q, *J* = 271.9 Hz, **C**F$_3$), 114.4 (**C**H), 55.4 (**C**H$_3$), 44.5 (**C**H), 21.8 (**C**H$_3$); **^{19}F NMR (377 MHz; $CDCl_3$)**: 60.51; **IR (film) cm^{-1}:** 2973w, 1604w, 1419w, 1107w, 825w; **HRMS (EI)**: $C_{22}H_{19}F_3O$ requires 356.1388; found 356.1392; $[\alpha]_D^{20}$ = +10.0 (*c* 1.0, CH_2Cl_2); mp 155.8-157 °C.

4'-(1-(4-methoxyphenyl)ethyl)-3,5-dimethyl-1,1'-biphenyl (compound **10aDf**, Figure 4a)

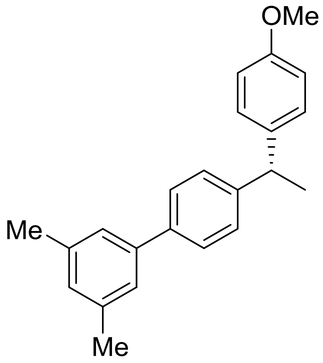

The title compound was prepared according to **general procedure 3** using benzylic boronic ester **9aD** (100.8 mg, 0.30 mmol, 1 equiv.), Pd(dba)$_2$ (13.2 mg, 0.023 mmol, 0.08 equiv.), PPh_3 (37.8 mg, 0.14 mmol, 0.48 equiv.), Ag_2O (103.6 mg, 0.45 mmol, 1.5 equiv.), 4-iodoanisole (105.0 mg, 0.45 mmol, 1.5 equiv.), and 1,2-dimethoxyethane (6.0 mL). After filtration, the crude material was purified by column chromatography (0.5% ethyl acetate/hexanes) to afford **10Df** (57.0 mg, 0.18 mmol, 60%) as a pale oil; e.r. 91:9, 90% e.s. (over 2 steps from 96:4 e.r. diboronate **8**). The e.r. was determined by analysis by chiral SFC:

IA, 2mL/min, 100 bar, 50 °C, 1% → 40% MeCN over 10 min then 40% MeCN for 3 min; t_R 10.33 (minor), 10.75 (major).

^{1}H NMR (400 MHz; CDCl$_3$): 7.45 (d, *J* = 8.2 Hz, 2H, Ar**H**), 7.22 (d, *J* = 8.2 Hz, 2H, Ar**H**), 7.14 (s, 4H, Ar**H**), 6.93 (s, 1H, Ar**H**), 6.81 (d, *J* = 8.7 Hz, 2H, Ar**H**), 4.10 (q, *J* = 7.2 Hz, 1H, C**H**-CH$_3$), 3.73 (s, 3H, OC**H**$_3$), 2.33 (s, 6H, C**H**$_3$), 1.61 (d, *J* = 7.2 Hz, 3H, CH-C**H**$_3$); **^{13}C NMR (100 MHz; CDCl$_3$)**: 158.0 (**C**), 145.8 (**C**), 141.2 (**C**), 139.2 (**C**), 138.6 (**C**), 138.3 (**C**), 128.8 (**C**H), 128.6 (**C**H), 128.0 (**C**H), 127.2 (**C**H), 125.1 (**C**H), 113.9 (**C**H), 55.3 (**C**H$_3$), 43.8 (**C**H), 22.2 (**C**H$_3$), 21.5 (**C**H$_3$); **IR (film) cm^{-1}:** 2961w, 1603w, 1508w, 1243w, 1033w, 826w; **HRMS (EI)**: $C_{23}H_{24}O$ requires 316.1827; found 316.13837; $[\alpha]_D^{20}$ = +3.6 (*c* 1.0, CH_2Cl_2).

1-(1-(3',5'-dimethyl-[1,1'-biphenyl]-4-yl)ethyl)naphthalene (compound **10aDm**, figure 4a)

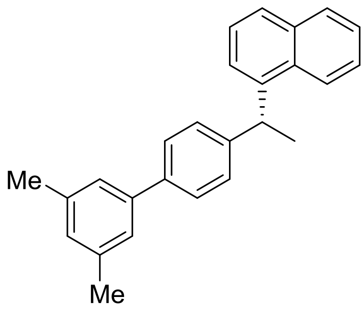

The title compound was prepared according to **general procedure 3** using benzylic boronic ester **9aD** (100.2 mg, 0.30 mmol, 1 equiv.), $Pd(dba)_2$ (13.3 mg, 0.023 mmol, 0.08 equiv.), PPh_3 (37.8 mg, 0.14 mmol, 0.48 equiv.), Ag_2O (107.4 mg, 0.46 mmol, 1.5 equiv.), 1-iodonaphthalene (111.5 mg, 0.43 mmol, 1.5 equiv.), and 1,2-dimethoxyethane (6.0 mL). After filtration, the crude material was purified by column chromatography (0.5% ethyl acetate/hexanes) to afford **10aDm** (58.0 mg, 0.17 mmol, 57%) as an ocher solid; e.r. 85:15, 76% e.s. (over 2 steps from 96:4 e.r. diboronate **8**). The e.r. was determined by analysis by chiral SFC:

IB, 2mL/min, 100 bar, 45 °C, 1% → 30% MeCN over 13 min then 30% MeCN for 1 min; t_R 13.12 (minor), 13.29 (major).

^{1}H NMR (400 MHz; CDCl$_3$): 8.06 (dd, *J* = 6.2 Hz, 3.5 Hz, 1H, Ar**H**), 7.83 (dd, *J* = 6.1 Hz, 3.4 Hz, 1H, Ar**H**), 7.73 (dd, *J* = 6.8 Hz, 2.2 Hz, 1H, Ar**H**), 7.43 (m, 6H, Ar**H**), 7.25 (d, *J* = 8.2 Hz, 2H, Ar**H**), 7.14 (s, 2H, Ar**H**), 6.93 (s, 1H, Ar**H**), 4.93 (q, *J* = 7.1 Hz, 1H, C**H**-CH_3), 2.32 (s, 6H, C**H**$_3$), 1.77 (d, *J* = 7.1 Hz, 3H, CH-C**H**$_3$); **^{13}C NMR (100 MHz; CDCl$_3$)**: 145.7 (**C**), 141.7 (**C**), 141.1 (**C**), 139.3 (**C**), 138.3 (**C**), 134.2 (**C**), 131.9 (**C**), 128.9 (**C**H), 128.8 (**C**H), 128.0 (**C**H), 127.3 (**C**H), 127.2 (**C**H), 126.0 (**C**H), 125.6 (**C**H), 125.5 (**C**H), 125.1 (**C**H), 124.5 (**C**H), 124.1 (**C**H), 40.4 (**C**H), 22.7 (**C**H$_3$), 21.5 (**C**H$_3$); **IR (film) cm^{-1}:** 2916w, 1596w, 1454w, 828w, 780w; **HRMS (EI)**: $C_{26}H_{24}$ requires 336.1878; found 336.1872; $[\alpha]_D^{20}$ = +47.3 (*c* 1.1, CH_2Cl_2); mp 123-125 °C.

1-(4'-(1-(pyridin-3-yl)ethyl)-[1,1'-biphenyl]-4-yl)ethan-1-one (compound **10aJi**, Figure 4a)

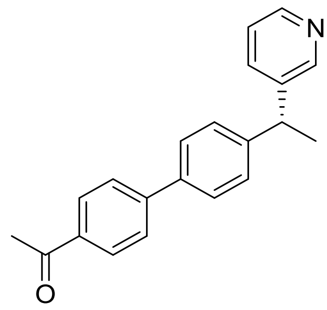

The title compound was prepared according to **general procedure 3** using benzylic boronic ester **9aJ** (104.9 mg, 0.30 mmol, 1 equiv.), $Pd(dba)_2$ (13.6 mg, 0.024 mmol, 0.08 equiv.), PPh_3 (39.6 mg, 0.15 mmol, 0.50 equiv.), Ag_2O (104.5 mg, 0.45 mmol, 1.5 equiv.), 3-iodopyridine (91.2 mg, 0.44 mmol, 1.5 equiv.), and 1,2-dimethoxyethane (6.0 mL). After filtration, the crude material was purified by column chromatography (30% ethyl acetate/hexanes + 2 v/v% NEt_3) to afford **10aJi** (57.0 mg, 0.19 mmol, 63%) as an ocher solid; e.r. 75:25, 54% e.s. (over 2 steps from 96:4 e.r. diboronate **8**). The e.r. was determined by analysis by chiral SFC:

IA, 2mL/min, 100 bar, 50 °C, 1% → 73% EtOH over 15 min; t_R 13.34 (major), 13.98 (minor).

^{1}H NMR (400 MHz; $CDCl_3$): 8.55 (brs, 1H, Ar**H**), 8.45 (brs, 1H, Ar**H**), 8.00 (d, *J* = 8.3 Hz, 2H, Ar**H**), 7.64 (d, *J* = 8.2 Hz, 2H, Ar**H**), 7.55 (d, *J* = 8.1 Hz, 2H, Ar**H**), 7.52 (d, *J* = 7.9 Hz, 1H, Ar**H**), 7.30 (d, *J* = 8.1 Hz, 2H, Ar**H**), 7.21 (dd, *J* = 7.5 Hz, 4.8 Hz, 1H, Ar**H**), 4.21 (q, *J* = 7.0 Hz, 1H, C**H**-CH_3), 2.60 (s, 3H, C**H**$_3$), 1.69 (d, *J* = 7.2 Hz, 3H, CH-C**H**$_3$); **^{13}C NMR (100 MHz; $CDCl_3$)**: 197.7 (**C**=O), 149.4 (**C**H), 147.7 (**C**H), 145.32 (**C**), 145.30 (**C**), 141.3 (**C**), 138.1 (**C**), 135.8 (**C**), 135.0 (**C**H), 129.0 (**C**H), 128.2 (**C**H), 127.5 (**C**H), 127.1 (**C**H), 123.5 (**C**H), 42.2 (**C**H), 26.7 (**C**H$_3$), 21.5 (**C**H$_3$); **IR (film) cm^{-1}:** 2976w, 1677s, 1599w, 1265w, 813w; **HRMS (EI)**: $C_{21}H_{19}NO$ requires 301.1467; found 301.1461; $[\alpha]_D^{20}$ = +6.0 (*c* 1.0, CH_2Cl_2); mp 78.2-80 °C.

1-(4'-(1-(4-(trifluoromethyl)phenyl)ethyl)-[1,1'-biphenyl]-4-yl)ethan-1-one (compound **10aJk**, Figure 4)

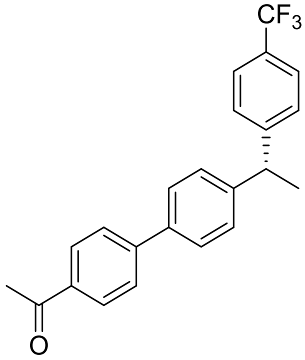

The title compound was prepared according to **general procedure 3** using benzylic boronic ester **9aJ** (105.8 mg, 0.30 mmol, 1 equiv.), Pd(dba)$_2$ (13.2 mg, 0.023 mmol, 0.08 equiv.), PPh$_3$ (37.5 mg, 0.14 mmol, 0.48 equiv.), Ag$_2$O (106.4 mg, 0.46 mmol, 1.5 equiv.), 4-iodobenzotrifluoride (120.2 mg, 0.44 mmol, 1.5 equiv.), and 1,2-dimethoxyethane (6.0 mL). After filtration, the crude material was purified by column chromatography (3.0% ethyl acetate/hexanes) to afford **10aJk** (70.0 mg, 0.17 mmol, 63%) as an ocher solid; e.r. 85:15, 76% e.s. (over 2 steps from 96:4 e.r. diboronate **8**). The e.r. was determined by analysis by chiral SFC:

IF, 2mL/min, 100 bar, 50 °C, 1% → 40% MeCN over 10 min then 40% MeCN for 3 min; t_R 12.58 (major), 13.12 (minor).

^{1}H NMR (400 MHz; CDCl$_3$): 8.03 (d, *J* = 8.1 Hz, 2H, Ar**H**), 7.67 (d, *J* = 8.0 Hz, 2H, Ar**H**), 7.58 (m, 4H, Ar**H**), 7.38 (d, *J* = 7.8 Hz, 2H, Ar**H**), 7.33 (d, *J* = 7.9 Hz, 2H, Ar**H**), 4.28 (q, *J* = 6.8 Hz, 1H, C**H**-CH$_3$), 2.64 (s, 3H, C**H**$_3$), 1.71 (d, *J* = 7.1 Hz, 3H, CH-C**H**$_3$); **^{13}C NMR (100 MHz; CDCl$_3$)**: 197.7 (**C**=O), 150.2 (**C**), 145.6 (**C**), 145.4 (**C**), 138.1 (**C**), 135.9 (**C**), 129.0 (**C**H), 128.6 (**C**H), 135.0 (**C**H), 129.0 (**C**H), 128.6 (q, *J* = 32.3 Hz, (**C**), 128.3 (**C**H), 128.1 (**C**H), 127.5 (**C**H), 127.1 (**C**H), 125.5 (q, *J* = 3.8 Hz, (**C**H), 124.4 (q, *J* = 271.9 Hz, **C**F$_3$), 44.5 (**C**H), 26.7 (CO**C**H$_3$), 21.7 (CH**C**H$_3$); **^{19}F NMR (377 MHz; CDCl$_3$)**: 62.8; **IR (film) cm^{-1}:** 2994w, 1677s, 1599w, 1324w, 819w; **HRMS (EI)**: $C_{23}H_{19}F_3O$ requires 368.1388; found 368.1381; $[\alpha]_D^{20}$ = +8.0 (*c* 1.0, CH_2Cl_2); mp 132.5-134.1 °C.

The Introduction of Three Unique Aryl Groups Sequentially by Coupling at (1) the Aryl B–C Bond, (2) the Linear Achiral Aliphatic B–C Bond, and (3), the Chiral Benzylic B–C Bond Shown in Figure 4b

2,2'-(1-(3-(4,4,5,5-tetramethyl-1,3,2-dioxaborolan-2-yl)phenyl)ethane-1,2-diyl)bis(4,4,5,5-tetramethyl-1,3,2-dioxaborolane) (compound **11a**, Figure 4b)

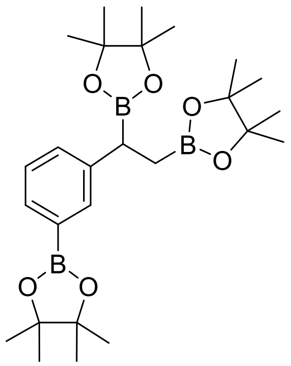

A 50mL two-necked flask containing a magnetic stirring bar was flame-dried under vacuum and filled with argon after cooling to room temperature. To the glass vessel were added 4,4,5,5-tetramethyl-2-(3-vinylphenyl)-[1,3,2]dioxaborolane (1.38 g, 6 mmol), bis(pinacolato)diboron (1.81 g, 7.2 mmol) and dry toluene (12 mL) under a steam of argon. Platinum(0)-1,3-divinyl-1,1,3,3-tetramethyldisiloxane complex solution (2% in xylene, 2.7 mL, 0.12 mmol) was added to this solution and the mixture was stirred at 50 ºC for 48 h. After cooling to room temperature, the mixture was passed through a pad of silica gel with copious washings with EtOAc (~30 mL). The filtrate was concentrated under reduced pressure. The crude product was purified by PTLC (EtOAc/hexane = 1:10) and GPC to afford **11a** (1.07 g, 37%) as a colorless oil.

^{1}H NMR (400 MHz, $CDCl_3$): 1.11 (dd, *J* = 16 Hz, 5.6 Hz, 1H), 1.18 (s, 6H), 1.20 (s, 18H), 1.32 (s, 12H), 1.37 (dd, *J* = 16 Hz, 10.8 Hz, 1H), 2.53 (dd, *J* = 10.8 Hz, 5.6 Hz, 1H), 7.23 (t, *J* = 7.6 Hz, 1H), 7.33 (d, *J* = 7.6 Hz, 1H), 7.55 (d, *J* = 7.6 Hz, 1H), 7.69 (s, 1H); **^{13}C NMR (100 MHz, $CDCl_3$)**: 24.5, 24.65, 24.69, 24.8, 24.9, 25.0, 82.9, 83.2, 83.5, 127.5, 131.0, 131.4, 134.8, 144.7; **^{11}B NMR (128 MHz, $CDCl_3$)**: 32.9; **HRMS (ESI)** *m/z* calcd for $C_{27}H_{46}O_7B_3$ $[M+MeO]^-$: 515.3528, found 515.3530.

Sequential Arylation of 11a

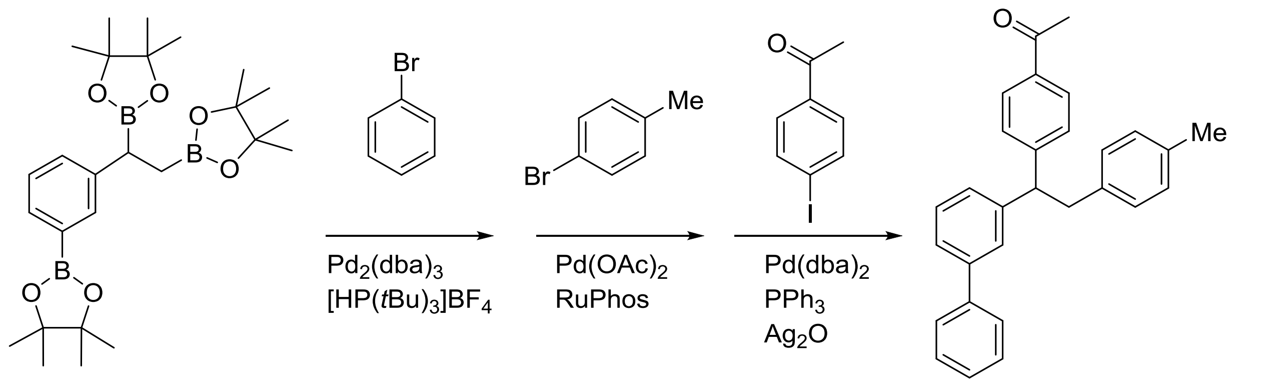

A 50 mL sealable glass vessel containing a magnetic stirring bar was flame-dried under vacuum and filled with argon after cooling to room temperature. To the glass vessel were added **11** (48.4 mg, 0.1 mmol), $Pd_2(dba)_3$ (4.6 mg, 5 μmol), P(*t*-Bu)$_3$·HBF_4 (26.1 mg, 20 μmol), and K_2CO_3 (41.5 mg, 0.3 mmol) under a stream of argon. Bromobenzene (12.5 μL, 0.12 mmol), dry toluene (200 μL) and H_2O (10 μL) were added, and then the vessel was sealed. The mixture was stirred at 80 °C for 24 h. After cooling to room temperature, reaction vessel was connected to a high vacuum line to remove excess bromobenzene and solvent for 6h. In independent experiments run not in series, complete conversion of substrate and formation of 1st arylation product were checked by ^{1}H NMR using anisole as internal standard, resulting in 92% yield of the desired product.

After the vessel was filled with argon, $Pd(OAc)_2$ (2.2 mg, 10 μmol), and RuPhos (11.2 mg, 20 μmol) were added under a stream of argon along with *p*-tolyl bromide (15.2 μL, 0.15 mmol), dry toluene (700 μL) and H_2O (1.4 mL), and then the vessel was sealed. The mixture was stirred at 80 °C for 24 h. After cooling to room temperature, the mixture was passed through a pad of silica gel with copious washings with EtOAc (~10 mL). The filtrate was concentrated under reduced pressure. In independent experiments run not in series, complete conversion of substrate and formation of 2nd arylation product were checked by ^{1}H NMR using anisole as internal standard, the desired product was obtained in 70% yield.The obtained crude mixture was

transferred to dried 10 mL sealable glass vessel containing a magnetic stirring bar and filled with argon. To the glass vessel were added *p*-iodoacetophenone (37 mg, 0.15 mmol), $Pd(PPh_3)_4$ (9.2 mg, 8 μmol), PPh_3 (12.6 mg, 48 μmol), Ag_2O (34.8 mg, 0.15 mmol), and dry THF (2.0 mL) under a stream of argon and then the vessel was sealed. The mixture was stirred at 70 °C for 24 h. After cooling to room temperature, the mixture was passed through a pad of silica gel with copious washings with EtOAc (~15 mL). The filtrate was concentrated under reduced pressure. The crude product was purified by PTLC (EtOAc/hexane = 1:20) to afford **12aBa** (12.5 mg, 32%) as colorless oil.

^{1}H NMR (400 MHz, $CDCl_3$): 2.27 (s, 3H), 2.55 (s, 3H), 3.33-3.43 (m, 2H), 4.34 (t, J = 8.0 Hz, 1H), 6.91 (d, J = 8.0 Hz, 2H), 6.99 (d, J = 8.0 Hz, 2H), 7.19 (d, J = 7.6 Hz, 1H), 7.31-7.36 (m, 4H), 7.39-7.43 (m, 4H), 7.48-7.51 (m, 2H), 7.84 (d, J = 8.4 Hz, 1H); **^{13}C NMR (100 MHz, $CDCl_3$)**: 21.0, 26.6, 41.4, 53.2, 125.4, 126.8, 127.0, 127.2, 127.3, 128.4, 128.5, 128.7, 128.88, 128.92, 129.2, 135.3, 135.6, 136.5, 141.2, 141.4, 144.2, 149.9, 197.8; **HRMS (ESI)** *m/z* calcd for $C_{29}H_{27}O$ $[M+H]^+$: 391.2062, found 391.2072.

Procedures for the Orthogonal Coupling in the Synthesis of Phosphodiesterase Inhibitor CDP 840 Shown in Figure 4c

2-(Cyclopentyloxy)-1-methoxy-4-vinylbenzene (compound **13**, Figure 4c)

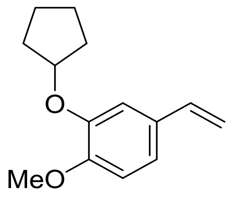

The title compound was synthesized according to a modified literature procedure.[12] To a suspension of methyltriphenylphosphonium bromide (9.61 g, 26.9 mmol, 1.3 equiv.) in THF (54 mL) at 0 °C was added a solution of potassium *tert*-butoxide (3.02 g, 26.89 mmol, 1.3 equiv.) in THF (26 mL) dropwise. The flask was rinsed with 2 x 1 mL of THF to ensure complete transfer. After stirring at room temperature for 10 minutes, the solution was cooled to 0 °C and a solution of 3-(cyclopentyloxy)-4-methoxybenzaldehyde (4.56 g, 20.7 mmol, 1.0 equiv.) in THF (10 mL) was added dropwise. The flask was rinsed with 3 x 2 mL of THF to ensure complete transfer. The resultant mixture was stirred at room temperature for 16 hours. After concentration *in vacuo*, the crude material was purified by column chromatography (10% ethyl acetate/hexanes) to afford **13** (4.48 g, 20.5 mmol, 99%) a yellow oil.

^{1}H NMR (400 MHz, $CDCl_3$): 6.97 (d, *J* = 2.0 Hz, 1H, Ar**H**), 6.93 (dd, *J* = 8.2 Hz, 1.9 Hz, 1H, Ar**H**), 6.82 (d, *J* = 8.2 Hz, 1H, Ar**H**), 6.64 (dd, *J* = 17.5 Hz, 10.8 Hz, 1H, C**H**=CH_2), 5.59 (dd, *J* = 17.5 Hz, 0.8 Hz 1H, CH=C**H**$_2$-(Z)), 5.13 (dd, *J* = 10.8 Hz, 0.8 Hz 1H, CH=C**H**$_2$-(E)), 4.85-4.75 (m, 1H, OC**H**-$(CH_2)_2$), 3.96 (s, 3H, OC**H**$_3$), 1.88 (m, 6H, Cp CH**H**/C**H**$_2$), 1.62 (m, *2*H, Cp CH**H**/C**H**$_2$); **^{13}C NMR (100 MHz, $CDCl_3$)**: 150.2 (**C**), 147.8 (**C**), 136.7 (**C**H), 130.8 (**C**), 119.4 (**C**H), 112.7 (**C**H), 111.9 (**C**H), 111.7 (**C**H_2), 80.6 (**C**H), 56.1 (**C**H_3), 32.9 (**C**H_2), 24.1 (**C**H_2); **IR (film) cm^{-1}:** 2956w, 1628w, 1508w, 1134w, 987w; **HRMS (EI)**: $C_{26}H_{42}B_2O_6$ requires 218.1307; found 218.1301.

(S)-2,2'-(1-(3-(cyclopentyloxy)-4-methoxyphenyl)ethane-1,2-diyl)bis(4,4,5,5-tetramethyl-1,3,2-dioxaborolane) (compound **16**, Figure 4c)

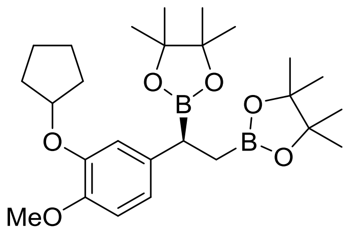

The title compound, diboronate **16**, was prepared according to Nishiyama and co-workers[5]: B_2Pin_2 (394 mg, 1.55 mmol, 1.2 eq), NaO^{t}Bu (6.2 mg, 0.065 mmol, 0.05 eq) and [Rh{(*R*,*R*)-Phebox-*i*Pr}$OAc_2(H_2O)$][6] (7.0 mg, 0.013 mmol, 0.01 eq) were placed in a dry Schlenk flask under argon. THF (1.3 mL) was added followed by a solution of **13** (282 mg, 6.10 mmol, 1 eq) in THF (1.3 mL) and the reaction was immediately placed into a preheated oil bath at 60 °C and stirred for 1 h. The reaction mixture was cooled and aq. sat. NH_4Cl (15 mL) and Et_2O (15 mL) were added. The layers were separated and the aqueous phase was extracted with Et_2O (2 × 15 mL). The combined organics were washed with brine (15 mL), dried ($MgSO_4$), filtered and concentrated *in vacuo*. Purification by column chromatography (pentane/EtOAc (10:1)) gave **5a** as a white solid (435 mg, 71% yield); e.r. 99:1. The e.r. was determined by oxidation to the diol (by **GP1**) and acylation followed by analysis by chiral SFC:

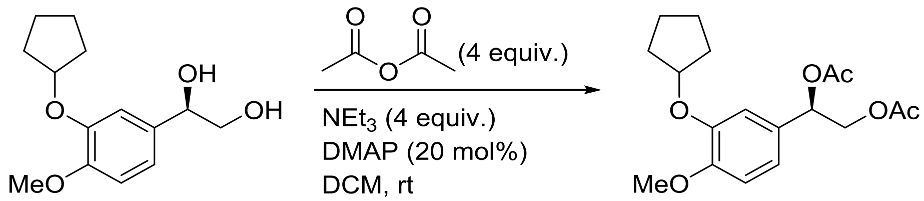

IC, 2mL/min, 100 bar, 50 °C, 1% → 40% MeOH over 10 min; t_R 7.39 (minor), 7.79 (major)

^{1}H NMR (400 MHz; $CDCl_3$): 6.78 (s, 1H, Ar**H**), 6.73 (m, 2H, Ar**H**), 4.73 (q, *J* = 6.8 Hz, 1H, OC**H**-$(CH_2)_2$), 3.78 (s, 3H, OC**H**$_3$), 2.43 (dd, *J* = 11.2 Hz, 5.4 Hz, 1H, C**H**CHH'), 1.89 (m, 4H, Cp CH**H**/C**H**$_2$). 1.81 (m, 2H, Cp CH**H**/C**H**$_2$), 1.57 (m, 2H, Cp CH**H**/C**H**$_2$), 1.33 (dd, *J* = 16.0 Hz, 11.3 Hz, 1H, CHC**H**H'), 1.20 (s, 12H, C(C**H**$_3$)$_2$C(C**H**$_3$)$_2$), 1.18 (s, 6H, C(C**H**$_3$)(CH$_3$)'C(C**H**$_3$)(CH$_3$)'), 1.17 (s, 6H, C(CH$_3$)(C**H**$_3$)'C(CH$_3$)(C**H**$_3$)'), 1.08 (dd, *J* = 16.0 Hz,

5.4 Hz, 1H, CHCH**H'**); **^{13}C NMR (100 MHz; $CDCl_3$)**: 147.7 (**C**), 147.4 (**C**), 138.0 (**C**), 119.7 (**C**H), 115.2 (**C**H), 112.2 (**C**H), 83.3 (**C**-OB), 83.2 (**C**-OB), 80.2 (O**C**H), 56.3 (**C**H_3), 32.99 (**C**H_2), 32.97 (**C**H_2), 25.1 (**C**H_3), 24.88 (**C**H_3), 24.82 (**C**H_3), 24.7 (**C**H_3), 24.2 (**C**H_2), peaks not observed for **C**'s bound to B's; **^{11}B NMR (128 MHz; $CDCl_3$)**: 33.2; **IR (film) cm^{-1}:** 2975w, 1509w, 1315w, 1135w, 850w; **HRMS (EI)**: $C_{26}H_{42}B_2O_6$ requires 472.3168; found 472.3172; $[\alpha]_D^{20}$ = +28.6 (*c* 0.7, $CHCl_3$); mp 84.0-85.0 °C.

(S)-4-(2-(3-(cyclopentyloxy)-4-methoxyphenyl)-2-(4,4,5,5-tetramethyl-1,3,2-dioxaborolan-2-yl)ethyl)pyridine (compound **17**, Figure 4c)

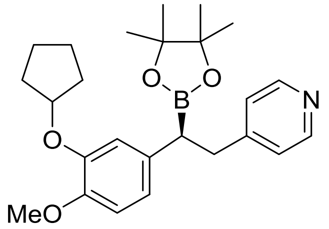

Diboronate **16** (280 mg, 0.59 mmol, 1 eq, 99:1 e.r.), $Pd(OAc)_2$ (6.7 mg, 0.030 mmol, 0.05 eq), RuPhos (27.7 mg, 0.060 mmol, 0.10 eq) and K_2CO_3 (164 mg, 1.19 mmol, 2 eq) were weighed into a Schlenk flask and placed under argon. A solution of 4-bromopyridine* (141 mg, 0.89 mmol, 1.5 eq) in DME (6 mL) was added followed by degassed water (0.6 mL). The reaction vessel was sealed and then heated at 80 °C for 16 h. The reaction mixture was cooled to room temperature, water (15 mL) and EtOAc (15 mL) were added and the layers were separated. The aqueous portion was extracted with EtOAc (2 × 15 mL) and the combined organics were washed with brine (15 mL), dried ($MgSO_4$), filtered and concentrated *in vacuo*. Purification by column chromatography (pentane/acetone (5:1)) gave **17** as a yellow oil (140 mg, 56%); e. r. was determined over two steps (see **14**).

^{1}H NMR (400 MHz, $CDCl_3$): 8.41 (brs, 2H, Ar**H**), 7.04 (d, J = 4.3 Hz, 2H, Ar**H**), 6.73 (m, 2H, Ar**H**), 6.66 (d, J = 8.1 Hz, 1H, Ar**H**), 4.71 (brs, 1H, C**H**O), 3.79 (s, 3H, OC**H**$_3$), 3.07 (dd, J = 13.3 Hz, 9.0 Hz, 1H, CHBC**H**H'), 2.89 (dd, J = 13.3 Hz, 7.7 Hz, 1H, CHBCH**H'**), 2.54 (app t, J = 8.1 Hz, 1H, C**H**B), 1.94-1.74 (m, 6H, Cp CH**H**/C**H**$_2$), 1.58 (brs, 2H, Cp CH**H**/C**H**$_2$), 1.14 (s, 6H, C(C**H**$_3$)(CH$_3$)'C(C**H**$_3$)(CH$_3$)'), 1.12 (s, 6H, C(CH$_3$)(C**H**$_3$)'C(CH$_3$)(C**H**$_3$)'); **^{13}C NMR (100 MHz, $CDCl_3$)**: 150.9 (**C**), 149.5 (**C**H), 148.3 (**C**), 147.5 (**C**), 133.9 (**C**), 124.4 (**C**H), 120.6 (**C**H), 115.7

---

* 4-Bromopyridine was freshly prepared from 4-bromopyridine hydrochloride as follows: 4-Bromopyridine hydrochloride (500 mg, 2.60 mmol) was dissolved in water (20 mL) and $NaHCO_3$ was added as a solid until the evolution of gas ceased. Et_2O (20 mL) was added and the layers were separated. The aqueous portion was extracted with Et_2O (2 × 10 mL) and the combined organics were dried (Na_2SO_4), filtered and concentrated *in vacuo* to give 4-bromopyridine as a colorless oil (328 mg, 80%). The product was used immediately without further purification.

(**C**H), 112.2 (**C**H), 83.7 (**C**-OB), 80.4 (**C**H), 56.1 (**C**H_3), 38.4 (**C**H_2), 33.0 (**C**H_2), 32.9 (**C**H_2), 24.71 (**C**H_3), 24.70 (**C**H_3), 24.16 (**C**H_2), 24.15 (**C**H_2), peak not observed for **C** bound to B; **^{11}B NMR (128 MHz, $CDCl_3$)**: 32.9; **IR (film) cm^{-1}**: 2973w, 1601w, 1510w; **HRMS (ESI$^+$)**: $C_{25}H_{35}BNO_4$ (M + H^+) requires 424.2654; found 424.2653; $[\alpha]_D^{20}$ = +60.0 (*c* 0.6, $CHCl_3$).

(R)-4-(2-(3-(cyclopentyloxy)-4-methoxyphenyl)-2-phenylethyl)pyridine (compound **14**, CDP 840, Figure 4c)

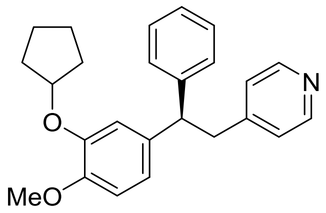

In a nitrogen filled glove box, boronic ester **17** (110 mg, 0.26 mmol, 1 eq, 99:1 e.r. (assumed based on e.r. of diboronate **16**)), iodobenzene (79.5 mg, 0.39 mmol, 1.5 eq), Pd(dba)$_2$ (12.0 mg, 0.021 mmol, 0.08 eq), PPh_3 (32.7 mg, 0.125 mmol, 0.48 eq) and Ag_2O (90 mg, 0.39 mmol, 1.5 eq) were weighed into a pressure tube and DME (5.2 mL) was added. The reaction vessel was sealed, removed from the glove box and heated at 70 °C for 16 h. The reaction mixture was cooled and filtered through a plug of silica, eluted with EtOAc (30 mL) and concentrated *in vacuo*. Purification by column chromatography (DCM/EtOAc (7:1)) gave **14** as a pale yellow oil (36 mg, 37%); e. r. 95.5:4.5, 93% e.s. (over 2 steps from diboronate **16**). The e.r. was determined by analysis by chiral SFC:

IB, 5mL/min, 3% MeOH, 100 bar, T = 35 °C; t_R 23.0 (minor), 25.4 (major).

^{1}H NMR (400 MHz, CDCl$_3$): 8.40 (brs, 2H, Ar**H**), 7.31-7.24 (m, 2H, Ar**H**), 7.23-7.15 (m, 3H, Ar**H**), 6.94 (d, *J* = 4.9 Hz, 2H, Ar**H**), 6.76 (d, *J* = 7.2 Hz, 1H, Ar**H**), 6.70 (dd, *J* = 7.2 Hz, 1.9 Hz, 1H, Ar**H**), 6.67 (d, *J* = 1.9 Hz, 1H, Ar**H**), 4.66 (m, 1H, C**H**O), 4.16 (t, *J* = 7.9 Hz, 2H, CHPhC**H**$_2$), 3.80 (s, 3H, OC**H**$_3$), 3.32 (d, *J* = 7.9 Hz, 1H, C**H**Ph), 1.89-1.69 (m, 6H, Cp CH**H**/C**H**$_2$), 1.65-1.51 (m, 2H, Cp CH**H**/C**H**$_2$); **^{13}C NMR (100 MHz, CDCl$_3$)**: 149.6 (**C**), 149.4 (**C**H), 148.9 (**C**), 147.6 (**C**), 144.1 (**C**), 136.1 (**C**), 128.6 (**C**H), 127.8 (**C**H), 126.6 (**C**H), 124.6 (**C**), 120.1 (**C**H), 115.6 (**C**H), 112.1 (**C**H), 80.6 (**C**H), 56.1 (**C**H$_3$), 51.7 (**C**H), 41.8 (**C**H$_2$), 32.9 (**C**H$_2$), 32.8 (**C**H$_2$), 24.1 (**C**H$_2$); **IR (film) cm^{-1}**: 2954w, 1598w, 1508w; **HRMS (ESI$^+$)**: $C_{25}H_{28}NO_2$ (M + H$^+$) requires 374.2115; found 374.2105; $[\alpha]_D^{20}$ = +40.0 (*c* 1.6, MeOH) {Lit.[13] $[\alpha]_D^{20}$ = +38.6 (*c* 1.0, MeOH) for e.r. 90:10}.

Data were consistent with those reported in the literature.[13]

Supplementary References

[1] Huang. W., Zhang, R., Zou, G., Tang, J. & Sun, J. An iodide/anion exchange route to benzimidazolylidene silver complexes from benzimidazolium iodide: Crystal structures of N,N-dibutylbenzimidazolylidene silver chloride, bromide, cyanide and nitrate. *J. Organomet. Chem.* **692**, 3804-3809 (2007).

[2] Gottlieb, H. E., Kotlyar, V. & Nudelman, A. NMR Chemical Shifts of Common Laboratory Solvents as Trace Impurities. *J. Org. Chem.* **62**, 7512-7515 (1997).

[3] Kliman, L. T., Mlynarski, S. N. & Morken, J. P. Pt-Catalyzed Enantioselective Diboration of Terminal Alkenes with $B_2(pin)_2$. *J. Am. Chem. Soc.* **131**, 13210-13211 (2009).

[4] Bonet, A., Pubill-Ulldemolins, C., Bo, C., Gulyas, H. & Fernández, E. Transition-metal-free diboration reaction by activation of diboron compounds with simple lewis bases. *Angew. Chem. Int. Ed.* **50**, 7158-7161 (2011).

[5] Toribatake, K. & Nishiyama, H. Asymmetric diboration of terminal alkenes with a rhodium catalyst and subsequent oxidation: Enantioselective synthesis of optically active 1,2-diols. *Angew. Chem. Int. Ed.* **52**, 11011 –11015 (2013).

[6] Prepared according to the literature: Kanazawa, Y., Tsuchiya, Y., Kobayashi, K., Shiomi, T., Itoh, J.-i., Kikuchi, M.; Yamamoto, Y. & Nishiyama, H. Asymmetric Conjugate Reduction of α,β-Unsaturated Ketones and Esters with Chiral Rhodium(2,6-bisoxazolinylphenyl) Catalysts. *Chem. Eur. J.* **12**, 63-71 (2006).

[7] Lee, Y., Jang, H. & Hoveyda, A. H. Vicinal Diboronates in High Enantiomeric Purity through Tandem Site-Selective NHC−Cu-Catalyzed Boron−Copper Additions to Terminal Alkynes. *J. Am. Chem. Soc.* **131**, 18234-18235 (2009).

[8] Prepared according to the literature: Aslam, S. N., Stevenson, P. C., Phythian, S. J., Veitch, N. C. & Hall, D. R. Synthesis of cicerfuran, an antifungal benzofuran, and some related analogues. *Tetrahedron* **62**, 4214-4226 (2006).

[9] Ganić. A. & Pfaltz. A. Iridium-Catalyzed Enantioselective Hydrogenation of Alkenylboronic Esters *Chem. Eur. J.* **18**, 6724-6728 (2012).

[10] Baltus, C. B., Chuckowree, I. S., Press, N. J., Day, I. J., Coles, S. J., Tizzard, G. J. & Spencer, J. Olefin cross-metathesis/Suzuki-Miyaura reactions on vinylphenylboronic acid pinacol esters. *Tet. Lett.* **54**, 1211-1217 (2013).

[11] Chen, A. C., Ren, L. & Crudden, C. M. Catalytic asymmetric carbon-carbon bond forming reactions: preparation of optically enriched 2-aryl propionic acids by a catalytic asymmetric hydroboration-homologation sequence. *Chem. Commun.* **7**, 611-612 (1999).

[12] Honda, T., Ishikawa, F., Kanai, K., Sato, S., Kato, D. & Tominaga, H. Chiral synthesis of phosphodiesterase inhibitor, (*R*)-(−)-rolipram, by means of enantioselective deprotonation strategy. *Heterocycles* **42**, 109-112 (1996)

[13] Aggarwal, V. K., Bae, I., Lee, H.-Y., Richardson, J. & Williams, D. T. Sulfur-Ylide-Mediated Synthesis of Functionalized and Trisubstituted Epoxides with High Enantioselectivity; Application to the Synthesis of CDP-840. *Angew. Chem. Int. Ed.* **42**, 3274-3278 (2003).
